# Rhodium-catalyzed intermolecular enantioselective Alder–ene type reaction of cyclopentenes with silylacetylenes

**DOI:** 10.1038/s41467-021-26955-9

**Published:** 2021-11-16

**Authors:** Dongquan Zhang, Miaomiao Li, Jiajia Li, Aijun Lin, Hequan Yao

**Affiliations:** State Key Laboratory of Natural Medicines (SKLNM) and Department of Medicinal Chemistry, School of Pharmacy, China Pharmaceutical University, Nanjing, P. R. China

**Keywords:** Asymmetric catalysis, Synthetic chemistry methodology

## Abstract

The Alder–ene type reaction between alkenes and alkynes provides an efficient and atom-economic method for the construction of C-C bond, which has been widely employed in the synthesis of natural products and other functional molecules. The intramolecular enantioselective Alder-ene cycloisomerization reactions of 1,n-enynes have been extensively investigated. However, the intermolecular asymmetric version has not been reported, and remains a challenging task. Herein, we describe a rhodium-catalyzed intermolecular enantioselective Alder-ene type reaction of cyclopentenes with silylacetylenes. A variety of chiral (E)-vinylsilane tethered cyclopentenes bearing one quaternary carbon and one tertiary carbon stereocenters are achieved in high yields and enantioselectivities. The reaction undergoes carbonyl-directed migratory insertion, β-H elimination and desymmetrization of prochiral cyclopentenes processes.

## Abstract (web summary)

The Alder–ene type reaction is a synthetically useful, redox-neutral method to rearrange carbon–carbon bonds, but has only been demonstrated intramolecularly. Here the authors present a method to perform the Alder–ene type reaction intermolecularly with cylcopentenes and silylynes, proceeding enantioselectively via rhodium catalysis.

## Introduction

**Fig. 1:**
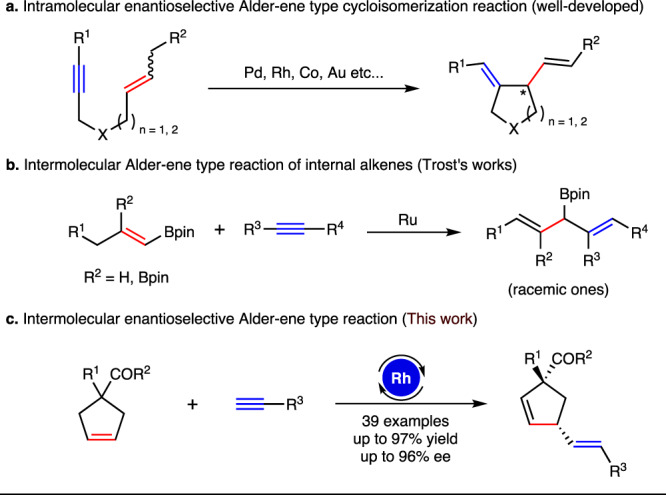
Transition metal-catalyzed Alder-ene type reaction. **a** Intramolecular enantioselective Alder-ene type cycloisomerization reaction (well-developed). **b** Intermolecular Alder-ene type reaction of internal alkenes (Trost’s work). **c** Intermolecular enantioselective Alder-ene type reaction (this work).

The production of synthetically valuable products from readily accessible substrates in a redox-neutral, atom- and step-economical approach is a long-standing goal in organic synthesis. The Alder-ene type reaction comprises a particularly efficient subset of this target^1–6^, which provides an appealing tool to construct C–C bond. Over the past decades, transition metal-catalyzed intramolecular enantioselective Alder-ene type cycloisomerization reactions of 1,6-enynes or 1,7-enynes have been extensively investigated for the rapid assembly of chiral five- or six membered carbo- and hetero-cyclic frameworks (Fig. 1a)^7–17^. However, the intermolecular Alder-ene reactions were mainly confined to less-hindered terminal alkenes^18–25^, and the studies on internal alkenes have rarely been reported^26,27^. Very recently, Trost and coworkers successfully implemented ruthenium-catalyzed intermolecular alkene–alkyne coupling reactions with well-designed vinyl boronate compounds to synthesize boron-functionalized 1,4-dienes (Fig. 1b)^28,29^, in which the boron substituent played a vital role to facilitate the transformations. However, the intermolecular enantioselective Alder-ene type reaction remains an unexplored territory.

All-carbon chiral quaternary stereocenters are fundamental structural motifs present in natural products and pharmaceuticals, which could improve the metabolic stability and target selectivity of biologically active compounds^30^. However, the synthesis of chiral quaternary carbon centers is a challenging task, especially those that are not formed at the direct reaction site^31–41^. Asymmetric desymmetrization of prochiral compounds or meso-compounds offers a commendable synthetic tool for achieving this objective^42–54^.

Herein, we describe an asymmetric desymmetrization of prochiral cyclopentenes with silylacetylenes enabled by rhodium-catalyzed intermolecular enantioselective Alder-ene type reaction. This protocol allows access to chiral (E)-vinylsilane tethered cyclopentenes bearing one quaternary carbon and one tertiary carbon stereocenters in high yields and enantioselectivities (Fig. 1c).

## Results

### Reaction optimization

We commenced our studies with the employment of N,1-diphenylcyclopent-3-ene-1-carboxamide **1a** and triisopropylsilylyne **2a** as the model substrates. After considerable screening of the reaction parameters (see the Supplementary Table 1 for details), the desired product **3a** was obtained in 96% yield and 95% ee with [Rh(COD)OMe]_2_ as the catalyst, phosphoramidite **L6** as the ligand, PhMe_2_CCO_2_H and NaBARF as the additives in DCM at 80 °C (Table 1, entry 1). The P,P-ligands **L1**, **L2** and N,P-ligand **L3** inhibited the transformation (entry 2). Phosphoramidite ligands **L4** and **L5** performed this reaction in less efficiency (entries 3 and 4). Rh(COD)_2_OTf and [Rh(COD)Cl]_2_ gave inferior results compared with [Rh(COD)OMe]_2_, [Cp*RhCl_2_]_2_ and Pd(dba)_2_ delivered trace amount yield of product **3a** (entries 5–8). AcOH and PhCO_2_H offered **3a** in diminished enantioselectivities (entries 9 and 10), and no product was detected in the presence of TsOH (entry 11). Using other additives, such as AgSbF_6_ and AgPF_6_ could not perform this transformation as well as NaBARF (entries 12 and 13). Conducting the reaction in CHCl_3_ and toluene led to lower yields and enantioselectivities (entries 14 and 15), and the reaction was completely suppressed in THF (entry 16).

**Table 1:** Effect of reaction parameters^a^.


Entry	Deviation of standard conditions	Yield of 3a (%)^b^	ee (%)^c^
1	None	96	95
2	**L1-3** instead of **L6**	trace	–
3	**L4** instead of **L6**	44	76
4	**L5** instead of **L6**	77	84
5	Rh(COD)_2_OTf instead of [Rh(COD)OMe]_2_	82	92
6	[Rh(COD)Cl]_2_ instead of [Rh(COD)OMe]_2_	90	90
7	[Cp*RhCl_2_]_2_ instead of [Rh(COD)OMe]_2_	trace	–
8	Pd(dba)_2_ instead of [Rh(COD)OMe]_2_	trace	–
9	AcOH instead of PhMe_2_CCO_2_H	55	69
10	PhCO_2_H instead of PhMe_2_CCO_2_H	95	68
11	TsOH instead of PhMe_2_CCO_2_H	n.d.	–
12	AgSbF_6_ instead of NaBARF	87	92
13	AgPF_6_ instead of NaBARF	40	60
14	CHCl_3_ instead of DCM	84	88
15	toluene instead of DCM	85	91
16	THF instead of DCM	n.d.	–

^a^Standard conditions: **1a** (0.10 mmol), **2a** (0.30 mmol), [Rh(COD)OMe]_2_ (2.5 mol%), **L6** (6.0 mol%), NaBARF (10 mol%), PhMe_2_CCO_2_H (60 mol%) in 1.0 mL DCM, 80 °C, 36 h, under argon. TIPS = Si^*i*^Pr_3_.

^b^Isolated yield, d.r. > 20:1, E/Z > 20:1, determined by ^1^H NMR analysis.

^c^Determined by HPLC analysis on a chiral stationary phase.

### Substrate scope

**Fig. 2:**
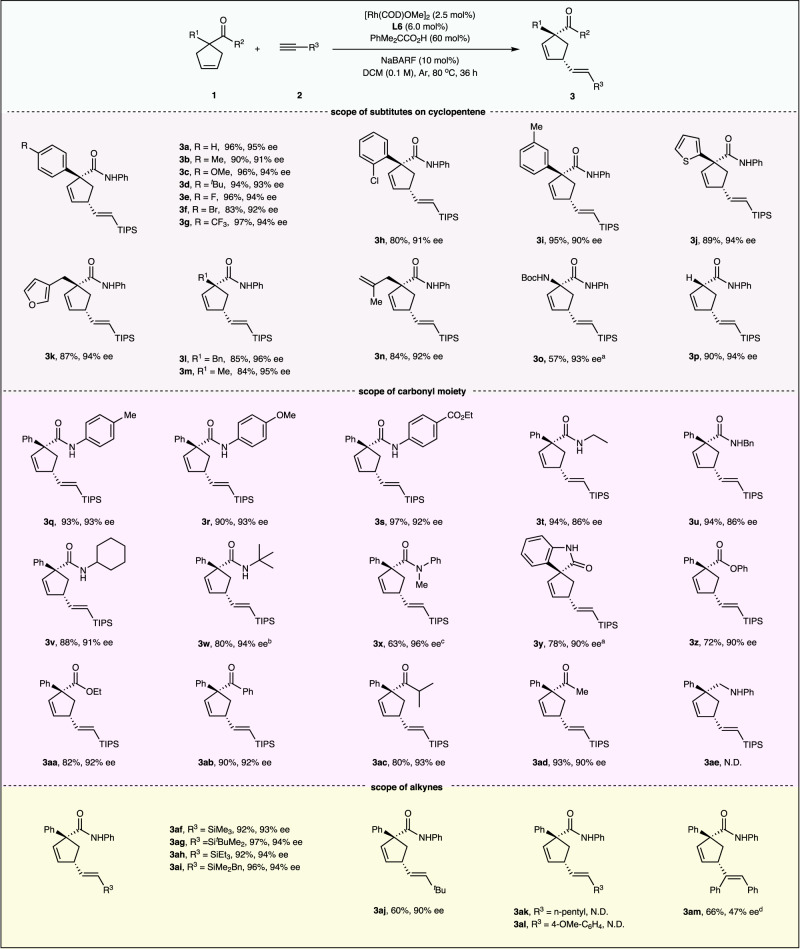
Substrate scope. Reaction conditions: **1a** (0.10 mmol), **2a** (0.30 mmol), [Rh(COD)OMe]_2_ (2.5 mol%), **L6** (6.0 mol%), PhMe_2_CCO_2_H (60 mol%), NaBARF (10 mol%), DCM (1.0 mL), 80 ^o^C, 36 h, under argon. Isolated yields, d.r. > 20:1, E/Z > 20:1, determined by ^1^H NMR analysis. The ee values were determined by chiral HPLC analysis. ^a^[Rh(COD)OMe]_2_ (5.0 mol%)_,_ 72 h. ^b^72 h. ^c^[Rh(COD)OMe]_2_ (5.0 mol%). ^d^[Rh(COD)Cl]_2_ (5.0 mol%), **L5** (12 mol%), Ph_3_CCO_2_H (60 mol%), NaBARF (20 mol%), d.r. > 20:1, Z/E > 20:1.

With the optimized reaction conditions in hand, we then explored the generality of this rhodium-catalyzed intermolecular enantioselective Alder-ene type reaction (Fig. 2). Various aryl and heteroaryl substituted cyclopentenes (see the Supplementary Methods for details) performed the reactions well, affording the products **3b-3k** in 90–94% ee. Replacing the aryl groups with a benzyl group or an alkyl group delivered the products **3l** and **3m** in 96 and 95% ee. Notably, the substrate **1n** containing two alkene moieties, performed the reaction selectively with the endocyclic double bond to give the product **3n** in 84% yield with 92% ee. The amino substituted cyclopentene **1o** was also suitable substrate for this transfomation, furnishing chiral α-amino acid derivative **3o** in 93% ee. N-benzylcyclopent-3-ene-1-carboxamide (R^1^ = H) offered the desired product **3p** in 90% yield and 94% ee.

Subsequently, the compatibilities of amide moiety were investigated, and the products **3q-3w** with aryl and alkyl groups were achieved in 86–94% ee. The substrate **1x** with tertiary amide group gave **3x** in 63% yield with 96% ee under slightly modified conditions. Besides, the enantioenriched spirolactam **3y** could also be synthesized in 78% yield and 90% ee. Ester or ketone groups substituted cyclopentenes proceeded this reaction efficiently, affording the products **3z-3ad** in 72–93% yields with 90–93% ee. When substrate **1ae** was subjected to the standard reaction conditions, no desired product **3ae** was detected.

After checking the character of cyclopentenes, we then turned our attention to the scope of alkynes. The reactions proceeded smoothly with diverse silylacetylenes, delivering products **3af-3ai** in 93–94% ee. In addition, sterically hindered alkyl alkyne was also proved to be qualified for this reaction, as demonstrated by the formation of product **3aj** with 90% ee. Unfortunately, simple alkyl and aryl substituted acetylenes were not yet compatible with the reaction system to give the desired products **3ak** and **3al**. The 1-heptyne caused the reaction to become confusing, 4-ethynylanisole offered a branched byproduct in a very low yield (see Supplementary Note 1 for details). The internal alkyne biphenylacetylene could participate in this transformation to afford product **3am** in 66% yield with 47% ee. The absolute configuration of the product was confirmed by the X-ray analysis of the compound from iododesilylation of **3f** (see Supplementary Note 2 for details).

### Gram-scale experiment and further transformations of the products

**Fig. 3:**
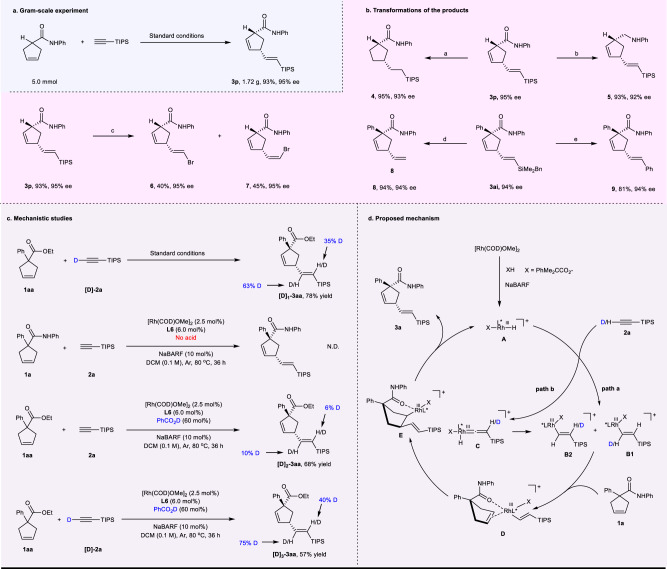
Further studies on the reaction. **a** Gram-scale experiment. **b** Transformations of the products. ^a^Pd/C (10 wt %), B_2_(OH)_4_, H_2_O, DCM, rt. ^b^LiAlH_4_, 1,4-dioxane, 0 ^o^C-80 ^o^C, 15 h. ^c^NBS, Ag_2_CO_3_, HFIP, rt, 2 h. ^d^TFA, DCM, 60 ^o^C, 12 h. ^e^Pd(dba)_2_, TBAF·3H_2_O, Iodobenzene, THF, rt, 24 h. **c** Mechanistic studies. **d** Proposed mechanism.

To demonstrate the synthetic utility of this reaction, a gram-scale experiment (Fig. 3a) and further transformations of the products **3p** and **3ai** were carried out (Fig. 3b). Product **3p** (1.72 g) could be synthesized in 93% yield and 95% ee on a 5.0 mmol scale under the standard conditions. A palladium-catalyzed hydrogenation reaction converted **3p** to compound **4** in 95% yield. The selective reduction of the amide group of **3p** using LiAlH_4_ afforded compound **5** in 93% yield. Moreover, compound **3p** could be decorated with NBS to give bromination compounds **6** in 40% yield and **7** in 45% yield, respectively. The desilylation of **3ai** with TFA offered compound **8** in 94% yield. Arylated product **9** could be constructed through palladium-catalyzed Hiyama cross-coupling reaction, maintaining the E/Z-stereochemistry and enantioselectivity.

### Mechanistic investigations

To gain insight into the mechanism of this reaction, several control experiments and deuterium-labeling experiments were carried out (Fig. 3c). When **[D]-2a** was employed, the reaction resulted in **[D]**_**1**_**-3aa** with 63% D atom at β-position to the TIPS group and 35% D atom at α-position. This result implied that a metal vinylidene intermediate could be involved^55–57^. The reaction was drastically prohibited in the absence of the acid. Conducting the reaction with PhCO_2_D provided **[D]**_**2**_**-3aa** with 10% D atom at β-position to the TIPS group and 6% D atom at α-position, which indicated that the acid may work with the rhodium(I) to catalyze this reaction by the formation of RhH species. The generation of product **[D]**_**3**_**-3aa** from **[D]-2a** with PhCO_2_D further supported the hypothesis.

Based on the above experiment results and previous works^45–47,55–60^, a possible reaction pathway is depicted in Fig. 3d with **1a** and **2a** as the model substrates. Oxidative addition of rhodium(I) into PhMe_2_CCO_2_H generates RhH species **A**, and the presence of NaBARF plays a pivotal role for the generation and stabilization of this cationic rhodium complex^59,60^. Regioselective alkyne insertion into the species **A** forms vinylrhodium intermediate **B1** directly (path a). On the other hand, η^2^-coordination of the alkyne to the species **A** followed by rearrangement generates an η^1^-vinylidenerhodium species **C**^55–57^, which could produce intermediate **B2** via migratory insertion (path b). Coordination of **B1** or **B2** to the double bond of **1a** directed by carbonyl group forms the rhodium(III) intermediate **D**^45–47,58^, which undergoes syn-migratory insertion to afford the intermediate **E**. Subsequent β-hydride elimination of **E** offers the desired product **3a**.

## Discussion

In conclusion, we have described a rhodium-catalyzed intermolecular enantioselective Alder-ene/desymmetrization reaction of prochiral cyclopentenes with alkynes directed by carbonyl group. This method provides a practical route for the synthesis of chiral (E)-vinylsilane tethered cyclopentenes bearing one quaternary carbon and one tertiary carbon stereocenters in high efficiency and atom-economy. Further studies on the reaction mechanism and expansion of the asymmetric intermolecular Alder-ene type reactions are underway in our laboratory.

## Methods

### General procedure for the rhodium-catalyzed intermolecular enantioselective Alder-ene type reaction of cyclopentenes with silylacetylenes

To an oven-dried 10-mL Schlenk tube equipped with a teflon-coated magnetic stir bar was added **1a** (26.3 mg, 0.100 mmol, 1.00 equiv), [Rh(COD)OMe]_2_ (1.2 mg, 2.5 mol%), **L6** (2.7 mg, 6.0 mol%), NaBARF (8.9 mg, 10 mol%) and PhMe_2_CCO_2_H (10 mg, 60 mol%). The vial was thoroughly flushed with argon, and **2a** (54.7 mg, 0.600 mmol, 3.00 equiv), as well as DCM (1.0 mL) was added under argon atmosphere. Then the reaction mixture was stirred at 80 °C for 36 h. After the reaction vessel was cooled to room temperature, the solution was concentrated in vacuum and purified by careful chromatography on silica gel (PE/EA = 50/1) to afford the desired product **3a**.

## Supplementary information

SUPPLEMENTARY INFO

## Data Availability

All data generated and analyzed in this study are provided in this article and its Supplementary Information, and also available from the corresponding authors upon request. The X-ray crystallographic coordinate for structure **10** reported in this study has been deposited at the Cambridge Crystallographic Data Centre (CCDC), under deposition numbers 2064563. These data could be obtained free of charge from The Cambridge Crystallographic Data Centre via www.ccdc.cam.ac.uk/data_request/cif. Source data are provided with this paper.

## Source data

Source Data
