# Supplementary material for: Rhodium-catalyzed intermolecular enantioselective Alder–ene type reaction of cyclopentenes with silylacetylenes
Source: Nat Commun. 2021 Nov 16;12:6627. doi: 10.1038/s41467-021-26955-9 (PMC8595345; doi:10.1038/s41467-021-26955-9)
Supplement: Supplementary file 1 — SUPPLEMENTARY INFO [file 41467_2021_26955_MOESM1_ESM.pdf]

Supplementary Information

Rhodium-catalyzed intermolecular enantioselective Alder–ene type reaction of cyclopentenes with silylacetylenes

Zhang et al

Supplementary Methods

1. General Information

^1H, ^{13}C and ^{19}F NMR spectra were collected on a 300 spectrometer using $CDCl_3$ or DMSO-d_6 as solvent. Chemical shifts of ^1H NMR were recorded in parts per million (ppm, δ) relative to tetramethylsilane (δ = 0.00 ppm). Data are reported as follows: chemical shift in ppm (δ), multiplicity (s = singlet, d = doublet, t = triplet, q = quartet, dd = double doublet, brs = broad singlet, m = multiplet), coupling constant (Hz), and integration. High Resolution Mass measurement was performed with Electron Spray Ionization (ESI) method on a Q-TOF mass spectrometer operating in positive-ion mode. Melting point (m.p.) was measured on a microscopic melting point apparatus. Optical rotations were measured on an automatic polarimeter with $[\alpha]_D^{20}$ values reported in degrees; concentration (c) is in g/100 mL. The enantiomeric excess (ee) was determined by HPLC analysis on Agilent 1260 Infinity II Prime using Daicel CHIRALPAK® column IC-3, IA-3, OD-H, AD-H and OJ-H. X-ray diffraction analyses were carried out on a microcrystalline powder using a Rigaku Oxford Diffraction XtaLAB Synergy-S diffractometer using Mo radiation (λ = 0.71073). PE refers to petroleum ether (b.p. 60–90 ℃) and EA refers to ethyl acetate. Flash column chromatography was carried out using commercially available 200–300 mesh under pressure unless otherwise indicated. Gradient flash chromatography was conducted eluting with PE/EA. DCM was distilled from CaH_2. All other starting materials and solvents were commercially available and were used without further purification unless otherwise stated.

2. Preparation of Substrates 1

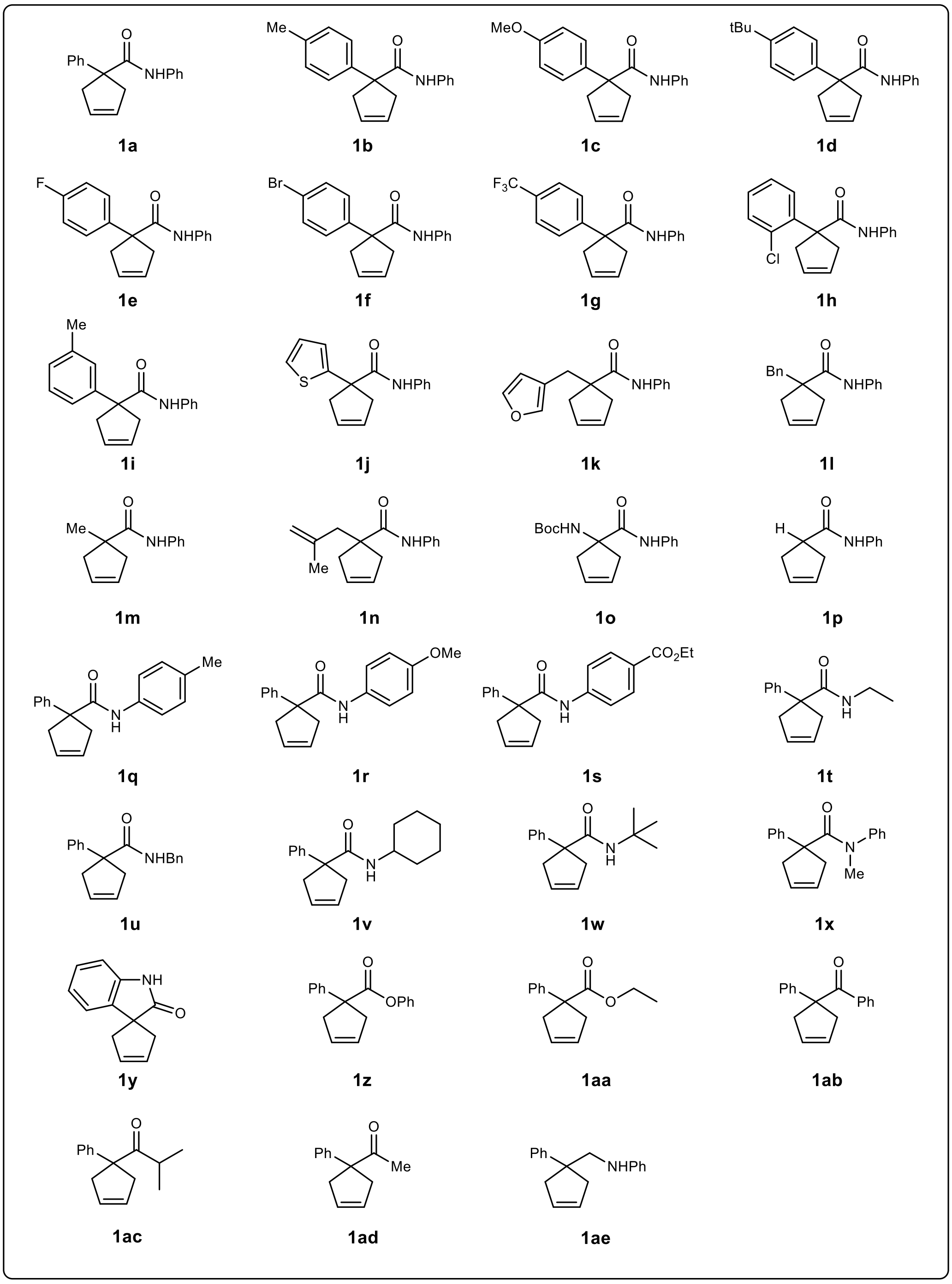

Supplementary Figure 1. Structures of Substrates **1**

General procedure for the synthesis of 1a-1j, 1p-1x, 1z-1ad[1,2] (synthesis of **1a** is used as an example: R = OEt)**:**

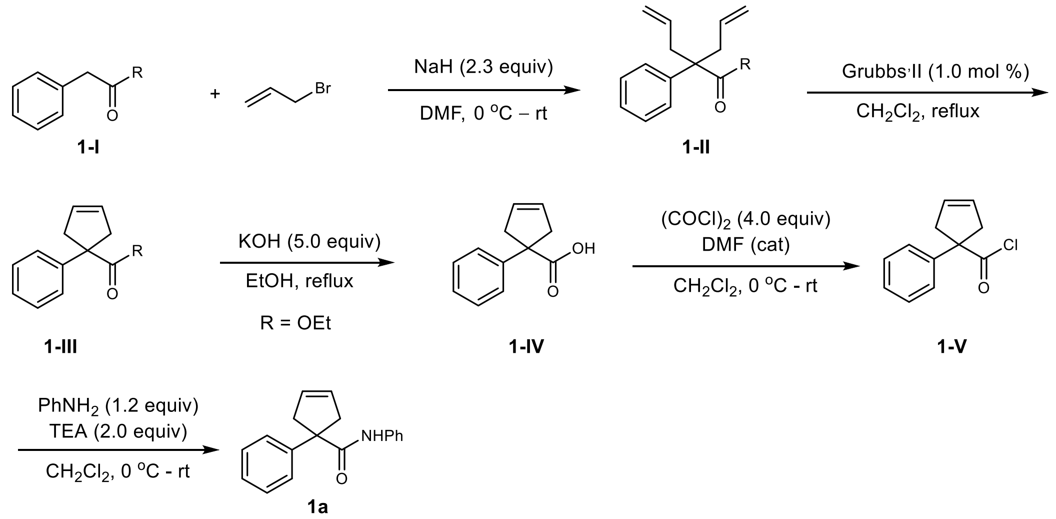

To a suspension of NaH (3.7 g, 92 mmol, 2.3 equiv) in DMF (100 mL) at 0 °C was added a solution of **1-I** (6.6 g, 40 mmol, 1.0 equiv) in DMF (20 mL) slowly, then the mixture was stirred at 0 °C for 30 min. Allylbromide (8.3 mL, 96 mmol, 2.4 equiv) was added and the mixture was stirred at roomtemperature for 4 h. After cooling to 0 °C, saturated NH_4Cl solution was added slowly to quench the reaction. Then the reaction mixture was extracted with ethyl acetate (100 mL x 3). The combined organic extracts were washed with brine and dried over Na_2SO_4. After concentrating, the residue was used in the next step without further purification.

To a solution of **1-II** (2.4 g, 10 mmol, 1.0 equiv) in CH_2Cl_2 (40 mL) was added Grubbs' catalyst II (85 mg, 0.10 mmol, 1.0 mol %), then the mixture was refluxed under Ar overnight. After concentrating, the residue was purified by flash chromatography (PE/EA = 100/1) to give **1-III** (2.0 g, 94%).

To a solution of **1-III** (1.7 g, 8.0 mmol, 1.0 equiv) in ethanol (30 mL) was added KOH (2.3 g, 40 mmol, 5.0 equiv), then the mixture was refluxed until the complete consumption of **1-III**. Ethanol was removed under reduced pressure, the residue was dissolved in minimal amount of water, then extracted with CH_2Cl_2 (10 mL x 3) and discarded. The aqueous solution was then cooled to 0 °C and was acidified with HCl (3 M) carefully. The acidic aqueous solution was extracted with CH_2Cl_2. The CH_2Cl_2 extracts were dried over Na_2SO_4 and concentrated. The residue was used in the next step without further purification.

To a solution of **1-IV** (0.94 g, 5.0 mmol, 1.0 equiv) in CH_2Cl_2 (20 mL) at 0 °C was added $(COCl)_2$ (1.7 mL, 20 mmol, 4.0 equiv) slowly, followed by 2 drops of DMF. Then the mixture was allowed to stir at 0 °C for 0.5 h and at rt for 1 h. Solvents were removed in vacuum and the residue was used in the next step directly.

To a solution of **1-V** in CH_2Cl_2 (40 mL) at 0 °C were added slowly aniline (0.55 mL, 6.0 mmol, 1.2 equiv) and triethylamine (1.4 mL, 10 mmol, 2.0 equiv) sequentially.

After stirring at rt overnight, water was added. The aqueous phase was extracted with CH_2Cl_2. The combined organic extracts were washed with diluted HCl (1 M) and brine. After drying over Na_2SO_4 and concentrating, the residue was purified by flash chromatography (PE/EA = 30/1) to give the corresponding products (1.1 g, 85%).

General procedure for the synthesis of 1k-1n[1] (synthesis of **1m** is used as an example)**:**

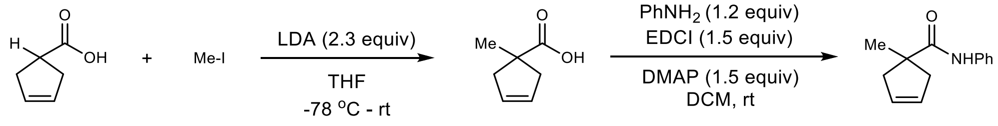

To a solution of **1-VI** (1.1 g, 10 mmol, 1.0 equiv) in THF (40 mL) cooled at -78 ℃ under argon atmosphere was added LDA (12 ml, 2.0 M solution in THF, 2.3 equiv) dropwise via a syringe. The resultant mixture was slowly rised to room temperature and stir for the total of 12 h. After that, the mixture was cooled to - 20 ^{o}C – - 40 ^{o}C and iodomethane (0.75 mL, 12 mmol, 1.2 equiv) was added dropwise. The resultant mixture was slowly rised to room temperature and stirred for the total of 18 h. The mixture was quenched with dilute HCl (3 M) and extracted with ethyl acetate (25 mL x 3). The combined organic layers were dried over Na_2SO_4 and concentrated under reduced pressure. The residue was purified by flash chromatography (PE/EA = 5/1) to give the corresponding product (1.1 g, 83%).

To a solution of **1-VII** (0.63 g, 5.0 mmol, 1.0 equiv) and DMAP (0.92 g, 7.5 mmol, 1.5 equiv) in CH_2Cl_2 (20 mL) at 0 ^{o}C were added aniline (0.55 mL, 6.0 mmol, 1.2 equiv) and EDCI (1.4 g, 7.5 mmol, 1.5 equiv) sequentially, then the mixture was stirred at rt overnight. The reaction mixture was quenched by the addition of water (50 mL) and extracted with CH_2Cl_2 (50 mL x 2). The combined organic extracts were washed with HCl (1 M) and dried over Na_2SO_4 and concentrated under reduced pressure, the residue was purified by flash chromatography (PE/EA = 30/1) to give the corresponding product (0.88 g, 87%).

General procedure for the synthesis of 1o

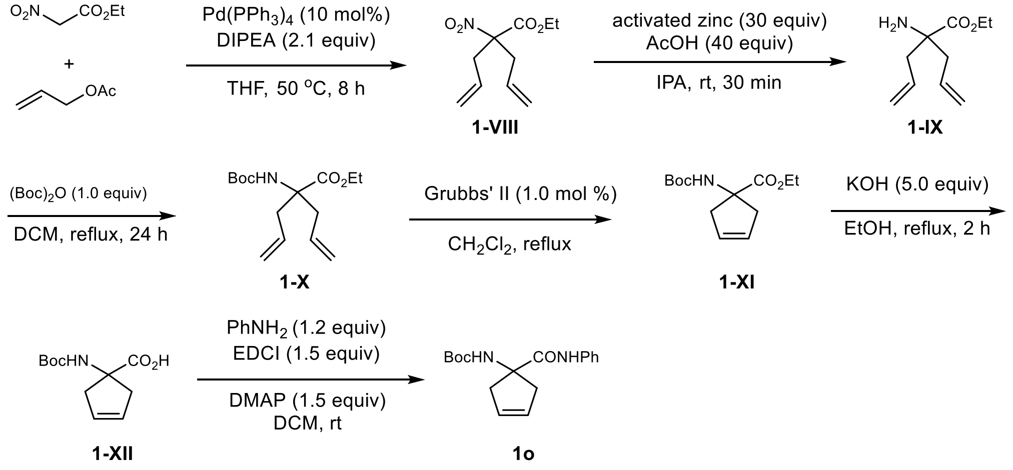

To a solution of Ethyl-2-nitroacetate (4.7 g, 35 mmol, 1.0 equiv) in dry THF (75 mL) were added Allylacetate (7.4 g, 74 mmol, 2.1 equiv) and $Pd(PPh_3)_4$ (4.0 g, 3.5 mmol, 10 mol%). After stirring for 15 minutes, DIPEA (9.5 g, 74 mmol, 2.1 equiv) was added and the reaction mixture was stirred under Ar atmosphere at 50 ℃ for 8 h. On completion of the reaction, the reaction mixture was filtered over celite-bed and washed with THF (100 mL × 2). The filterate was concentrated under reduced pressure, the residue obtained was dissolved in DCM (75 mL) and washed with $NaHCO_3$ aq. solution (75 mL). The aq. layer was extracted with DCM (75 mL × 2). The combined organic layers were dried on anhydrous Na_2SO_4 and concentrated under reduced pressure. The product obtained was purified by flash column chromatography (PE/EA = 20/1) to give **1-VIII** (7.2 g, 96%).

To a solution of Ethyl-2,2-bis(allyl)-2-nitroacetate **1- VIII** (4.26 g, 20.0 mmol, 1.00 equiv) in isopropanol (100 mL) in a 500 mL round bottom flask, activated zinc (600 mmol, 39.2 g, 30.0 equiv) was added. Then AcOH (800 mmol, 45.8 mL, 40.0 equiv) was added slowly (in 10 minutes) and the reaction mixture was stirred at rt for 1 h. The progress of the reaction was checked by TLC. On completion of the reaction, the reaction mixture was filtered and washed with ethylacetate. The solvent and excess AcOH were removed under reduced pressure on rotary evaporator. The residue obtained was dissolved in EtOAc (100 mL) and washed with 100 mL $NaHCO_3$ aq. The aq. layer was extracted with EtOAc (100 mL × 3). The combined organic layers were dried on anhydrous Na_2SO_4 and under reduced pressure on rotary evaporator to give a pure product **1-IX** (3.6 g, 97%).

Compound **1-IX** (2.8 g, 15 mmol, 1.0 equiv) was treated with di-*tert*-butyl dicarbonate (3.3 g, 15 mmol, 1.0 equiv) in CH_2Cl_2 (50 mL) at reflux for 24 h. The reaction mixture was quenched by the addition of water (50 mL) and extracted with CH_2Cl_2 (50 mL x 2). The combined organic extracts were washed with brine and dried over Na_2SO_4 and concentrated under reduced pressure, the residue was purified by flash chromatography (PE/EA = 10/1) to give the corresponding product **1-X** (3.7 g, 87%).

To a solution of **1-X** (2.8 g, 10 mmol, 1.0 equiv) in CH_2Cl_2 (50 mL) was added Grubbs' II (85 mg, 1.0 mol%), then the mixture was refluxed under Ar for 3 h. After concentrating, the residue was purified by flash chromatography (PE/EA = 10/1) to give the corresponding product **1-XI** (2.5 g, 96%).

To a solution of **1-XI** (2.0 g, 8.0 mmol, 1.0 equiv) in ethanol (30 mL) was added KOH (2.2 g, 40 mmol, 5.0 equiv), then the mixture was refluxed until the complete consumption of **1-XI**. Ethanol was removed under reduced pressure, the residue was dissolved in minimal amount of water, then extracted with CH_2Cl_2 (10 mL x 3) and discarded. The aqueous solution was then cooled to 0 ^oC and was acidified with HCl (1 M) carefully. The acidic aqueous solution was extracted with CH_2Cl_2. The CH_2Cl_2 extracts were dried over Na_2SO_4 and concentrated. The residue was used in the next step without further purification.

To a solution of **1-XII** (1.1 g, 5.0 mmol, 1.0 equiv) and DMAP (0.92 g, 7.5 mmol, 1.5 equiv) in CH_2Cl_2 (20 mL) at 0 ^oC were added aniline (0.55 mL, 6.0 mmol, 1.2 equiv) and EDCI (1.4 g, 7.5 mmol, 1.5 equiv) sequentially, then the mixture was stirred at rt overnight. The reaction mixture was quenched by the addition of water (50 mL) and extracted with CH_2Cl_2 (50 mL x 2). The combined organic extracts were washed with HCl (1 M) and dried over Na_2SO_4 and concentrated under reduced pressure, the residue was purified by flash chromatography (toluene/EA = 9/1) to give the corresponding product **1o** (1.3 g, 83%).

General procedure for the synthesis of 1y

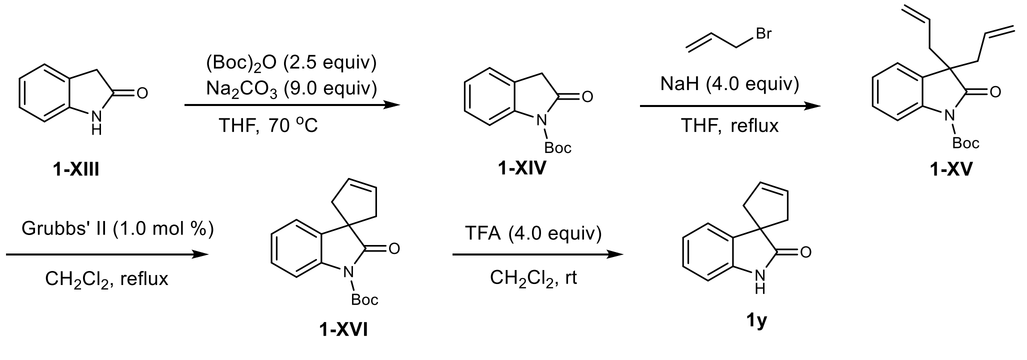

To a solution of indolin-2-one **1-XIII** (4.1 g, 30 mmol, 1.0 equiv) in THF (120 mL) were added Na_2CO_3 (28.6 g, 270 mmol, 9.00 equiv) and $(Boc)_2O$ (16 g, 75 mmol, 2.5 equiv) sequentially, then the suspension was stirred at 70 ^oC overnight. After cooling to rt, the solid was removed by filtration. The solvents were evaporated and the residue was purified by flash chromatography (PE/EA = 10/1) to give **1-XIV** (4.6 g, 65%).

To a suspension of NaH (1.6 g, 40 mmol, 4.0 equiv) in THF (30 mL) at 0 ^oC was added a solution of **1-XIV** (2.3 g, 10 mmol, 1.0 equiv) in THF (10 mL) slowly, then the mixture was stirred at 0 ^oC for 1 h. Allyl bromide (3.5 mL, 40 mmol, 4.0 equiv) was added and the mixture was allowed to reflux for 5 hrs. After cooling to 0 ^oC,

saturated aqueous NH_4Cl solution was added slowly. Layers were separated and the aqueous layer was extracted with CH_2Cl_2, the combined organic extracts were washed with brine and dried over Na_2SO_4. After concentrating, the residue was purified by flash chromatography (PE/EA = 10/1) to give **1-XV** as colorless oil (2.1 g, 67%).

To a solution of **1-XV** (1.6 g, 5.0 mmol, 1.0 equiv) in CH_2Cl_2 (60 mL) was added Grubbs' II (43 mg, 1.0 mol %), then the mixture was refluxed under Ar overnight. After concentrating, the residue was purified by flash chromatography (PE/EA = 10/1) to give **1-XVI** as colorless oil (1.2 g, 87%).

To a solution of **1-XVI** (0.72 g, 2.5 mmol, 1.0 equiv) in CH_2Cl_2 (20 mL) was added trifluoroacetic acid (0.80 mL, 10 mmol, 4.0 equiv), then the mixture was stirred at rt for 1 h. After concentrating, the residue was dissolved in CH_2Cl_2 and the mixture was neutralized at 0 ^{o}C with saturated $NaHCO_3$ solution. Layers were separated and the aqueous layer was extracted with CH_2Cl_2. The combined organic layers were dried over Na_2SO_4 and concentrated. The residue was purified by flash chromatography (PE/EA = 5/1) to give **1y** as colorless solid (0.44 g, 96%).

General procedure for the synthesis of 1ae:

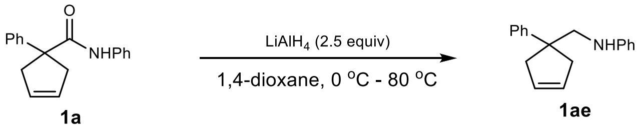

To an oven-dried 25-mL two-necked bottle equipped with a tefloncoated magnetic stir bar was added $LiAlH_4$ (0.19 g, 5.0 mmol, 2.5 equiv). Then, the bottle was evacuated and filled with argon (three cycles). After that, the solution of **1a** (0.53 g, 2.0 mmol, 1.0 equiv) in 1,4-dioxane (10 mL) was added dropwised via a syringe at 0 ^{o}C. The mixture was stirred at 80 ^{o}C for 20 h. The mixture was queched by $Na_2SO_4 \cdot 10H_2O$, and filtered through a short pad of celite washed with CH_2Cl_2. The solvent was concentrated in vacuum and the crude product was purified by flash chromatography on silica gel (PE/EA = 100/1) to afford the desired product (0.47 g, 94%).

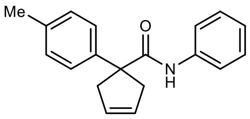

N-phenyl-1-(p-tolyl)cyclopent-3-ene-1-carboxamide (1b)

R_f = 0.6 (PE/EA = 10/1); White solid, m.p. 100 – 102 °C.

^{1}H NMR (300 MHz, $CDCl_3$) δ 7.37 (dd, *J* = 8.5, 1.4 Hz, 2H), 7.28 – 7.21 (m, 4H), 7.17 (d, *J* = 8.0 Hz, 2H), 7.06 – 7.01 (m, 1H), 6.92 (brs, 1H), 5.75 (s, 2H), 3.40 – 3.33 (m, 2H), 2.89 – 2.81 (m, 2H), 2.34 (s, 3H) ppm.

^{13}C NMR (75 MHz, $CDCl_3$) δ 174.8, 141.5, 138.2, 137.0, 129.7, 128.9, 128.8, 126.7, 124.1, 119.6, 58.9, 43.9, 21.0 ppm.

HRMS (ESI) calcd for $[C_{19}H_{19}NO+H]^+$ 278.1539, found 278.1541.

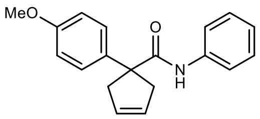

1-(4-methoxyphenyl)-N-phenylcyclopent-3-ene-1-carboxamide (1c)

R_f = 0.5 (PE/EA = 10/1); White solid, m.p. 98 – 100 °C.

^{1}H NMR (300 MHz, $CDCl_3$) δ 7.39 – 7.36 (m, 2H), 7.32 – 7.22 (m, 4H), 7.07 – 7.02 (m, 1H), 6.94 (brs, 1H), 6.93 – 6.87 (m, 2H), 5.76 (s, 2H), 3.80 (s, 3H), 3.40 – 3.32 (m, 2H), 2.88 – 2.81 (m, 2H) ppm.

^{13}C NMR (75 MHz, $CDCl_3$) δ 174.9, 158.7, 138.2, 136.5, 128.9, 128.8, 128.0, 124.1, 119.6, 114.4, 58.6, 55.3, 44.0 ppm.

HRMS (ESI) calcd for $[C_{19}H_{19}NO_2+H]^+$ 294.1489, found 294.1492.

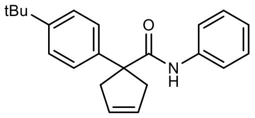

1-(4-(tert-butyl)phenyl)-N-phenylcyclopent-3-ene-1-carboxamide (1d)

R_f = 0.6 (PE/EA = 10/1); White solid, m.p. 149 – 151 °C.

^{1}H NMR (300 MHz, $CDCl_3$) δ 7.41 – 7.37 (m, 4H), 7.33 – 7.23 (m, 4H), 7.07 – 7.02 (m, 1H), 6.94 (brs, 1H), 5.76 (s, 2H), 3.42 – 3.34 (m, 2H), 2.92 – 2.83 (m, 2H), 1.32 (s, 9H) ppm.

^{13}C NMR (75 MHz, $CDCl_3$) δ 174.7, 150.1, 141.4, 138.2, 128.9, 128.8, 126.4, 125.9, 124.1, 119.6, 58.8, 44.0, 34.5, 31.3 ppm.

HRMS (ESI) calcd for $[C_{22}H_{25}NO+H]^+$ 320.2009, found 320.2014.

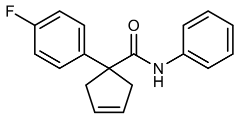

1-(4-fluorophenyl)-N-phenylcyclopent-3-ene-1-carboxamide (1e)

R_f = 0.6 (PE/EA = 10/1); White solid, m.p. 123 – 125 °C.

^{1}H NMR (300 MHz, $CDCl_3$) δ 7.40 – 7.32 (m, 4H), 7.29 – 7.24 (m, 2H), 7.06 (m, 3H), 6.91 (brs, 1H), 5.77 (s, 2H), 3.42 – 3.34 (m, 2H), 2.86 – 2.79 (m, 2H) ppm.

^{13}C NMR (75 MHz, $CDCl_3$) δ 174.2, 161.9 (d, *J* = 246.6 Hz), 140.3 (d, *J* = 3.4 Hz), 138.0, 129.0, 128.8, 128.4, 128.3, 124.3, 119.7, 115.8 (d, *J* = 21.3 Hz), 58.7, 44.0 ppm.

^{19}F NMR (282 MHz, $CDCl_3$) δ -115.29 ppm.
HRMS (ESI) calcd for $[C_{18}H_{16}FNO+H]^+$ 282.1289, found 282.1292.

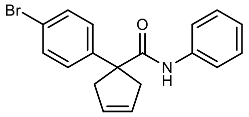

1-(4-bromophenyl)-N-phenylcyclopent-3-ene-1-carboxamide (1f)
R_f = 0.6 (PE/EA = 10/1); White solid, m.p. 142 – 144 °C.
^{1}H NMR (300 MHz, $CDCl_3$) δ 7.50 – 7.45 (m, 2H), 7.40 – 7.35 (m, 2H), 7.29 – 7.22 (m, 4H), 7.09 – 7.04 (m, 1H), 6.95 (brs, 1H), 5.76 (s, 2H), 3.41 – 3.31 (m, 2H), 2.84 – 2.76 (m, 2H) ppm.
^{13}C NMR (75 MHz, $CDCl_3$) δ 173.9, 143.7, 137.9, 132.0, 129.0, 128.8, 128.5, 124.4, 121.3, 119.8, 58.9, 43.8 ppm.
HRMS (ESI) calcd for $[C_{18}H_{16}BrNO+H]^+$ 342.0488, found 342.0485.

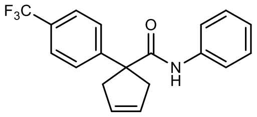

N-phenyl-1-(4-(trifluoromethyl)phenyl)cyclopent-3-ene-1-carboxamide (1g)
R_f = 0.6 (PE/EA = 10/1); White solid, m.p. 121 – 123 °C.
^{1}H NMR (300 MHz, $CDCl_3$) δ 7.61 (d, J = 8.2 Hz, 2H), 7.50 – 7.47 (m, 2H), 7.41 – 7.36 (m, 2H), 7.29 – 7.23 (m, 2H), 7.10– 7.04 (m, 1H), 7.01 (brs, 1H), 5.78 (s, 2H), 3.45 – 3.35 (m, 2H), 2.88 – 2.80 (m, 2H) ppm.
^{13}C NMR (75 MHz, $CDCl_3$) δ 173.5, 148.6, 137.8, 129.4 (d, J = 32.7 Hz), 129.0, 128.8, 127.0, 125.8 (q, J = 3.8 Hz), 124.5, 122.2, 119.9, 59.2, 43.8 ppm.
^{19}F NMR (282 MHz, $CDCl_3$) δ -62.50 ppm.
HRMS (ESI) calcd for $[C_{19}H_{16}F_3NO+H]^+$ 332.1257, found 332.1248.

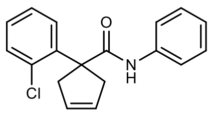

1-(2-chlorophenyl)-N-phenylcyclopent-3-ene-1-carboxamide (1h)
R_f = 0.6 (PE/EA = 10/1); White solid, m.p. 123 – 125 °C.
^{1}H NMR (300 MHz, $CDCl_3$) δ 7.47 – 7.43 (m, 2H), 7.37 – 7.34 (m, 2H), 7.33 – 7.23 (m, 4H), 7.09 – 7.03 (m, 1H), 6.83 (brs, 1H), 5.71 (s, 2H), 3.48 – 3.40 (m, 2H), 2.93 – 2.83 (m, 2H) ppm.
^{13}C NMR (75 MHz, $CDCl_3$) δ 173.7, 140.6, 138.1, 134.9, 131.5, 129.1, 128.9, 128.5, 128.3, 127.1, 124.3, 120.3, 59.0, 43.4 ppm.
HRMS (ESI) calcd for $[C_{18}H_{16}ClNO+H]^+$ 298.0993, found 298.0997.

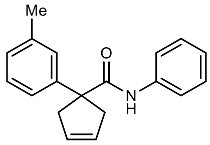

N-phenyl-1-(m-tolyl)cyclopent-3-ene-1-carboxamide (1i)
R_f = 0.6 (PE/EA = 10/1); White solid, m.p. 95 – 97 °C.

^{1}H NMR (300 MHz, $CDCl_3$) δ 7.36 (dd, *J* = 8.3, 1.5 Hz, 2H), 7.27 – 7.16 (m, 5H), 7.10 – 6.98 (m, 3H), 5.74 (s, 2H), 3.40 – 3.33 (m, 2H), 2.90 – 2.82 (m, 2H), 2.34 (s, 3H) ppm.

^{13}C NMR (75 MHz, $CDCl_3$) δ 174.7, 144.5, 138.7, 138.3, 128.9, 128.8, 128.1, 127.6, 124.1, 123.8, 119.7, 59.2, 43.9, 21.7 ppm.

HRMS (ESI) calcd for $[C_{19}H_{19}NO+H]^+$ 278.1539, found 278.1540.

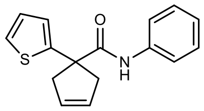

N-phenyl-1-(thiophen-2-yl)cyclopent-3-ene-1-carboxamide (1j)

R_f = 0.6 (PE/EA = 10/1); White solid, m.p. 102 – 104 ^{o}C.

^{1}H NMR (300 MHz, $CDCl_3$) δ 7.41 – 7.38 (m, 2H), 7.29 – 7.23 (m, 4H), 7.10 – 7.00 (m, 3H), 5.74 (s, 2H), 3.49 – 3.38 (m, 2H), 3.02 – 2.95 (m, 2H) ppm.

^{13}C NMR (75 MHz, $CDCl_3$) δ 173.4, 149.3, 137.9, 129.0, 128.5, 127.4, 125.2, 124.9, 124.4, 119.7, 56.6, 46.4 ppm.

HRMS (ESI) calcd for $[C_{16}H_{15}NOS+H]^+$ 270.0947, found 270.0951.

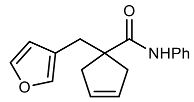

1-(furan-3-ylmethyl)-N-phenylcyclopent-3-ene-1-carboxamide (1k)

R_f = 0.6 (PE/EA = 10/1); White solid, m.p. 110 – 112 ^{o}C.

^{1}H NMR (300 MHz, $CDCl_3$) δ 7.42 (d, *J* = 8.1 Hz, 2H), 7.37 (s, 1H), 7.29 – 7.23 (m, 4H), 7.06 (t, *J* = 7.4 Hz, 1H), 6.23 (s, 1H), 5.69 (s, 2H), 2.87 – 2.79 (m, 4H), 2.52 – 2.44 (m, 2H) ppm.

^{13}C NMR (75 MHz, $CDCl_3$) δ 175.3, 142.7, 140.8, 138.0, 129.1, 129.0, 124.3, 121.0, 120.3, 112.2, 54.0, 42.4, 34.2. ppm.

HRMS (ESI) calcd for $[C_{17}H_{17}NO_2+H]^+$ 268.1332, found 268.1330.

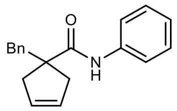

1-benzyl-N-phenylcyclopent-3-ene-1-carboxamide (1l)

R_f = 0.6 (PE/EA = 10/1); White solid, m.p. 134 – 136 ^{o}C.

^{1}H NMR (300 MHz, $CDCl_3$) δ 7.34 – 7.20 (m, 7H), 7.15 (dd, *J* = 7.1, 2.5 Hz, 2H), 7.10 – 7.05 (m, 1H), 6.83 (brs, 1H), 5.74 (s, 2H), 3.03 (s, 2H), 2.82 – 2.75 (m, 2H), 2.61 – 2.51 (m, 2H) ppm.

^{13}C NMR (75 MHz, $CDCl_3$) δ 175.0, 137.8, 137.7, 130.3, 129.1, 128.9, 128.2, 126.8, 124.3, 120.3, 55.3, 44.6, 41.7 ppm.

HRMS (ESI) calcd for $[C_{19}H_{19}NO+H]^+$ 278.1539, found 278.1536.

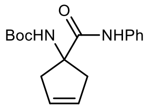

tert-butyl (1-(phenylcarbamoyl)cyclopent-3-en-1-yl)carbamate (1o)

R_f = 0.4 (toluene/EA = 9/1); White solid, m.p. 194 – 196 ^{o}C.

¹H NMR (300 MHz, DMSO-d_6) δ 9.49 (d, 1H), 7.60 (d, *J* = 8.0 Hz, 2H), 7.31 – 7.24 (m, 3H), 7.02 (t, *J* = 7.4 Hz, 1H), 5.62 (s, 2H), 3.01 (d, *J* = 16.8 Hz, 2H), 2.56 (d, *J* = 18.0 Hz, 2H), 1.38 – 1.24 (m, 9H) ppm.
¹³C NMR (75 MHz, DMSO-d_6) δ 173.0, 155.2, 139.8, 128.9, 128.2, 123.5, 120.4, 78.9, 65.9, 44.0, 28.6 ppm.
HRMS (ESI) calcd for $[C_{17}H_{22}N_2O_3+Na]^+$ 325.1523, found 325.1520.

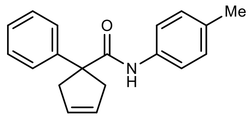

1-phenyl-N-(p-tolyl)cyclopent-3-ene-1-carboxamide (1q)
R_f = 0.6 (PE/EA = 10/1); White solid, m.p. 121 – 123 °C.
¹H NMR (300 MHz, $CDCl_3$) δ 7.40 – 7.31 (m, 4H), 7.30 – 7.23 (m, 3H), 7.04 (d, *J* = 8.2 Hz, 2H), 6.88 (brs, 1H), 5.75 (s, 2H), 3.42 – 3.34 (m, 2H), 2.90 – 2.82 (m, 2H), 2.26 (s, 3H) ppm.
¹³C NMR (75 MHz, $CDCl_3$) δ 174.5, 144.7, 135.6, 133.7, 129.4, 129.0, 128.8, 127.2, 126.8, 119.8, 59.2, 43.9, 20.9 ppm.
HRMS (ESI) calcd for $[C_{19}H_{19}NO+H]^+$ 278.1539, found 278.1543.

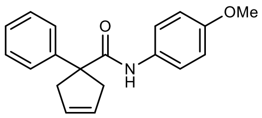

N-(4-methoxyphenyl)-1-phenylcyclopent-3-ene-1-carboxamide (1r)
R_f = 0.5 (PE/EA = 10/1); White solid, m.p. 123 – 125 °C.
¹H NMR (300 MHz, $CDCl_3$) δ 7.40 – 7.34 (m, 4H), 7.31 – 7.25 (m, 3H), 6.82 – 6.76 (m, 3H), 5.77 (s, 2H), 3.75 (s, 3H), 3.42 – 3.35 (m, 2H), 2.91 – 2.83 (m, 2H) ppm.
¹³C NMR (75 MHz, $CDCl_3$) δ 174.5, 156.3, 144.7, 131.3, 129.0, 128.8, 127.2, 126.8, 121.5, 114.0, 59.0, 55.5, 44.0 ppm.
HRMS (ESI) calcd for $[C_{19}H_{19}NO_2+H]^+$ 294.1489, found 294.1489.

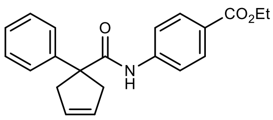

ethyl 4-(1-phenylcyclopent-3-ene-1-carboxamido)benzoate (1s)
R_f = 0.5 (PE/EA = 10/1); White solid, m.p. 155 – 157 °C.
¹H NMR (300 MHz, $CDCl_3$) δ 7.95 – 7.91 (m, 2H), 7.48 – 7.43 (m, 2H), 7.38 (d, *J* = 4.1 Hz, 4H), 7.33 – 7.28 (m, 1H), 7.13 (brs, 1H), 5.78 (s, 2H), 4.32 (q, *J* = 7.1 Hz, 2H), 3.43 – 3.35 (m, 2H), 2.94 – 2.85 (m, 2H), 1.36 (t, *J* = 7.1 Hz, 3H) ppm.
¹³C NMR (75 MHz, $CDCl_3$) δ 174.7, 166.2, 144.1, 142.2, 130.7, 129.2, 128.8, 127.5, 126.7, 125.8, 118.6, 60.9, 59.4, 43.8, 14.4 ppm.
HRMS (ESI) calcd for $[C_{21}H_{21}NO_3+H]^+$ 336.1594, found 336.1596.

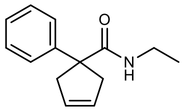

***N*-ethyl-1-phenylcyclopent-3-ene-1-carboxamide (1t)**

R_f = 0.6 (PE/EA = 10/1); White solid, m.p. 67 – 68 ^{o}C.

^{1}H NMR (300 MHz, $CDCl_3$) δ 7.32 (d, *J* = 3.2 Hz, 4H), 7.27 – 7.21 (m, 1H), 5.72 (s, 2H), 5.25 (brs, 1H), 3.33 – 3.17 (d, 4H), 2.81 – 2.73 (d, 2H), 1.01 (t, *J* = 7.2 Hz, 3H) ppm.

^{13}C NMR (75 MHz, $CDCl_3$) δ 176.4, 145.3, 128.7, 128.7, 126.8, 126.7, 58.2, 44.0, 34.8, 14.7 ppm

HRMS (ESI) calcd for $[C_{14}H_{17}NO+H]^+$ 216.1383, found 216.1388.

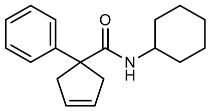

***N*-cyclohexyl-1-phenylcyclopent-3-ene-1-carboxamide (1v)**

R_f = 0.6 (PE/EA = 10/1); White solid, m.p. 99 – 101 ^{o}C.

^{1}H NMR (300 MHz, $CDCl_3$) δ 7.36 – 7.29 (m, 4H), 7.24 (ddd, *J* = 5.7, 3.6, 2.6 Hz, 1H), 5.72 (s, 2H), 5.02 (d, 1H), 3.78 – 3.68 (m, 1H), 3.31 – 3.22 (m, 2H), 2.80 – 2.71 (m, 2H), 1.78 (dt, *J* = 12.3, 4.1 Hz, 2H), 1.62 – 1.51 (m, 3H), 1.37 – 1.25 (m, 2H), 1.16 – 0.90 (m, 3H) ppm

^{13}C NMR (75 MHz, $CDCl_3$) δ 175.6, 145.5, 128.7, 128.6, 126.8, 126.6, 58.2, 48.4, 44.1, 32.7, 25.5, 24.7 ppm.

HRMS (ESI) calcd for $[C_{18}H_{23}NO+H]^+$ 270.1852, found 270.1855.

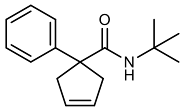

***N*-(tert-butyl)-1-phenylcyclopent-3-ene-1-carboxamide (1w)**

R_f = 0.6 (PE/EA = 10/1); White solid, m.p. 88 – 90 ^{o}C.

^{1}H NMR (300 MHz, $CDCl_3$) δ 7.36 – 7.27 (m, 4H), 7.26 – 7.21 (m, 1H), 5.71 (s, 2H), 4.97 (brs, 1H), 3.28 – 3.22 (m, 2H), 2.77 – 2.70 (m, 2H), 1.24 (s, 9H) ppm.

^{13}C NMR (75 MHz, $CDCl_3$) δ 175.9, 145.8, 128.7, 128.6, 126.7, 126.5, 58.7, 51.1, 44.0, 28.6 ppm.

HRMS (ESI) calcd for $[C_{16}H_{21}NO+H]^+$ 244.1696, found 244.1701.

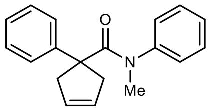

***N*-methyl-*N*,1-diphenylcyclopent-3-ene-1-carboxamide (1x)**

R_f = 0.6 (PE/EA = 10/1); White solid, m.p. 59 – 61 ^{o}C.

^{1}H NMR (300 MHz, $CDCl_3$) δ 7.20 (d, 3H), 7.14 – 7.04 (m, 5H), 6.63 (s, 2H), 5.53 (s, 2H), 3.21 (d, 5H), 2.33 (d, 2H) ppm.

^{13}C NMR (75 MHz, $CDCl_3$) δ 175.3, 146.1, 143.6, 128.8 – 127.5 (m), 127.2, 126.3, 125.2, 58.7, 44.8, 40.1 ppm.

HRMS (ESI) calcd for $[C_{19}H_{19}NO+H]^+$ 278.1539, found 278.1545.

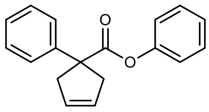

phenyl 1-phenylcyclopent-3-ene-1-carboxylate (1z)

R_f = 0.5 (PE/EA = 50/1); Colorless oil.

^{1}H NMR (300 MHz, $CDCl_3$) δ 7.45 – 7.41 (m, 2H), 7.38 – 7.23 (m, 5H), 7.17 – 7.12 (m, 1H), 6.92 – 6.88 (m, 2H), 5.82 (s, 2H), 3.59 – 3.50 (m, 2H), 2.91 – 2.83 (m, 2H) ppm.

^{13}C NMR (75 MHz, $CDCl_3$) δ 174.8, 151.2, 143.2, 129.4, 129.3, 128.7, 127.1, 126.7, 125.8, 121.3, 58.8, 42.8 ppm.

HRMS (ESI) calcd for $[C_{18}H_{16}O_2+H]^+$ 265.1223, found 265.1218.

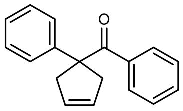

phenyl(1-phenylcyclopent-3-en-1-yl)methanone (1ab)

R_f = 0.6 (PE/EA = 20/1); White solid, m.p. 101 – 103 °C.

^{1}H NMR (300 MHz, $CDCl_3$) δ 7.65 – 7.61 (m, 2H), 7.39 – 7.31 (m, 1H), 7.30 – 7.17 (m, 7H), 5.68 (s, 2H), 3.43 – 3.32 (m, 2H), 2.89 – 2.79 (m, 2H) ppm.

^{13}C NMR (75 MHz, $CDCl_3$) δ 201.3, 145.9, 135.9, 132.0, 130.2, 129.0, 128.1, 128.1, 126.6, 125.5, 62.2, 44.4 ppm.

HRMS (ESI) calcd for $[C_{18}H_{16}O+H]^+$ 249.1274, found 249.1280.

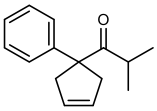

2-methyl-1-(1-phenylcyclopent-3-en-1-yl)propan-1-one (1ac)

R_f = 0.6 (PE/EA = 20/1); Colorless oil.

^{1}H NMR (300 MHz, $CDCl_3$) δ 7.36 – 7.21 (m, 5H), 5.72 (s, 2H), 3.35 – 3.26 (m, 2H), 2.90 (p, J = 6.7 Hz, 1H), 2.77 – 2.70 (m, 2H), 0.81 (s, 3H), 0.79 (s, 3H) ppm.

^{13}C NMR (75 MHz, $CDCl_3$) δ 214.4, 142.1, 128.9, 128.6, 127.5, 126.9, 65.0, 40.0, 35.7, 20.9 ppm.

HRMS (ESI) calcd for $[C_{15}H_{18}O+H]^+$ 215.1430, found 215.1428.

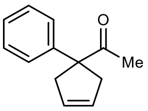

1-(1-phenylcyclopent-3-en-1-yl)ethan-1-one (1ad)

R_f = 0.6 (PE/EA = 20/1); Colorless oil.

^{1}H NMR (300 MHz, $CDCl_3$) δ 7.36 – 7.30 (m, 2H), 7.26 – 7.19 (m, 3H), 5.70 (s, 2H), 3.28 – 3.20 (m, 2H), 2.77 – 2.67 (m, 2H), 1.98 (s, 3H) ppm.

^{13}C NMR (75 MHz, $CDCl_3$) δ 208.4, 143.5, 129.0, 128.9, 127.1, 127.0, 64.8, 41.1, 26.0 ppm.

HRMS (ESI) calcd for $[C_{13}H_{14}O+H]^+$ 187.1117, found 187.1117.

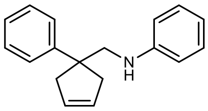

N-((1-phenylcyclopent-3-en-1-yl)methyl)aniline (1ae)

R_f = 0.6 (PE/EA = 50/1); Pale yellow oil.

^{1}H NMR (300 MHz, $CDCl_3$) δ 7.37 – 7.28 (m, 4H), 7.25 – 7.20 (m, 1H), 7.13 – 7.05 (m, 2H), 6.63 (t, *J* = 7.3 Hz, 1H), 6.48 (d, *J* = 8.0 Hz, 2H), 5.80 (s, 2H), 3.49 (s, 1H), 3.26 (s, 2H), 2.75 (s, 4H) ppm.

^{13}C NMR (75 MHz, $CDCl_3$) δ 148.9, 147.5, 129.7, 129.2, 128.6, 127.0, 126.3, 117.1, 112.6, 54.4, 51.0, 43.2 ppm.

HRMS (ESI) calcd for $[C_{18}H_{19}N+H]^+$ 250.1590, found 250.1587.

3. Optimization of Reaction Conditions

Supplementary Table 1. Details of Optimization of Reaction Conditions[a]

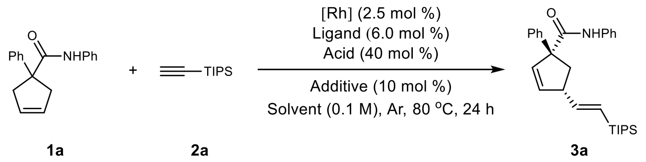

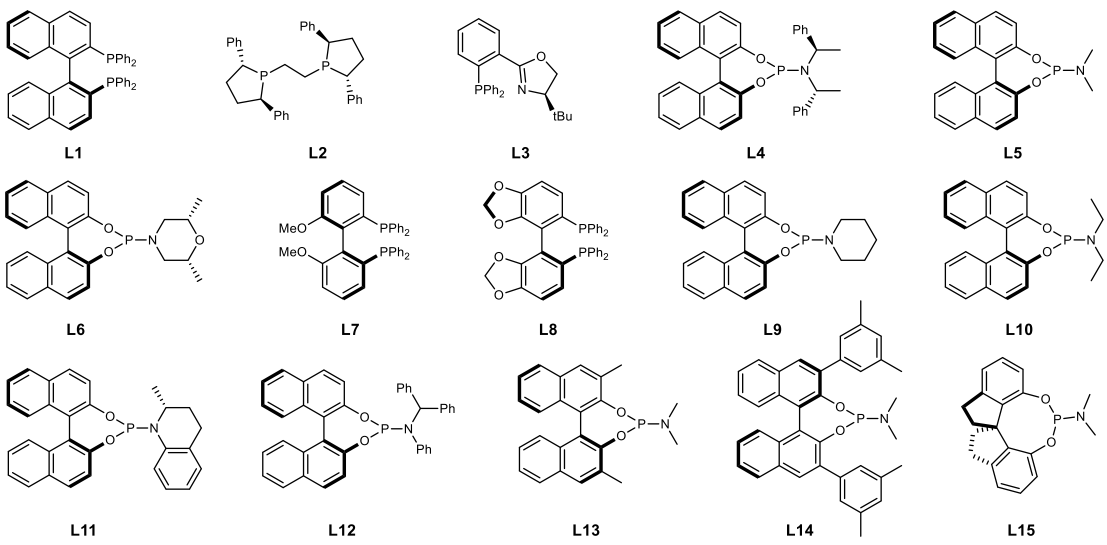

entry	[Rh]	ligand	acid	solvent	additive	yield (%)	ee (%)
1	$[Rh(COD)OMe]_2$	**L1**	Ph_2CHCO_2H	DCM	NaBARF	trace	--
2	$[Rh(COD)OMe]_2$	**L2**	Ph_2CHCO_2H	DCM	NaBARF	trace	--
3	$[Rh(COD)OMe]_2$	**L3**	Ph_2CHCO_2H	DCM	NaBARF	trace	--
4	$[Rh(COD)OMe]_2$	**L4**	Ph_2CHCO_2H	DCM	NaBARF	11	44
5	$[Rh(COD)OMe]_2$	**L5**	Ph_2CHCO_2H	DCM	NaBARF	80	78
6	$[Rh(COD)OMe]_2$	**L6**	Ph_2CHCO_2H	DCM	NaBARF	94	92
7	$[Rh(COD)OMe]_2$	**L7**	Ph_2CHCO_2H	DCM	NaBARF	trace	--
8	$[Rh(COD)OMe]_2$	**L8**	Ph_2CHCO_2H	DCM	NaBARF	trace	--
9	$[Rh(COD)OMe]_2$	**L9**	Ph_2CHCO_2H	DCM	NaBARF	97	85
10	$[Rh(COD)OMe]_2$	**L10**	Ph_2CHCO_2H	DCM	NaBARF	93	87
11	$[Rh(COD)OMe]_2$	**L11**	Ph_2CHCO_2H	DCM	NaBARF	27	39
12	$[Rh(COD)OMe]_2$	**L12**	Ph_2CHCO_2H	DCM	NaBARF	trace	--
13	$[Rh(COD)OMe]_2$	**L13**	Ph_2CHCO_2H	DCM	NaBARF	95	64
14	$[Rh(COD)OMe]_2$	**L14**	Ph_2CHCO_2H	DCM	NaBARF	92	-14
15	$[Rh(COD)OMe]_2$	**L15**	Ph_2CHCO_2H	DCM	NaBARF	34	74
16	$Rh(COD)_2OTf$	**L6**	Ph_2CHCO_2H	DCM	NaBARF	91	81
17	$Rh(COD)_2BF_4$	**L6**	Ph_2CHCO_2H	DCM	NaBARF	87	80
18	$[Rh(COD)Cl]_2$	**L6**	Ph_2CHCO_2H	DCM	NaBARF	91	80
19	$[Rh(COE)_2Cl]_2$	**L6**	Ph_2CHCO_2H	DCM	NaBARF	83	80
20	$[Rh(COD)OH]_2$	**L6**	Ph_2CHCO_2H	DCM	NaBARF	74	83
21	$Rh(PPh_3)_3Cl$	**L6**	Ph_2CHCO_2H	DCM	NaBARF	81	6

22	$[Rh(COD)OMe]_2$	**L6**	Ph_2CHCO_2H	DCE	NaBARF	94	86
23	$[Rh(COD)OMe]_2$	**L6**	Ph_2CHCO_2H	$CHCl_3$	NaBARF	92	86
24	$[Rh(COD)OMe]_2$	**L6**	Ph_2CHCO_2H	THF	NaBARF	n.d.	--
25	$[Rh(COD)OMe]_2$	**L6**	Ph_2CHCO_2H	dioxane	NaBARF	n.d.	--
26	$[Rh(COD)OMe]_2$	**L6**	Ph_2CHCO_2H	DME	NaBARF	trace	--
27	$[Rh(COD)OMe]_2$	**L6**	Ph_2CHCO_2H	toluene	NaBARF	90	83
28	$[Rh(COD)OMe]_2$	**L6**	Ph_2CHCO_2H	$PhCF_3$	NaBARF	92	87
29	$[Rh(COD)OMe]_2$	**L6**	Ph_2CHCO_2H	PhCl	NaBARF	93	86
30.	$[Rh(COD)OMe]_2$	**L6**	Ph_2CHCO_2H	Mesitylene	NaBARF	88	88
31	$[Rh(COD)OMe]_2$	**L6**	Ph_2CHCO_2H	DMF	NaBARF	n.d.	--
32	$[Rh(COD)OMe]_2$	**L6**	Ph_2CHCO_2H	CH_3OH	NaBARF	trace	--
33	$[Rh(COD)OMe]_2$	**L6**	Ph_2CHCO_2H	EA	NaBARF	trace	--
34	$[Rh(COD)OMe]_2$	**L6**	Ph_2CHCO_2H	acetone	NaBARF	trace	--
35	$[Rh(COD)OMe]_2$	**L6**	Ph_2CHCO_2H	MeCN	NaBARF	trace	--
36	$[Rh(COD)OMe]_2$	**L6**	m-xylylic acid	DCM	NaBARF	66	76
37	$[Rh(COD)OMe]_2$	**L6**	$(PhO)_2P(O)OH$	DCM	NaBARF	trace	--
38	$[Rh(COD)OMe]_2$	**L6**	$MesCO_2H$	DCM	NaBARF	53	54
39	$[Rh(COD)OMe]_2$	**L6**	Ph_3CCO_2H	DCM	NaBARF	24	82
40	$[Rh(COD)OMe]_2$	**L6**	PivOH	DCM	NaBARF	29	82
41	$[Rh(COD)OMe]_2$	**L6**	TsOH	DCM	NaBARF	n.d.	--
42	$[Rh(COD)OMe]_2$	**L6**	$PhCO_2H$	DCM	NaBARF	84	67
43	$[Rh(COD)OMe]_2$	**L6**	$1\text{-}AdCO_2H$	DCM	NaBARF	35	92
44	$[Rh(COD)OMe]_2$	**L6**	$PhCH_2CO_2H$	DCM	NaBARF	57	69
45	$[Rh(COD)OMe]_2$	**L6**	$MesCH_2CO_2H$	DCM	NaBARF	37	81
46	$[Rh(COD)OMe]_2$	**L6**	$PhMe_2CCO_2H$	DCM	NaBARF	72	92
47[b]	$[Rh(COD)OMe]_2$	**L6**	$PhMe_2CCO_2H$	DCM	NaBARF	56	94
**48**[c]	**$[Rh(COD)OMe]_2$**	**L6**	**$PhMe_2CCO_2H$**	**DCM**	**NaBARF**	**96**	**95**
49[d]	$[Rh(COD)OMe]_2$	**L6**	$PhMe_2CCO_2H$	DCM	NaBARF	26	94
50[ce]	$[Rh(COD)OMe]_2$	**L6**	$PhMe_2CCO_2H$	DCM	NaBARF	72	94
51	$[Rh(COD)OMe]_2$	**L6**	$PhMe_2CCO_2H$	DCM	$AgSbF_6$	87	92
52	$[Rh(COD)OMe]_2$	**L6**	$PhMe_2CCO_2H$	DCM	$AgPF_6$	40	60
53	$[Rh(COD)OMe]_2$	**L6**	$PhMe_2CCO_2H$	DCM	AgOAc	trace	--
54	$[Rh(COD)OMe]_2$	**L6**	$PhMe_2CCO_2H$	DCM	$AgBF_4$	38	73
55	$[Rh(COD)OMe]_2$	**L6**	$PhMe_2CCO_2H$	DCM	AgOTs	27	2
56[c]	$[Rh(COD)OMe]_2$	**L6**	$PhMe_2CCO_2H$	DCM	--	10	0
57[cf]	$[Rh(COD)OMe]_2$	**L6**	$PhMe_2CCO_2H$	DCM	NaBARF	57	96
58[cg]	$[Rh(COD)OMe]_2$	**L6**	$PhMe_2CCO_2H$	DCM	NaBARF	10	98
59[c]	$[Rh(COD)OMe]_2$	**L6**	$PhMe_2CCO_2H$	DCE	NaBARF	86	92
60[c]	$[Rh(COD)OMe]_2$	**L6**	$PhMe_2CCO_2H$	CCl_4	NaBARF	trace	--
61[c]	$[Rh(COD)OMe]_2$	**L6**	$PhMe_2CCO_2H$	$CHCl_3$	NaBARF	85	89
62[c]	$[Rh(COD)OMe]_2$	**L6**	$PhMe_2CCO_2H$	toluene	NaBARF	85	91
63[c]	$[Rh(COD)OMe]_2$	**L6**	$PhMe_2CCO_2H$	PhCl	NaBARF	86	92
64[ch]	$[Rh(COD)OMe]_2$	**L6**	$PhMe_2CCO_2H$	DCM	NaBARF	86	94
65[ci]	$[Rh(COD)OMe]_2$	**L6**	$PhMe_2CCO_2H$	DCM	NaBARF	81	95

66[cj]	$[Rh(COD)OMe]_2$	**L6**	$PhMe_2CCO_2H$	DCM	NaBARF	47	94

[a]Reaction conditions: **1a** (0.10 mmol), **2a** (0.30 mmol), [Rh] (2.5 mol%), ligand (6.0 mol%), acid (40 mol%), additive (10 mol%), solvent (1.0 mL), 80 ^{o}C, 24 h, under argon. Yields of isolated products are given, d.r. > 20:1, E/Z > 20:1, determined by ^{1}H NMR analysis. The ee value was determined by chiral-phase HPLC. [b]$PhMe_2CCO_2H$ (40 mol%), 36 h. [c]$PhMe_2CCO_2H$ (60 mol%), 36 h. [d]$PhMe_2CCO_2H$ (20 mol%), 36 h. [e]5.0 mol% NaBARF. [f]60 ^{o}C. [g]rt. [h]2.0 eq **2a**. [ih]1.5 eq **2a**. [j]1.0 eq **2a**.

4. General Procedure for the rhodium-catalyzed intermolecular enantioselective Alder–ene type reaction of cyclopentenes with silylacetylenes.

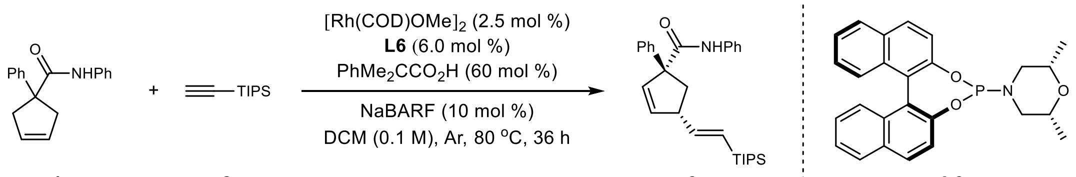

To an oven-dried 10-mL schlenk tube equipped with a tefloncoated magnetic stir bar was added **1a** (26.3 mg, 0.100 mmol, 1.00 equiv), $[Rh(COD)OMe]_2$ (1.2 mg, 2.5 mol%), **L6** (2.7 mg, 6.0 mol%), NaBARF (8.9 mg, 10 mol%) and $PhMe_2CCO_2H$ (10 mg, 60 mol%). The vial was thoroughly flushed with Ar, and **2a** (54.7 mg, 0.600 mmol, 3.00 equiv), as well as DCM (1.0 mL) was added under Ar atmosphere. Then the reaction mixture was stirred at 80 ^{o}C for 36 h. After the reaction vessel was cooled to room temperature, the solution was concentrated in vacuum and purified by careful chromatography on silica gel (PE/EA = 50/1) to afford the desired product **3a**.

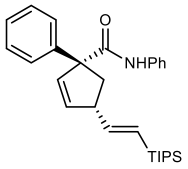

(1R,4S)-N,1-diphenyl-4-((E)-2-(triisopropylsilyl)vinyl)cyclopent-2-ene-1-carboxamide (3a)

42.8 mg, 96% yield, 95% *ee*, d.r. > 20:1, E/Z > 20:1; R_f = 0.7 (PE/EA = 10/1); Colorless oil. $[\alpha]_D^{20}$ = -86 (*c* = 0.13, MeOH).

^{1}H NMR (300 MHz, $CDCl_3$) δ 7.41 – 7.31 (m, 6H), 7.29 – 7.20 (m, 3H), 7.08 – 7.01 (m, 2H), 6.14 (dd, *J* = 5.5, 2.3 Hz, 1H), 6.08 (dd, *J* = 18.8, 7.5 Hz, 2H), 5.98 (dd, *J* = 5.6, 2.1 Hz, 1H), 5.58 (dd, *J* = 18.8, 1.1 Hz, 1H), 3.45 (q, *J* = 9.0, 6.7, 2.3, 1.2 Hz, 1H), 2.81 (dd, *J* = 13.2, 6.9 Hz, 1H), 2.48 (dd, *J* = 13.2, 7.8 Hz, 1H), 1.09 – 0.98 (m, 21H) ppm.

^{13}C NMR (75 MHz, $CDCl_3$) δ 173.1, 150.3, 143.3, 138.1, 138.1, 132.9, 129.0, 128.9, 127.4, 126.7, 124.3, 124.1, 119.8, 66.1, 52.4, 42.8, 18.7, 10.9 ppm.

HRMS (ESI) calcd for $[C_{29}H_{39}NOSi+H]^+$ 446.2874, found 446.2881.

HPLC: Daicel Chiralcel OD-H, *n*-hexane/isopropanol 95/5, flow rate = 1.0 mL/min, uv-vis λ = 250 nm, t_{R1} = 4.8 min (major), t_{R2} = 5.7 min (minor).

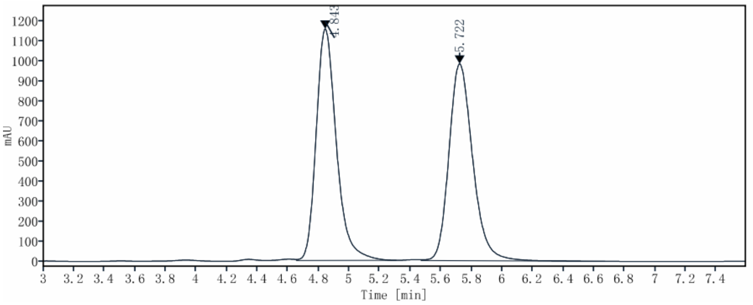

RetTime[min]	Type	Width[min]	Area[mAU*s]	Height[mAU]	Area%
4.843	VB	0.6527	10648.0881	1159.9641	50.0019
5.722	VB	1.2013	10647.2896	985.9181	49.9981

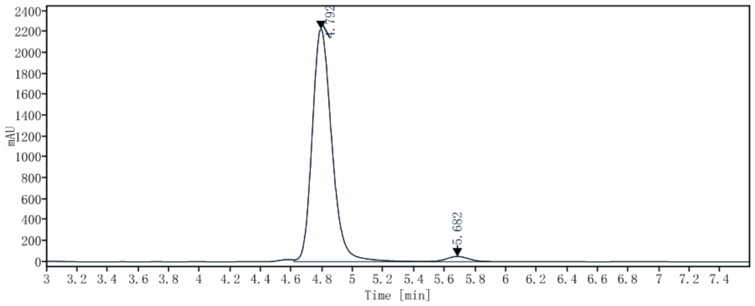

RetTime[min]	Type	Width[min]	Area[mAU*s]	Height[mAU]	Area%
4.792	VM m	0.1380	19856.5459	2228.2349	97.4883
5.682	MM m	0.1593	511.5813	49.6002	2.5117

Supplementary Figure 2. HPLC Trace of **3a**

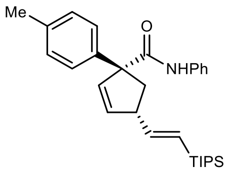

(1R,4S)-N-phenyl-1-(p-tolyl)-4-((E)-2-(triisopropylsilyl)vinyl)cyclopent-2-ene-1-carboxamide (3b)

41.4 mg, 90% yield, 91% *ee*, d.r. > 20:1, E/Z > 20:1; R_f = 0.7 (PE/EA = 10/1); Colorless oil. $[\alpha]_D^{20}$ = -81 (*c* = 0.11, MeOH).

^{1}H NMR (300 MHz, $CDCl_3$) δ 7.40 (d, *J* = 7.9 Hz, 2H), 7.25 (td, *J* = 8.1, 2.4 Hz, 4H), 7.16 (d, *J* = 8.0 Hz, 2H), 7.07 – 7.02 (m, 2H), 6.14 – 6.12 (dd, 1H), 6.11 – 6.03 (dd, 1H), 5.96 (dd, *J* = 5.5, 2.0 Hz, 1H), 5.58 (dd, *J* = 18.8, 1.1 Hz, 1H), 3.44 (q, *J* = 7.7 Hz, 1H), 2.78 (dd, *J* = 13.1, 7.0 Hz, 1H), 2.47 (dd, *J* = 13.1, 7.7 Hz, 1H), 2.33 (s, 3H), 1.11 – 0.93 (m, 21H) ppm.

^{13}C NMR (75 MHz, $CDCl_3$) δ 173.3, 150.3, 140.2, 138.1, 137.8, 137.1, 133.1, 129.6, 128.9, 126.6, 124.2, 124.0, 119.7, 65.7, 52.4, 42.8, 21.0, 18.7, 10.8 ppm.

HRMS (ESI) calcd for $[C_{30}H_{41}NOSi+H]^+$ 460.3030, found 460.3037.

HPLC: Daicel Chiralcel IC-3, *n*-hexane/isopropanol 98/2, flow rate = 0.5 mL/min, uv-vis λ = 250 nm, t_{R1} = 15.7 min (minor), t_{R2} = 19.6 min (major).

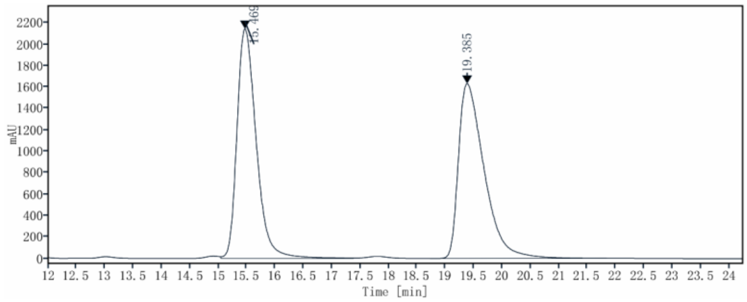

RetTime[min]	Type	Width[min]	Area[mAU*s]	Height[mAU]	Area%
15.469	VV	2.3352	50767.3850	2144.0652	49.7543
19.385	BB	3.1088	51268.7692	1630.9838	50.2457

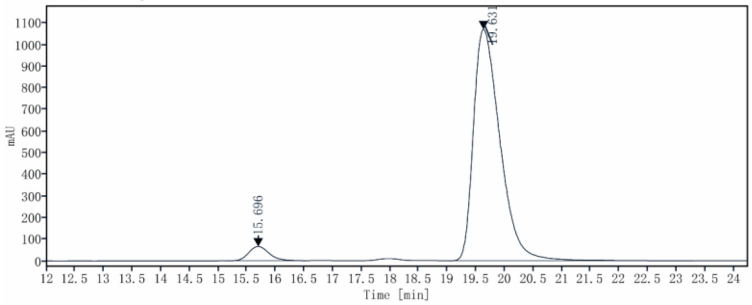

RetTime[min]	Type	Width[min]	Area[mAU*s]	Height[mAU]	Area%
15.696	MB m	0.3859	1614.0352	65.4050	4.4701
19.631	BB	4.2667	34493.4260	1068.5168	95.5299

Supplementary Figure 3. HPLC Trace of **3b**

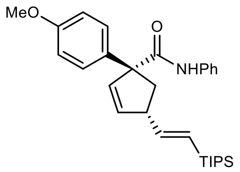

(1R,4S)-1-(4-methoxyphenyl)-N-phenyl-4-((E)-2-(triisopropylsilyl)vinyl)cyclopent-2-ene-1-carboxamide (3c)

45.7 mg, 96% yield, 94% *ee*, d.r. > 20:1, E/Z > 20:1; R_f = 0.5 (PE/EA = 10/1); Colorless oil. $[\alpha]_D^{20}$ = -68 (*c* = 0.15, MeOH).

^{1}H NMR (300 MHz, $CDCl_3$) δ 7.47 – 7.43 (m, 2H), 7.37 – 7.26 (m, 4H), 7.12 – 7.06 (m, 2H), 6.95 – 6.90 (m, 2H), 6.16 (dd, *J* = 5.5, 2.4 Hz, 1H), 6.15 – 6.07 (dd, 1H), 6.01 (dd, *J* = 5.5, 2.0 Hz, 1H), 5.62 (dd, *J* = 18.8, 1.1 Hz, 1H), 3.83 (s, 3H), 3.55 – 3.41 (m, 1H), 2.82 (dd, *J* = 13.1, 7.1 Hz, 1H), 2.50 (dd, *J* = 13.1, 7.7 Hz, 1H), 1.13 – 1.00 (m, 21H) ppm.

^{13}C NMR (75 MHz, $CDCl_3$) δ 173.4, 158.8, 150.3, 138.1, 137.8, 135.2, 133.1, 128.9, 127.9, 124.2, 124.0, 119.7, 114.3, 65.4, 55.3, 52.4, 42.9, 18.7, 10.8 ppm.

HRMS (ESI) calcd for $[C_{30}H_{41}NO_2Si+H]^+$ 476.2979, found 476.2984.

HPLC: Daicel Chiralcel IC-3, *n*-hexane/isopropanol 98/2, flow rate = 0.5 mL/min, uv-vis λ = 250 nm, t_{R1} = 28.8 min (minor), t_{R2} = 33.3 min (major).

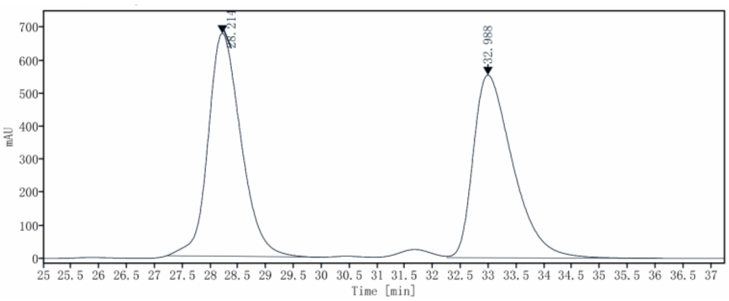

RetTime[min]	Type	Width[min]	Area[mAU*s]	Height[mAU]	Area%
28.214	MB m	0.6299	27729.0129	675.5001	50.3664
32.988	VB	3.7742	27325.5932	553.8832	49.6336

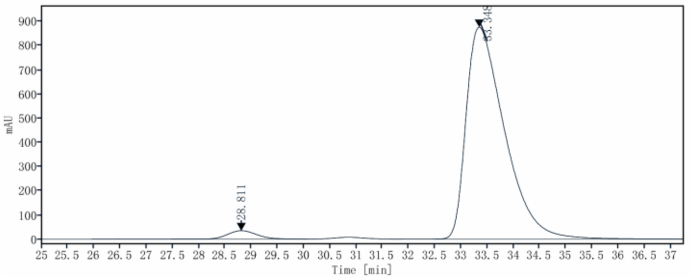

RetTime[min]	Type	Width[min]	Area[mAU*s]	Height[mAU]	Area%
28.811	BB	2.5894	1362.3803	35.0909	2.9698
33.348	BB	5.8900	44512.7232	875.6461	97.0302

Supplementary Figure 4. HPLC Trace of **3c**

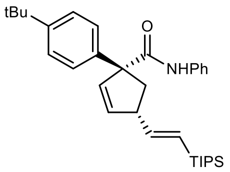

(1R,4S)-1-(4-(tert-butyl)phenyl)-N-phenyl-4-((E)-2-(triisopropylsilyl)vinyl)cyclopent-2-ene-1-carboxamide (3d)

47.2 mg, 94% yield, 93% *ee*, d.r. > 20:1, E/Z > 20:1; R_f = 0.8 (PE/EA = 10/1); White solid, m.p. 108 – 110 °C; $[\alpha]_D^{20}$ = -75 (*c* = 0.13, MeOH).

¹H NMR (300 MHz, $CDCl_3$) δ 7.43 – 7.35 (m, 4H), 7.31 – 7.22 (m, 4H), 7.07 – 7.01 (m, 2H), 6.14 (dd, *J* = 5.5, 2.3 Hz, 1H), 6.07 (dd, *J* = 18.8, 7.5 Hz, 1H), 5.96 (dd, *J* = 5.5, 2.0 Hz, 1H), 5.58 (dd, *J* = 18.8, 1.1 Hz, 1H), 3.45 (q, *J* = 7.4 Hz, 1H), 2.78 (dd, *J* = 13.1, 6.9 Hz, 1H), 2.49 (dd, *J* = 13.1, 7.7 Hz, 1H), 1.31 (s, 9H), 1.10 – 0.95 (m, 21H) ppm.

¹³C NMR (75 MHz, $CDCl_3$) δ 173.3, 150.4, 150.2, 140.1, 138.1, 137.8, 133.1, 128.9, 126.3, 125.9, 124.2, 124.0, 119.7, 65.7, 52.4, 42.8, 34.5, 31.4, 18.7, 10.8 ppm.

HRMS (ESI) calcd for $[C_{33}H_{47}NOSi+H]^+$ 502.3500, found 502.3506.

HPLC: Daicel Chiralcel IC-3, *n*-hexane/isopropanol 98/2, flow rate = 0.5 mL/min, uv-vis λ = 250 nm, t_{R1} = 13.1 min (minor), t_{R2} = 17.0 min (major).

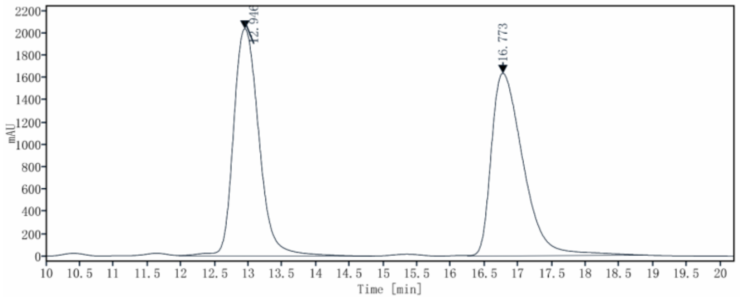

RetTime[min]	Type	Width[min]	Area[mAU*s]	Height[mAU]	Area%
12.946	VB	2.8605	53503.3271	2038.2066	49.8642
16.773	VM m	0.5102	53794.7350	1637.8208	50.1358

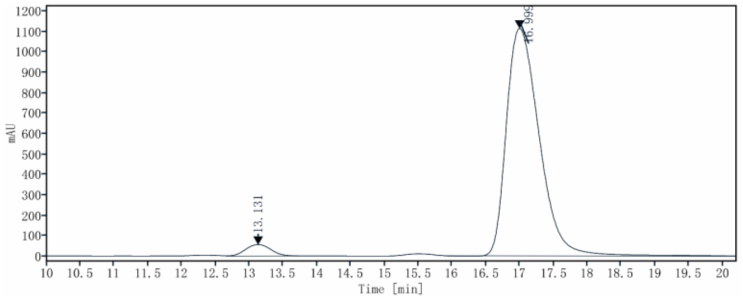

RetTime[min]	Type	Width[min]	Area[mAU*s]	Height[mAU]	Area%
13.131	VM m	0.4182	1443.3072	55.5389	3.6887
16.999	MB m	0.5319	37684.6476	1113.4334	96.3113

Supplementary Figure 5. HPLC Trace of **3d**

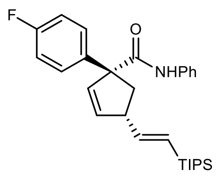

(1R,4S)-1-(4-fluorophenyl)-N-phenyl-4-((E)-2-(triisopropylsilyl)vinyl)cyclopent-2-ene-1-carboxamide (3e)

44.5 mg, 96% yield, 94% *ee*, d.r. > 20:1, E/Z > 20:1; R_f = 0.7 (PE/EA = 10/1); Colorless oil. $[\alpha]_D^{20}$ = -56 (*c* = 0.16, MeOH).

^{1}H NMR (300 MHz, $CDCl_3$) δ 7.45 – 7.40 (m, 2H), 7.39 – 7.31 (m, 2H), 7.31 – 7.25 (m, 3H), 7.11 – 7.00 (m, 4H), 6.13 (dd, *J* = 5.6, 2.3 Hz, 1H), 6.10 – 6.04 (dd, 1H), 6.03 – 6.01 (dd, 1H), 5.59 (dd, *J* = 18.8, 1.1 Hz, 1H), 3.46 (m, 1H), 2.79 (dd, *J* = 13.2, 6.7 Hz, 1H), 2.46 (dd, *J* = 13.2, 7.8 Hz, 1H), 1.10 – 0.95 (m, 21H) ppm.

^{13}C NMR (75 MHz, $CDCl_3$) δ 172.8, 162.0 (d, *J* = 246.5 Hz), 149.9, 139.0 (d, *J* = 3.3 Hz), 138.6, 137.9, 132.5, 128.9, 128.4, 128.3, 124.4, 124.3, 119.8, 115.7 (d, *J* = 21.3 Hz), 65.4, 52.2, 43.2, 18.7, 10.8 ppm.

^{19}F NMR (282 MHz, $CDCl_3$) δ -115.21 ppm.

HRMS (ESI) calcd for $[C_{29}H_{38}FNOSi+H]^+$ 464.2779, found 464.2781.

HPLC: Daicel Chiralcel IC-3, *n*-hexane/isopropanol 98/2, flow rate = 0.5 mL/min, uv-vis λ = 250 nm, t_{R1} = 13.3 min (minor), t_{R2} = 16.1 min (major).

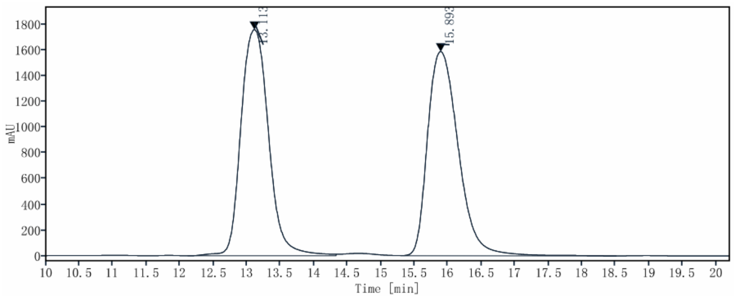

RetTime[min]	Type	Width[min]	Area[mAU*s]	Height[mAU]	Area%
13.113	BV	2.2140	50435.6888	1757.4664	49.9299
15.893	VB	3.2204	50577.2717	1587.4992	50.0701

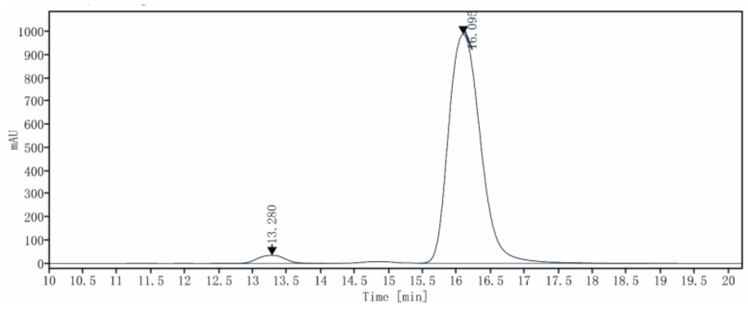

RetTime[min]	Type	Width[min]	Area[mAU*s]	Height[mAU]	Area%
13.280	BB	1.8030	1001.1584	35.0416	3.0048
16.095	VB	3.3760	32317.1540	987.6861	96.9952

Supplementary Figure 6. HPLC Trace of **3e**

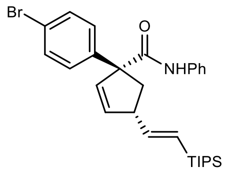

(1R,4S)-1-(4-bromophenyl)-N-phenyl-4-((E)-2-(triisopropylsilyl)vinyl)cyclopent-2-ene-1-carboxamide (3f)

43.5 mg, 83% yield, 92% *ee*, d.r. > 20:1, E/Z > 20:1; R_f = 0.7 (PE/EA = 10/1); Colorless oil. $[\alpha]_D^{20}$ = -68 (*c* = 0.10, MeOH).

^{1}H NMR (300 MHz, $CDCl_3$) δ 7.48 – 7.38 (m, 4H), 7.28 – 7.23 (m, 4H), 7.10 – 7.04 (m, 2H), 6.11 (dd, *J* = 5.5, 2.3 Hz, 1H), 6.09 – 6.03 (dd, 1H), 6.01 (dd, *J* = 4.7, 2.7 Hz, 1H), 5.59 (dd, *J* = 18.8, 1.1 Hz, 1H), 3.45 (q, *J* = 7.2 Hz, 1H), 2.78 (dd, *J* = 13.3, 6.6 Hz, 1H), 2.44 (dd, *J* = 13.3, 7.8 Hz, 1H), 1.08 – 0.95 (m, 21H) ppm.

^{13}C NMR (75 MHz, $CDCl_3$) δ 172.4, 149.7, 142.4, 138.9, 137.8, 132.1, 131.9, 129.0, 128.5, 124.5, 124.4, 121.4, 119.8, 65.6, 52.2, 43.0, 18.6, 10.8 ppm.

HRMS (ESI) calcd for $[C_{29}H_{38}BrNOSi+H]^+$ 524.1979, found 524.1978.

HPLC: Daicel Chiralcel IC-3, *n*-hexane/isopropanol 98/2, flow rate = 0.5 mL/min, uv-vis λ = 250 nm, t_{R1} = 15.4 min (minor), t_{R2} = 17.5 min (major).

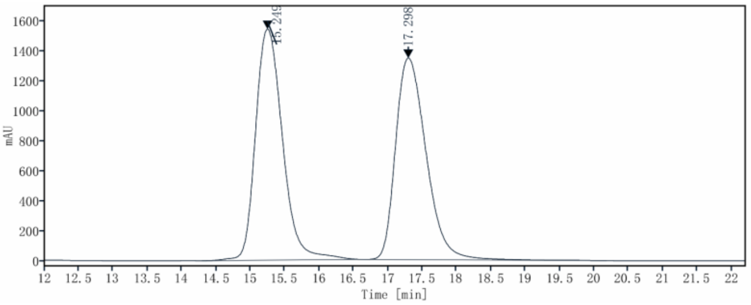

RetTime[min]	Type	Width[min]	Area[mAU*s]	Height[mAU]	Area%
15.249	VM m	0.4285	42124.6015	1543.0303	50.3678
17.298	MM m	0.4847	41509.3711	1346.1487	49.6322

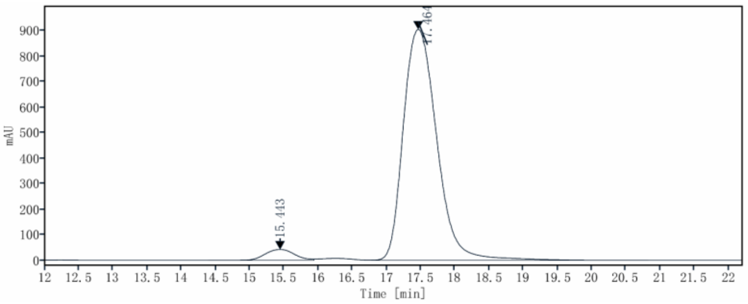

RetTime[min]	Type	Width[min]	Area[mAU*s]	Height[mAU]	Area%
15.443	BV	1.5467	1267.8033	42.6011	3.9540
17.464	VB	4.0426	30795.6367	904.1663	96.0460

Supplementary Figure 7. HPLC Trace of **3f**

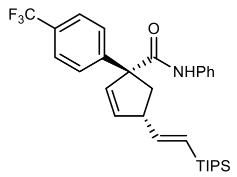

(1R,4S)-N-phenyl-1-(4-(trifluoromethyl)phenyl)-4-((E)-2-(triisopropylsilyl)vinyl)cyclopent-2-en e-1-carboxamide (3g)

49.8 mg, 97% yield, 94% *ee*, d.r. > 20:1, E/Z > 20:1; R_f = 0.7 (PE/EA = 10/1); Colorless oil. $[\alpha]_D^{20}$ = -60 (*c* = 0.18, MeOH).

^{1}H NMR (300 MHz, $CDCl_3$) δ 7.59 (d, *J* = 8.4 Hz, 2H), 7.51 (d, *J* = 8.3 Hz, 2H), 7.44 – 7.39 (m, 2H), 7.30 – 7.23 (m, 2H), 7.14 (brs, 1H), 7.10 – 7.05 (m, 1H), 6.16 (dd, *J* = 5.6, 2.2 Hz, 1H), 6.10 – 6.08 (m, 1H), 6.07 – 6.01 (m, 1H), 5.61 (dd, *J* = 18.8, 1.1 Hz, 1H), 3.52 – 3.43 (m, 1H), 2.81 (dd, *J* = 13.4, 6.4 Hz, 1H), 2.50 (dd, *J* = 13.3, 8.0 Hz, 1H), 1.13 – 0.90 (m, 21H) ppm.

^{13}C NMR (75 MHz, $CDCl_3$) δ 172.2, 149.5, 147.4, 139.4, 137.7, 131.8, 129.5 (d, *J* = 32.4 Hz), 129.0, 127.1, 125.9, 125.7 (q, *J* = 3.4 Hz), 124.6, 122.3, 119.9, 65.9, 52.2, 43.2, 18.6, 10.8 ppm.

^{19}F NMR (282 MHz, $CDCl_3$) δ -62.48 ppm.

HRMS (ESI) calcd for $[C_{30}H_{38}F_3NOSi+H]^+$ 514.2748, found 514.2761.

HPLC: Daicel Chiralcel IA-3, *n*-hexane/isopropanol 90/10, flow rate = 0.5 mL/min, uv-vis λ = 250 nm, t_{R1} = 8.6 min (major), t_{R2} = 12.3 min (minor).

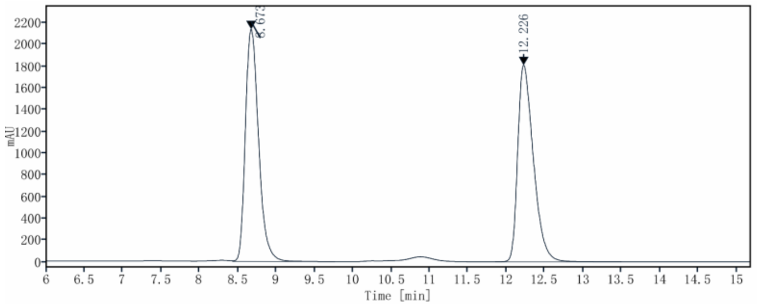

RetTime[min]	Type	Width[min]	Area[mAU*s]	Height[mAU]	Area%
8.673	VB	1.3324	25528.4042	2135.7430	49.9041
12.226	BB	1.6433	25626.5307	1807.3012	50.0959

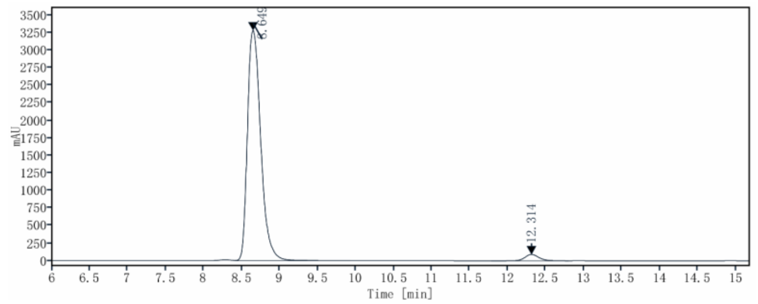

RetTime[min]	Type	Width[min]	Area[mAU*s]	Height[mAU]	Area%
8.649	VB	1.5080	39895.0174	3281.9320	97.1643
12.314	BV	1.0179	1164.3318	90.6486	2.8357

Supplementary Figure 8. HPLC Trace of **3g**

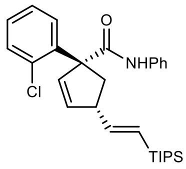

(1R,4S)-1-(2-chlorophenyl)-N-phenyl-4-((E)-2-(triisopropylsilyl)vinyl)cyclopent-2-ene-1-carboxamide (3h)

38.4 mg, 80% yield, 91% *ee*, d.r. > 20:1, E/Z > 20:1; R_f = 0.6 (PE/EA = 10/1); White solid, m.p. 107 – 109 °C. $[\alpha]_D^{20}$ = +44 (*c* = 0.12, MeOH).

^{1}H NMR (300 MHz, $CDCl_3$) δ 7.50 – 7.42 (m, 2H), 7.39 – 7.36 (m, 2H), 7.33 – 7.23 (m, 4H), 7.09 – 7.03 (m, 1H), 6.92 (brs, 1H), 6.13 (dd, *J* = 18.7, 7.8 Hz, 1H), 6.06 (dd, *J* = 5.5, 2.5 Hz, 1H), 6.01 (dd, *J* = 5.5, 1.8 Hz, 1H), 5.60 (d, *J* = 18.7 Hz, 1H), 3.42 (q, *J* = 7.8 Hz, 1H), 3.04 (dd, *J* = 13.7, 7.6 Hz, 1H), 2.46 (dd, *J* = 13.7, 7.9 Hz, 1H), 1.17 – 0.93 (m, 21H) ppm.

^{13}C NMR (75 MHz, $CDCl_3$) δ 171.9, 150.3, 140.8, 139.6, 138.0, 134.4, 132.2, 131.3, 129.2, 128.9, 128.6, 127.2, 124.4, 120.2, 66.4, 52.8, 40.4, 18.7, 10.9 ppm.

HRMS (ESI) calcd for $[C_{29}H_{38}ClNOSi+H]^+$ 480.2484, found 480.2492.

HPLC: Daicel Chiralcel OD-H, *n*-hexane/isopropanol 95/5, flow rate = 0.5 mL/min, uv-vis λ = 250 nm, t_{R1} = 9.8 min (major), t_{R2} = 13.4 min (minor).

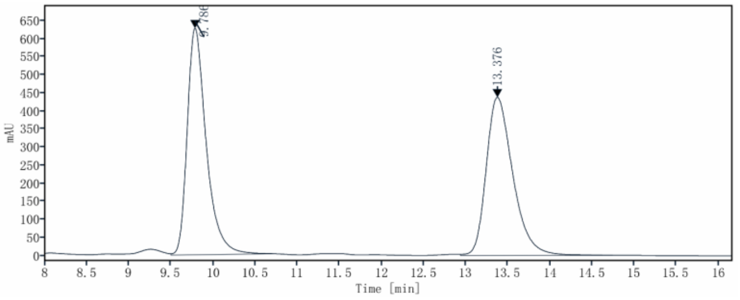

RetTime[min]	Type	Width[min]	Area[mAU*s]	Height[mAU]	Area%
9.786	VM m	0.2372	9835.7045	626.3822	50.5700
13.376	VB	1.8057	9613.9592	437.1564	49.4300

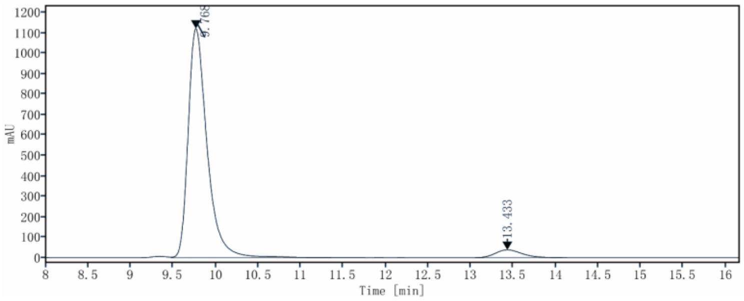

RetTime[min]	Type	Width[min]	Area[mAU*s]	Height[mAU]	Area%
9.768	VB	1.8794	17328.7594	1120.7044	95.4310
13.433	MM m	0.3349	829.6547	38.2762	4.5690

Supplementary Figure 9. HPLC Trace of **3h**

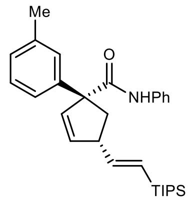

(1R,4S)-N-phenyl-1-(m-tolyl)-4-((E)-2-(triisopropylsilyl)vinyl)cyclopent-2-ene-1-carboxamide (3i)

43.7 mg, 95% yield, 90% *ee*, d.r. > 20:1, E/Z > 20:1; R_f = 0.7 (PE/EA = 10/1); Colorless oil. $[\alpha]_D^{20}$ = -59 (*c* = 0.17, MeOH).

^{1}H NMR (300 MHz, $CDCl_3$) δ 7.42 – 7.39 (m, 2H), 7.28 – 7.16 (m, 5H), 7.10 – 7.02 (m, 3H), 6.15 – 6.12 (m, 1H), 6.12 – 6.03 (m, 1H), 5.97 (dd, *J* = 5.5, 2.0 Hz, 1H), 5.58 (d, *J* = 18.7 Hz, 1H), 3.45 (q, *J* = 7.5 Hz, 1H), 2.80 (dd, *J* = 13.1, 6.9 Hz, 1H), 2.48 (dd, *J* = 13.2, 7.8 Hz, 1H), 2.34 (s, 3H), 1.12 – 0.95 (m, 21H) ppm.

^{13}C NMR (75 MHz, $CDCl_3$) δ 173.3, 150.5, 143.4, 138.8, 138.3, 138.1, 133.1, 129.1, 129.0, 128.4, 127.6, 124.4, 124.2, 123.8, 119.9, 66.2, 52.6, 42.9, 21.8, 18.8, 11.0 ppm.

HRMS (ESI) calcd for $[C_{30}H_{41}NOSi+H]^+$ 460.3030, found 460.3037.

HPLC: Daicel Chiralcel IC-3, *n*-hexane/isopropanol 98/2, flow rate = 0.5 mL/min, uv-vis λ = 250 nm, t_{R1} = 15.0 min (minor), t_{R2} = 19.7 min (major).

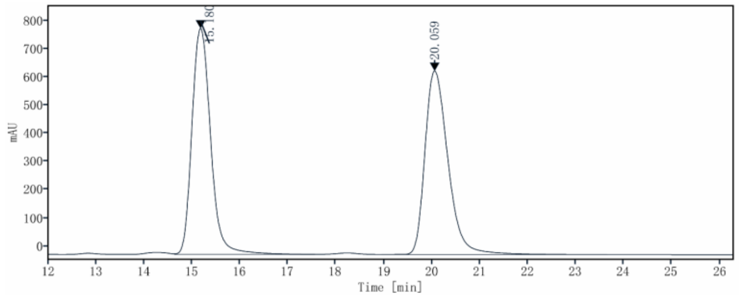

RetTime[min]	Type	Width[min]	Area[mAU*s]	Height[mAU]	Area%
15.180	VB	2.9233	21782.2704	804.1624	49.9576
20.059	BB	4.6867	21819.2014	653.2559	50.0424

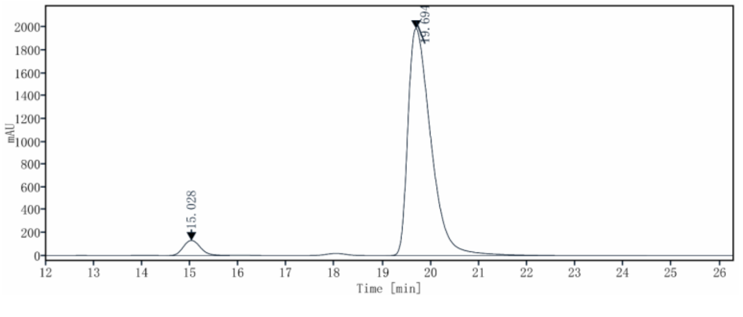

RetTime[min]	Type	Width[min]	Area[mAU*s]	Height[mAU]	Area%
15.028	VV	1.3205	3361.7110	133.2637	4.8519
19.694	BB	5.4033	65924.5594	1990.8625	95.1481

Supplementary Figure 10. HPLC Trace of **3i**

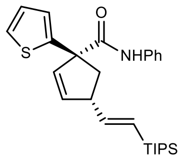

(1S,4S)-N-phenyl-1-(thiophen-2-yl)-4-((E)-2-(triisopropylsilyl)vinyl)cyclopent-2-ene-1-carboxamide (3j)

40.2 mg, 89% yield, 94% *ee*, d.r. > 20:1, E/Z > 20:1; R_f = 0.7 (PE/EA = 10/1); Colorless oil. $[\alpha]_D^{20}$ = -76 (*c* = 0.14, MeOH).

^{1}H NMR (300 MHz, $CDCl_3$) δ 7.47 – 7.42 (m, 2H), 7.36 (brs, 1H), 7.31 – 7.24 (m, 3H), 7.10 – 7.04 (m, 1H), 7.03 – 6.96 (m, 2H), 6.15 (dd, *J* = 5.5, 2.4 Hz, 1H), 6.09 – 6.00 (m, 2H), 5.61 (dd, *J* = 18.8, 1.1 Hz, 1H), 3.55 (m, 1H), 2.69 (dd, *J* = 13.1, 7.2 Hz, 1H), 2.61 (dd, *J* = 13.1, 7.7 Hz, 1H), 1.08 – 0.95 (m, 21H) ppm.

^{13}C NMR (75 MHz, $CDCl_3$) δ 172.1, 149.6, 146.9, 138.6, 137.7, 132.9, 128.9, 127.1, 125.1, 124.7, 124.5, 124.4, 119.8, 62.9, 52.2, 44.7, 18.6, 10.8 ppm.

HRMS (ESI) calcd for $[C_{27}H_{37}NOSSi+H]^+$ 452.2438, found 452.2449.

HPLC: Daicel Chiralcel OD-H, *n*-hexane/isopropanol 98/2, flow rate = 0.5 mL/min, uv-vis λ = 250 nm, t_{R1} = 15.0 min (major), t_{R2} = 17.5 min (minor).

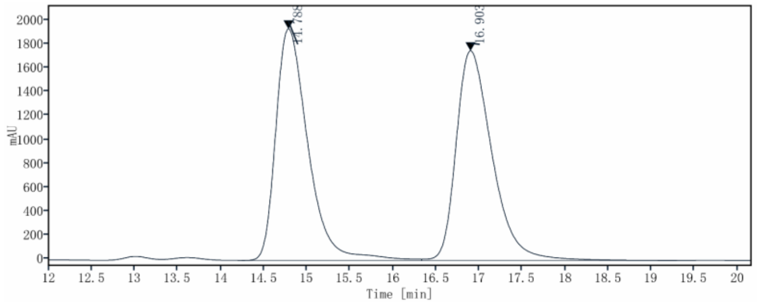

RetTime[min]	Type	Width[min]	Area[mAU*s]	Height[mAU]	Area%
14.788	BV	2.0882	50387.5573	1936.2403	49.8789
16.903	VB	2.9642	50632.1374	1752.6231	50.1211

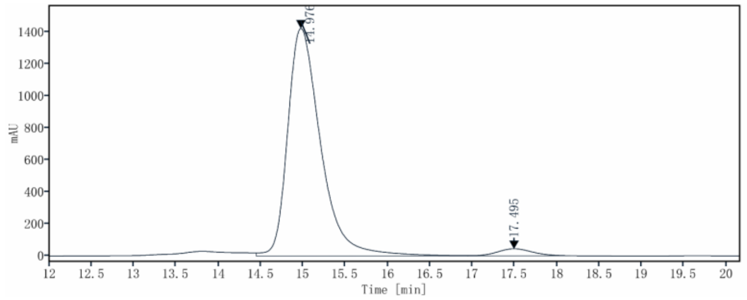

RetTime[min]	Type	Width[min]	Area[mAU*s]	Height[mAU]	Area%
14.976	VM m	0.4130	38584.2647	1424.6216	96.8570
17.495	MM m	0.4432	1252.0619	43.8441	3.1430

Supplementary Figure 11. HPLC Trace of **3j**

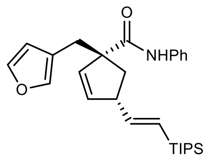

(1S,4S)-1-(furan-3-ylmethyl)-N-phenyl-4-((E)-2-(triisopropylsilyl)vinyl)cyclopent-2-ene-1-carboxamide (3k)

39.1 mg, 87% yield, 94% *ee*, d.r. > 20:1, E/Z > 20:1; R_f = 0.7 (PE/EA = 10/1); Colorless oil. $[\alpha]_D^{20}$ = -22 (*c* = 0.10, MeOH).

^{1}H NMR (300 MHz, $CDCl_3$) δ 7.47 – 7.43 (m, 2H), 7.32 – 7.25 (m, 5H), 7.12 - 7.05 (m, 1H), 6.26 (d, *J* = 1.8 Hz, 1H), 6.03 (dd, *J* = 5.6, 2.3 Hz, 1H), 5.96 (dd, *J* = 18.8, 7.0 Hz, 1H), 5.83 (dd, *J* = 5.6, 2.1 Hz, 1H), 5.55 (dd, *J* = 18.8, 1.2 Hz, 1H), 3.48 – 3.39 (m, 1H), 3.23 (d, *J* = 13.9 Hz, 1H), 2.65 (d, *J* = 14.0 Hz, 1H), 2.34 (dd, *J* = 13.7, 8.5 Hz, 1H), 2.07 (dd, *J* = 13.7, 5.8 Hz, 1H), 1.00 - 0.94 (m, 21H) ppm.

^{13}C NMR (75 MHz, $CDCl_3$) δ 174.4, 150.0, 142.5, 140.8, 140.0, 137.7, 132.3, 128.9, 124.4, 123.9, 121.0, 119.9, 112.2, 61.7, 51.9, 42.3, 32.8, 18.6, 10.8 ppm.

HRMS (ESI) calcd for $[C_{28}H_{39}NO_2Si+H]^+$ 450.2823, found 450.2820.

HPLC: Daicel Chiralcel OD-H, *n*-hexane/isopropanol 98/2, flow rate = 0.5 mL/min, uv-vis λ = 250 nm, t_{R1} = 15.8 min (minor), t_{R2} = 17.6 min (major).

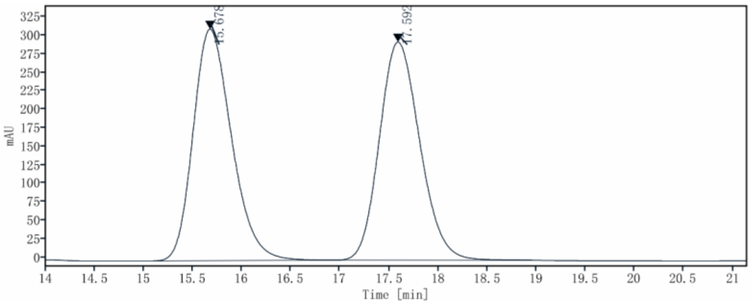

RetTime[min]	Type	Width[min]	Area[mAU*s]	Height[mAU]	Area%
15.678	BB	1.8736	8524.4610	312.9544	50.2949
17.592	BM m	0.4455	8424.4854	294.6740	49.7051

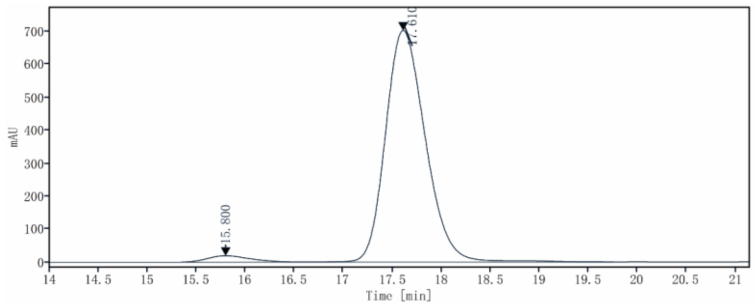

RetTime[min]	Type	Width[min]	Area[mAU*s]	Height[mAU]	Area%
15.800	BB	1.4606	598.0490	19.6099	2.9237
17.610	BB	3.1183	19857.3220	702.4930	97.0763

Supplementary Figure 12. HPLC Trace of **3k**

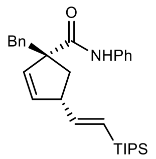

(1S,4S)-1-benzyl-N-phenyl-4-((E)-2-(triisopropylsilyl)vinyl)cyclopent-2-ene-1-carboxamide (3l)

39.1 mg, 85% yield, 96% *ee*, d.r. > 20:1, E/Z > 20:1; R_f = 0.7 (PE/EA = 10/1); Colorless oil. $[\alpha]_D^{20}$ = -17 (*c* = 0.10, MeOH).

^{1}H NMR (300 MHz, $CDCl_3$) δ 7.43 – 7.39 (m, 2H), 7.30 – 7.17 (m, 7H), 7.13 (brs, 1H), 7.07 (t, *J* = 7.3 Hz, 1H), 5.99 – 5.90 (m, 2H), 5.78 (dd, *J* = 5.6, 2.1 Hz, 1H), 5.53 (dd, *J* = 18.7, 1.2 Hz, 1H), 3.44 (d, *J* = 13.1 Hz, 1H), 3.37 – 3.28 (m, 1H), 2.82 (d, *J* = 13.1 Hz, 1H), 2.41 (dd, *J* = 13.6, 8.3 Hz, 1H), 2.08 (dd, *J* = 13.6, 6.2 Hz, 1H), 1.05 – 0.96 (m, 21H) ppm.

^{13}C NMR (75 MHz, $CDCl_3$) δ 174.3, 150.1, 139.7, 138.0, 137.7, 132.2, 130.3, 128.9, 128.1, 126.5, 124.4, 123.8, 120.0, 62.3, 51.8, 43.5, 42.5, 18.6, 10.8 ppm.

HRMS (ESI) calcd for $[C_{30}H_{41}NOSi+H]^+$ 460.3030, found 460.3028.

HPLC: Daicel Chiralcel OD-H, *n*-hexane/isopropanol 99.5/0.5, flow rate = 0.5 mL/min, uv-vis λ = 250 nm, t_{R1} = 38.2 min (minor), t_{R2} = 40.9 min (major).

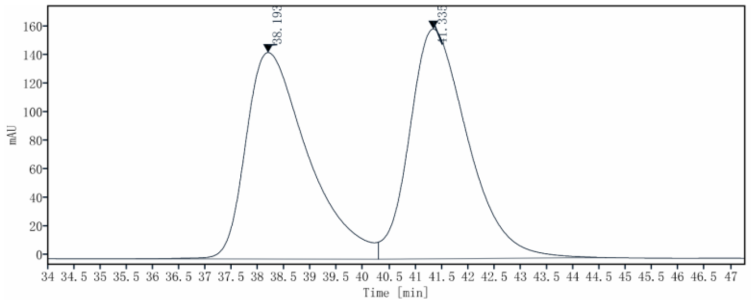

RetTime[min]	Type	Width[min]	Area[mAU*s]	Height[mAU]	Area%
38.193	BM m	1.2295	11861.3889	144.5234	48.9021
41.335	MM m	1.1822	12393.9744	160.8048	51.0979

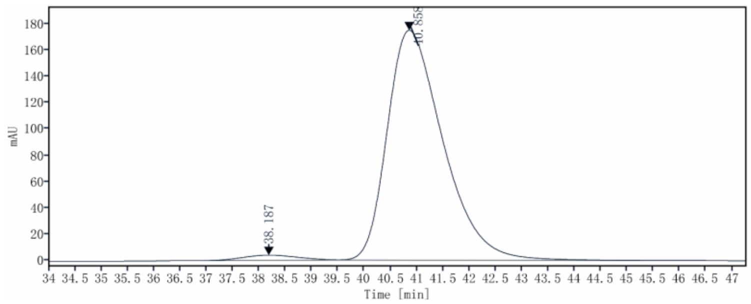

RetTime[min]	Type	Width[min]	Area[mAU*s]	Height[mAU]	Area%
38.187	BB	2.6100	280.1827	3.9171	2.1351
40.858	BB	5.7367	12842.4442	174.4125	97.8649

Supplementary Figure 13. HPLC Trace of **3l**

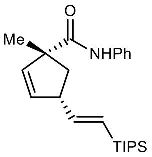

(1R,4S)-1-methyl-N-phenyl-4-((E)-2-(triisopropylsilyl)vinyl)cyclopent-2-ene-1-carboxamide (3m)

32.2 mg, 84% yield, 95% *ee*, d.r. > 20:1, E/Z > 20:1; R_f = 0.6 (PE/EA = 10/1); Colorless oil. $[\alpha]_D^{20}$ = -15 (*c* = 0.16, MeOH).

^{1}H NMR (300 MHz, $CDCl_3$) δ 7.51 – 7.48 (m, 2H), 7.35 (brs, 1H), 7.29 (t, *J* = 7.9 Hz, 2H), 7.08 (t, *J* = 7.4 Hz, 1H), 6.02 (dd, *J* = 17.9, 6.1 Hz, 1H), 5.97 – 5.95 (m, 1H), 5.85 (dd, *J* = 5.5, 2.1 Hz, 1H), 5.58 (dd, *J* = 18.8, 1.2 Hz, 1H), 3.61 – 3.53 (m, 1H), 2.22 (dd, *J* = 13.5, 8.4 Hz, 1H), 2.10 (dd, *J* = 13.5, 6.0 Hz, 1H), 1.40 (s, 3H), 1.06 – 0.94 (m, 21H) ppm.

^{13}C NMR (75 MHz, $CDCl_3$) δ 175.3, 150.3, 138.0, 138.0, 134.9, 128.9, 124.2, 123.6, 119.7, 56.8, 51.9, 43.9, 23.8, 18.6, 10.8 ppm.

HRMS (ESI) calcd for $[C_{24}H_{37}NOSi+H]^+$ 384.2717, found 384.2721.

HPLC: Daicel Chiralcel OD-H, *n*-hexane/isopropanol 98/2, flow rate = 0.5 mL/min, uv-vis λ = 250 nm, t_{R1} = 13.8 min (minor), t_{R2} = 16.1 min (major).

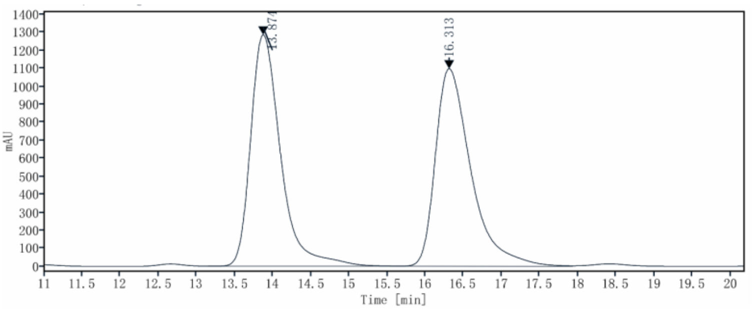

RetTime[min]	Type	Width[min]	Area[mAU*s]	Height[mAU]	Area%
13.874	BB	2.5167	35122.7971	1286.0943	49.7429
16.313	BV	2.2619	35485.8318	1097.6839	50.2571

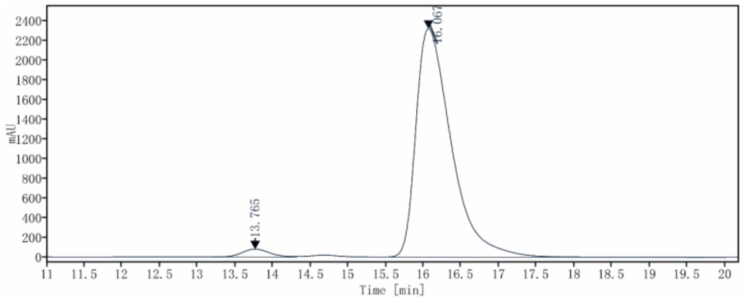

RetTime[min]	Type	Width[min]	Area[mAU*s]	Height[mAU]	Area%
13.765	MV m	0.3935	2013.4453	79.7620	2.5900
16.067	BB	4.2573	75725.5578	2322.1526	97.4100

Supplementary Figure 14. HPLC Trace of **3m**

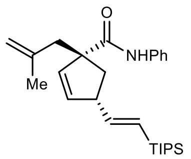

(1S,4S)-1-(2-methylallyl)-N-phenyl-4-((E)-2-(triisopropylsilyl)vinyl)cyclopent-2-ene-1-carboxamide (3n)

35.6 mg, 84% yield, 92% *ee*, d.r. > 20:1, E/Z > 20:1; R_f = 0.8 (PE/EA = 10/1); Colorless oil. $[\alpha]_D^{20}$ = -70 (*c* = 0.10, MeOH).

^{1}H NMR (300 MHz, $CDCl_3$) δ 7.51 – 7.47 (m, 2H), 7.39 (brs, 1H), 7.29 (t, *J* = 7.9 Hz, 2H), 7.11 – 7.06 (m, 1H), 6.02 – 5.92 (m, 3H), 5.56 (dd, *J* = 18.8, 1.2 Hz, 1H), 4.83 (s, 1H), 4.73 (s, 1H), 3.56 – 3.47 (m, 1H), 2.92 (d, *J* = 13.7 Hz, 1H), 2.37 – 2.30 (m, 1H), 2.27 (d, *J* = 13.4 Hz, 1H), 2.06 (dd, *J* = 13.6, 5.9 Hz, 1H), 1.72 (s, 3H), 1.09 – 0.90 (m, 21H) ppm.

^{13}C NMR (75 MHz, $CDCl_3$) δ 174.5, 150.2, 142.7, 139.3, 137.9, 132.6, 128.9, 124.3, 123.8, 119.8, 114.1, 60.5, 51.7, 45.5, 43.1, 24.0, 18.6, 10.8 ppm.

HRMS (ESI) calcd for $[C_{27}H_{41}NOSi+H]^+$ 424.3030, found 424.3035.

HPLC: Daicel Chiralcel OD-H, *n*-hexane/isopropanol 98/2, flow rate = 0.5 mL/min, uv-vis λ = 250 nm, t_{R1} = 9.7 min (minor), t_{R2} = 12.1 min (major).

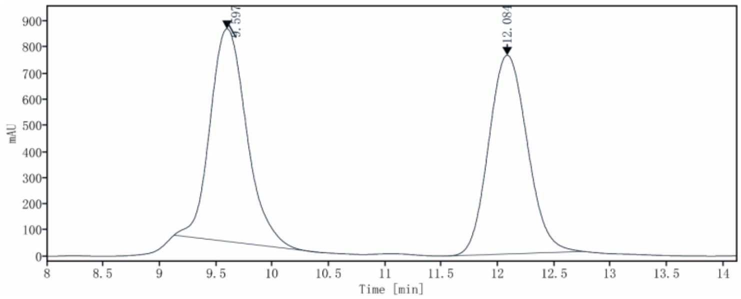

RetTime[min]	Type	Width[min]	Area[mAU*s]	Height[mAU]	Area%
9.597	MM m	0.3519	18427.4813	814.6608	50.3981
12.084	VM m	0.3767	18136.3869	761.8676	49.6019

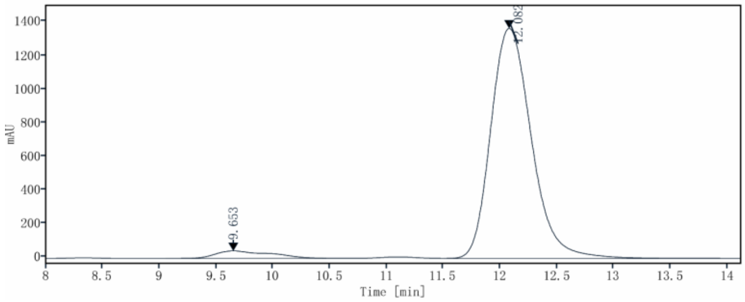

RetTime[min]	Type	Width[min]	Area[mAU*s]	Height[mAU]	Area%
9.653	MM m	0.4721	1485.3162	44.1922	4.1771
12.082	VB	2.9955	34072.8425	1366.5168	95.8229

Supplementary Figure 15. HPLC Trace of **3n**

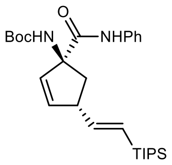

tert-butyl ((1S,4S)-1-(phenylcarbamoyl)-4-((E)-2-(triisopropylsilyl)vinyl)cyclopent-2-en-1-yl)carbamate (3o)

27.6 mg, 57% yield, 93% *ee*, d.r. > 20:1, E/Z > 20:1; R_f = 0.6 (toluene/EA = 9/1); White solid, m.p. 145 – 147 ^{o}C. $[\alpha]_D^{20}$ = -78 (c = 0.14, MeOH).

^{1}H NMR (300 MHz, $CDCl_3$) δ 8.29 (brs, 1H), 7.50 (d, *J* = 8.0 Hz, 2H), 7.31 (t, *J* = 7.9 Hz, 2H), 7.10 (t, *J* = 7.4 Hz, 1H), 6.10 (brs, 1H), 6.06 – 5.97 (m, 2H), 5.62 (dd, *J* = 18.8, 1.1 Hz, 1H), 5.53 (s, 1H), 3.72 (s, 1H), 2.50 – 2.35 (m, 2H), 1.44 (s, 9H), 1.10 – 0.94 (m, 21H) ppm.

^{13}C NMR (75 MHz, $CDCl_3$) δ 171.3, 155.3, 149.6, 140.8, 137.9, 131.3, 129.0, 124.6, 124.3, 119.8, 80.4, 71.9, 51.8, 41.8, 28.3, 18.6, 10.8 ppm.

HRMS (ESI) calcd for $[C_{28}H_{44}N_2O_3Si+Na]^+$ 507.3013, found 507.3009.

HPLC: Daicel Chiralcel AD-H, n-hexane/isopropanol 95/5, flow rate = 0.5 mL/min, uv-vis λ = 250 nm, t_{R1} = 16.5 min (minor), t_{R2} = 21.2 min (major).

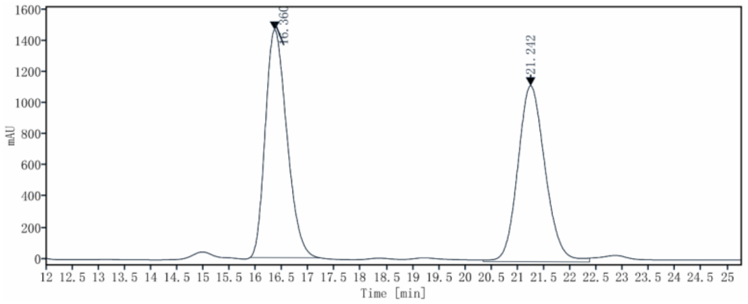

RetTime[min]	Type	Width[min]	Area[mAU*s]	Height[mAU]	Area%
16.360	MM m	0.4457	41783.0877	1460.8960	50.4697
21.242	MM m	0.5598	41005.2937	1128.8799	49.5303

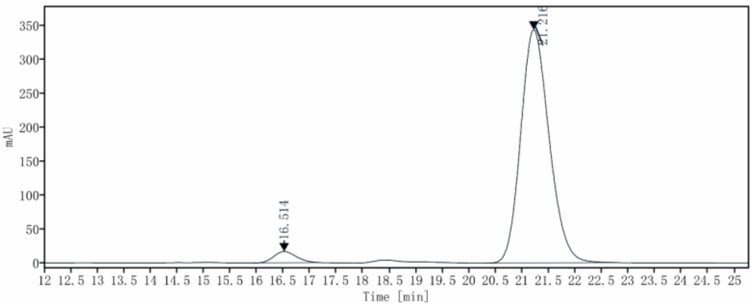

RetTime[min]	Type	Width[min]	Area[mAU*s]	Height[mAU]	Area%
16.514	MM m	0.4558	490.0769	16.6796	3.6740
21.216	BM m	0.5756	12848.9377	344.9473	96.3260

Supplementary Figure 16. HPLC Trace of **3o**

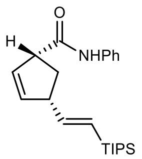

(1R,4S)-N-phenyl-4-((E)-2-(triisopropylsilyl)vinyl)cyclopent-2-ene-1-carboxamide (3p)

33.3 mg, 90% yield, 94% *ee*, d.r. > 20:1, E/Z > 20:1; R_f = 0.4 (PE/EA = 10/1); White solid, m.p. 106 – 108 °C; $[\alpha]_D^{20}$ = 3.8 (*c* = 0.65, MeOH).

^{1}H NMR (300 MHz, $CDCl_3$) δ 7.53 – 7.49 (m, 2H), 7.45 (brs, 1H), 7.29 (t, *J* = 7.9 Hz, 2H), 7.12 – 7.06 (m, 1H), 6.03 (dd, *J* = 18.8, 7.2 Hz, 1H), 5.96 (dt, *J* = 5.6, 2.2 Hz, 1H), 5.84 (dt, *J* = 5.5, 2.2 Hz, 1H), 5.58 (dd, *J* = 18.8, 1.2 Hz, 1H), 3.57 (ddq, *J* = 9.3, 7.0, 1.9 Hz, 1H), 3.45 (m, 1H), 2.58 (dt, *J* = 13.3, 8.7 Hz, 1H), 1.90 (dt, *J* = 13.6, 7.0 Hz, 1H), 1.09 – 0.88 (m, 21H) ppm.

^{13}C NMR (75 MHz, $CDCl_3$) δ 172.8, 150.8, 139.2, 137.9, 129.1, 129.0, 124.3, 123.7, 119.9, 53.6, 52.7, 35.1, 18.6, 10.8 ppm.

HRMS (ESI) calcd for $[C_{23}H_{35}NOSi+H]^+$ 370.2561, found 370.2559.

HPLC: Daicel Chiralcel OD-H, *n*-hexane/isopropanol 90/10, flow rate = 1.0 mL/min, uv-vis λ = 250 nm, t_{R1} = 7.3 min (minor), t_{R2} = 10.9 min (major).

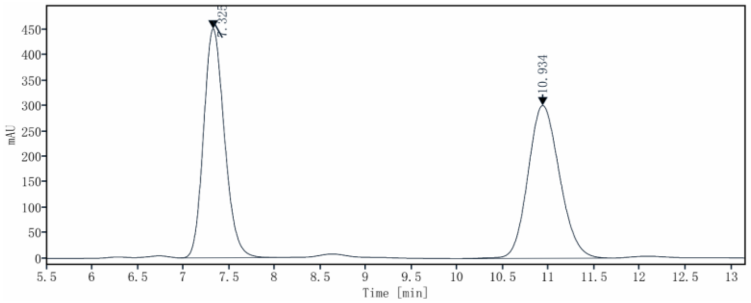

RetTime[min]	Type	Width[min]	Area[mAU*s]	Height[mAU]	Area%
7.325	BB	1.0490	7128.8303	449.6184	49.7339
10.934	BB	1.6485	7205.1236	300.0031	50.2661

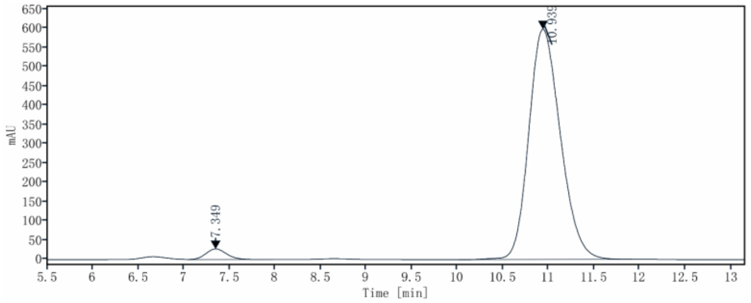

RetTime[min]	Type	Width[min]	Area[mAU*s]	Height[mAU]	Area%
7.349	VB	0.9351	438.5566	27.5833	2.9596
10.939	BB	1.9000	14379.4797	596.7542	97.0404

Supplementary Figure 17. HPLC Trace of **3p**

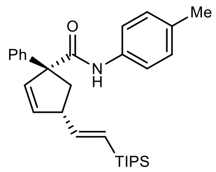

(1R,4S)-1-phenyl-N-(p-tolyl)-4-((E)-2-(triisopropylsilyl)vinyl)cyclopent-2-ene-1-carboxamide (3q)

42.8 mg, 93% yield, 93% *ee*, d.r. > 20:1, E/Z > 20:1; R_f = 0.7 (PE/EA = 10/1); Colorless oil. $[\alpha]_D^{20}$ = -53 (*c* = 0.14, MeOH).

^{1}H NMR (300 MHz, $CDCl_3$) δ 7.40 -7.34 (m, 4H), 7.30 – 7.25 (m, 3H), 7.07 – 7.04 (m, 2H), 6.99 (brs, 1H), 6.13 (dd, *J* = 5.6, 2.4 Hz, 1H), 6.07 (dd, *J* = 18.8, 7.6 Hz, 1H), 5.97 (dd, *J* = 5.5, 2.0 Hz, 1H), 5.58 (dd, *J* = 18.7, 1.1 Hz, 1H), 3.49 – 3.40 (m, 1H), 2.80 (dd, *J* = 13.2, 7.0 Hz, 1H), 2.48 (dd, *J* = 13.2, 7.8 Hz, 1H), 2.27 (s, 3H), 1.15 – 0.93 (m, 21H) ppm.

^{13}C NMR (75 MHz, $CDCl_3$) δ 173.0, 150.3, 143.4, 138.0, 135.5, 133.8, 132.9, 129.4, 128.9, 127.3, 126.7, 124.0, 119.8, 66.0, 52.4, 42.8, 20.9, 18.7, 10.9 ppm.

HRMS (ESI) calcd for $[C_{30}H_{41}NOSi+H]^+$ 460.3030, found 460.3040.

HPLC: Daicel Chiralcel OD-H, *n*-hexane/isopropanol 95/5, flow rate = 0.5 mL/min, uv-vis λ = 250 nm, t_{R1} = 9.3 min (major), t_{R2} = 11.6 min (minor).

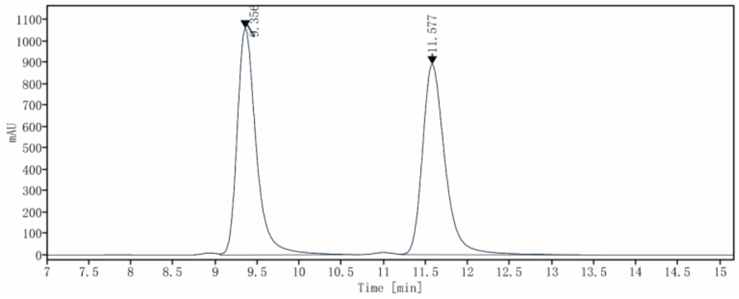

RetTime[min]	Type	Width[min]	Area[mAU*s]	Height[mAU]	Area%
9.356	VB	1.6540	16640.8320	1060.6679	50.0423
11.577	VB	2.3761	16612.6784	891.0106	49.9577

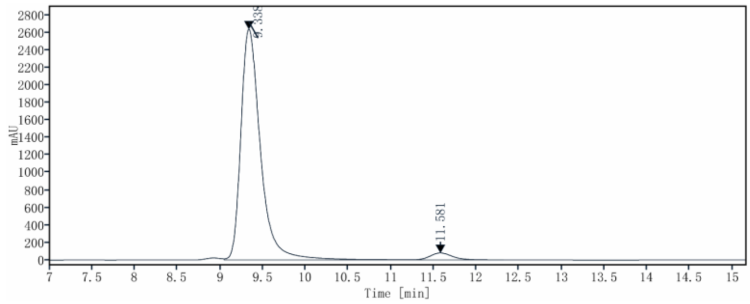

RetTime[min]	Type	Width[min]	Area[mAU*s]	Height[mAU]	Area%
9.338	VM m	0.2435	42227.5156	2641.7894	96.6338
11.581	MM m	0.2810	1470.9735	80.1791	3.3662

Supplementary Figure 18. HPLC Trace of **3q**

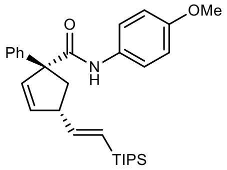

(1R,4S)-N-(4-methoxyphenyl)-1-phenyl-4-((E)-2-(triisopropylsilyl)vinyl)cyclopent-2-ene-1-carboxamide (3r)

42.8 mg, 90% yield, 93% *ee*, d.r. > 20:1, E/Z > 20:1; R_f = 0.5 (PE/EA = 10/1); Colorless oil. $[\alpha]_D^{20}$ = -45 (*c* = 0.20, MeOH).

^{1}H NMR (300 MHz, $CDCl_3$) δ 7.40 – 7.24 (m, 7H), 6.98 (brs, 1H), 6.81 – 6.76 (m, 2H), 6.14 (dd, *J* = 5.6, 2.4 Hz, 1H), 6.07 (dd, *J* = 18.8, 7.5 Hz, 1H), 5.98 (dd, *J* = 5.5, 2.0 Hz, 1H), 5.58 (dd, *J* = 18.7, 1.1 Hz, 1H), 3.74 (s, 3H), 3.49 – 3.40 (m, 1H), 2.80 (dd, *J* = 13.2, 7.0 Hz, 1H), 2.49 (dd, *J* = 13.2, 7.8 Hz, 1H), 1.12 – 0.95 (m, 21H) ppm.

^{13}C NMR (75 MHz, $CDCl_3$) δ 173.0, 156.4, 150.3, 143.4, 138.0, 133.0, 131.1, 128.9, 127.3, 126.7, 124.0, 121.6, 114.0, 65.9, 55.5, 52.4, 42.9, 18.7, 10.8 ppm.

HRMS (ESI) calcd for $[C_{30}H_{41}NO_2Si+H]^+$ 476.2979, found 476.2982.

HPLC: Daicel Chiralcel OD-H, *n*-hexane/isopropanol 95/5, flow rate = 0.5 mL/min, uv-vis λ = 250 nm, t_{R1} = 14.6 min (major), t_{R2} = 19.5 min (minor).

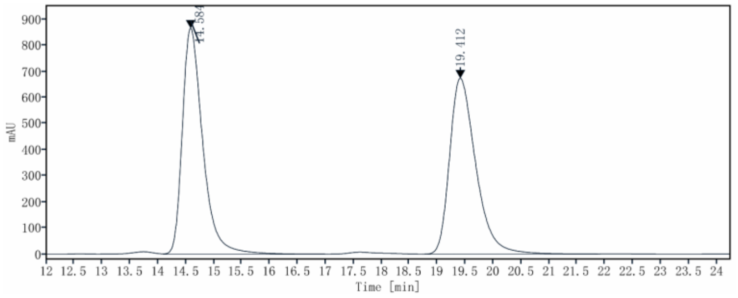

RetTime[min]	Type	Width[min]	Area[mAU*s]	Height[mAU]	Area%
14.584	VB	2.9510	21951.4075	864.3114	50.0344
19.412	BB	3.1450	21921.2385	673.0298	49.9656

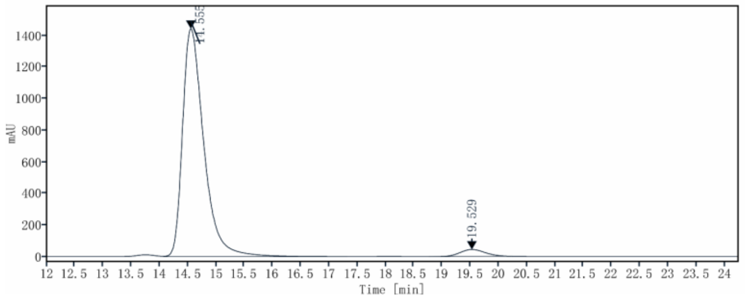

RetTime[min]	Type	Width[min]	Area[mAU*s]	Height[mAU]	Area%
14.555	VB	3.2458	37772.1254	1442.8146	96.4755
19.529	BM m	0.4841	1379.9157	44.4629	3.5245

Supplementary Figure 19. HPLC Trace of **3r**

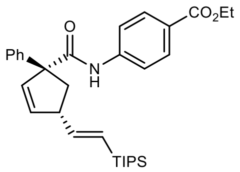

ethyl 4-((1R,4S)-1-phenyl-4-((E)-2-(triisopropylsilyl)vinyl)cyclopent-2-ene-1-carboxamido)benzoate (3s)

50.2 mg, 97% yield, 92% *ee*, d.r. > 20:1, E/Z > 20:1; R_f = 0.4 (PE/EA = 10/1); Colorless oil. $[\alpha]_D^{20}$ = -21 (*c* = 0.17, MeOH).

^{1}H NMR (300 MHz, $CDCl_3$) δ 7.97 – 7.92 (m, 2H), 7.52 – 7.47 (m, 2H), 7.41 – 7.33 (m, 4H), 7.32 – 7.29 (m, 1H), 7.22 (brs, 1H), 6.15 (dd, *J* = 5.6, 2.3 Hz, 1H), 6.07 (dd, 1H), 6.02 (dd, *J* = 5.5 Hz, 1H), 5.59 (dd, *J* = 18.7, 1.1 Hz, 1H), 4.33 (q, *J* = 7.1 Hz, 2H), 3.51 – 3.43 (m, 1H), 2.83 (dd, *J* = 13.2, 6.6 Hz, 1H), 2.48 (dd, *J* = 13.2, 7.8 Hz, 1H), 1.37 (t, *J* = 7.1 Hz, 3H), 1.11 – 0.95 (m, 21H) ppm.

^{13}C NMR (75 MHz, $CDCl_3$) δ 173.2, 166.1, 150.0, 142.9, 142.1, 138.6, 132.3, 130.7, 129.1, 127.5, 126.6, 125.9, 124.2, 118.7, 66.2, 60.9, 52.3, 42.8, 18.6, 14.3, 10.8 ppm.

HRMS (ESI) calcd for $[C_{32}H_{43}NO_3Si+H]^+$ 518.3085, found 518.3097.

HPLC: Daicel Chiralcel OD-H, *n*-hexane/isopropanol 95/5, flow rate = 0.5 mL/min, uv-vis λ = 250 nm, t_{R1} = 17.8 min (minor), t_{R2} = 22.6 min (major).

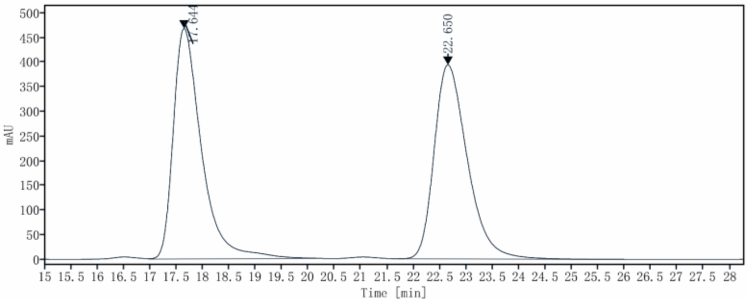

RetTime[min]	Type	Width[min]	Area[mAU*s]	Height[mAU]	Area%
17.644	VM m	0.5625	17435.3217	468.1937	50.2602
22.650	BB	4.3067	17254.8253	394.2125	49.7398

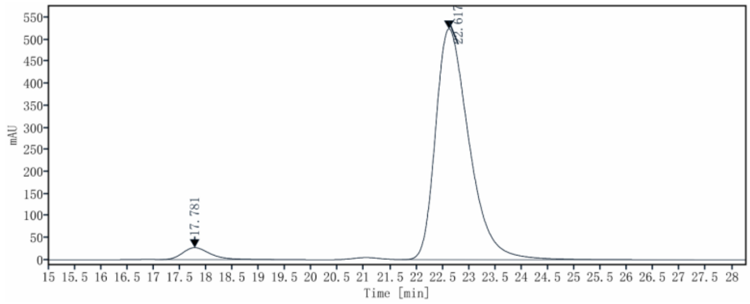

RetTime[min]	Type	Width[min]	Area[mAU*s]	Height[mAU]	Area%
17.781	BM m	0.5511	970.6228	26.8897	4.0219
22.617	BB	5.5567	23162.7872	523.9229	95.9781

Supplementary Figure 20. HPLC Trace of **3s**

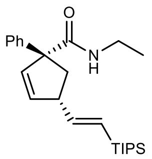

(1R,4S)-N-ethyl-1-phenyl-4-((E)-2-(triisopropylsilyl)vinyl)cyclopent-2-ene-1-carboxamide (3t)

34.6 mg, 87% yield, 86% *ee*, d.r. > 20:1, E/Z > 20:1; R_f = 0.5 (PE/EA = 10/1);Colorless oil. $[\alpha]_D^{20}$ = -118 (*c* = 0.10, MeOH).

^{1}H NMR (300 MHz, $CDCl_3$) δ 7.32 (d, *J* = 4.3 Hz, 4H), 7.26 – 7.22 (m, 1H), 6.09 – 6.03 (m, 1H), 6.02 – 5.99 (m, 1H), 5.91 (dd, *J* = 5.5, 2.0 Hz, 1H), 5.55 (dd, *J* = 18.8, 1.1 Hz, 1H), 5.35 (brs, 1H), 3.39 (q, *J* = 7.5 Hz, 1H), 3.29 – 3.20 (m, *J* = 7.2, 5.6 Hz, 2H), 2.69 (dd, *J* = 13.1, 7.0 Hz, 1H), 2.42 (dd, *J* = 13.1, 7.7 Hz, 1H), 1.15 – 0.89 (m, 24H) ppm.

^{13}C NMR (75 MHz, $CDCl_3$) δ 174.9, 150.6, 143.9, 137.4, 133.4, 128.6, 127.0, 126.6, 123.7, 65.0, 52.4, 42.9, 34.8, 18.6, 14.7, 10.8 ppm.

HRMS (ESI) calcd for $[C_{25}H_{39}NOSi + H]^+$ 398.2874, found 398.2880.

HPLC: Daicel Chiralcel IA-3, *n*-hexane/isopropanol 99.5/0.5, flow rate = 1.0 mL/min, uv-vis λ = 210 nm, t_{R1} = 25.5 min (major), t_{R2} = 34.3 min (minor).

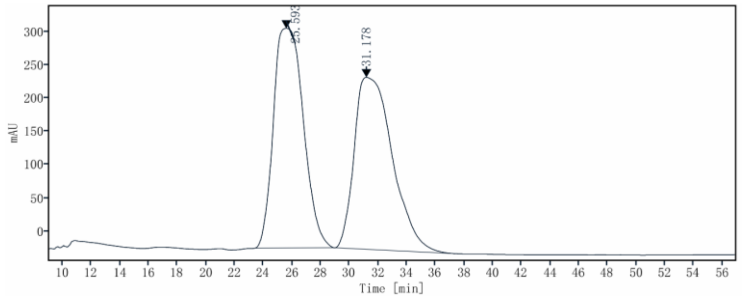

RetTime[min]	Type	Width[min]	Area[mAU*s]	Height[mAU]	Area%
25.593	MM m	2.2897	48049.3504	330.0075	50.0481
31.178	MM m	2.8956	47957.0813	258.3534	49.9519

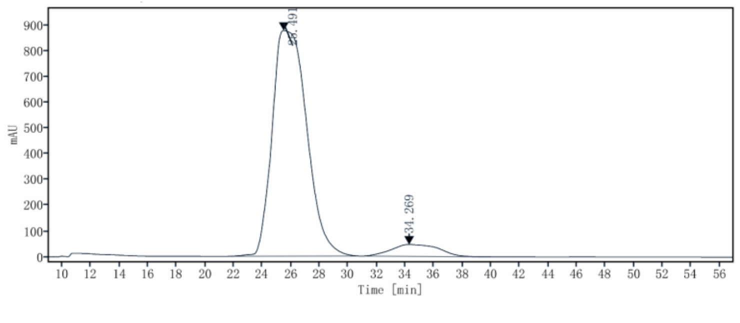

RetTime[min]	Type	Width[min]	Area[mAU*s]	Height[mAU]	Area%
25.491	BB	9.0867	147566.4004	876.0637	93.2240
34.269	BB	9.0567	10725.9511	46.2585	6.7760

Supplementary Figure 21. HPLC Trace of **3t**

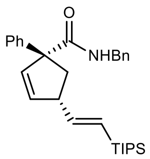

(1R,4S)-N-benzyl-1-phenyl-4-((E)-2-(triisopropylsilyl)vinyl)cyclopent-2-ene-1-carboxamide (3u)

43.2 mg, 94% yield, 86% *ee*, d.r. > 20:1, E/Z > 20:1; R_f = 0.5 (PE/EA = 10/1); White solid, m.p. 103 – 105 ^oC; $[\alpha]_D^{20}$ = -66 (*c* = 0.18, MeOH).

^{1}H NMR (300 MHz, $CDCl_3$) δ 7.35 – 7.17 (m, 8H), 7.14 – 7.10 (m, 2H), 6.11 – 6.05 (m, 2H), 5.92 (dd, *J* = 5.6, 2.0 Hz, 1H), 5.66 (t, *J* = 5.8 Hz, 1H), 5.56 (dd, *J* = 18.7, 1.1 Hz, 1H), 4.39 (dd, *J* = 5.8, 1.7 Hz, 2H), 3.45 – 3.36 (m, 1H), 2.75 (dd, *J* = 13.2, 7.1 Hz, 1H), 2.45 (dd, *J* = 13.1, 7.7 Hz, 1H), 1.15 – 0.94 (m, 21H) ppm.

^{13}C NMR (75 MHz, $CDCl_3$) δ 174.9, 150.5, 143.8, 138.4, 137.6, 133.2, 128.7, 128.6, 127.5, 127.4, 127.1, 126.6, 123.8, 65.2, 52.4, 43.9, 42.9, 18.7, 10.9 ppm.

HRMS (ESI) calcd for $[C_{30}H_{41}NOSi+H]^+$ 460.3030, found 460.3035.

HPLC: Daicel Chiralcel OD-H, *n*-hexane/isopropanol 95/5, flow rate = 0.5 mL/min, uv-vis λ = 250 nm, t_{R1} = 13.9 min (major), t_{R2} = 17.2 min (minor).

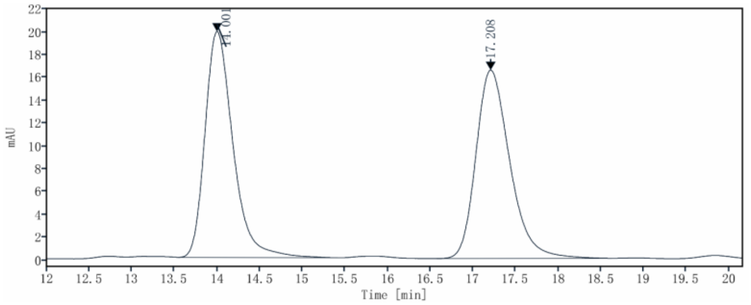

RetTime[min]	Type	Width[min]	Area[mAU*s]	Height[mAU]	Area%
14.001	BB	1.8667	452.6461	19.8113	49.8365
17.208	BB	2.0483	455.6166	16.5167	50.1635

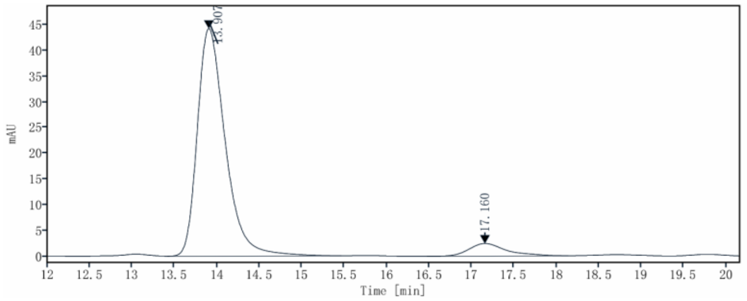

RetTime[min]	Type	Width[min]	Area[mAU*s]	Height[mAU]	Area%
13.907	BB	2.0483	1007.0245	44.0459	93.1778
17.160	BB	1.6617	73.7310	2.4365	6.8222

Supplementary Figure 22. HPLC Trace of **3u**

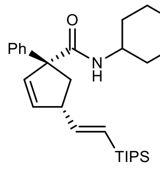

(1R,4S)-N-cyclohexyl-1-phenyl-4-((E)-2-(triisopropylsilyl)vinyl)cyclopent-2-ene-1-carboxamide (3v)

39.8 mg, 88% yield, 91% *ee*, d.r. > 20:1, E/Z > 20:1; R_f = 0.7 (PE/EA = 10/1); Colorless oil. $[\alpha]_D^{20}$ = -98 (*c* = 0.13, MeOH).

^{1}H NMR (300 MHz, $CDCl_3$) δ 7.31 (d, *J* = 4.3 Hz, 4H), 7.26 – 7.20 (m, 1H), 6.07 – 5.99 (m, 2H), 5.90 (dd, *J* = 5.5, 2.0 Hz, 1H), 5.55 (dd, *J* = 18.7, 1.0 Hz, 1H), 5.18 (d, *J* = 8.2 Hz, 1H), 3.75 (m, 1H), 3.40 (q, *J* = 7.6 Hz, 1H), 2.68 (dd, *J* = 13.2, 7.0 Hz, 1H), 2.41 (dd, *J* = 13.2, 7.8 Hz, 1H), 1.86 – 1.80 (m, 2H), 1.65 – 1.51 (m, 3H), 1.38 – 1.22 (m, 2H), 1.15 – 0.92 (m, 24H) ppm.

^{13}C NMR (75 MHz, $CDCl_3$) δ 174.1, 150.7, 144.1, 137.3, 133.5, 128.6, 126.9, 126.6, 123.7, 65.2, 52.5, 48.4, 42.9, 32.8, 32.8, 25.5, 24.8, 24.7, 18.7, 10.8 ppm.

HRMS (ESI) calcd for $[C_{29}H_{45}NOSi+H]^+$ 452.3343, found 452.3349.

HPLC: Daicel Chiralcel OD-H, *n*-hexane/isopropanol 95/5, flow rate = 0.5 mL/min, uv-vis λ = 250 nm, t_{R1} = 7.4 min (major), t_{R2} = 14.1 min (minor).

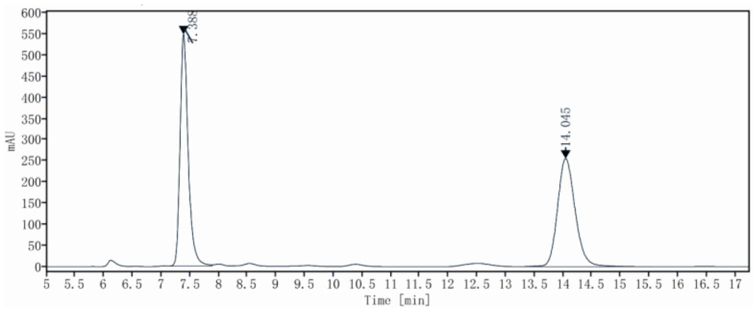

RetTime[min]	Type	Width[min]	Area[mAU*s]	Height[mAU]	Area%
7.388	BV	0.7560	5422.3802	549.3881	49.7957
14.045	BB	2.3200	5466.8726	256.0829	50.2043

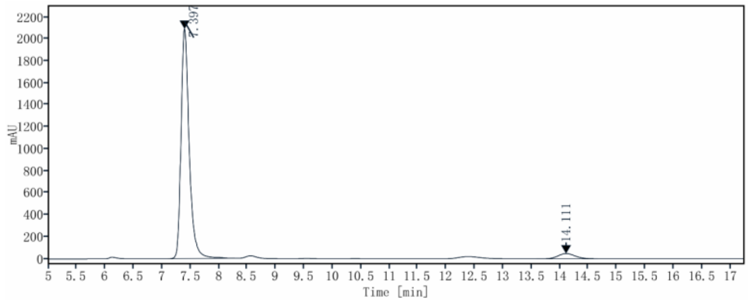

RetTime[min]	Type	Width[min]	Area[mAU*s]	Height[mAU]	Area%
7.397	BB	1.1694	20956.3386	2090.8895	95.4606
14.111	MM m	0.3225	996.5279	47.9391	4.5394

Supplementary Figure 23. HPLC Trace of **3v**

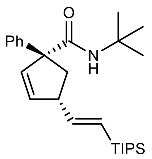

(1R,4S)-N-(tert-butyl)-1-phenyl-4-((E)-2-(triisopropylsilyl)vinyl)cyclopent-2-ene-1-carboxamide (3w)

34.1 mg, 80% yield, 94% *ee*, d.r. > 20:1, E/Z > 20:1; R_f = 0.7 (PE/EA = 10/1); Colorless oil. $[\alpha]_D^{20}$ = -114 (*c* = 0.11, MeOH).

^{1}H NMR (300 MHz, $CDCl_3$) δ 7.31 (d, *J* = 4.2 Hz, 4H), 7.26 – 7.21 (m, 1H), 6.07 – 5.99 (m, 1H), 5.99 – 5.97 (m, 1H), 5.89 (dd, *J* = 5.5, 2.0 Hz, 1H), 5.55 (dd, *J* = 18.8, 1.0 Hz, 1H), 5.10 (brs, 1H), 3.38 (q, *J* = 7.6 Hz, 1H), 2.67 (dd, *J* = 13.2, 7.1 Hz, 1H), 2.37 (dd, *J* = 13.1, 7.7 Hz, 1H), 1.28 (s, 9H), 1.12 – 0.96 (m, 21H) ppm.

^{13}C NMR (75 MHz, $CDCl_3$) δ 174.3, 150.7, 144.2, 137.3, 133.6, 128.6, 126.9, 126.5, 123.7, 65.7, 52.5, 51.2, 42.9, 28.6, 18.7, 10.8 ppm.

HRMS (ESI) calcd for $[C_{27}H_{43}NOSi+H]^+$ 426.3187, found 426.3194.

HPLC: Daicel Chiralcel IC-3, *n*-hexane/isopropanol 99.5/0.5, flow rate = 0.5 mL/min, uv-vis λ = 210 nm, t_{R1} = 18.0 min (minor), t_{R2} = 20.1 min (major).

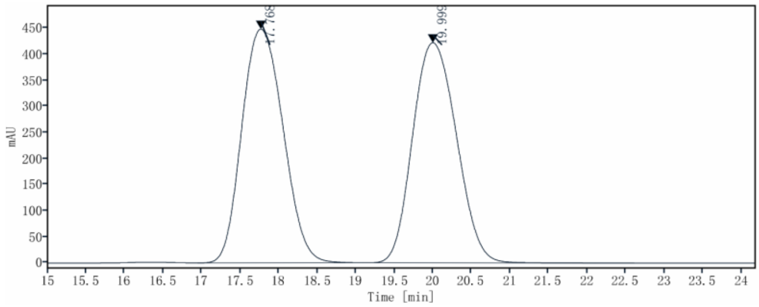

RetTime[min]	Type	Width[min]	Area[mAU*s]	Height[mAU]	Area%
17.768	BB	2.1979	16708.3588	448.1787	49.9933
19.999	BB	3.4633	16712.8309	422.1321	50.0067

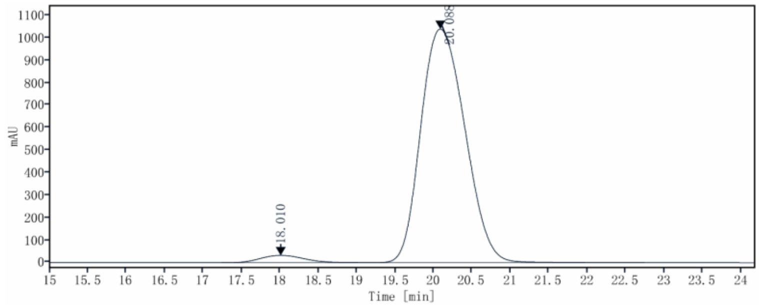

RetTime[min]	Type	Width[min]	Area[mAU*s]	Height[mAU]	Area%
18.010	BV	1.9802	1277.2269	32.2799	2.9815
20.088	VB	3.5231	41560.6856	1039.6830	97.0185

Supplementary Figure 24. HPLC Trace of **3w**

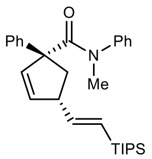

(1R,4S)-N-methyl-N,1-diphenyl-4-((E)-2-(triisopropylsilyl)vinyl)cyclopent-2-ene-1-carboxamide (3x)

29.0 mg, 63% yield, 96% *ee*, d.r. > 20:1, E/Z > 20:1; R_f = 0.7 (PE/EA = 10/1); Colorless oil. $[\alpha]_D^{20}$ = -145 (*c* = 0.13, MeOH).

^{1}H NMR (300 MHz, CDCl3) δ 7.21 – 7.05 (m, 8H), 6.65 (s, 2H), 6.01 (dd, *J* = 18.8, 7.4 Hz, 1H), 5.81 (s, 1H), 5.60 (d, *J* = 5.6 Hz, 1H), 5.52 (d, *J* = 18.7 Hz, 1H), 3.29 (d, *J* = 10.3 Hz, 1H), 3.18 (s, 3H), 2.50 (dd, *J* = 13.0, 8.0 Hz, 1H), 2.23 (s, 1H), 1.14 – 0.95 (m, 21H) ppm.

^{13}C NMR (75 MHz, CDCl3) δ 174.4, 151.0, 144.1, 135.3, 133.9, 128.7, 128.6, 127.3, 126.5, 125.1, 123.5, 65.4, 52.0, 43.6, 39.9, 18.7, 10.9 ppm.

HRMS (ESI) calcd for $[C_{30}H_{41}NOSi+H]^+$ 460.3030, found 460.3042.

HPLC: Daicel Chiralcel IA-3, *n*-hexane/isopropanol 98/2, flow rate = 0.5 mL/min, uv-vis λ = 250 nm, t_{R1} = 10.7 min (minor), t_{R2} = 13.5 min (major).

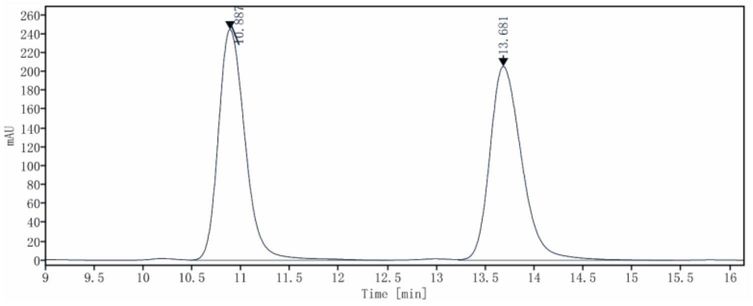

RetTime[min]	Type	Width[min]	Area[mAU*s]	Height[mAU]	Area%
10.887	VB	2.0185	4648.6250	244.5999	49.9689
13.681	VB	2.1933	4654.4092	205.3556	50.0311

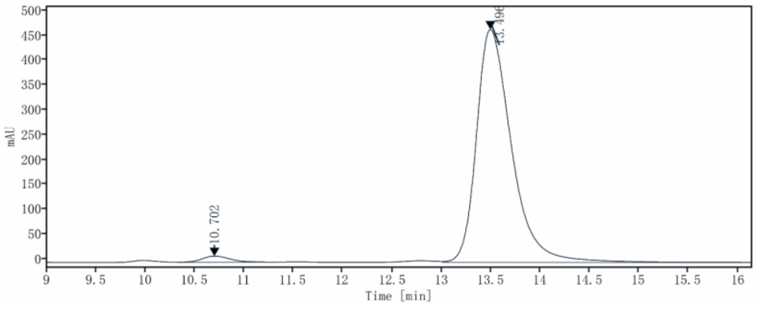

RetTime[min]	Type	Width[min]	Area[mAU*s]	Height[mAU]	Area%
10.702	VB	1.0069	246.3761	12.2677	2.0764
13.496	VB	3.3122	11619.2764	468.8769	97.9236

Supplementary Figure 25. HPLC Trace of **3x**

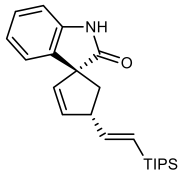

(1R,4S)-4-((E)-2-(triisopropylsilyl)vinyl)spiro[cyclopentane-1,3'-indolin]-2-en-2'-one (3y)

28.7 mg, 78% yield, 90% *ee*, d.r. > 20:1, E/Z > 20:1; R_f = 0.5 (PE/EA = 5/1); Colorless oil. $[\alpha]_D^{20}$ = -125 (*c* = 0.20, MeOH).

^{1}H NMR (300 MHz, $CDCl_3$) δ 9.43 (s, 1H), 7.19 (td, *J* = 7.6, 1.4 Hz, 1H), 7.11 (dd, *J* = 7.5, 1.3 Hz, 1H), 7.00 (td, *J* = 7.5, 1.1 Hz, 1H), 6.93 (d, *J* = 7.6 Hz, 1H), 6.17 (dd, *J* = 18.7, 8.0 Hz, 1H), 6.06 (dd, *J* = 5.3, 2.0 Hz, 1H), 5.67 (dd, *J* = 18.7, 0.9 Hz, 1H), 5.56 (dd, *J* = 5.4, 2.3 Hz, 1H), 3.87 – 3.78 (m, 1H), 2.38 (dd, *J* = 13.1, 7.7 Hz, 1H), 2.29 (dd, *J* = 13.1, 7.3 Hz, 1H), 1.12 – 0.98 (m, 21H) ppm.

^{13}C NMR (75 MHz, $CDCl_3$) δ 182.8, 151.0, 140.2, 139.1, 134.2, 131.6, 128.1, 124.5, 123.3, 122.7, 110.1, 61.9, 54.1, 42.6, 18.7, 10.9 ppm.

HRMS (ESI) calcd for $[C_{23}H_{33}NOSi+Na]^+$ 390.2224, found 390.2219.

HPLC: Daicel Chiralcel OD-H, *n*-hexane/isopropanol 95/5, flow rate = 0.5 mL/min, uv-vis λ = 250 nm, t_{R1} = 8.6 min (minor), t_{R2} = 9.7 min (major).

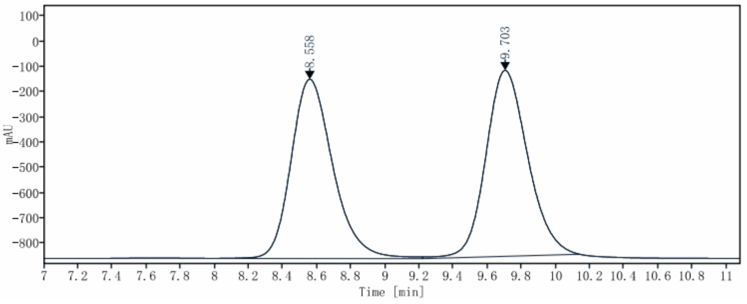

RetTime[min]	Type	Width[min]	Area[mAU*s]	Height[mAU]	Area%
8.558	VM m	0.2625	12165.7688	711.5964	49.1408
9.703	MM m	0.2631	12591.1975	737.7794	50.8592

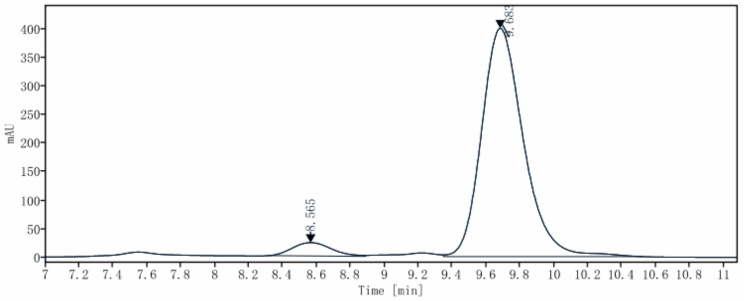

RetTime[min]	Type	Width[min]	Area[mAU*s]	Height[mAU]	Area%
8.565	MV m	0.2545	375.2083	23.3457	5.1925
9.683	VM m	0.2628	6850.7711	400.0938	94.8075

Supplementary Figure 26. HPLC Trace of **3y**

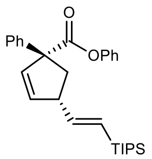

phenyl (1R,4S)-1-phenyl-4-((E)-2-(triisopropylsilyl)vinyl)cyclopent-2-ene-1-carboxylate (3z)

32.2 mg, 72% yield, 90% *ee*, d.r. > 20:1, E/Z > 20:1; R_f = 0.6 (PE/EA = 50/1); Colorless oil. $[\alpha]_D^{20}$ = -49 (*c* = 0.14, MeOH).

^{1}H NMR (300 MHz, $CDCl_3$) δ 7.42 – 7.25 (m, 7H), 7.21 – 7.15 (m, 1H), 6.99 – 6.94 (m, 2H), 6.31 (dd, *J* = 5.6, 2.3 Hz, 1H), 6.10 – 6.03 (m, 1H), 6.02 – 6.00 (m, 1H), 5.58 (dd, *J* = 18.7, 1.1 Hz, 1H), 3.51 – 3.42 (m, 1H), 2.78 (dd, *J* = 13.4, 6.3 Hz, 1H), 2.47 (dd, *J* = 13.4, 8.0 Hz, 1H), 1.12 – 0.97 (m, 21H) ppm.

^{13}C NMR (75 MHz, $CDCl_3$) δ 173.3, 151.0, 150.2, 143.0, 137.5, 132.4, 129.4, 128.8, 127.2, 126.0, 125.8, 123.9, 121.4, 64.7, 52.0, 42.8, 18.7, 10.9 ppm.

HRMS (ESI) calcd for $[C_{29}H_{38}O_2Si+H]^+$ 447.2714, found 447.2715.

HPLC: Daicel Chiralcel OJ-H, *n*-hexane/isopropanol 98/2, flow rate = 0.5 mL/min, uv-vis λ = 210 nm, t_{R1} = 8.8 min (major), t_{R2} = 11.5 min (minor).

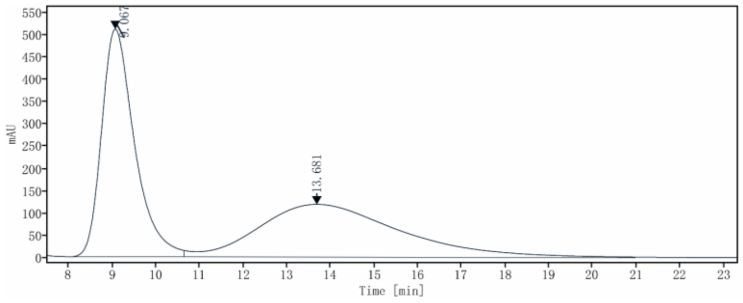

RetTime[min]	Type	Width[min]	Area[mAU*s]	Height[mAU]	Area%
9.067	BM m	0.7921	26736.7713	509.6414	50.1529
13.681	MM m	3.2797	26573.7598	117.8956	49.8471

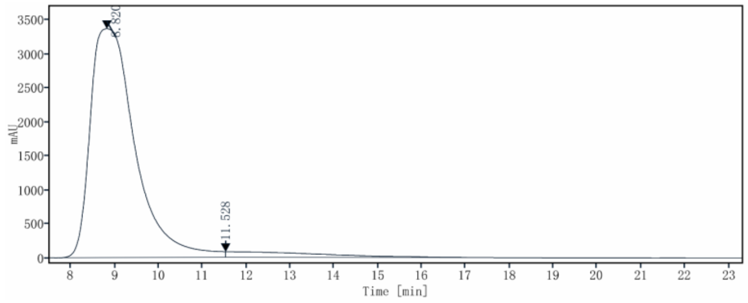

RetTime[min]	Type	Width[min]	Area[mAU*s]	Height[mAU]	Area%
8.820	MM m	1.0801	239113.4410	3368.6682	95.1925
11.528	MM m	2.5032	12075.9415	80.4022	4.8075

Supplementary Figure 27. HPLC Trace of **3z**

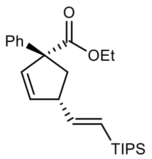

ethyl (1R,4S)-1-phenyl-4-((E)-2-(triisopropylsilyl)vinyl)cyclopent-2-ene-1-carboxylate (3aa)

32.7 mg, 82% yield, 92% *ee*, d.r. > 20:1, E/Z > 20:1; R_f = 0.6 (PE/EA = 50/1); Colorless oil. $[\alpha]_D^{20}$ = -17 (*c* = 0.19, MeOH).

^{1}H NMR (300 MHz, $CDCl_3$) δ 7.34 – 7.29 (m, 4H), 7.25 – 7.19 (m, 1H), 6.17 (dd, *J* = 5.5, 2.3 Hz, 1H), 6.00 (dd, *J* = 18.7, 7.3 Hz, 1H), 5.90 (dd, *J* = 5.6, 2.1 Hz, 1H), 5.53 (dd, *J* = 18.8, 1.1 Hz, 1H), 4.22 – 4.06 (m, 2H), 3.43 – 3.34 (m, 1H), 2.65 (dd, *J* = 13.4, 6.4 Hz, 1H), 2.38 (dd, *J* = 13.3, 7.9 Hz, 1H), 1.19 (t, *J* = 7.1 Hz, 3H), 1.11 – 0.94 (m, 21H) ppm.

^{13}C NMR (75 MHz, $CDCl_3$) δ 174.8, 150.5, 143.5, 136.5, 133.1, 128.5, 126.8, 125.9, 123.5, 64.4, 61.1, 51.9, 42.9, 18.6, 14.1, 10.8 ppm.

HRMS (ESI) calcd for $[C_{25}H_{38}O_2Si+H]^+$ 399.2714, found 399.2712.

HPLC: Daicel Chiralcel OJ-H, *n*-hexane/isopropanol 98/2, flow rate = 0.5 mL/min, uv-vis λ = 210 nm, t_{R1} = 6.6 min (minor), t_{R2} = 8.8 min (major).

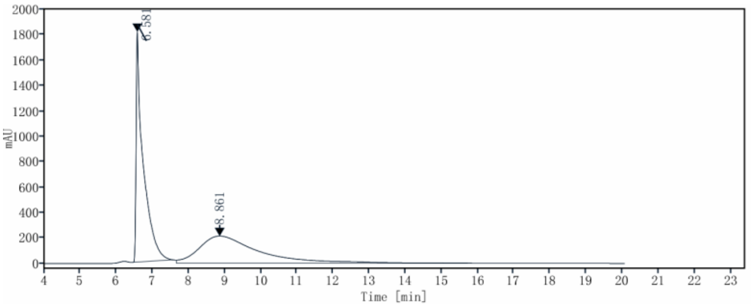

RetTime[min]	Type	Width[min]	Area[mAU*s]	Height[mAU]	Area%
6.581	MM m	0.1773	24932.2946	1814.2428	50.3655
8.861	MM m	1.6657	24570.4379	213.6513	49.6345

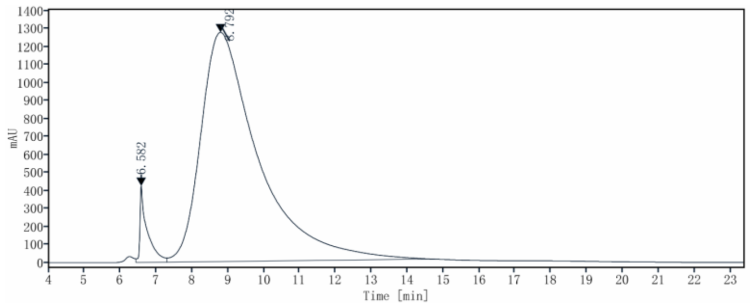

RetTime[min]	Type	Width[min]	Area[mAU*s]	Height[mAU]	Area%
6.582	VM m	0.1831	6071.2374	426.3982	4.0897
8.792	MM m	1.6205	142379.2279	1276.2411	95.9103

Supplementary Figure 28. HPLC Trace of **3aa**

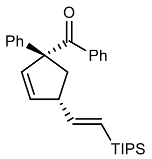

phenyl((1R,4S)-1-phenyl-4-((E)-2-(triisopropylsilyl)vinyl)cyclopent-2-en-1-yl)methanone (3ab)

38.8 mg, 90% yield, 92% *ee*, d.r. > 20:1, E/Z > 20:1; R_f = 0.6 (PE/EA = 20/1); Colorless oil. $[\alpha]_D^{20}$ = -5 (*c* = 0.13, MeOH).

^{1}H NMR (300 MHz, $CDCl_3$) δ 7.68 – 7.63 (m, 2H), 7.42 – 7.32 (m, 2H), 7.31 – 7.21 (m, 6H), 6.17 (dd, *J* = 5.6, 2.3 Hz, 1H), 5.99 (dd, *J* = 18.8, 7.4 Hz, 1H), 5.88 (dd, *J* = 5.6, 2.1 Hz, 1H), 5.53 (dd, *J* = 18.8, 1.1 Hz, 1H), 3.51 (q, *J* = 7.4 Hz, 1H), 2.82 (dd, *J* = 13.4, 6.4 Hz, 1H), 2.41 (dd, *J* = 13.3, 7.9 Hz, 1H), 1.14 – 0.93 (m, 21H) ppm.

^{13}C NMR (75 MHz, $CDCl_3$) δ 200.1, 150.5, 144.1, 136.9, 136.3, 133.9, 132.1, 130.0, 129.2, 128.1, 126.9, 125.6, 123.6, 69.7, 52.0, 42.9, 18.7, 10.9 ppm.

HRMS (ESI) calcd for $[C_{29}H_{38}OSi+H]^+$ 431.2765, found 431.2764.

HPLC: Daicel Chiralcel AD-H, *n*-hexane/isopropanol 98/2, flow rate = 0.5 mL/min, uv-vis λ = 250 nm, t_{R1} = 6.6 min (major), t_{R2} = 10.5 min (minor).

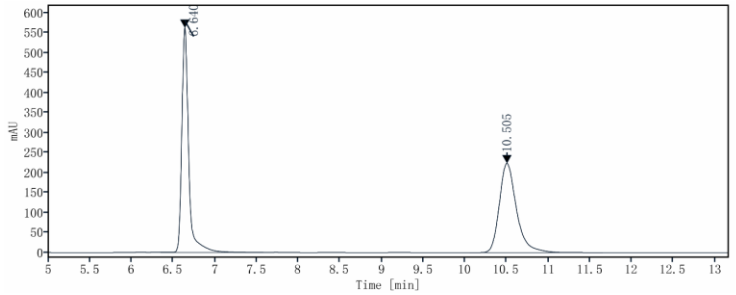

RetTime[min]	Type	Width[min]	Area[mAU*s]	Height[mAU]	Area%
6.640	VB	1.2285	3226.3321	562.8497	50.2330
10.505	BB	1.5900	3196.3987	223.5664	49.7670

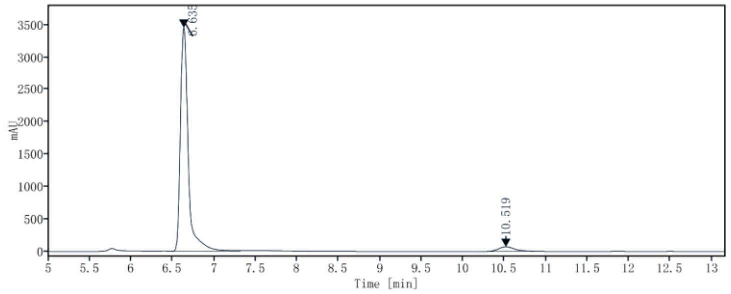

RetTime[min]	Type	Width[min]	Area[mAU*s]	Height[mAU]	Area%
6.635	BV	0.8251	22741.8028	3453.3872	95.9912
10.519	BM m	0.2074	949.7367	70.8492	4.0088

Supplementary Figure 29. HPLC Trace of **3ab**

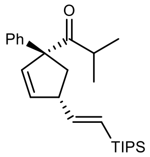

2-methyl-1-((1R,4S)-1-phenyl-4-((E)-2-(triisopropylsilyl)vinyl)cyclopent-2-en-1-yl)propan-1-one (3ac)

31.7 mg, 80% yield, 93% *ee*, d.r. > 20:1, E/Z > 20:1; R_f = 0.6 (PE/EA = 20/1); Colorless oil. $[\alpha]_D^{20}$ = -38 (*c* = 0.14, MeOH).

^{1}H NMR (300 MHz, $CDCl_3$) δ 7.35 – 7.28 (m, 2H), 7.26 – 7.19 (m, 3H), 6.23 (dd, *J* = 5.6, 2.2 Hz, 1H), 6.00 – 5.95 (m, 1H), 5.94 – 5.90 (m, 1H), 5.50 (dd, *J* = 18.8, 1.2 Hz, 1H), 3.41 – 3.34 (m, 1H), 2.85 – 2.74 (m, 2H), 2.15 (dd, *J* = 13.2, 8.3 Hz, 1H), 1.10- 1.02 (m, 21H), 0.99 (d, *J* = 6.8 Hz, 3H), 0.83 (d, *J* = 6.6 Hz, 3H) ppm.

^{13}C NMR (75 MHz, $CDCl_3$) δ 150.4, 142.6, 137.9, 131.2, 128.7, 127.0, 126.7, 123.2, 71.6, 52.0, 40.2, 37.4, 20.8, 20.5, 18.6, 10.8 ppm.

HRMS (ESI) calcd for $[C_{26}H_{40}OSi+H]^+$ 397.2921, found 397.2915.

HPLC: Daicel Chiralcel OJ-H, *n*-hexane/isopropanol 99.5/0.5, flow rate = 0.5 mL/min, uv-vis λ = 210 nm, t_{R1} = 6.8 min (minor), t_{R2} = 8.0 min (major).

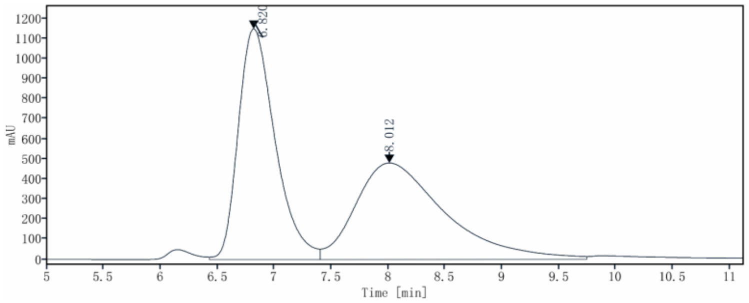

RetTime[min]	Type	Width[min]	Area[mAU*s]	Height[mAU]	Area%
6.820	VV	0.9737	25826.0170	1147.9798	49.6865
8.012	VV	2.3431	26151.9623	480.1116	50.3135

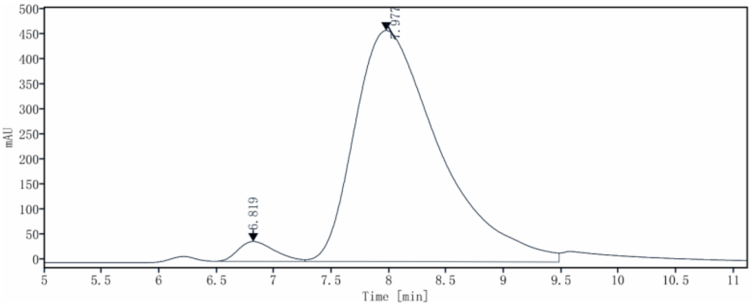

RetTime[min]	Type	Width[min]	Area[mAU*s]	Height[mAU]	Area%
6.819	BV	0.7871	874.6902	39.5201	3.5652
7.977	VV	2.2113	23659.1248	461.6208	96.4348

Supplementary Figure 30. HPLC Trace of **3ac**

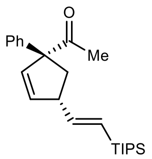

1-((1R,4S)-1-phenyl-4-((E)-2-(triisopropylsilyl)vinyl)cyclopent-2-en-1-yl)ethan-1-one (3ad)

34.3 mg, 93% yield, 90% *ee*, d.r. > 20:1, E/Z > 20:1; R_f = 0.6 (PE/EA = 20/1); Colorless oil. $[\alpha]_D^{20}$ = -50 (*c* = 0.08, MeOH).

^{1}H NMR (300 MHz, $CDCl_3$) δ 7.36 – 7.30 (m, 2H), 7.27 – 7.20 (m, 3H), 6.17 (dd, *J* = 5.6, 2.2 Hz, 1H), 6.01 – 5.92 (m, 2H), 5.51 (dd, *J* = 18.7, 1.1 Hz, 1H), 3.40 (q, *J* = 7.1 Hz, 1H), 2.72 (dd, *J* = 13.2, 5.9 Hz, 1H), 2.21 (dd, *J* = 13.2, 8.2 Hz, 1H), 2.03 (s, 3H), 1.14 – 0.93 (m, 21H) ppm.

^{13}C NMR (75 MHz, $CDCl_3$) δ 207.3, 150.4, 143.0, 137.7, 131.8, 128.9, 127.0, 126.2, 123.4, 71.5, 52.0, 40.4, 26.7, 18.6, 10.9 ppm.

HRMS (ESI) calcd for $[C_{24}H_{36}OSi+H]^+$ 369.2608, found 369.2612.

HPLC: Daicel Chiralcel OD-H, *n*-hexane/isopropanol 99.5/0.5, flow rate = 0.5 mL/min, uv-vis λ = 210 nm, t_{R1} = 8.0 min (minor), t_{R2} = 9.0 min (major).

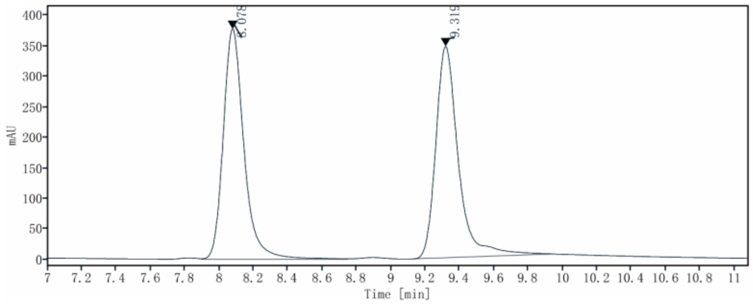

RetTime[min]	Type	Width[min]	Area[mAU*s]	Height[mAU]	Area%
8.078	VV	0.8515	3154.3094	377.4180	49.9602
9.319	BM m	0.1374	3159.3289	346.5573	50.0398

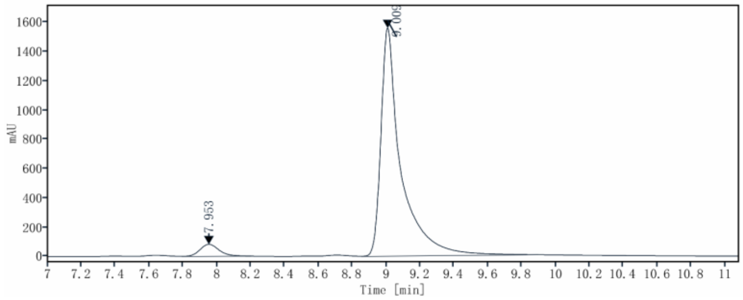

RetTime[min]	Type	Width[min]	Area[mAU*s]	Height[mAU]	Area%
7.953	VM m	0.1209	648.2515	82.2638	4.8156
9.009	VM m	0.1159	12813.3369	1559.2662	95.1844

Supplementary Figure 31. HPLC Trace of **3ad**

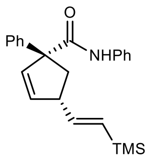

(1R,4S)-N,1-diphenyl-4-((E)-2-(trimethylsilyl)vinyl)cyclopent-2-ene-1-carboxamide (3af)

33.3 mg, 92% yield, 93% *ee*, d.r. > 20:1, E/Z > 20:1; R_f = 0.7 (PE/EA = 10/1); Colorless oil. $[\alpha]_D^{20}$ = -15 (c = 0.10, MeOH).

^{1}H NMR (300 MHz, $CDCl_3$) δ 7.58 – 7.47 (m, 6H), 7.47 – 7.39 (m, 3H), 7.25 – 7.19 (m, 2H), 6.30 (dd, J = 5.6, 2.3 Hz, 1H), 6.25 – 6.17 (m, 1H), 6.16 – 6.14 (m, 1H), 5.86 (dd, J = 18.5, 1.1 Hz, 1H), 3.65 – 3.56 (m, 1H), 2.96 (dd, J = 13.3, 6.7 Hz, 1H), 2.64 (dd, J = 13.2, 7.9 Hz, 1H), 0.20 (s, 9H) ppm.

^{13}C NMR (75 MHz, $CDCl_3$) δ 173.1, 148.0, 143.3, 137.9, 137.8, 132.8, 130.1, 128.9, 128.9, 127.3, 126.6, 124.2, 119.7, 66.0, 51.6, 42.4, -1.2 ppm.

HRMS (ESI) calcd for $[C_{23}H_{27}NOSi+H]^+$ 362.1935, found 362.1940.

HPLC: Daicel Chiralcel OD-H, *n*-hexane/isopropanol 98/2, flow rate = 0.5 mL/min, uv-vis λ = 250 nm, t_{R1} = 14.4 min (major), t_{R2} = 18.1 min (minor).

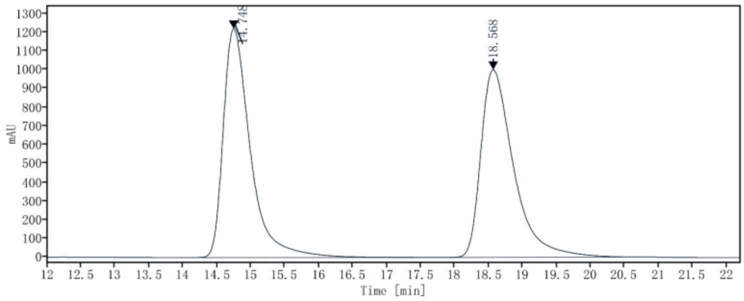

RetTime[min]	Type	Width[min]	Area[mAU*s]	Height[mAU]	Area%
14.748	BB	3.4275	33777.7652	1218.3216	50.2775
18.568	VB	2.7769	33404.8871	999.0106	49.7225

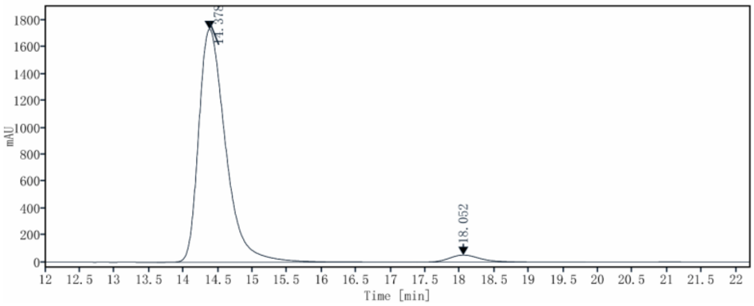

RetTime[min]	Type	Width[min]	Area[mAU*s]	Height[mAU]	Area%
14.378	BB	3.7617	46957.6886	1733.7942	96.5106
18.052	BB	2.4400	1697.7909	52.8376	3.4894

Supplementary Figure 32. HPLC Trace of **3af**

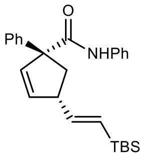

(1R,4S)-4-((E)-2-(tert-butyldimethylsilyl)vinyl)-N,1-diphenylcyclopent-2-ene-1-carboxamide (3ag)

39.1 mg, 97% yield, 94% *ee*, d.r. > 20:1, E/Z > 20:1; R_f = 0.7 (PE/EA = 10/1); Colorless oil. $[\alpha]_D^{20}$ = -50 (*c* = 0.10, MeOH).

^{1}H NMR (300 MHz, $CDCl_3$) δ 7.62 – 7.52 (m, 6H), 7.50 – 7.42 (m, 3H), 7.28 – 7.23 (m, 2H), 6.34 (dd, *J* = 5.6, 2.3 Hz, 1H), 6.25 (dd, *J* = 18.5, 7.3 Hz, 1H), 6.18 (dd, *J* = 5.6, 2.1 Hz, 1H), 5.89 (dd, *J* = 18.5, 1.1 Hz, 1H), 3.65 (q, *J* = 7.3 Hz, 1H), 3.01 (dd, *J* = 13.2, 6.8 Hz, 1H), 2.68 (dd, *J* = 13.2, 7.9 Hz, 1H), 1.05 (s, 9H), 0.20 (s, 6H) ppm.

^{13}C NMR (75 MHz, $CDCl_3$) δ 173.1, 149.4, 143.3, 138.0, 138.0, 132.8, 129.0, 128.9, 127.4, 127.3, 126.7, 124.3, 119.8, 66.1, 51.9, 42.6, 26.5, 16.5, -6.0 ppm.

HRMS (ESI) calcd for $[C_{26}H_{33}NOSi+H]^+$ 404.2404, found 404.2411.

HPLC: Daicel Chiralcel OD-H, *n*-hexane/isopropanol 98/2, flow rate = 0.5 mL/min, uv-vis λ = 250 nm, t_{R1} = 12.5 min (major), t_{R2} = 15.9 min (minor).

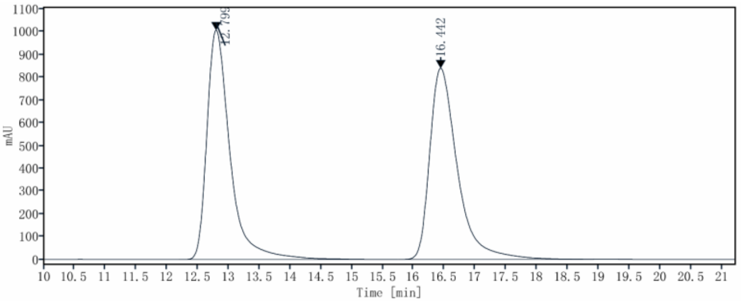

RetTime[min]	Type	Width[min]	Area[mAU*s]	Height[mAU]	Area%
12.799	BB	3.5467	26247.8852	1006.5873	50.1620
16.442	BB	2.9894	26078.3293	839.1065	49.8380

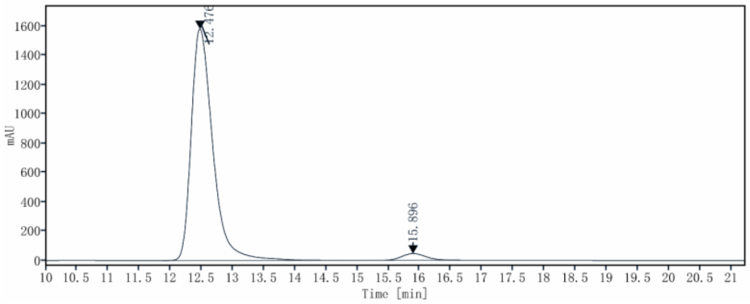

RetTime[min]	Type	Width[min]	Area[mAU*s]	Height[mAU]	Area%
12.476	BB	3.3828	38121.3107	1580.7511	96.7935
15.896	BM m	0.4232	1262.8591	46.2994	3.2065

Supplementary Figure 33. HPLC Trace of **3ag**

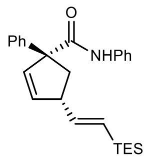

(1R,4S)-N,1-diphenyl-4-((E)-2-(triethylsilyl)vinyl)cyclopent-2-ene-1-carboxamide (3ah)

37.1 mg, 92% yield, 94% *ee*, d.r. > 20:1, E/Z > 20:1; R_f = 0.7 (PE/EA = 10/1); Colorless oil. $[\alpha]_D^{20}$ = -19 (*c* = 0.10, MeOH).

^{1}H NMR (300 MHz, $CDCl_3$) δ 7.43 – 7.32 (m, 6H), 7.30 – 7.22 (m, 3H), 7.08 – 7.03 (m, 2H), 6.14 (dd, *J* = 5.6, 2.4 Hz, 1H), 6.05 (dd, *J* = 18.7, 7.3 Hz, 1H), 5.99 (dd, *J* = 5.5, 2.1 Hz, 1H), 5.63 (dd, *J* = 18.7, 1.1 Hz, 1H), 3.50 – 3.40 (m, 1H), 2.80 (dd, *J* = 13.2, 6.8 Hz, 1H), 2.48 (dd, J = 13.2, 7.8 Hz, 1H), 0.91 (t, *J* = 7.8 Hz, 9H), 0.58 – 0.50 (m, 6H) ppm.

^{13}C NMR (75 MHz, $CDCl_3$) δ 173.0, 149.3, 143.3, 138.0, 138.0, 132.8, 128.9, 128.9, 127.3, 126.6, 126.2, 124.2, 119.7, 66.0, 51.9, 42.6, 7.3, 3.4 ppm.

HRMS (ESI) calcd for $[C_{26}H_{33}NOSi+H]^+$ 404.2404, found 404.2411.

HPLC: Daicel Chiralcel OD-H, *n*-hexane/isopropanol 98/2, flow rate = 0.5 mL/min, uv-vis λ = 250 nm, t_{R1} = 13.1 min (major), t_{R2} = 16.5 min (minor).

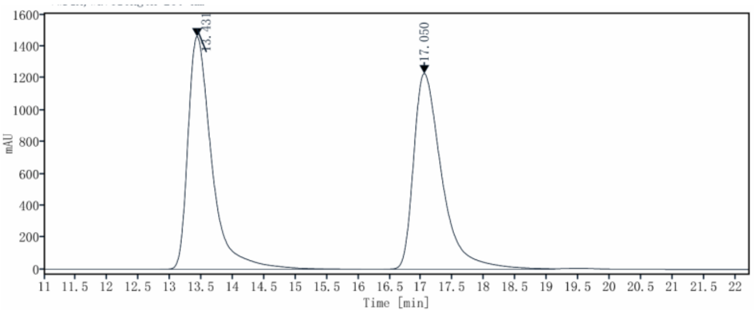

RetTime[min]	Type	Width[min]	Area[mAU*s]	Height[mAU]	Area%
13.431	BB	3.1950	38522.9676	1462.3515	50.1161
17.050	BV	2.7271	38344.5320	1229.7165	49.8839

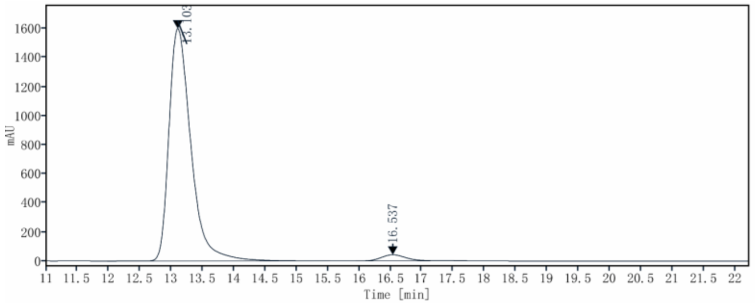

RetTime[min]	Type	Width[min]	Area[mAU*s]	Height[mAU]	Area%
13.103	BB	3.5583	38940.2204	1600.1524	97.0829
16.537	BV	1.1871	1170.0674	42.6405	2.9171

Supplementary Figure 34. HPLC Trace of **3ah**

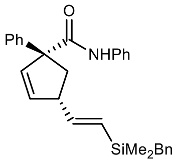

(1R,4S)-4-((E)-2-(benzyldimethylsilyl)vinyl)-N,1-diphenylcyclopent-2-ene-1-carboxamide (3ai)

42.0 mg, 96% yield, 94% *ee*, d.r. > 20:1, E/Z > 20:1; R_f = 0.6 (PE/EA = 10/1); Colorless oil. $[\alpha]_D^{20}$ = -48 (*c* = 0.43, MeOH).

^{1}H NMR (300 MHz, $CDCl_3$) δ 7.59 – 7.50 (m, 6H), 7.48 – 7.34 (m, 5H), 7.26 – 7.20 (m, 3H), 7.18 – 7.14 (m, 2H), 6.32 (dd, *J* = 5.6, 2.3 Hz, 1H), 6.21 (dd, *J* = 18.6, 7.3 Hz, 1H), 6.14 (dd, *J* = 5.5, 2.1 Hz, 1H), 5.85 (dd, *J* = 18.6, 1.1 Hz, 1H), 3.61 (q, *J* = 7.5 Hz, 1H), 2.98 (dd, *J* = 13.3, 6.7 Hz, 1H), 2.64 (dd, *J* = 13.3, 7.9 Hz, 1H), 2.28 (s, 2H), 0.20 (d, *J* = 2.0 Hz, 6H) ppm.

^{13}C NMR (75 MHz, $CDCl_3$) δ 173.1, 149.4, 143.4, 140.1, 138.0, 137.8, 133.0, 129.0, 129.0, 128.4, 128.1, 128.1, 127.5, 126.7, 124.4, 124.0, 119.8, 66.2, 51.7, 42.4, 26.2, -3.2, -3.3 ppm.

HRMS (ESI) calcd for $[C_{29}H_{31}NOSi+H]^+$ 438.2248, found 438.2244.

HPLC: Daicel Chiralcel OD-H, *n*-hexane/isopropanol 95/5, flow rate = 0.5 mL/min, uv-vis λ = 250 nm, t_{R1} = 18.0 min (major), t_{R2} = 21.9 min (minor).

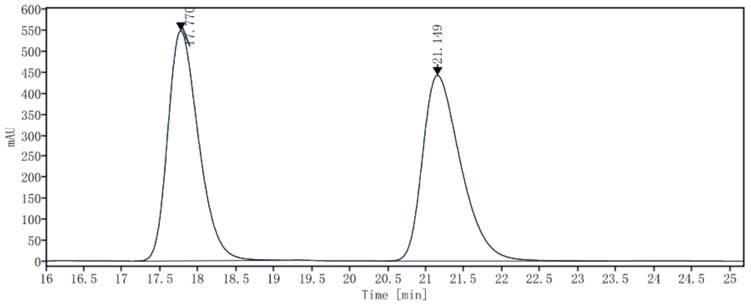

RetTime[min]	Type	Width[min]	Area[mAU*s]	Height[mAU]	Area%
17.770	BB	1.9277	15390.9898	548.4446	49.9061
21.149	BB	2.9267	15448.9283	442.6097	50.0939

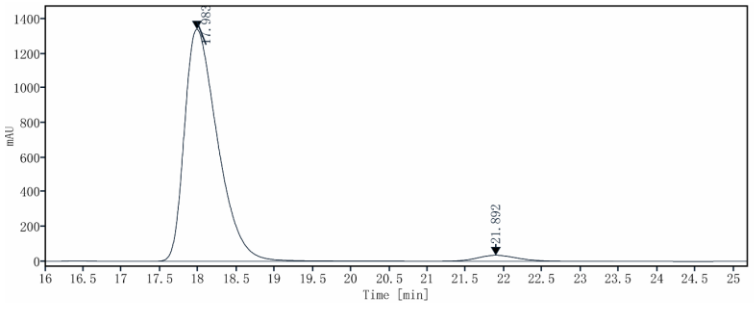

RetTime[min]	Type	Width[min]	Area[mAU*s]	Height[mAU]	Area%
17.983	BB	3.6194	40437.9336	1342.1164	96.9066
21.892	BB	2.4300	1290.8587	34.7610	3.0934

Supplementary Figure 35. HPLC Trace of **3ai**

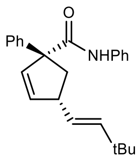

(1R,4R)-4-((E)-3,3-dimethylbut-1-en-1-yl)-N,1-diphenylcyclopent-2-ene-1-carboxamide (3aj)

20.7 mg, 60% yield, 90% *ee*, d.r. > 20:1, E/Z > 20:1; R_f = 0.7 (PE/EA = 10/1); Colorless oil. $[\alpha]_D^{20}$ = -21 (*c* = 0.10, MeOH).

^{1}H NMR (300 MHz, $CDCl_3$) δ 7.42 – 7.31 (m, 6H), 7.30 – 7.22 (m, 3H), 7.08 – 7.02 (m, 2H), 6.10 (dd, *J* = 5.6, 2.3 Hz, 1H), 5.95 (dd, *J* = 5.6, 2.0 Hz, 1H), 5.50 (d, *J* = 15.5 Hz, 1H), 5.35 (dd, *J* = 15.6, 7.8 Hz, 1H), 3.40 – 3.31 (m, 1H), 2.71 (dd, *J* = 13.2, 6.8 Hz, 1H), 2.46 (dd, *J* = 13.2, 7.8 Hz, 1H), 0.98 (s, 9H) ppm.

^{13}C NMR (75 MHz, $CDCl_3$) δ 173.3, 143.4, 141.9, 138.9, 138.0, 132.3, 129.0, 127.3, 126.7, 126.7, 124.3, 119.8, 66.1, 48.2, 43.3, 32.9, 29.7 ppm.

HRMS (ESI) calcd for $[C_{24}H_{27}NO+H]^+$ 346.2165, found 346.2166.

HPLC: Daicel Chiralcel OD-H, *n*-hexane/isopropanol 98/2, flow rate = 0.5 mL/min, uv-vis λ = 250 nm, t_{R1} = 15.6 min (major), t_{R2} = 19.4 min (minor).

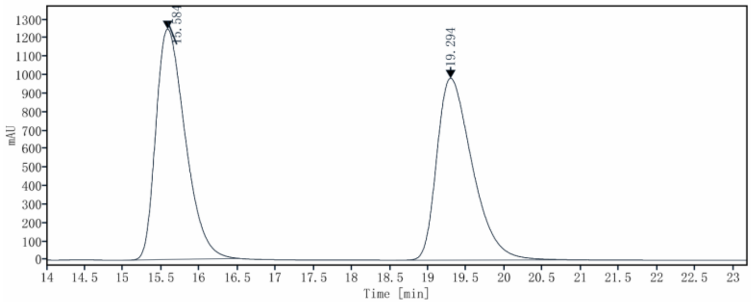

RetTime[min]	Type	Width[min]	Area[mAU*s]	Height[mAU]	Area%
15.584	BM m	0.4154	33413.4129	1247.6981	51.2059
19.294	MM m	0.4983	31839.6816	984.6536	48.7941

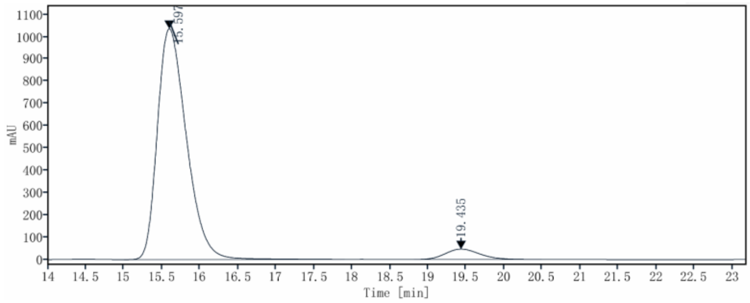

RetTime[min]	Type	Width[min]	Area[mAU*s]	Height[mAU]	Area%
15.597	BB	2.8950	27471.0846	1036.0464	94.9197
19.435	MM m	0.4876	1470.2984	46.6616	5.0803

Supplementary Figure 36. HPLC Trace of **3aj**

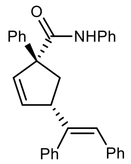

(1R,4S)-4-((Z)-1,2-diphenylvinyl)-N,1-diphenylcyclopent-2-ene-1-carboxamide (3am)

29.2 mg, 66% yield, 47% *ee*, d.r. > 20:1, Z/E > 20:1; R_f = 0.4 (PE/EA = 10/1); Colorless oil.

^{1}H NMR (300 MHz, $CDCl_3$) δ 7.48 – 7.30 (m, 12H), 7.28 – 7.24 (m, 2H), 7.18 – 7.09 (m, 5H), 6.99 – 6.93 (m, 2H), 6.58 (s, 1H), 6.34 (qd, *J* = 5.6, 1.8 Hz, 2H), 3.95 (tq, *J* = 7.8, 1.6 Hz, 1H), 2.98 (dd, *J* = 13.2, 7.6 Hz, 1H), 2.62 (dd, *J* = 13.2, 7.9 Hz, 1H) ppm.

^{13}C NMR (75 MHz, $CDCl_3$) δ 172.9, 144.4, 143.0, 140.9, 138.0, 137.3, 137.1, 133.9, 129.2, 129.0, 128.8, 127.9, 127.4, 127.2, 126.8, 126.5, 124.3, 119.9, 65.7, 53.7, 42.7 ppm.

HRMS (ESI) calcd for $[C_{32}H_{27}NO+H]^+$ 442.2165, found 442.2168.

HPLC: Daicel Chiralcel OD-H, n-hexane/isopropanol 98/2, flow rate = 0.5 mL/min, uv-vis λ = 250 nm, t_{R1} = 55.1 min (major), t_{R2} = 67.5 min (minor).

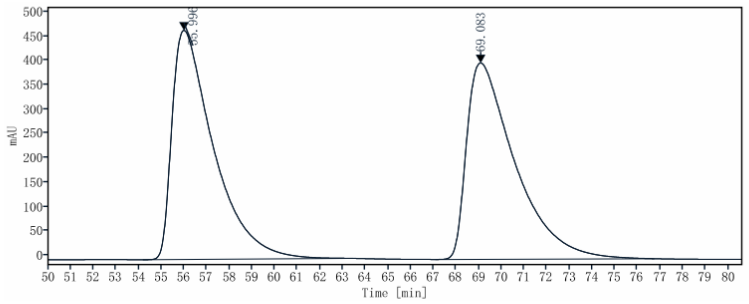

RetTime[min]	Type	Width[min]	Area[mAU*s]	Height[mAU]	Area%
55.996	BM m	1.8835	59690.0391	470.3740	49.9905
69.083	MM m	2.1790	59712.7651	403.1632	50.0095

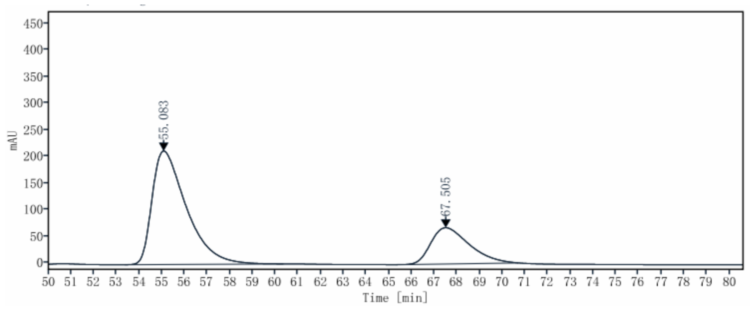

RetTime[min]	Type	Width[min]	Area[mAU*s]	Height[mAU]	Area%
55.083	BB	6.9800	22402.3348	213.3853	73.6631
67.505	MM m	1.7708	8009.5626	68.1180	26.3369

Supplementary Figure 37. HPLC Trace of **3am**

5. Further Studies of the Reaction

5.1 Procedure for Gram-scale Experiment

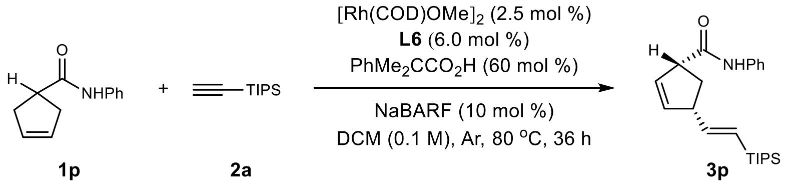

To an oven-dried 100-mL schlenk tube equipped with a tefloncoated magnetic stir bar was added the substrate **1p** (0.94 g, 5.0 mmol, 1.0 equiv), $[Rh(COD)OMe]_2$ (60 mg, 2.5 mol%), **L6** (0.14 g, 6.0 mol%), NaBARF (0.44 g, 10 mol%) and $PhMe_2CCO_2H$ (0.50 g, 60 mol%). The vial is thoroughly flushed with Ar, and **2a** (2.7 g, 15 mmol, 3.0 equiv), as well as DCM (40 mL) was added under Ar atmosphere. Then the reaction mixture was stirred at 80 ^{o}C for 36 h. After the reaction vessel was cooled to room temperature, the solution was concentrated in vacuum and purified by careful chromatography on silica gel (PE/EA = 30/1) to afford the desired product **3p** (93%, 95% ee).

5.2 Transformations of 3p and 3ai

Synthesis of (1S,3R)-N-phenyl-3-(2-(triisopropylsilyl)ethyl)cyclopentane-1-carboxamide (4)

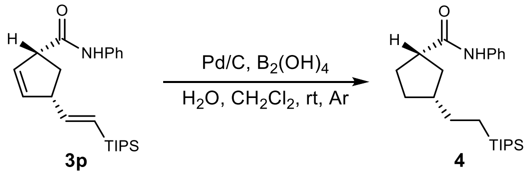

The hydrogenation of **3p** was performed by adapting a literature procedure[3]. To an oven-dried 25-mL schlenk tube equipped with a tefloncoated magnetic stir bar was added **3p** (74 mg, 0.20 mmol, 1.0 equiv), tetrahydroxydiboron (20 mg, 0.22 mmol, 1.1 equiv), Pd/C (10 wt %, 20 mg), Then the schlenk tube was evacuated and filled with argon (three cycles). After that, H_2O (33 mg, 1.8 mmol, 9.0 equiv), CH_2Cl_2 (2.0 mL), was added to the tube via a syringe under a positive flow of argon. The resulting mixture was stirred at room temperature for 5 h. The solvent was concentrated in vacuum and the crude product purified by flash chromatography on silica gel (PE/EA = 50/1) to afford the desired product **4** in 95% yield with 93% ee.

Synthesis of N-(((1R,4S)-4-((E)-2-(triisopropylsilyl)vinyl)cyclopent-2-en-1-yl)methyl)aniline (5)

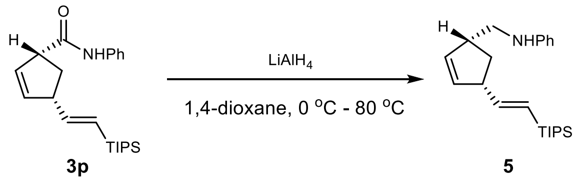

3p 5

To an oven-dried 25-mL two-necked bottle equipped with a tefloncoated magnetic stir bar was added $LiAlH_4$ (19 mg, 0.50 mmol, 2.5 equiv). Then bottle was evacuated and filled with argon (three cycles). After that, the solution of **3p** (74 mg, 0.20 mmol, 1.0 equiv) in 1,4-dioxane (2.0 mL) was added dropwised via a syringe at 0 °C. The mixture was stirred at 80 °C for 20 h. Then, the mixture was quenched by $Na_2SO_4 \cdot 10H_2O$, and filtered through a short pad of celite washed with CH_2Cl_2. The solvent was concentrated in vacuum and the crude product was purified by flash chromatography on silica gel (PE/EA = 100/1) to afford the desired product **5** in 95% yield with 92% ee.

Synthesis of (1R,4S)-4-((E)-2-bromovinyl)-N-phenylcyclopent-2-ene-1-carboxamide (6) and (1R,4S)-4-((Z)-2-bromovinyl)-N-phenylcyclopent-2-ene-1-carboxamide (7)

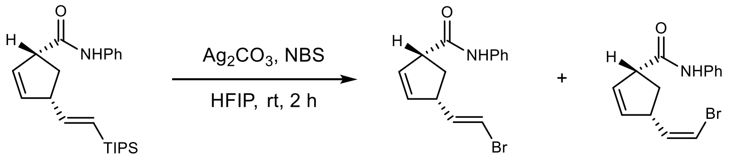

3p 6 7

The brominedesilylation of of vinylsilane moiety was performed by adapting a literature procedure.[4] To a stirred solution of **3p** (74 mg, 0.20 mmol 1.0 equiv) in 1,1,1,3,3,3-hexafluoropropan-2-ol (HFIP, 1.0 mL) at 0 °C, silver carbonate (17 mg, 0.060 mmol, 30 mol%) was added. The reaction mixture was protected from light before the addition of N-Bromosuccinimide (43 mg, 0.24 mmol, 1.2 equiv). The resulting mixture was stirred at room temperature for 2 h. The mixture was filtered through a short pad of celite washed with CH_2Cl_2. The solvent was concentrated in vacuum and the crude product was purified by flash chromatography on silica gel (PE/EA = 10/1) to afford the desired product **6** in 40% yield, 95% *ee* and **7** in 45% yield, 95% *ee.*

Synthesis of (1R,4R)-N,1-diphenyl-4-vinylcyclopent-2-ene-1-carboxamide (8)

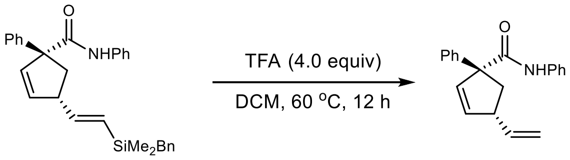

3ai **8**

To a solution of **3ai** (88 mg, 0.2mmol, 1.0 equiv) in DCM (2.0 mL) was added TFA (91 mg, 0.80 mmol, 4.0 equiv), the resulting mixture was stirred at 60 ^{o}C for 12 h. After that, the solvent was concentrated in vacuum and the crude product purified by flash chromatography on silica gel (PE/EA = 30/1) to afford the desired product **8** in 94% yield with 94% ee.

Synthesis of (1R,4R)-N,1-diphenyl-4-((E)-styryl)cyclopent-2-ene-1-carboxamide (9)

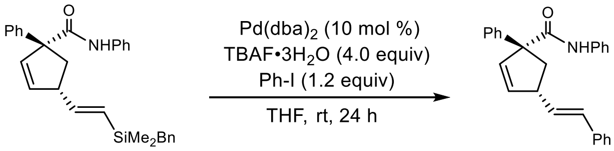

3ai **9**

The Hiyama crosscoupling was performed by adapting a literature procedure[5]. To an oven-dried 10-mL schlenk tube equipped with a tefloncoated magnetic stir bar was added **3ai** (88 mg, 0.20 mmol, 1.0 equiv), TBAF 3H_2O (0.25 g, 0.80 mmol, 4.0 equiv), $Pd(dba)_2$ (12 mg, 10 mol%). Then the schlenk tube was evacuated and filled with argon (three cycles). After that, iodobenzene (49 mg, 0.24 mmol, 1.2 equiv), THF (2.0 mL) was added to the tube via a syringe under a positive flow of argon. The resulting mixture was stirred at room temperature for 24 h. The solvent was concentrated in vacuum and the crude product purified by flash chromatography on silica gel (PE/EA = 30/1) to afford the desired product **9** in 81% yield with 94% ee.

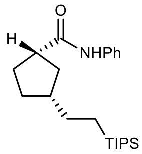

(1S,3R)-N-phenyl-3-(2-(triisopropylsilyl)ethyl)cyclopentane-1-carboxamide (4)

71.0 mg, 95% yield, 93% *ee*, d.r. > 20:1; R_f = 0.6 (PE/EA = 5/1); Colorless oil. $[\alpha]_D^{20}$ = +7.9 (*c* = 0.4, MeOH).

^{1}H NMR (300 MHz, $CDCl_3$) δ 7.54 – 7.51 (m, 2H), 7.33 – 7.25 (m, 3H), 7.11 – 7.05 (m, 1H), 2.77 – 2.66 (m, 1H), 2.20 – 2.11 (m, 1H), 2.05 – 1.77 (m, 4H), 1.56 – 1.29 (m, 4H), 1.03 (s, 21H), 0.64 – 0.54 (m, 2H) ppm.

^{13}C NMR (75 MHz, $CDCl_3$) δ 174.5, 138.2, 129.0, 124.0, 119.7, 46.9, 44.8, 37.3, 31.7, 30.1, 29.2, 18.9, 10.9, 8.3 ppm.

HRMS (ESI) calcd for $[C_{23}H_{39}NO_3Si+H]^+$ 374.2874, found 374.2876.

HPLC: Daicel Chiralcel OD-H, *n*-hexane/isopropanol 90/10, flow rate = 1 mL/min, uv-vis λ = 250 nm, t_{R1} = 7.9 min (minor), t_{R2} = 14.1 min (major).

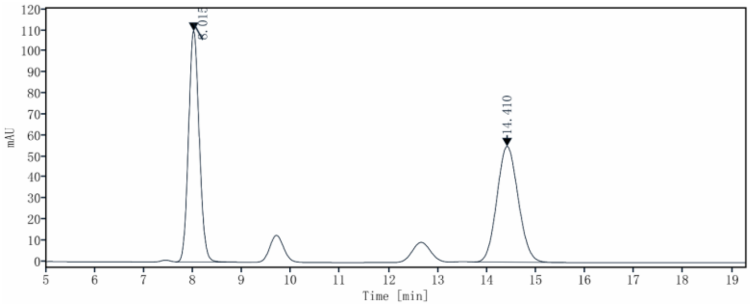

RetTime[min]	Type	Width[min]	Area[mAU*s]	Height[mAU]	Area%
8.015	VB	1.5328	1673.1818	110.2154	50.0945
14.410	BB	1.8300	1666.8683	55.2402	49.9055

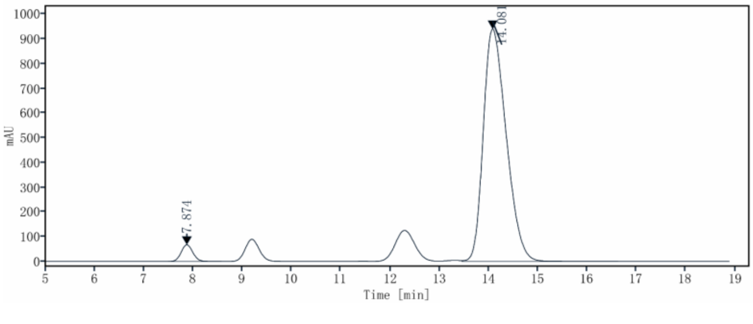

RetTime[min]	Type	Width[min]	Area[mAU*s]	Height[mAU]	Area%
7.874	VB	1.0807	1105.3763	67.4035	3.5128
14.081	VB	3.1671	30361.4722	939.9098	96.4872

Supplementary Figure 38. HPLC Trace of **4**

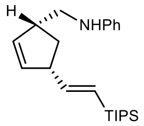

***N*-(((1R,4S)-4-((E)-2-(triisopropylsilyl)vinyl)cyclopent-2-en-1-yl)methyl)aniline (5)**

66.1 mg, 93% yield, 92% *ee*, d.r. > 20:1, E/Z > 20:1; R_f = 0.4 (PE/EA = 50/1); Colorless oil. $[\alpha]_D^{20}$ = -66 (*c* = 0.19, MeOH).

^{1}H NMR (300 MHz, $CDCl_3$) δ 7.20 – 7.13 (m, 2H), 6.71 – 6.66 (m, 1H), 6.62 – 6.58 (m, 2H), 6.03 (dd, *J* = 18.8, 7.1 Hz, 1H), 5.78 – 5.72 (m, 2H), 5.52 (dd, *J* = 18.8, 1.2 Hz, 1H), 3.65 (brs, 1H), 3.45 – 3.35 (m, 1H), 3.19 – 3.12 (m, 1H), 3.09 – 3.03 (m, 2H), 2.37 (dt, *J* = 13.0, 8.2 Hz, 1H), 1.36 (dt, *J* = 13.2, 6.8 Hz, 1H), 1.13 – 0.96 (m, 21H) ppm.

^{13}C NMR (75 MHz, $CDCl_3$) δ 152.5, 148.5, 135.6, 133.0, 129.3, 122.3, 117.2, 112.8, 52.6, 49.0, 45.9, 35.1, 18.7, 10.9 ppm.

HRMS (ESI) calcd for $[C_{23}H_{37}NSi+H]^+$ 356.2768, found 356.2773.

HPLC: Daicel Chiralcel OD-H, *n*-hexane/isopropanol 99/1, flow rate = 0.5 mL/min, uv-vis λ = 250 nm, t_{R1} = 12.3 min (major), t_{R2} = 13.1 min (minor).

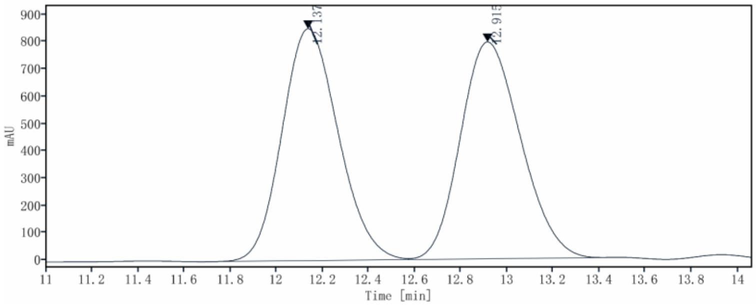

RetTime[min]	Type	Width[min]	Area[mAU*s]	Height[mAU]	Area%
12.137	BM m	0.2649	14416.3850	849.7186	49.9773
12.915	MM m	0.2839	14429.4523	794.4171	50.0227

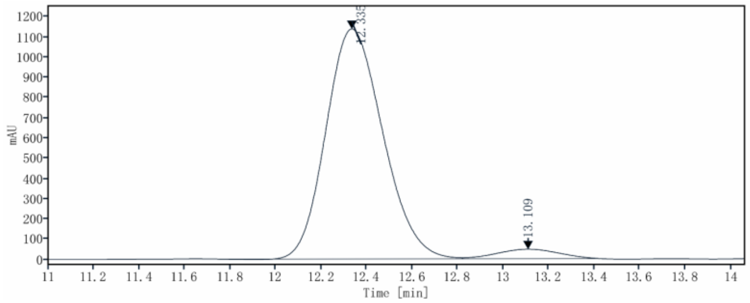

RetTime[min]	Type	Width[min]	Area[mAU*s]	Height[mAU]	Area%
12.335	MM m	0.2717	19737.4625	1136.1540	95.7549
13.109	MM m	0.3017	875.0185	46.4599	4.2451

Supplementary Figure 39. HPLC Trace of **5**

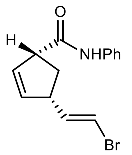

(1R,4S)-4-((E)-2-bromovinyl)-N-phenylcyclopent-2-ene-1-carboxamide (6)

40% yield, 95% *ee*, d.r. > 20:1; R_f = 0.4 (PE/EA = 5/1); White solid, m.p. 118 – 120 ^{o}C; $[\alpha]_D^{20}$ = +26 (*c* = 0.11, MeOH).

^{1}H NMR (300 MHz, $CDCl_3$) δ 7.56 (brs, 1H), 7.52 – 7.49 (m, 2H), 7.34 – 7.27 (m, 2H), 7.13 – 7.07 (m, 1H), 6.21 (dd, *J* = 13.6, 7.6 Hz, 1H), 6.12 (d, *J* = 13.6 Hz, 1H), 5.86 (s, 2H), 3.58 – 3.52 (m, *J* = 8.9, 6.6, 2.3 Hz, 1H), 3.46 – 3.38 (m, 1H), 2.50 (dt, *J* = 13.4, 8.7 Hz, 1H), 1.93 (dt, *J* = 13.3, 6.6 Hz, 1H) ppm.

^{13}C NMR (75 MHz, $CDCl_3$) δ 172.4, 140.3, 137.8, 136.5, 130.4, 129.0, 124.5, 120.2, 105.2, 53.3, 49.0, 34.1 ppm.

HRMS (ESI) calcd for $[C_{14}H_{14}BrNO+H]^+$ 292.0332, found 292.0329.

HPLC: Daicel Chiralcel OD-H, *n*-hexane/isopropanol 90/10, flow rate = 1 mL/min, uv-vis λ = 250 nm, t_{R1} = 15.1 min (minor), t_{R2} = 25.9 min (major).

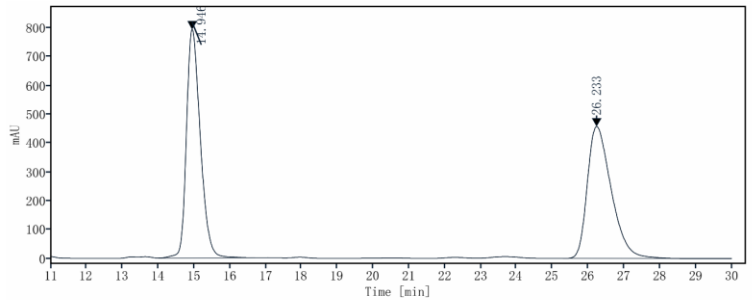

RetTime[min]	Type	Width[min]	Area[mAU*s]	Height[mAU]	Area%
14.946	VB	2.6792	21300.5994	793.2071	50.0538
26.233	BBA	4.7100	21254.8351	458.0343	49.9462

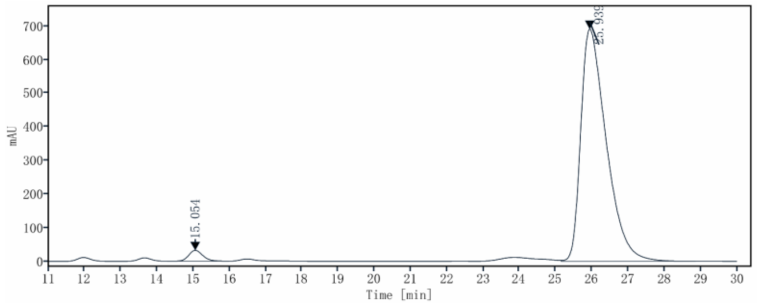

RetTime[min]	Type	Width[min]	Area[mAU*s]	Height[mAU]	Area%
15.054	BB	1.7000	866.5453	32.1778	2.5111
25.939	VB	3.7003	33641.7325	690.5022	97.4889

Supplementary Figure 40. HPLC Trace of **6**

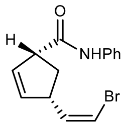

(1R,4S)-4-((Z)-2-bromovinyl)-N-phenylcyclopent-2-ene-1-carboxamide (7)

45% yield, 95% *ee*, d.r. > 20:1; R_f = 0.6 (PE/EA = 5/1); White solid, m.p. 98 – 100 ^oC; $[\alpha]_D^{20}$ = +116 (c = 0.12, MeOH).

^{1}H NMR (300 MHz, $CDCl_3$) δ 7.67 (brs, 1H), 7.54 – 7.49 (m, J = 8.8, 1.9 Hz, 2H), 7.33 – 7.26 (m, 2H), 7.13 – 7.07 (m, 1H), 6.14 (dd, J = 6.9, 0.9 Hz, 1H), 6.07 (dd, J = 8.4, 6.9 Hz, 1H), 5.88 – 5.83 (m, 2H), 3.90 – 3.81 (m, J = 12.1, 8.5, 4.2 Hz, 1H), 3.62 – 3.53 (m, J = 8.9, 6.7, 2.1 Hz, 1H), 2.56 (dt, J = 13.3, 8.7 Hz, 1H), 1.91 (dt, J = 13.1, 6.5 Hz, 1H) ppm.

^{13}C NMR (75 MHz, $CDCl_3$) δ 172.6, 137.8, 137.5, 136.7, 130.1, 129.0, 124.5, 120.1, 107.5, 53.4, 46.0, 34.0 ppm.

HRMS (ESI) calcd for $[C_{14}H_{14}BrNO+H]^+$ 292.0332, found 292.0331.

HPLC: Daicel Chiralcel OD-H, *n*-hexane/isopropanol 90/10, flow rate = 1 mL/min, uv-vis λ = 250 nm, t_{R1} = 13.2 min (minor), t_{R2} = 17.7 min (major).

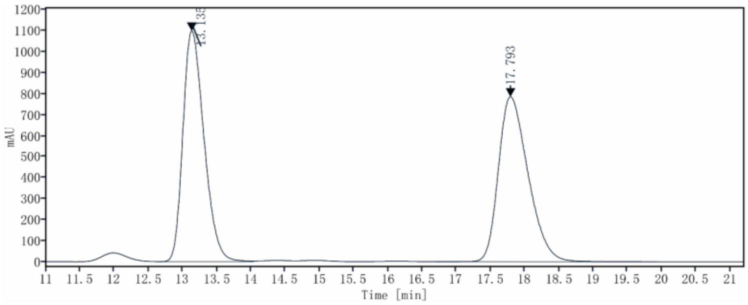

RetTime[min]	Type	Width[min]	Area[mAU*s]	Height[mAU]	Area%
13.135	BV	1.3783	23557.5357	1099.6940	49.8610
17.793	BB	3.2333	23688.8872	786.8297	50.1390

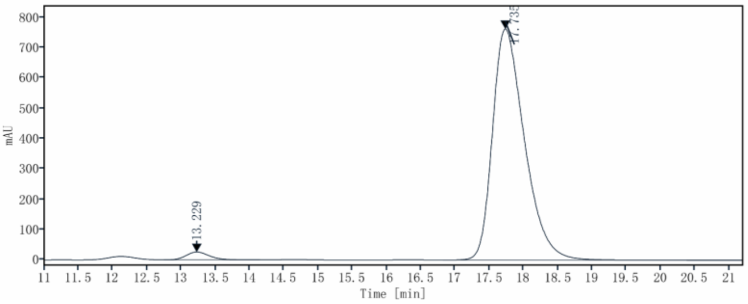

RetTime[min]	Type	Width[min]	Area[mAU*s]	Height[mAU]	Area%
13.229	VB	1.4021	619.5598	26.1675	2.4356
17.735	BB	4.8800	24817.8609	764.2150	97.5644

Supplementary Figure 41. HPLC Trace of **7**

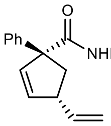

(1R,4R)-N,1-diphenyl-4-vinylcyclopent-2-ene-1-carboxamide (8)

54.3 mg, 94% yield, 94% *ee*, d.r. > 20:1; R_f = 0.5 (PE/EA = 10/1); White solid, m.p. 105 – 107 ^{o}C; $[\alpha]_D^{20}$ = +24 (*c* = 0.16, MeOH).

^{1}H NMR (300 MHz, $CDCl_3$) δ 7.42 – 7.33 (m, 6H), 7.32 – 7.24 (m, 3H), 7.09 – 7.04 (m, 1H), 7.00 (brs, 1H), 6.14 (dd, *J* = 5.5, 2.3 Hz, 1H), 5.99 (dd, *J* = 5.6, 2.1 Hz, 1H), 5.94 – 5.83 (m, 1H), 5.11 – 4.98 (m, 2H), 3.43 (q, *J* = 7.6 Hz, 1H), 2.83 (dd, *J* = 13.2, 6.2 Hz, 1H), 2.44 (dd, *J* = 13.2, 8.1 Hz, 1H) ppm.

^{13}C NMR (75 MHz, $CDCl_3$) δ 173.0, 143.4, 140.7, 138.0, 137.9, 132.8, 129.0, 129.0, 127.4, 126.7, 124.3, 119.8, 114.5, 66.2, 49.2, 42.6 ppm.

HRMS (ESI) calcd for $[C_{20}H_{19}NO+H]^+$ 290.1539, found 290.1540.

HPLC: Daicel Chiralcel OD-H, *n*-hexane/isopropanol 95/5, flow rate = 1 mL/min, uv-vis λ = 250 nm, t_{R1} = 8.4 min (major), t_{R2} = 10.2 min (minor).

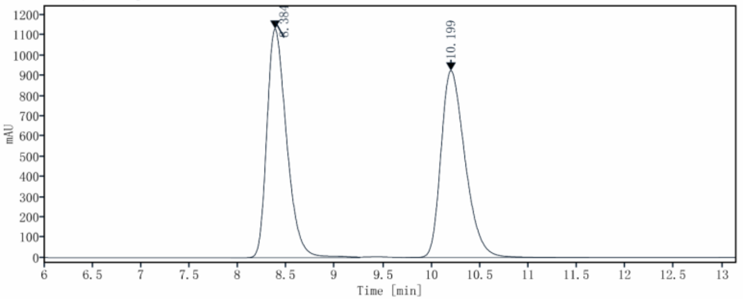

RetTime[min]	Type	Width[min]	Area[mAU*s]	Height[mAU]	Area%
8.384	BV	1.3346	16074.8730	1132.2751	50.0260
10.199	VB	1.8902	16058.1446	924.6884	49.9740

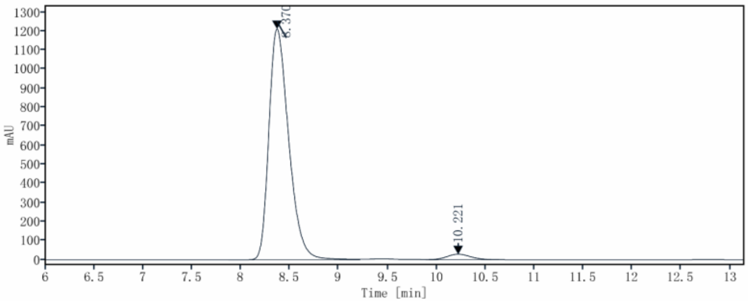

RetTime[min]	Type	Width[min]	Area[mAU*s]	Height[mAU]	Area%
8.370	BV	1.3441	17081.4342	1212.5589	97.1598
10.221	VM m	0.2663	499.3344	29.0895	2.8402

Supplementary Figure 42. HPLC Trace of **8**

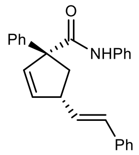

(1R,4R)-N,1-diphenyl-4-((E)-styryl)cyclopent-2-ene-1-carboxamide (9)

59.2 mg, 81% yield, 94% *ee*, d.r. > 20:1, E/Z > 20:1; R_f = 0.5 (PE/EA = 10/1); Colorless oil. $[\alpha]_D^{20}$ = -54 (*c* = 0.10, MeOH).

^{1}H NMR (300 MHz, $CDCl_3$) δ 7.42 – 7.20 (m, 14H), 7.07 (t, *J* = 7.4 Hz, 1H), 7.00 (brs, 1H), 6.44 (d, *J* = 15.8 Hz, 1H), 6.26 (dd, *J* = 15.8, 8.2 Hz, 1H), 6.18 (dd, *J* = 5.4, 2.3 Hz, 1H), 6.03 (dd, *J* = 5.5, 2.1 Hz, 1H), 3.60 (q, *J* = 7.6 Hz, 1H), 2.92 (dd, *J* = 13.3, 6.5 Hz, 1H), 2.51 (dd, *J* = 13.3, 8.0 Hz, 1H) ppm.

^{13}C NMR (75 MHz, $CDCl_3$) δ 173.0, 143.4, 138.0, 137.9, 137.3, 133.1, 132.4, 129.9, 129.1, 129.0, 128.6, 127.5, 127.3, 126.8, 126.3, 124.4, 119.9, 66.4, 48.7, 42.9 ppm.

HRMS (ESI) calcd for $[C_{26}H_{23}NO+H]^+$ 366.1852, found 366.1856.

HPLC: Daicel Chiralcel OD-H, *n*-hexane/isopropanol 95/5, flow rate = 0.5 mL/min, uv-vis λ = 250 nm, t_{R1} = 42.9 min (major), t_{R2} = 49.0 min (minor).

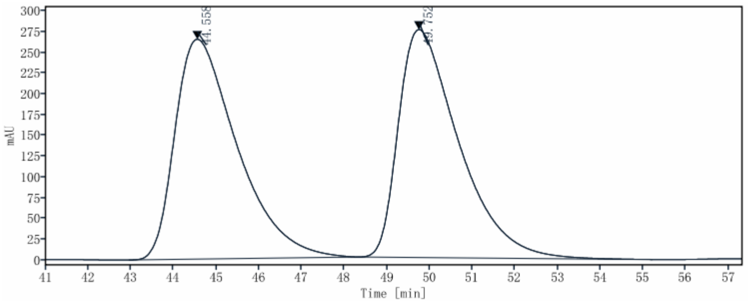

RetTime[min]	Type	Width[min]	Area[mAU*s]	Height[mAU]	Area%
44.558	BB	5.3900	26842.4715	264.7488	49.8779
49.752	BB	6.8800	26973.8393	274.5296	50.1221

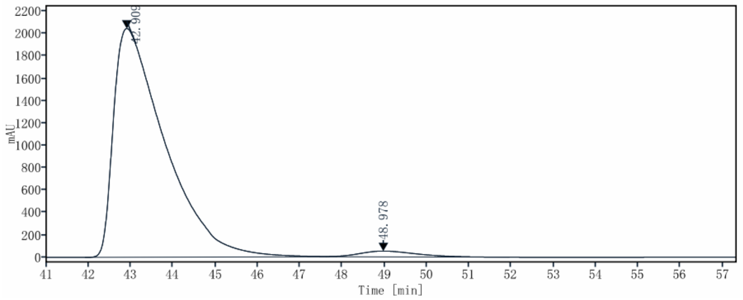

RetTime[min]	Type	Width[min]	Area[mAU*s]	Height[mAU]	Area%
42.909	BM m	1.2408	172810.7111	2047.1484	97.2076
48.978	MB m	1.4499	4964.2282	52.3706	2.7924

Supplementary Figure 43. HPLC Trace of **9**

6. Mechanistic Studies

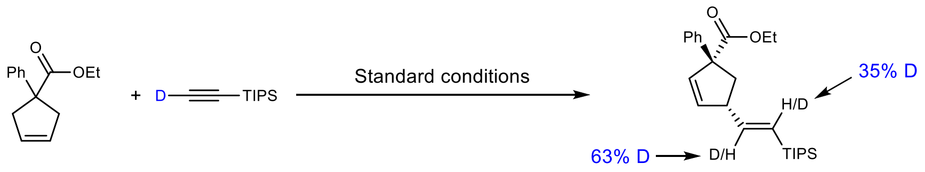

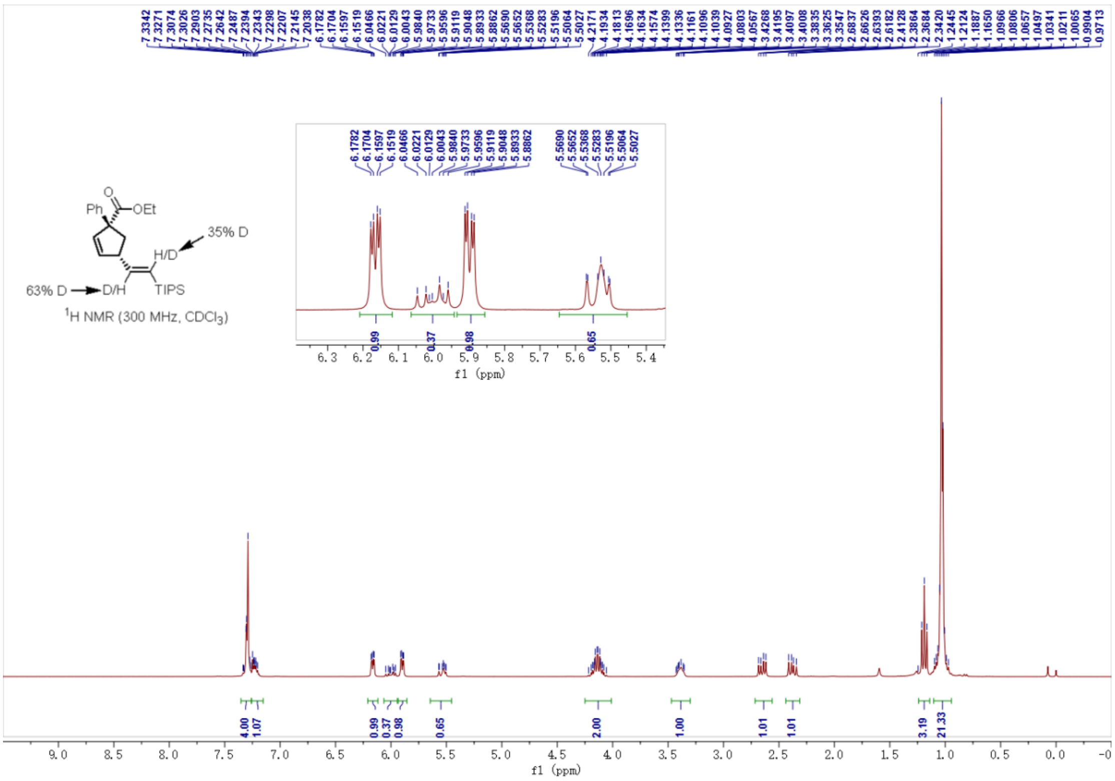

Supplementary Figure 44. ^{1}H NMR (300 MHz, $CDCl_3$) of **[D]$_1$-3aa**

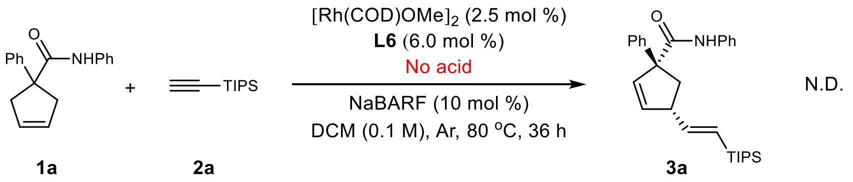

To an oven-dried 10-mL schlenk tube equipped with a tefloncoated magnetic stir bar was added **1a** (52.6 mg, 0.200 mmol, 1.00 equiv), $[Rh(COD)OMe]_2$ (2.4 mg, 2.5 mol%), **L6** (5.4 mg, 6.0 mol%) and NaBARF (17.7 mg, 10.0 mol%). The vial is thoroughly flushed with Ar, **2a** (109 mg, 0.600 mmol, 3.00 equiv), as well as DCM (2.0 mL) was added via a syringe under Ar atmosphere. Then the reaction mixture was stirred at 80 ^{o}C for 36 h. No desired product was detected with 97% recovery of **1a**.

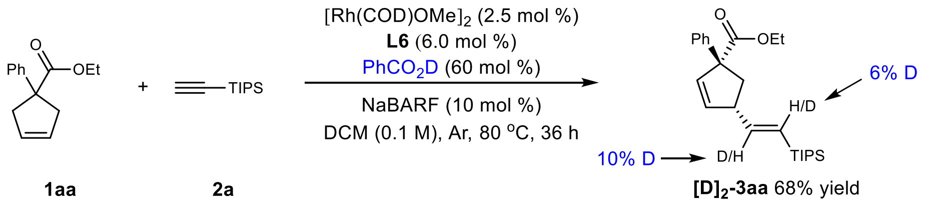

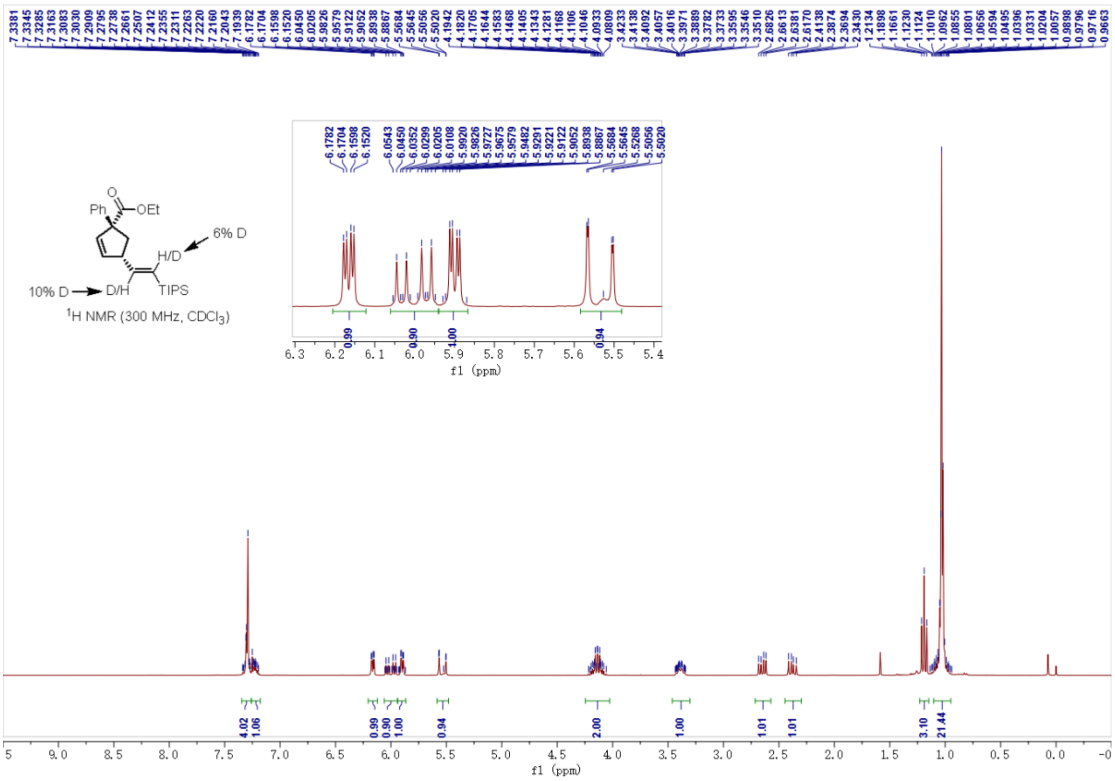

Supplementary Figure 45. ^{1}H NMR (300 MHz, $CDCl_3$) of **[D]$_2$-3aa**

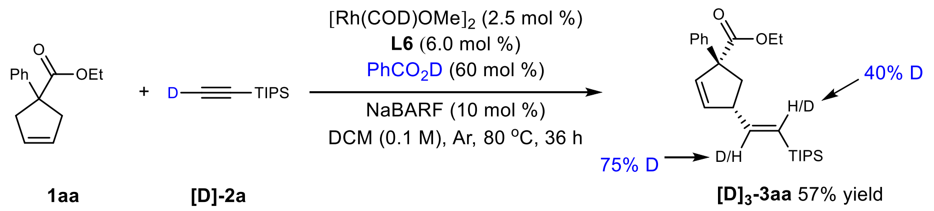

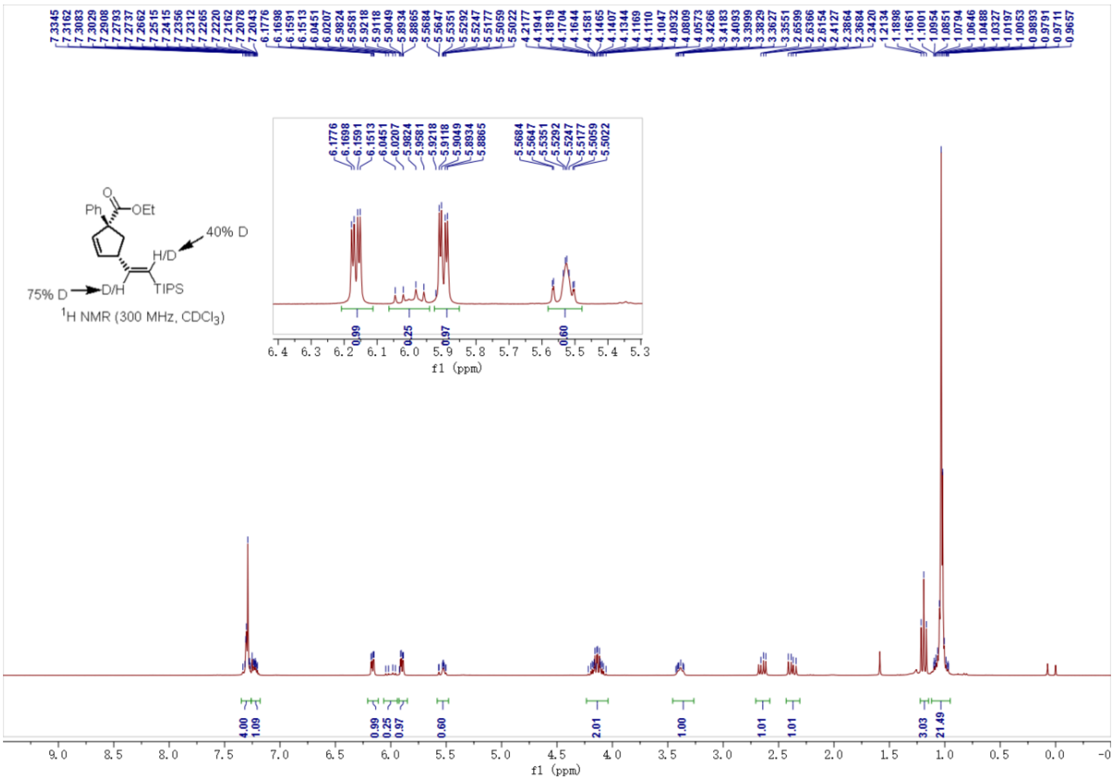

Supplementary Figure 46. ^{1}H NMR (300 MHz, $CDCl_3$) of **[D]$_3$-3aa**

Supplementary Notes

Supplementary Note 1. Reactions of 1a with other terminal alkynes.

1-heptyne (**2g**) and 4-ethynylanisole (**2h**) were tested under the standard conditions. Unfortunately, simple alkyl and aryl substituted acetylenes were not yet compatible with our reaction system to give the desired products **3ak** and **3al**.

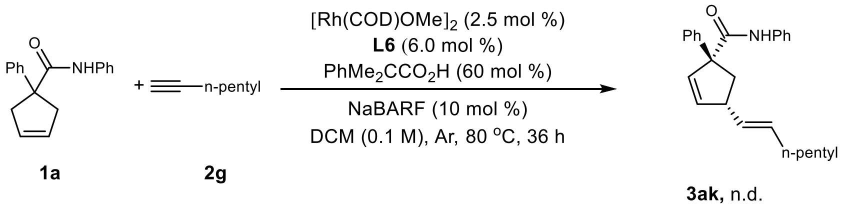

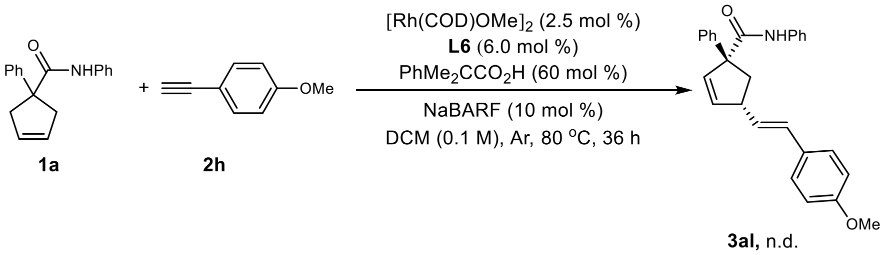

Under standard conditions, when **2g** was used as the substrate, the reaction became very confusing and complex, and it is difficult for us to isolate and identify the main products.

When 4-ethynylanisole (**2h**) was tested, a branched byproduct **3al'** was isolated in 17% yield with 8% ee under the standard conditions.

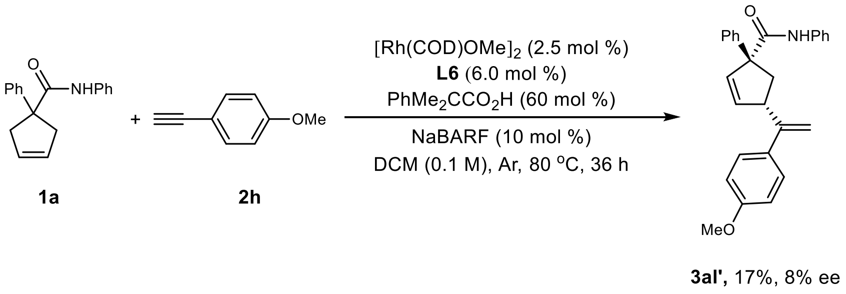

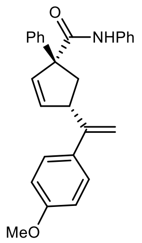

(1R,4S)-4-(1-(4-methoxyphenyl)vinyl)-N,1-diphenylcyclopent-2-ene-1-carboxamide (3al')

6.7 mg, 17% yield, 8% *ee*, d.r. > 20:1; R_f = 0.3 (PE/EA = 10/1); yellow oil.

^{1}H NMR (300 MHz, $CDCl_3$) δ 7.41 – 7.23 (m, 12H), 7.09 – 7.03 (m, 2H), 6.88 – 6.83 (m, 2H), 6.25 (dd, *J* = 5.6, 2.3 Hz, 1H), 6.17 (dd, *J* = 5.6, 2.0 Hz, 1H), 5.26 (s, 1H), 5.07 (s, 1H), 3.99 – 3.92 (m, 1H), 3.79 (s, 3H), 2.81 (dd, *J* = 13.3, 7.1 Hz, 1H), 2.61 (dd, *J* = 13.3, 8.3 Hz, 1H) ppm.

^{13}C NMR (75 MHz, $CDCl_3$) δ 172.8, 159.2, 150.1, 143.4, 138.0, 137.4, 133.8, 133.3, 129.0, 127.6, 127.4, 126.7, 124.3, 119.7, 113.7, 110.8, 65.9, 55.3, 49.1, 43.4 ppm.

HRMS (ESI) calcd for $[C_{27}H_{25}NO_2+H]^+$ 396.1958, found 396.1961.

HPLC: Daicel Chiralcel AD-H, n-hexane/isopropanol 75/25, flow rate = 0.5 mL/min, uv-vis λ = 250 nm, t_{R1} = 19.3 min (major), t_{R2} = 26.1 min (minor).

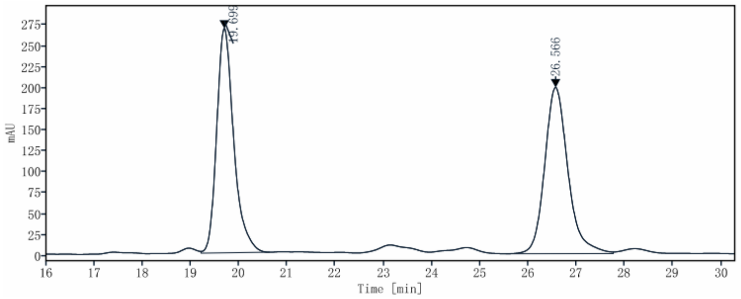

RetTime[min]	Type	Width[min]	Area[mAU*s]	Height[mAU]	Area%
19.699	VB	1.4216	6568.2491	267.3069	49.6825
26.566	BV	2.2275	6652.2036	198.0476	50.3175

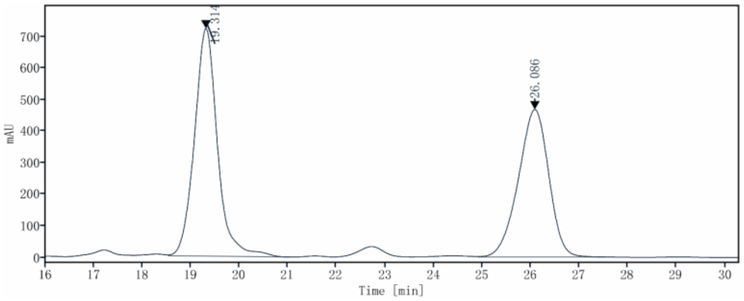

RetTime[min]	Type	Width[min]	Area[mAU*s]	Height[mAU]	Area%
19.314	VB	2.4987	23761.6831	721.8191	53.8884
26.086	VM m	0.6671	20332.5422	467.7741	46.1116

Supplementary Figure 47. HPLC Trace of **3al'**

Supplementary Note 2. Determination of the Absolute Configuration of the Product

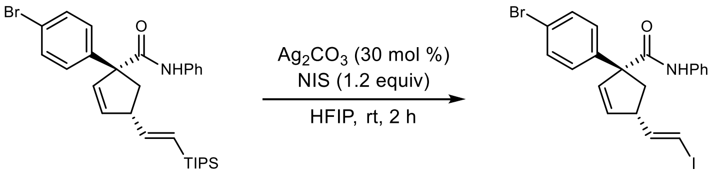

3f **10**

The iododesilylation of vinylsilane moiety was performed by adapting a literature procedure[4]. To a stirred solution of **3f** (105 mg, 0.200 mmol, 1.00 equiv) in 1,1,1,3,3,3- hexafluoropropan-2-ol (HFIP, 1.0 mL) at 0 ℃, Ag_2CO_3 (17 mg, 0.060 mmol, 30 mol%) was added. The reaction mixture was protected from light before the addition of N-Iodosuccinimide (54 mg, 0.24 mmol, 1.2 equiv). The resulting mixture was stirred at room temperature for 2 h. The mixture was filtered through a short pad of celite washed with CH_2Cl_2. The solvent was concentrated in vacuum and the crude product was purified by flash chromatography on silica gel (PE/EA = 20/1) to afford the desired product **10**.

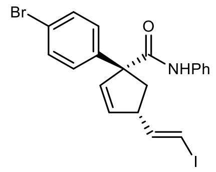

(1R,4S)-1-(4-bromophenyl)-4-((E)-2-iodovinyl)-N-phenylcyclopent-2-ene-1-carboxamide (10)

93% *ee*, d.r. > 20:1, E/Z > 20:1; R_f = 0.5 (PE/EA = 10/1); White solid, m.p. 170 – 172 °C; $[\alpha]_D^{20}$ = -46 (*c* = 0.11, MeOH).

^{1}H NMR (300 MHz, $CDCl_3$) δ 7.51 – 7.47 (m, 2H), 7.41 – 7.37 (m, 2H), 7.32 – 7.26 (m, 2H), 7.24 – 7.20 (m, 2H), 7.13 – 7.07 (m, 1H), 6.92 (brs, 1H), 6.58 (dd, *J* = 14.4, 8.4 Hz, 1H), 6.18 – 6.13 (m, 1H), 6.13 – 6.11 (m, 1H), 5.98 (dd, *J* = 5.5, 2.2 Hz, 1H), 3.49 -3.40 (m, 1H), 2.89 (dd, *J* = 13.5, 5.9 Hz, 1H), 2.32 (dd, *J* = 13.4, 8.2 Hz, 1H) ppm.

^{13}C NMR (75 MHz, $CDCl_3$) δ 171.9, 147.3, 142.2, 137.6, 136.8, 132.8, 132.2, 129.0, 128.4, 124.7, 121.6, 120.0, 75.9, 65.7, 51.5, 41.8 ppm.

HRMS (ESI) calcd for $[C_{20}H_{17}BrINO+H]^+$ 493.9611, found 493.9610.

HPLC: Daicel Chiralcel OD-H, *n*-hexane/isopropanol 95/5, flow rate = 0.5 mL/min, uv-vis λ = 250 nm, t_{R1} = 48.5 min (major), t_{R2} = 53.7 min (minor).

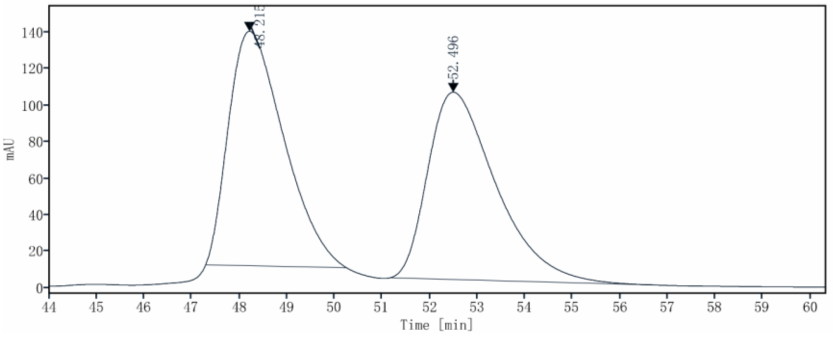

RetTime[min]	Type	Width[min]	Area[mAU*s]	Height[mAU]	Area%
48.215	MM m	1.2589	10405.5627	128.6148	50.3781
52.496	MM m	1.5055	10249.3563	102.7767	49.6219

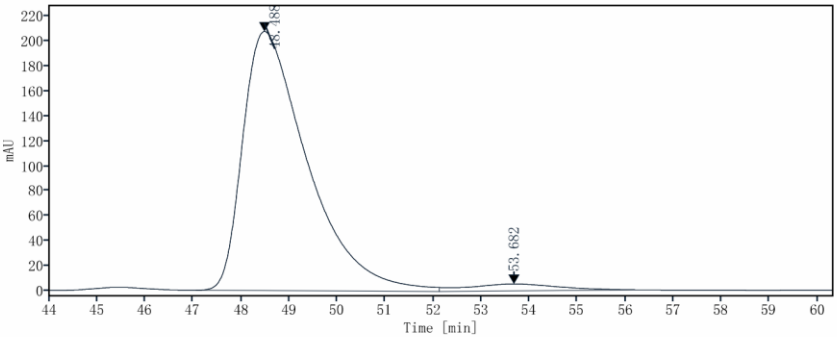

RetTime[min]	Type	Width[min]	Area[mAU*s]	Height[mAU]	Area%
48.488	BM m	1.3976	19484.4871	208.0601	96.4192
53.682	MB m	1.5657	723.6150	5.4974	3.5808

Supplementary Figure 48. HPLC Trace of **10**

Vapor diffusion crystallization method was used for crystal growth of **10**. The compound **10** was dissolved in DCM to make saturated solution in small vial and placed in closed bottle with another solvent as *n*-hexane.

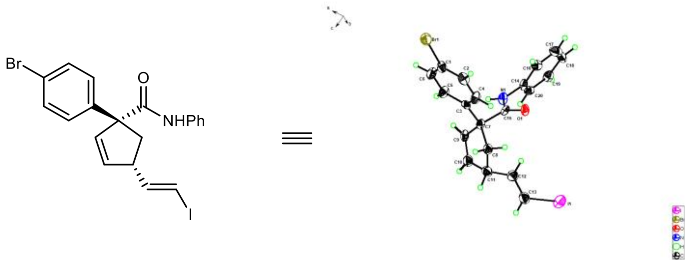

Supplementary Figure 49. ORTEP plot of the crystal structure of 10, and thermal ellipsoid is set at 50% probability.

Supplementary Table 2： X-ray Crystallographic Data of **10**

CCDC number	2064563
Bond precision	C-C = 0.0110 A Wavelength=0.71073
Cell	a=9.9538(2) b=10.1307(3) c=18.1659(5)<br>alpha=90 beta=90 gamma=90
Temperature	170 K
Volume	1831.83(8)
Space group	P 21 21 21
Sum formula	$C_{20}H_{17}BrINO$
Mr	494.15
Dx, g cm-3	1.792
Z	4
Mu (mm-1)	3.935
F000	960.0
h, k, 1max	12, 12, 22
Nref	3741
Tmin, Tmax	0.462, 0.745
Correction method	# Reported T Limits: Tmin=0.462 Tmax=0.745
AbsCorr	MULTI-SCAN
Data completeness	1.74/1.00
Theta(max)	26.395
R(reflections)	0.0376(3264)
wR2(reflections)	0.0376(3264)
S	1.070

7. ^{1}H and ^{13}C NMR Spectra of Title Compounds

***N*-phenyl-1-(*p*-tolyl)cyclopent-3-ene-1-carboxamide (1b)**

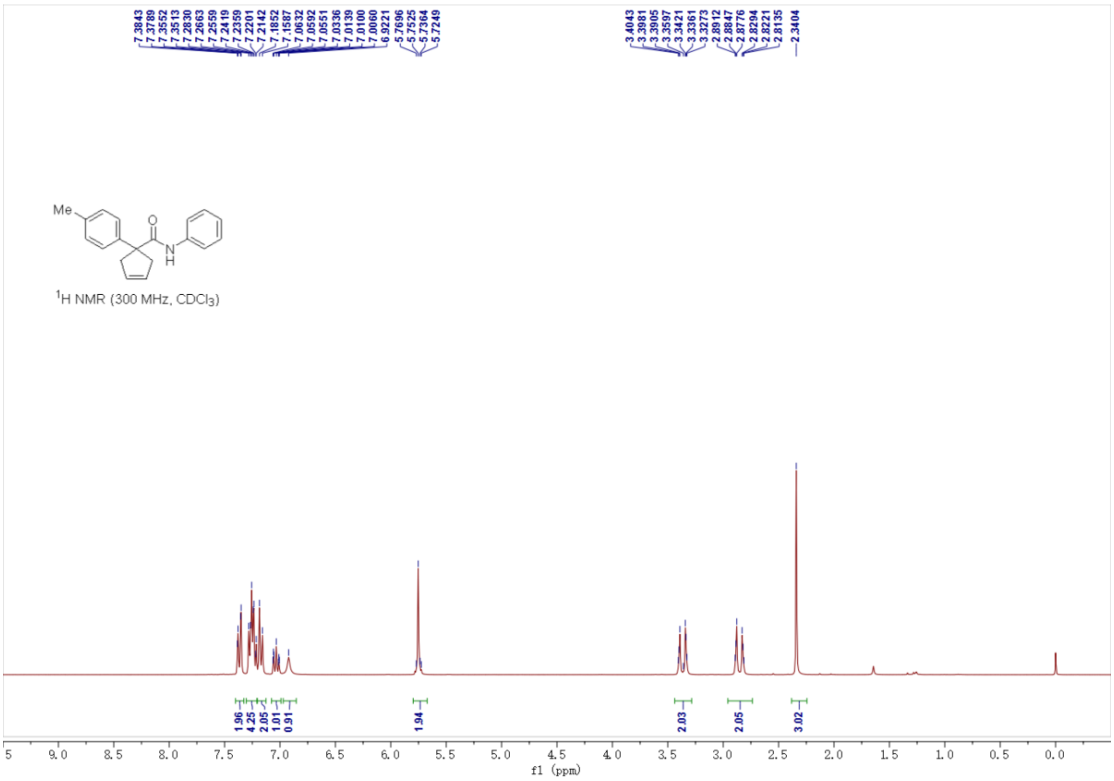

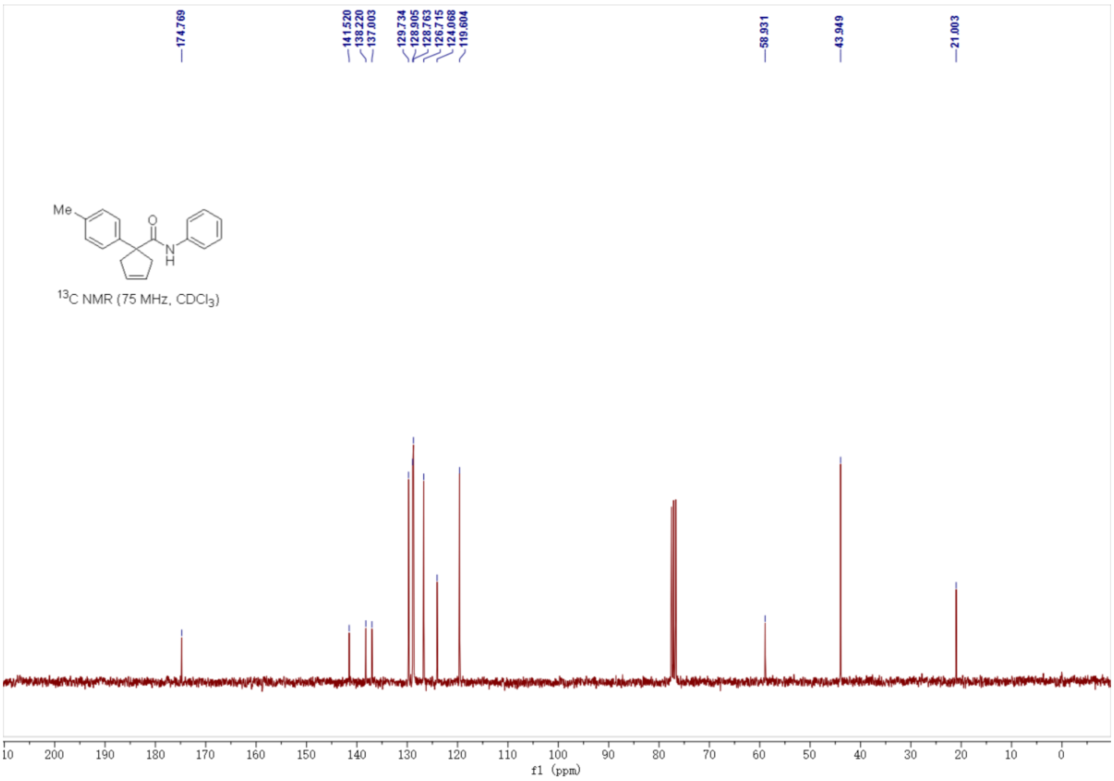

Supplementary Figure 50. ^{1}H and ^{13}C NMR spectra of **1b**

1-(4-methoxyphenyl)-N-phenylcyclopent-3-ene-1-carboxamide (1c)

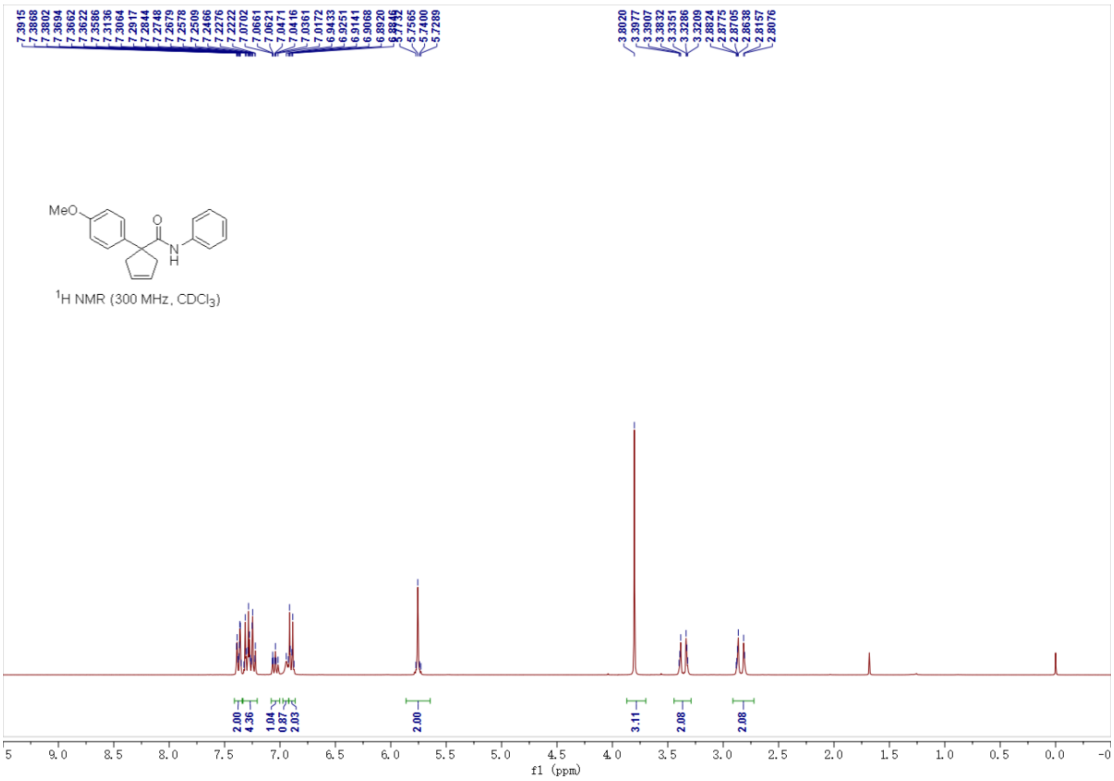

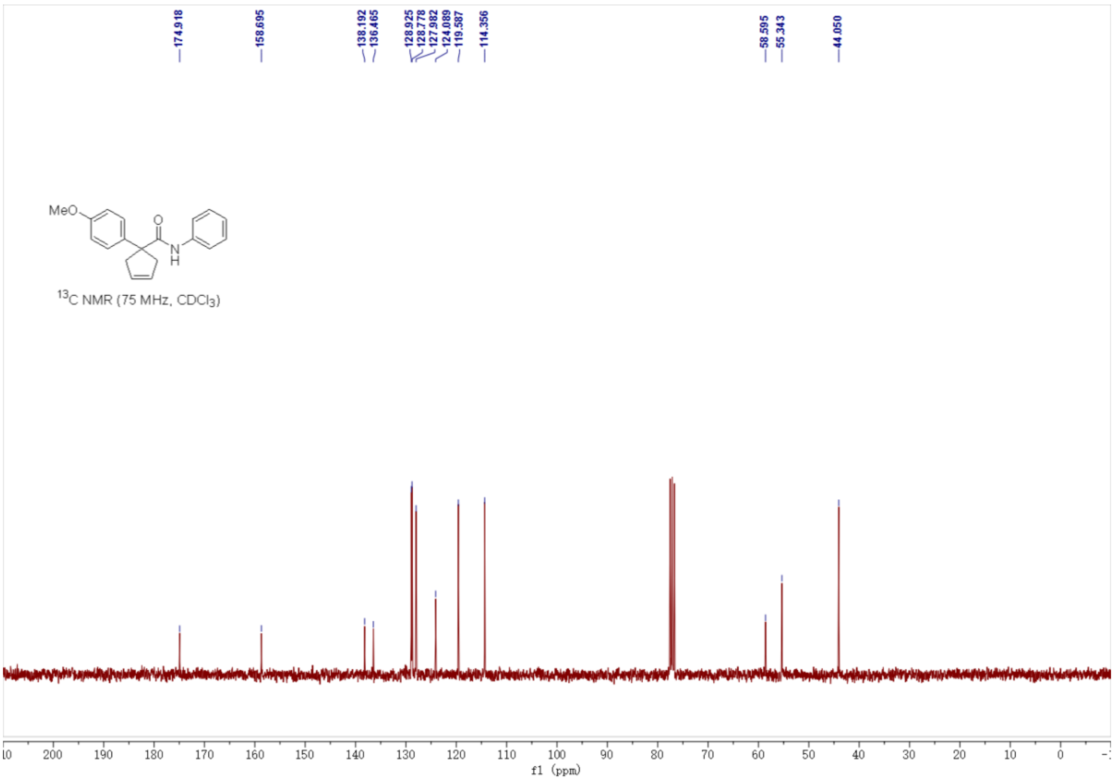

Supplementary Figure 51. ^{1}H and ^{13}C NMR spectra of **1c**

1-(4-(tert-butyl)phenyl)-N-phenylcyclopent-3-ene-1-carboxamide (1d)

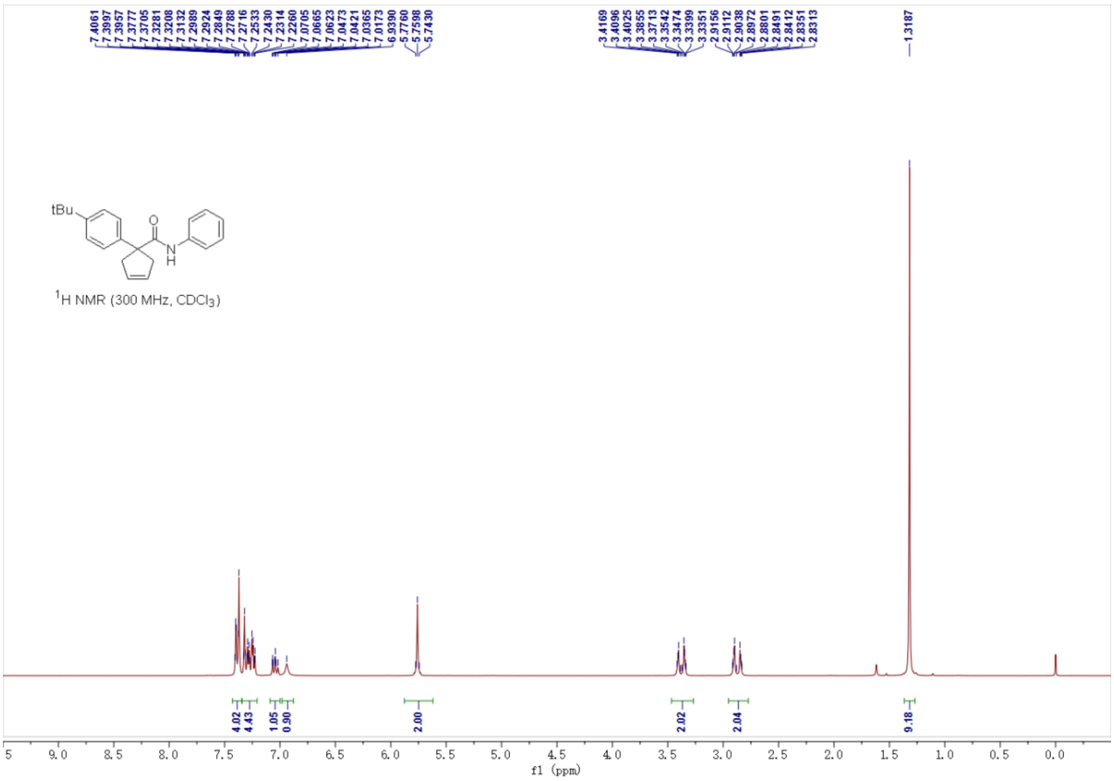

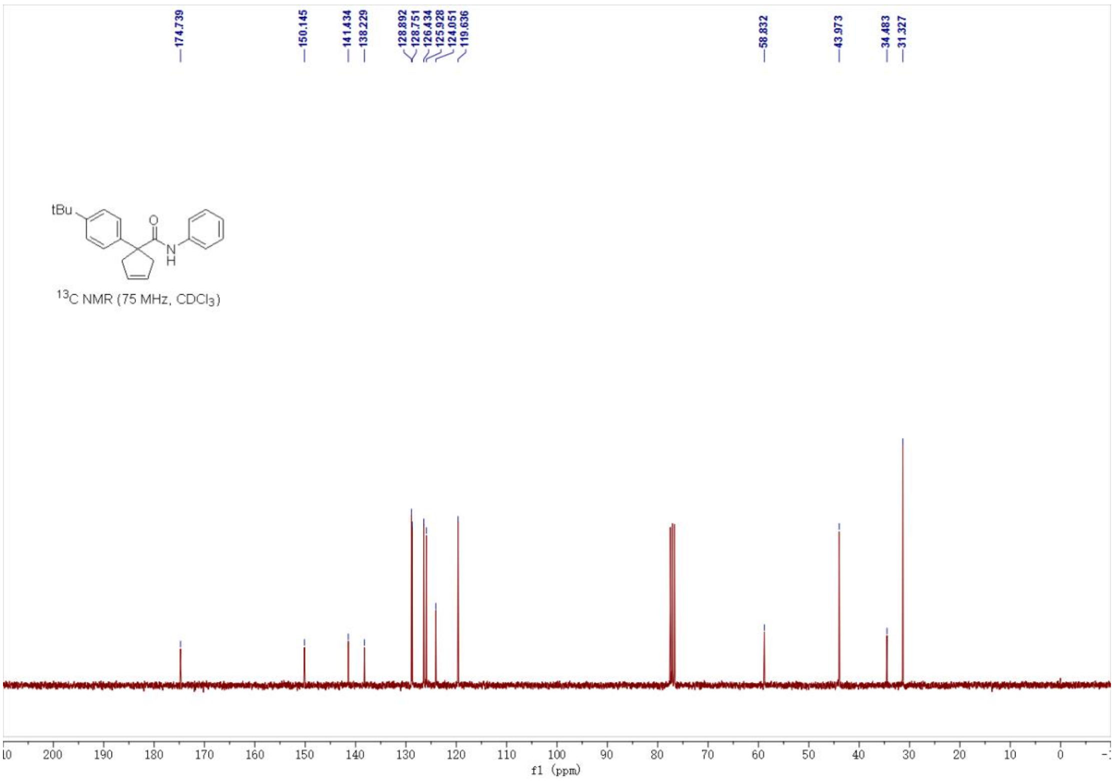

Supplementary Figure 52. ^{1}H and ^{13}C NMR spectra of **1d**

1-(4-fluorophenyl)-N-phenylcyclopent-3-ene-1-carboxamide (1e)

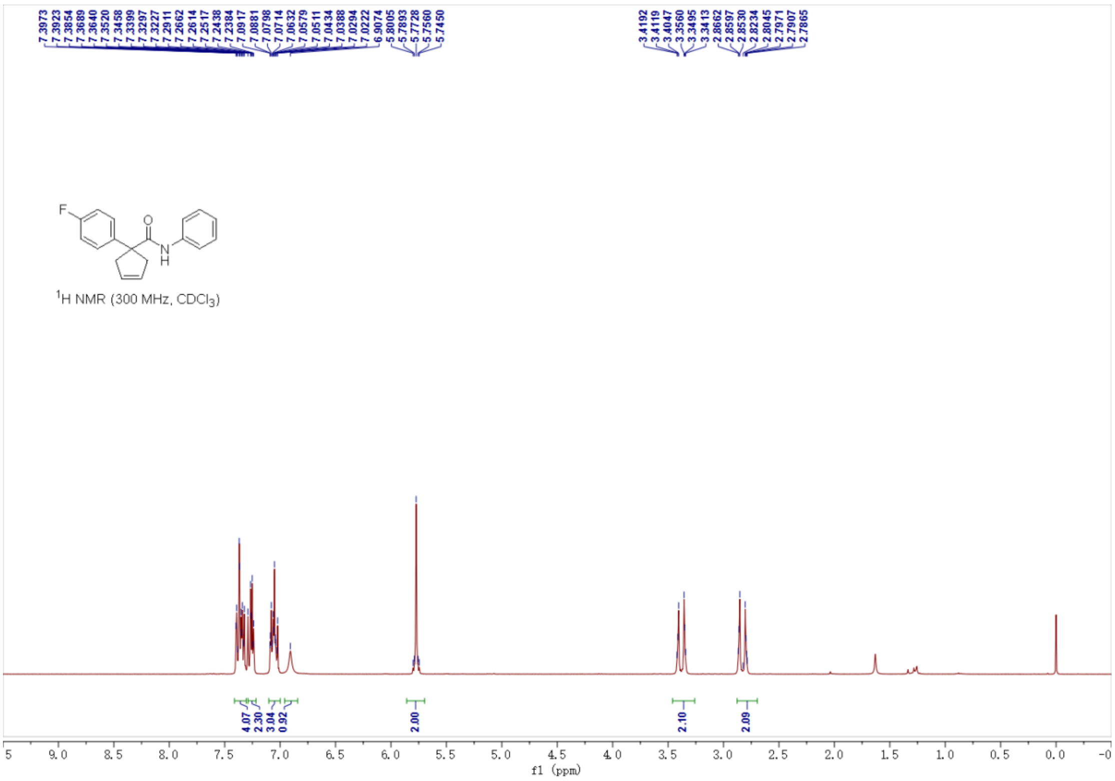

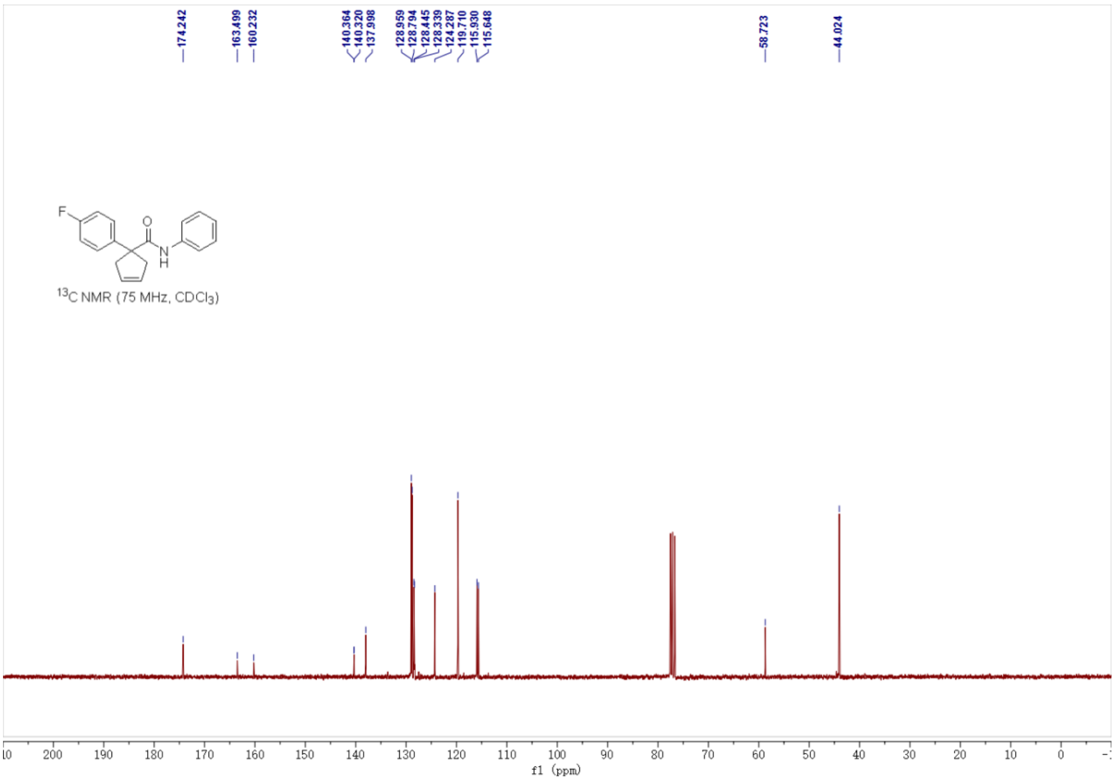

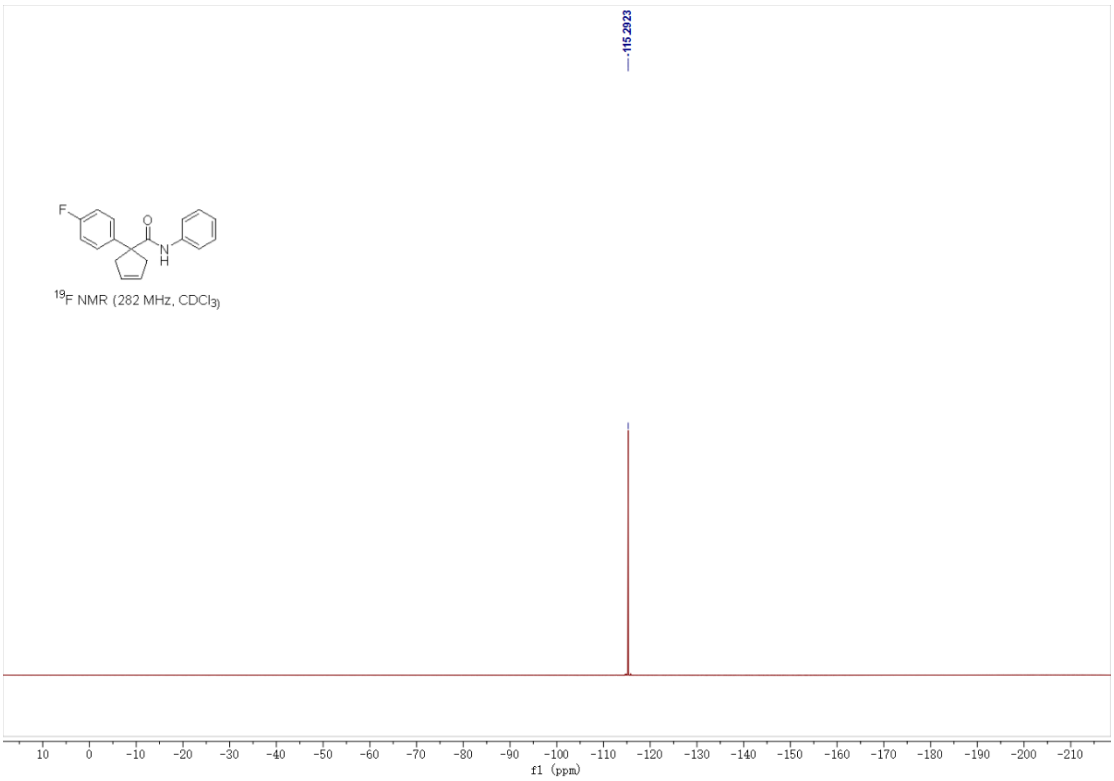

Supplementary Figure 53. ^{1}H , ^{13}C and ^{19}F NMR spectra of **1e**

1-(4-bromophenyl)-N-phenylcyclopent-3-ene-1-carboxamide (1f)

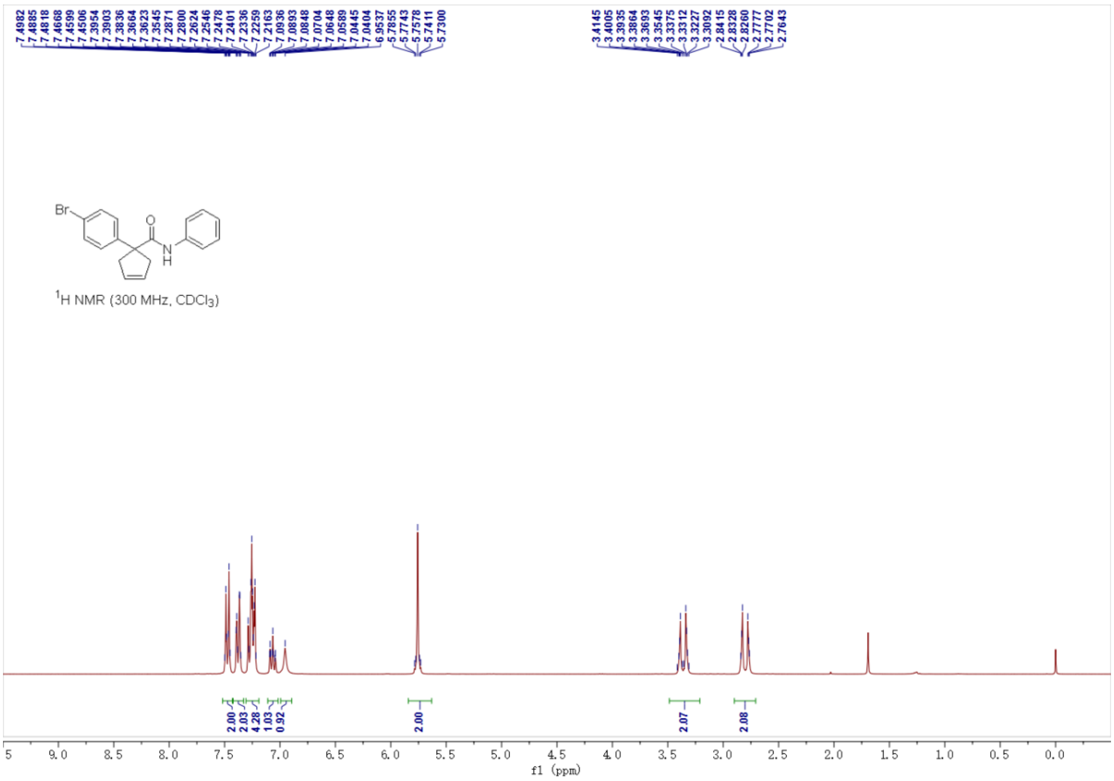

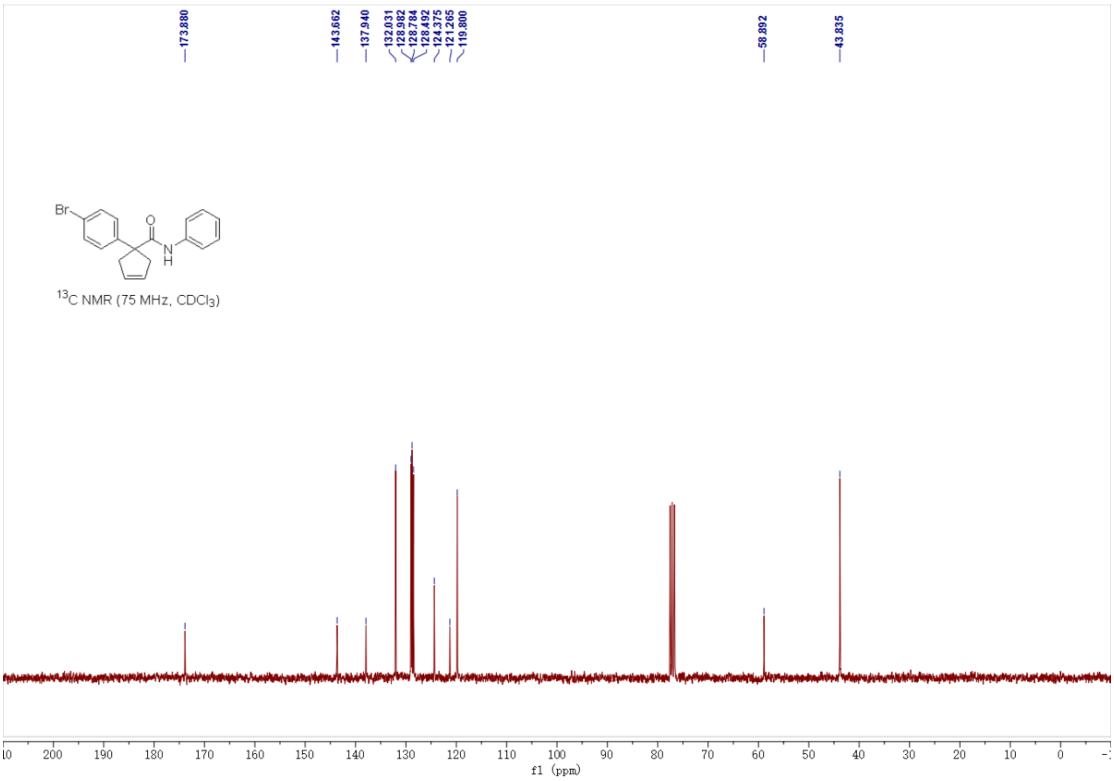

Supplementary Figure 54. ^{1}H and ^{13}C NMR spectra of **1f**

N-phenyl-1-(4-(trifluoromethyl)phenyl)cyclopent-3-ene-1-carboxamide (1g)

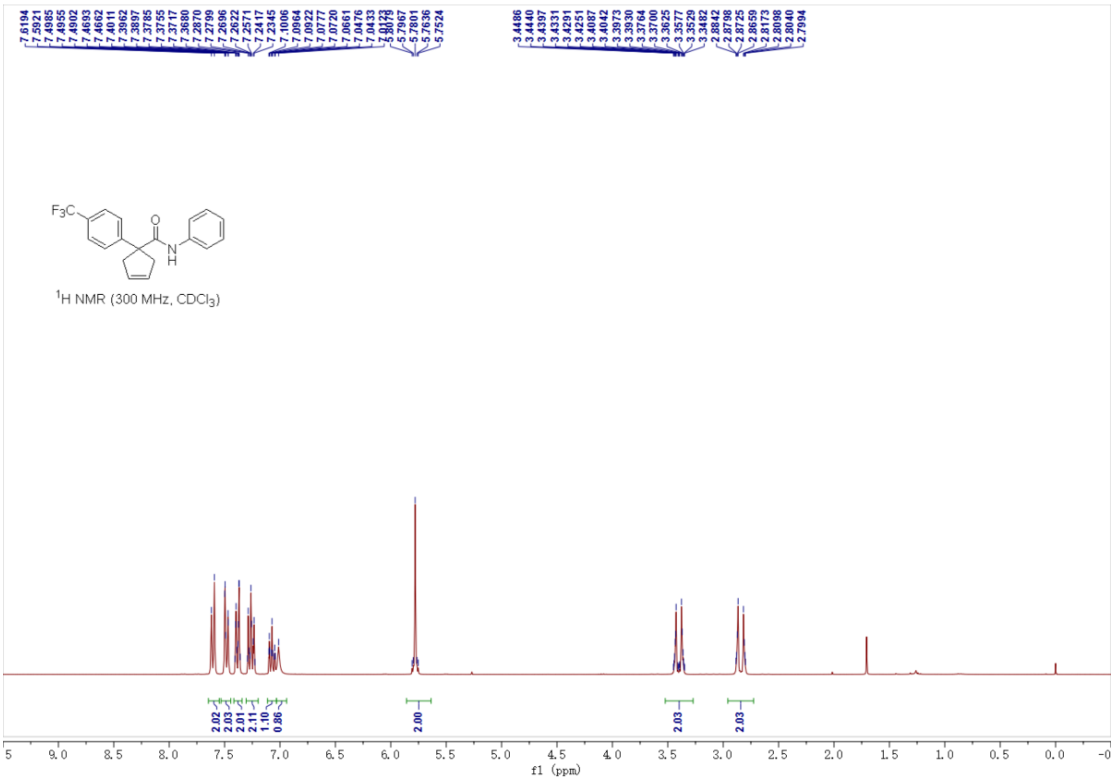

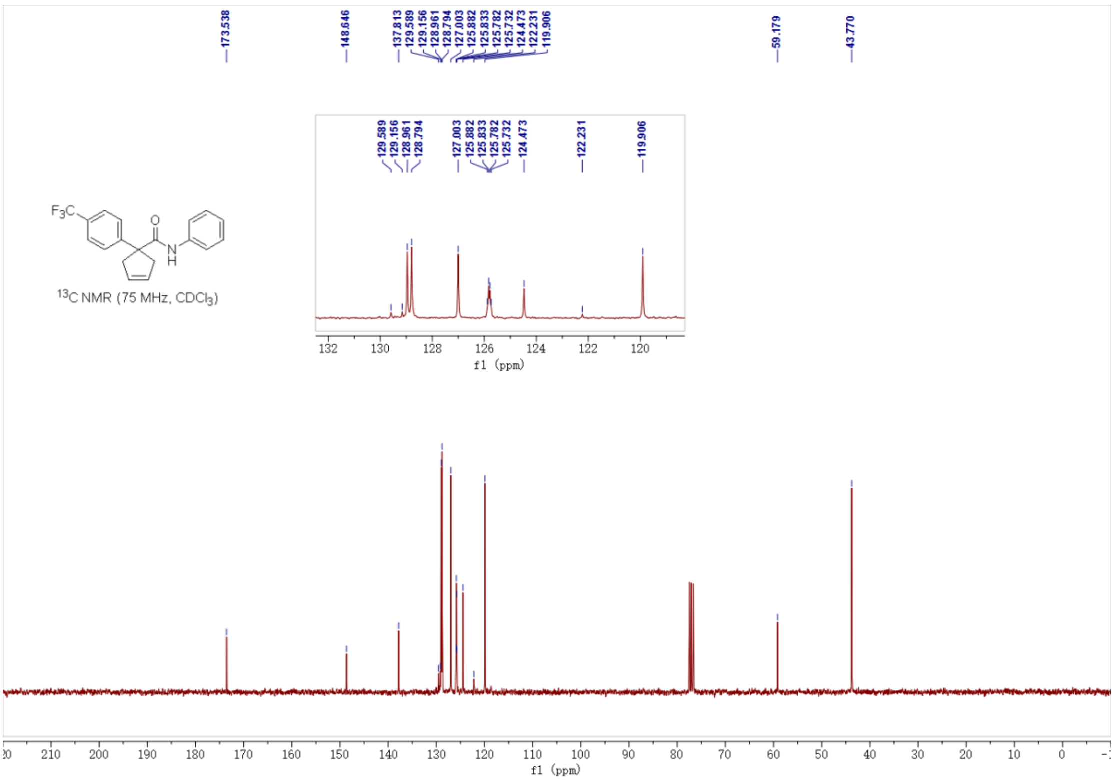

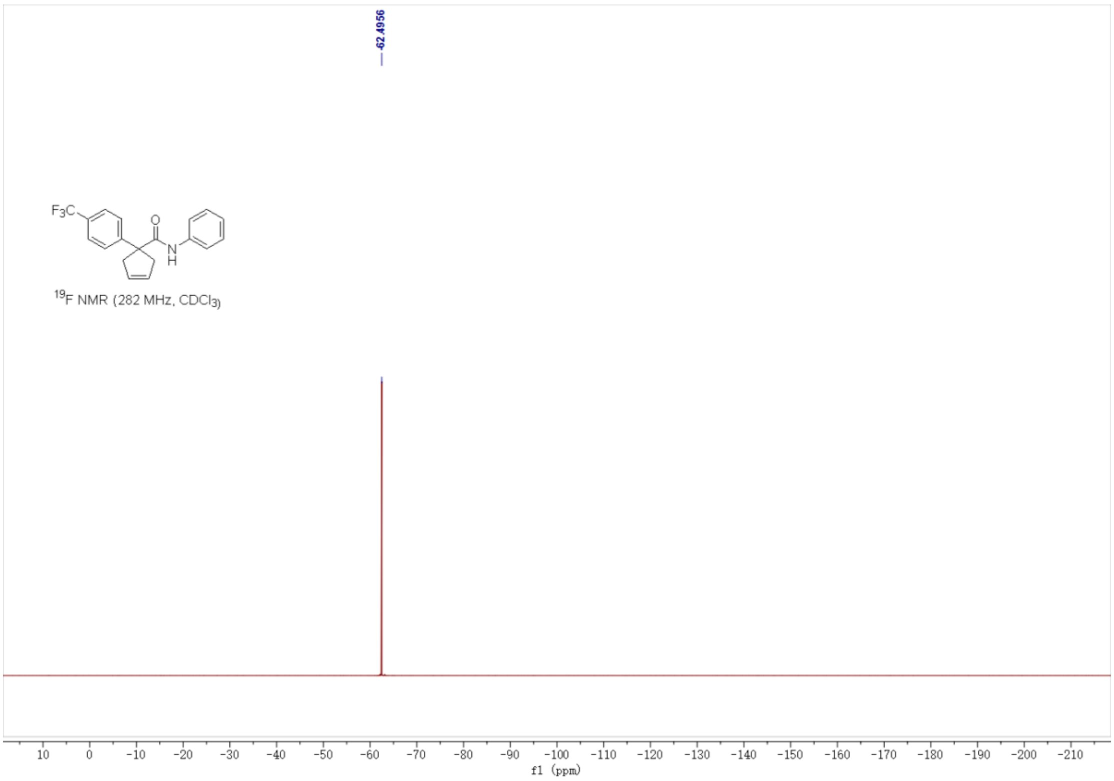

Supplementary Figure 55. ^{1}H , ^{13}C and ^{19}F NMR spectra of **1g**

1-(2-chlorophenyl)-N-phenylcyclopent-3-ene-1-carboxamide (1h)

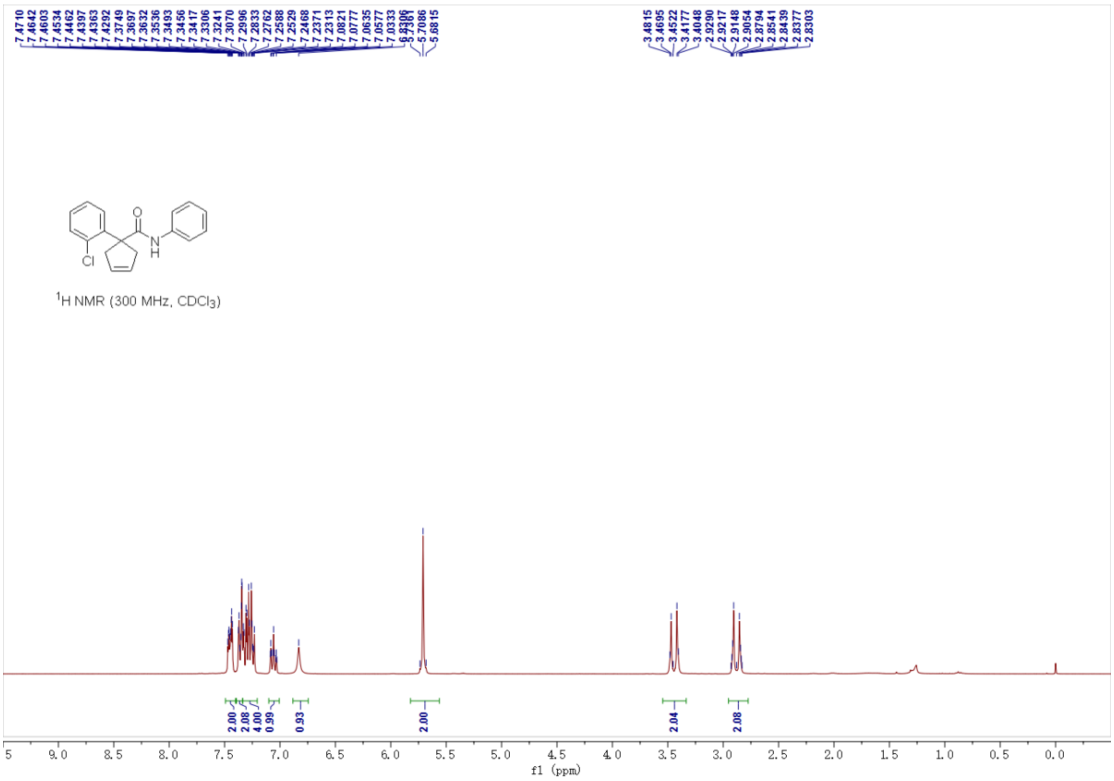

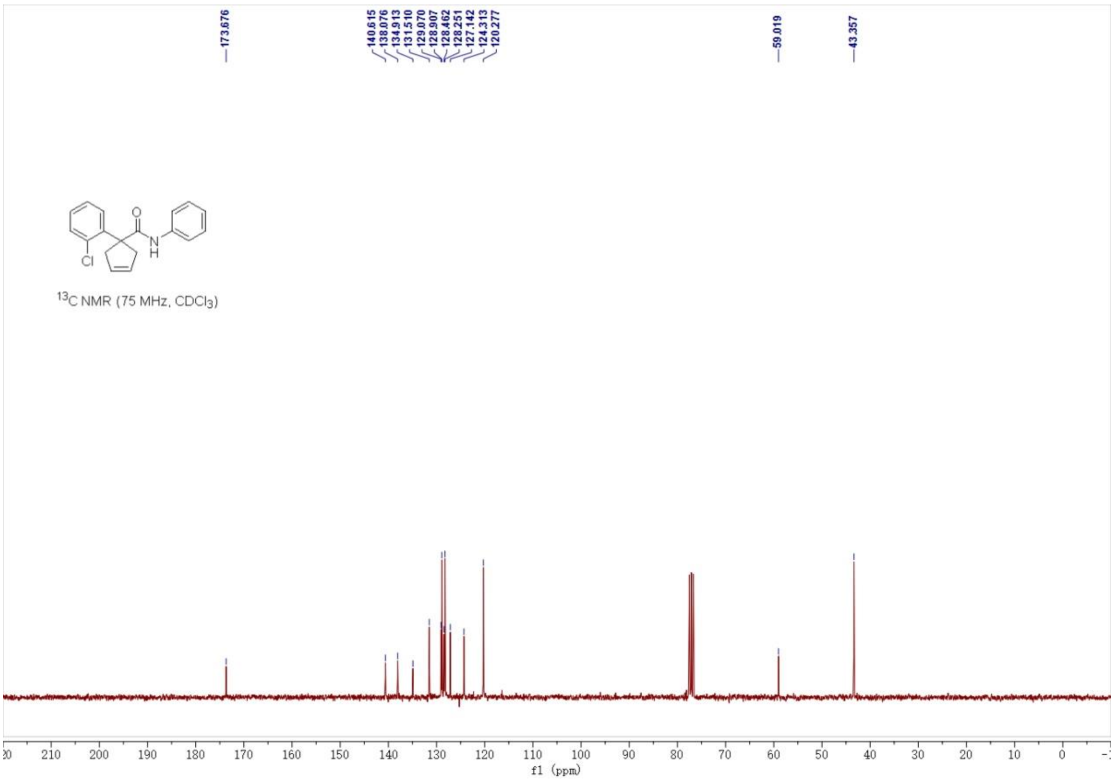

Supplementary Figure 56. ^{1}H and ^{13}C NMR spectra of **1h**

N-phenyl-1-(m-tolyl)cyclopent-3-ene-1-carboxamide (1i)

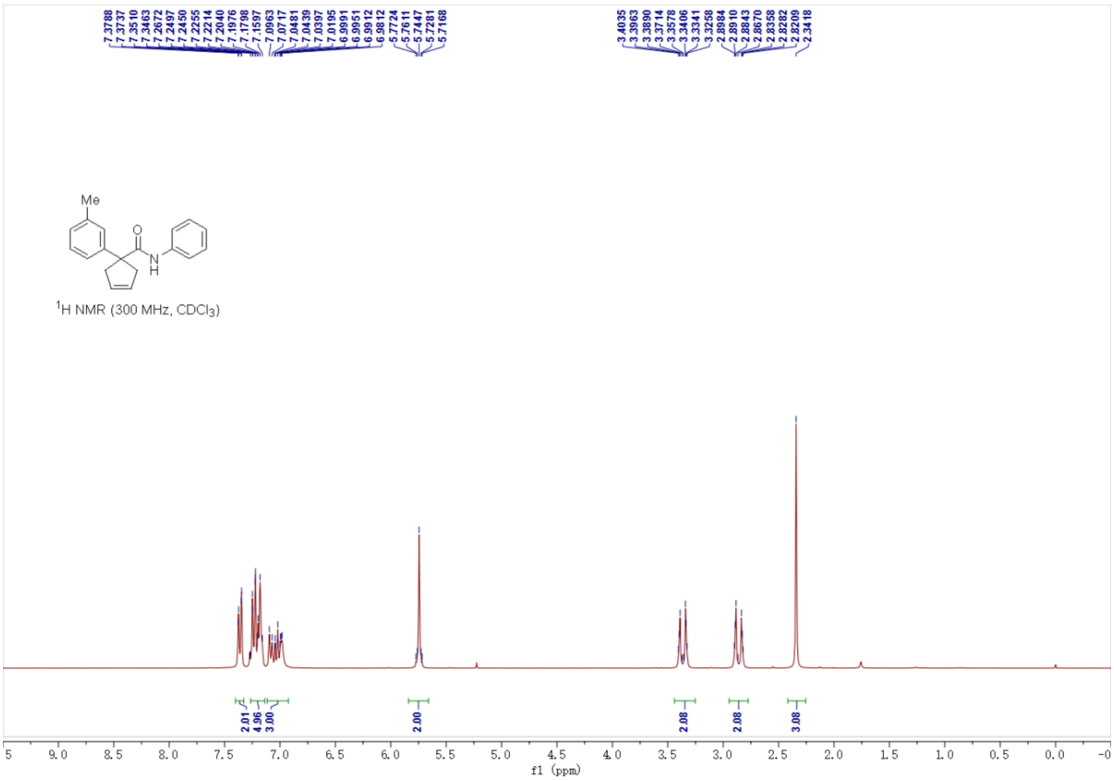

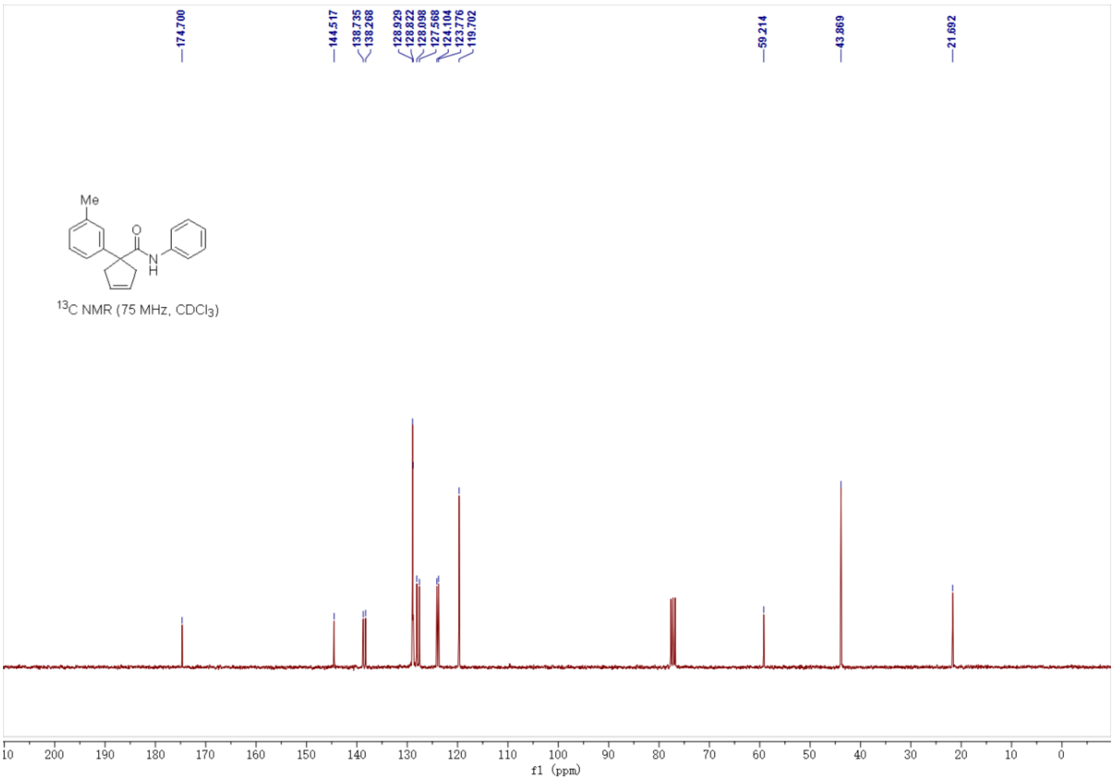

Supplementary Figure 57. ^{1}H and ^{13}C NMR spectra of **1i**

N-phenyl-1-(thiophen-2-yl)cyclopent-3-ene-1-carboxamide (1j)

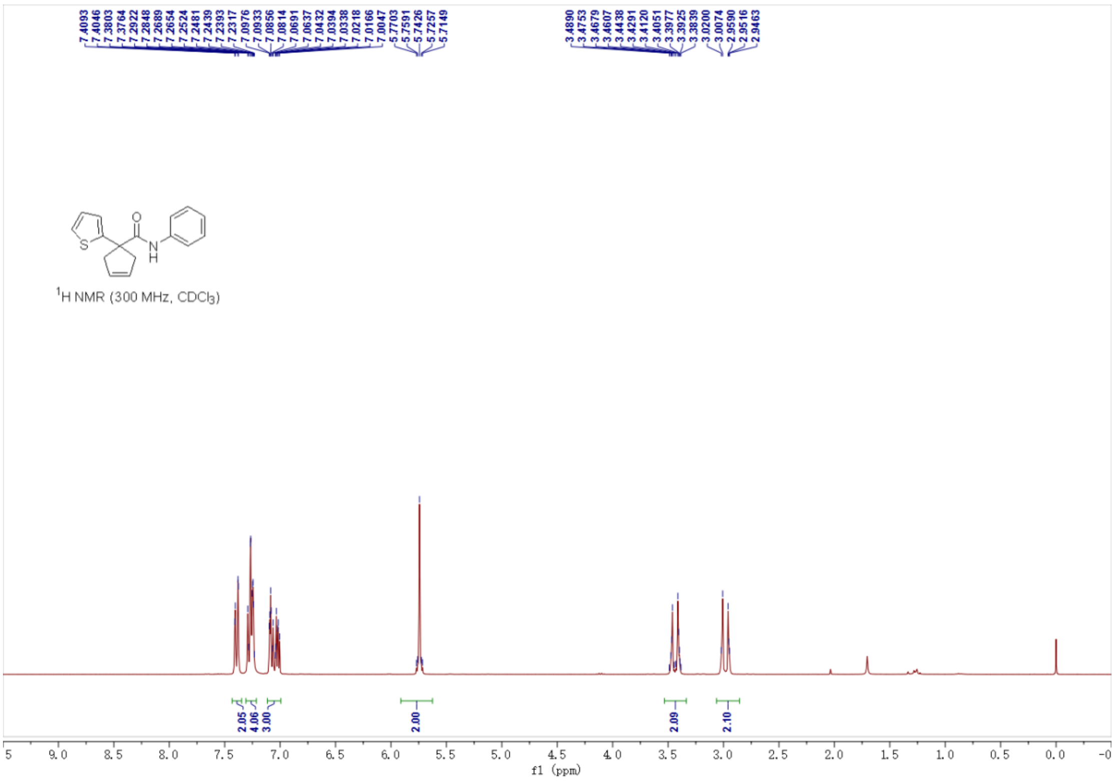

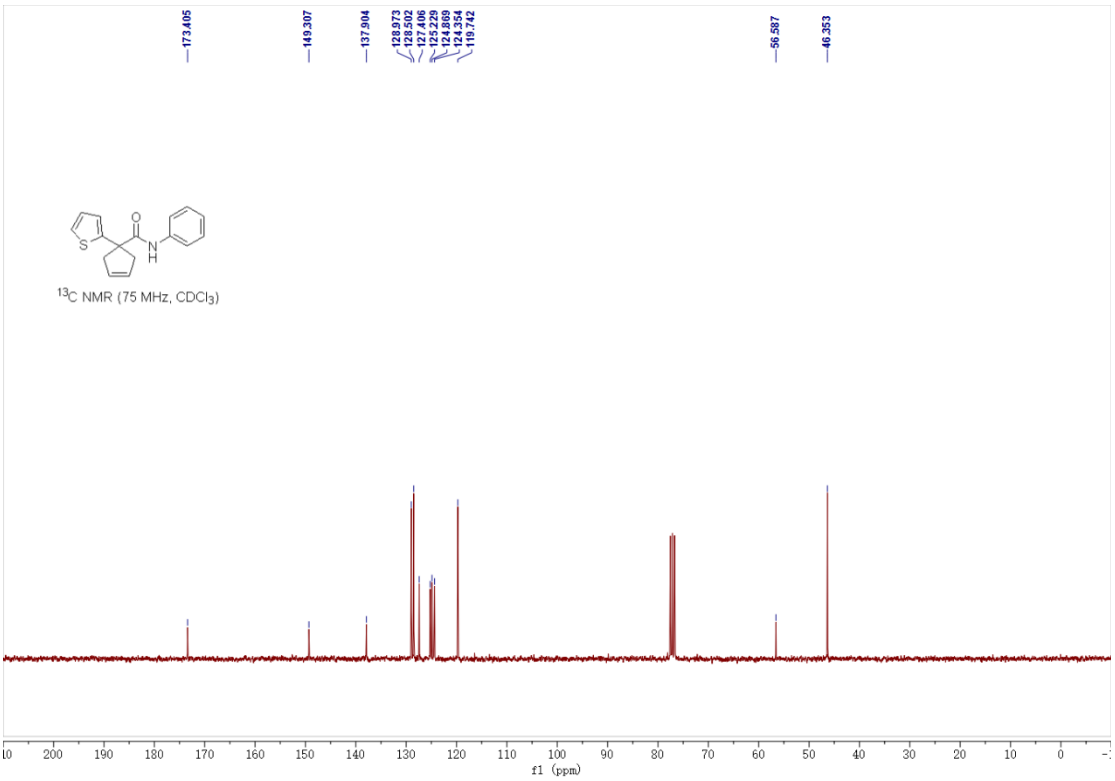

Supplementary Figure 58. ^{1}H and ^{13}C NMR spectra of **1j**

1-(furan-3-ylmethyl)-N-phenylcyclopent-3-ene-1-carboxamide (1k)

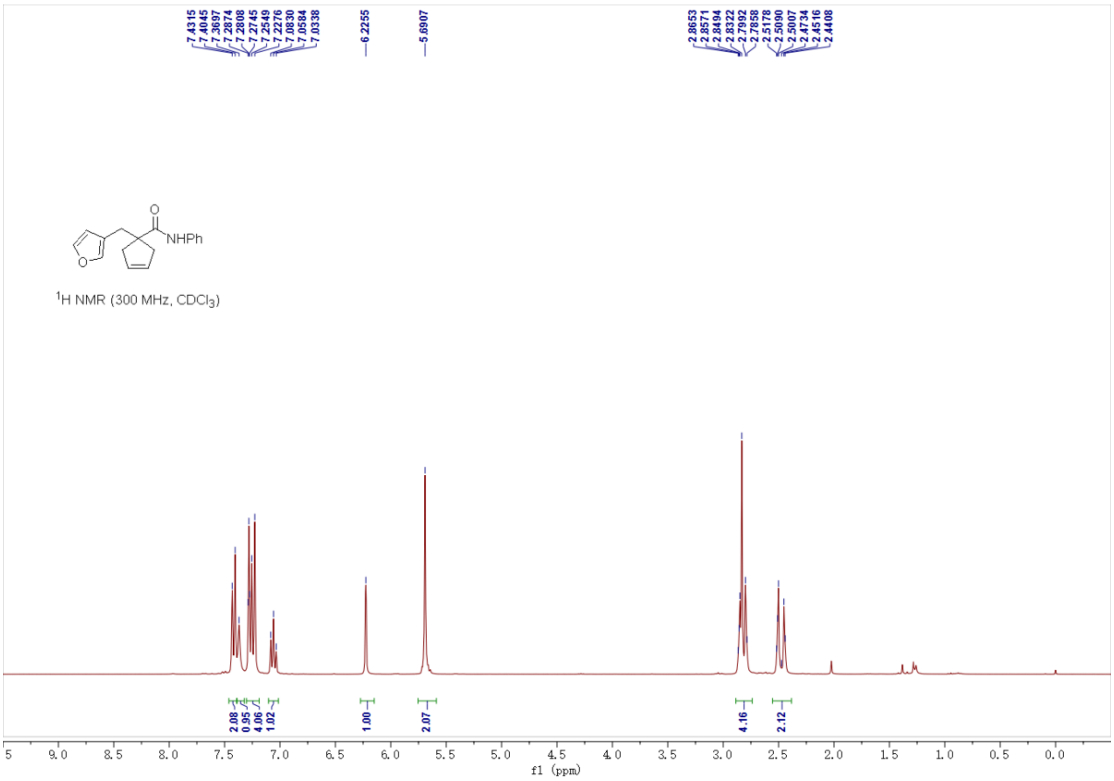

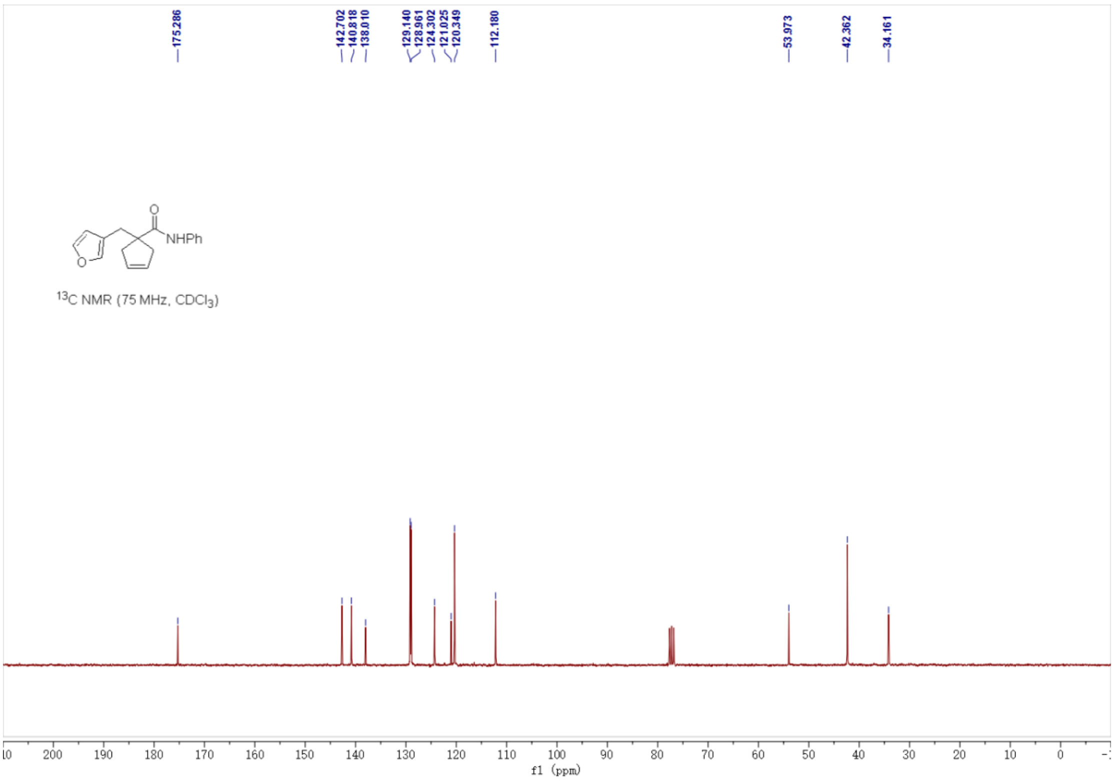

Supplementary Figure 59. ^{1}H and ^{13}C NMR spectra of **1k**

1-benzyl-N-phenylcyclopent-3-ene-1-carboxamide (1l)

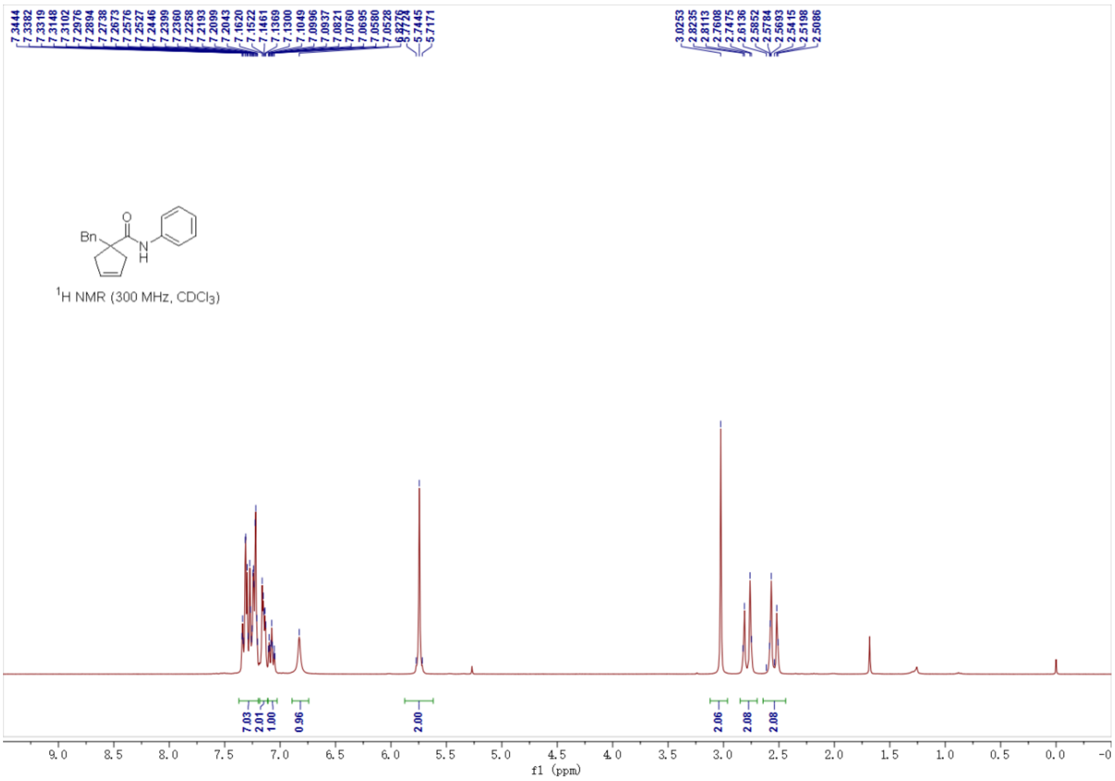

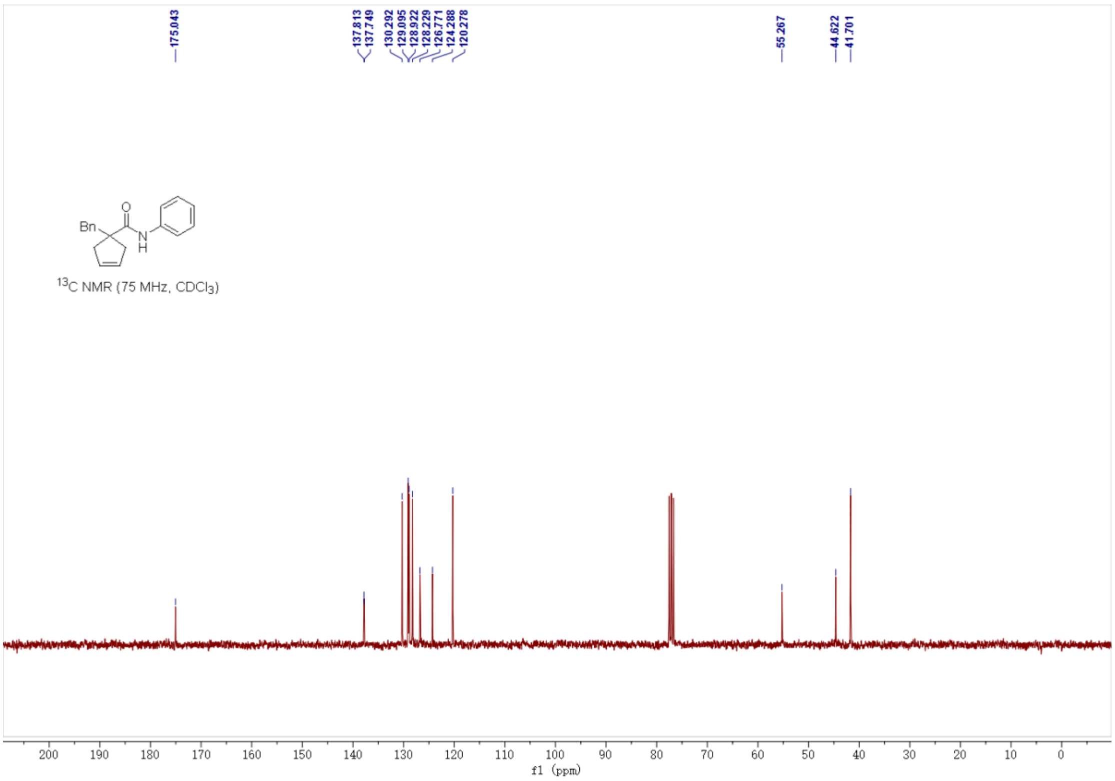

Supplementary Figure 60. ^{1}H and ^{13}C NMR spectra of **1l**

***tert*-butyl (1-(phenylcarbamoyl)cyclopent-3-en-1-yl)carbamate (1o)**

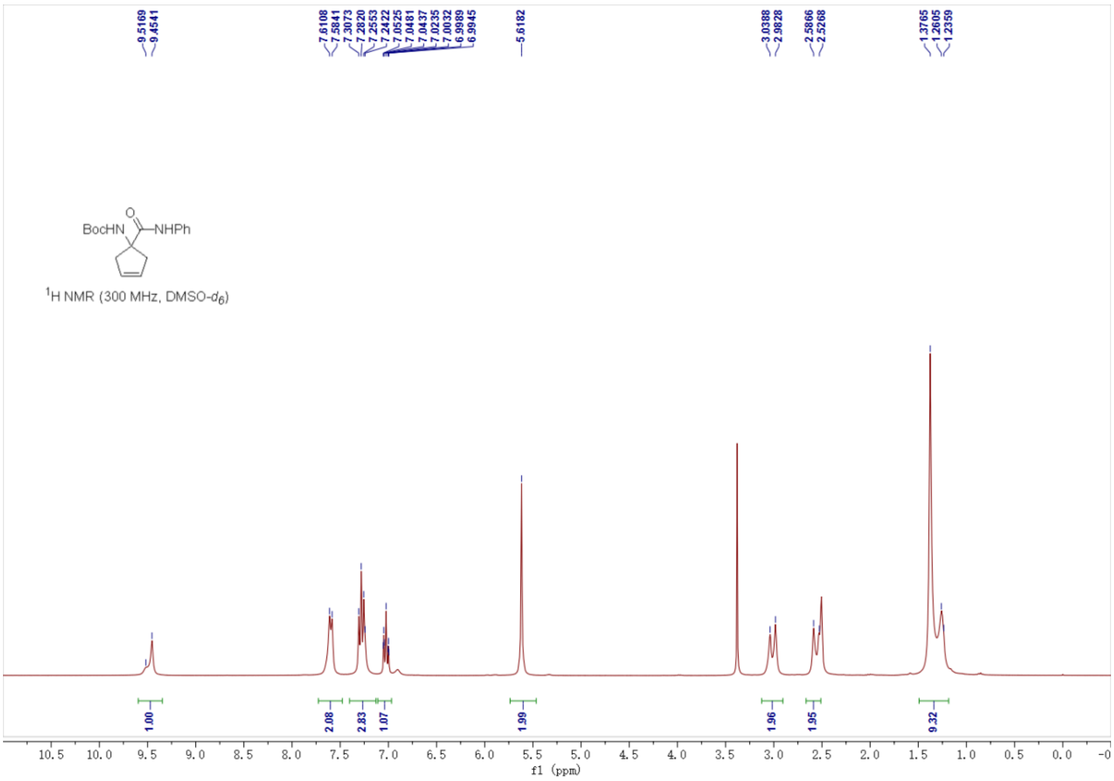

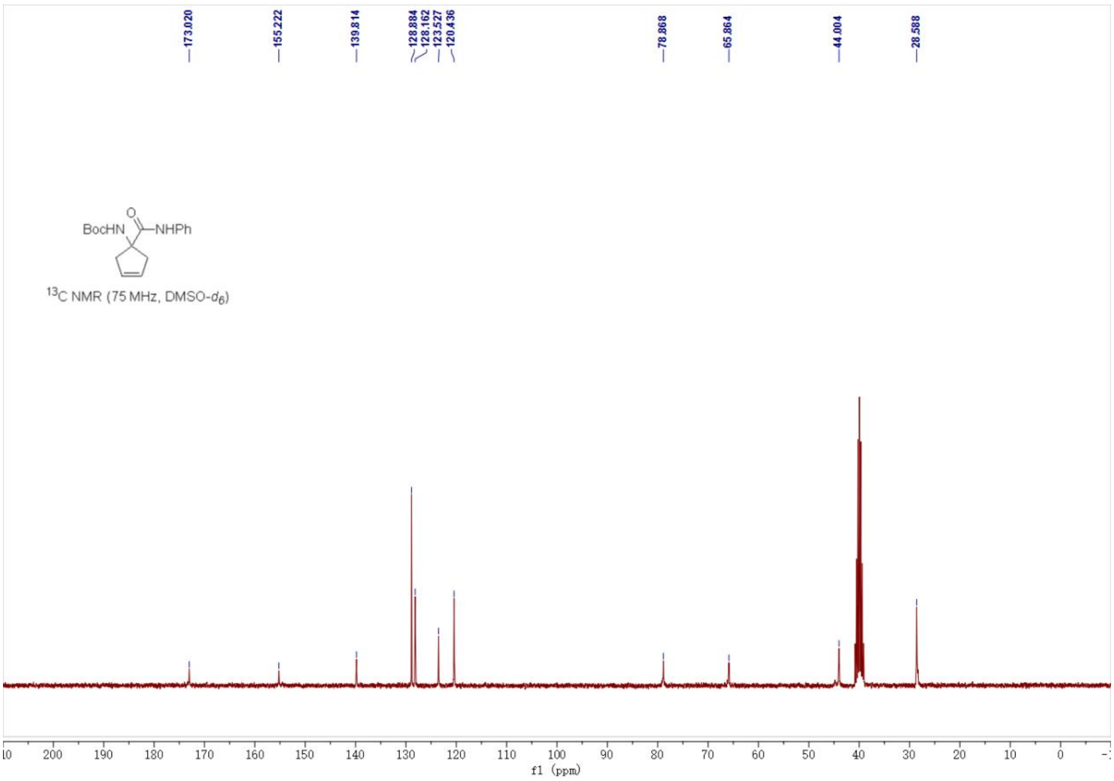

Supplementary Figure 61. ^{1}H and ^{13}C NMR spectra of **1o**

1-phenyl-N-(p-tolyl)cyclopent-3-ene-1-carboxamide (1q)

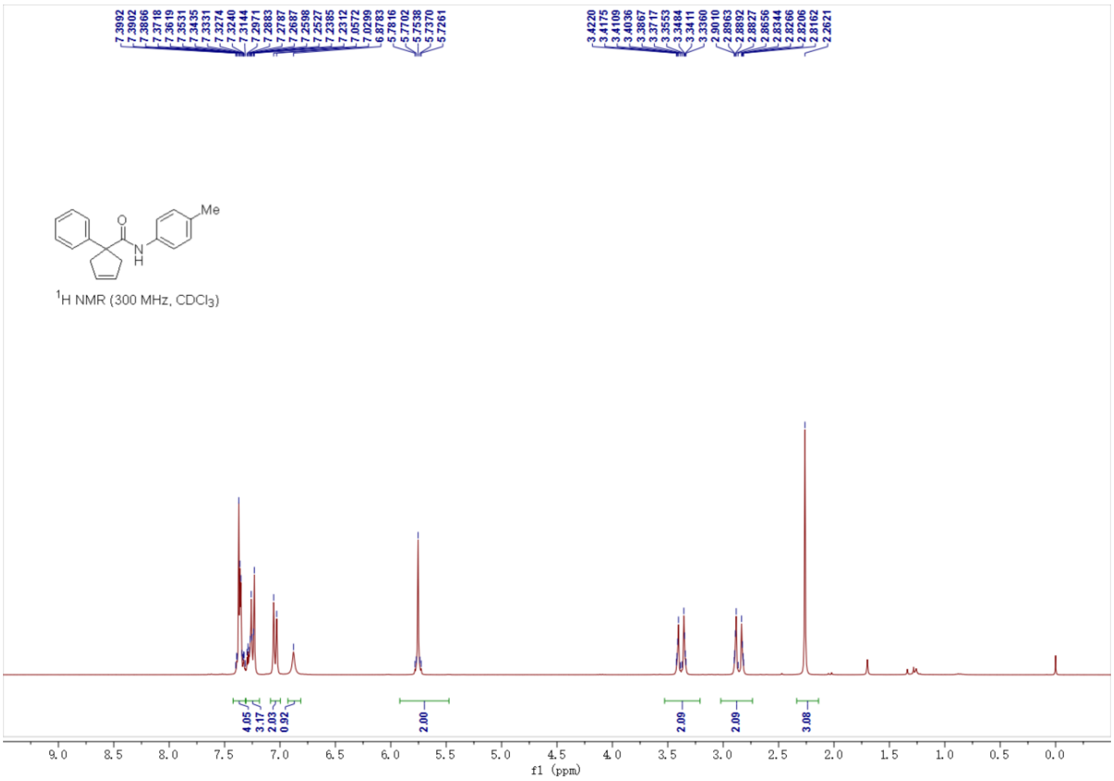

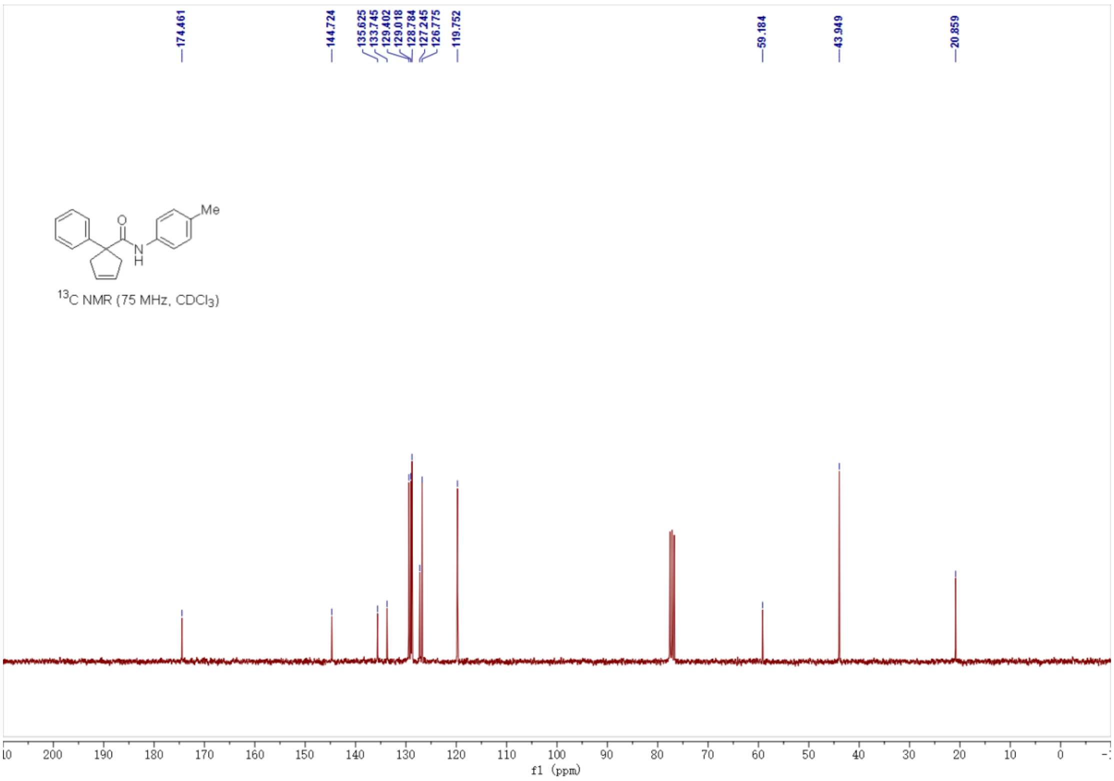

Supplementary Figure 62. ^{1}H and ^{13}C NMR spectra of **1q**

***N*-(4-methoxyphenyl)-1-phenylcyclopent-3-ene-1-carboxamide (1r)**

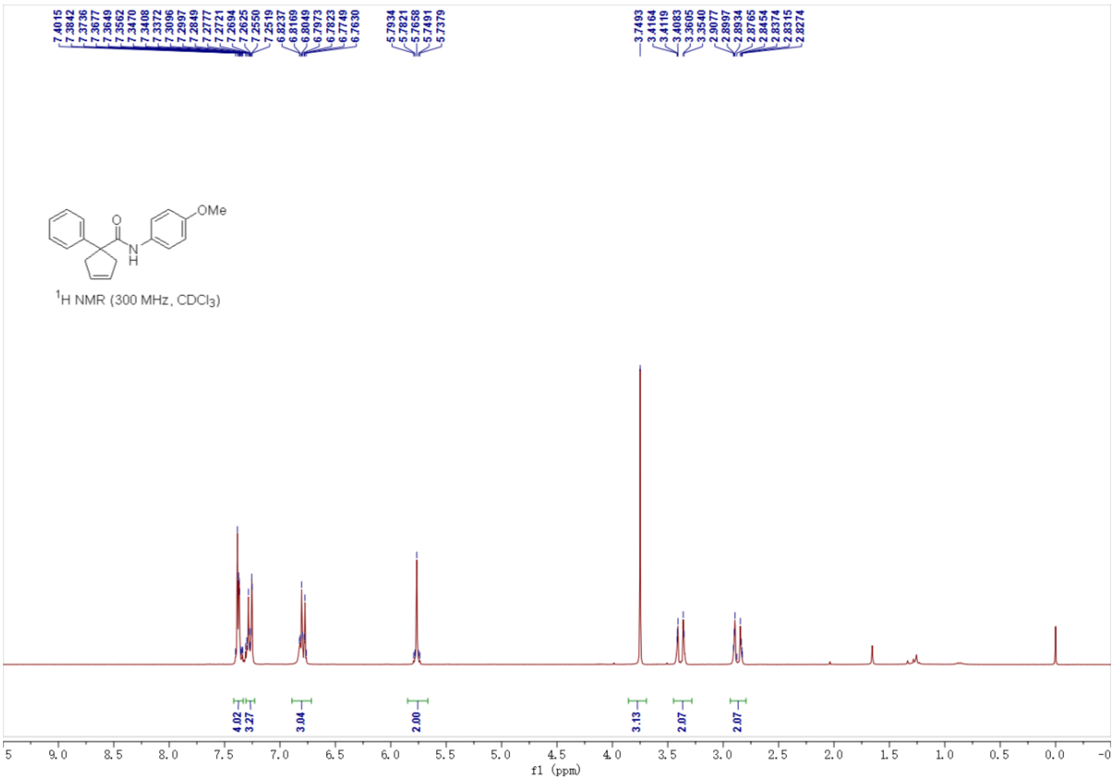

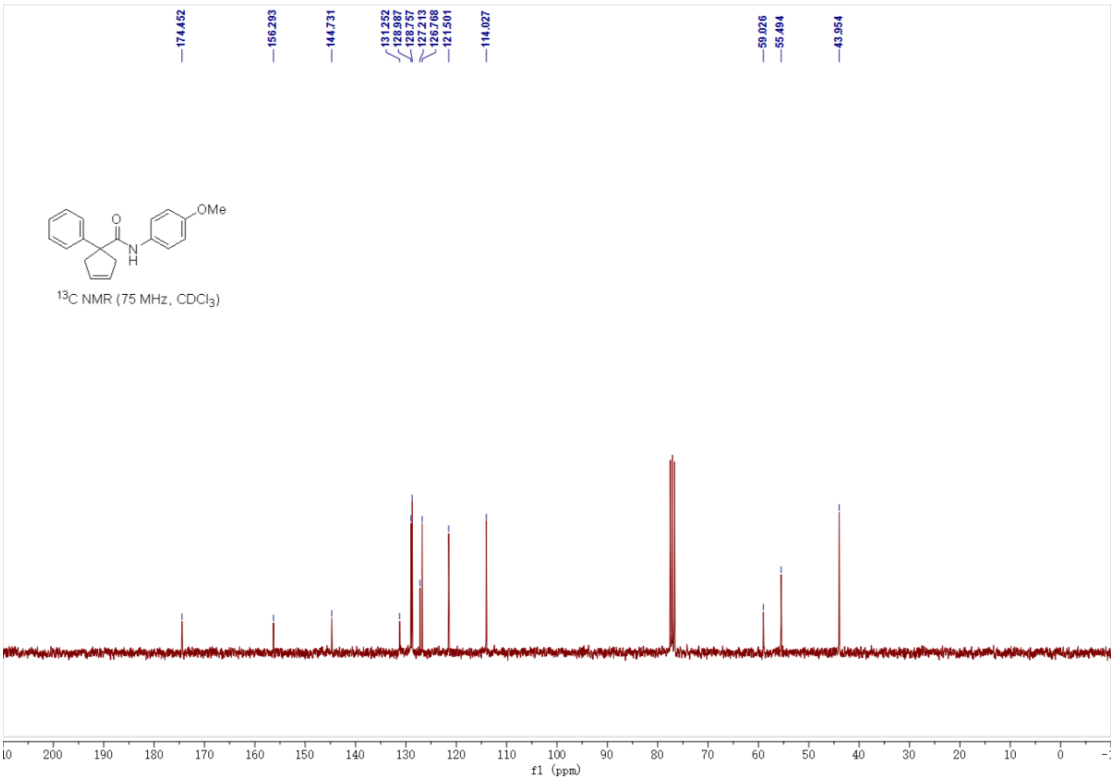

Supplementary Figure 63. ^{1}H and ^{13}C NMR spectra of **1r**

N-(4-methoxyphenyl)-1-phenylcyclopent-3-ene-1-carboxamide (1s)

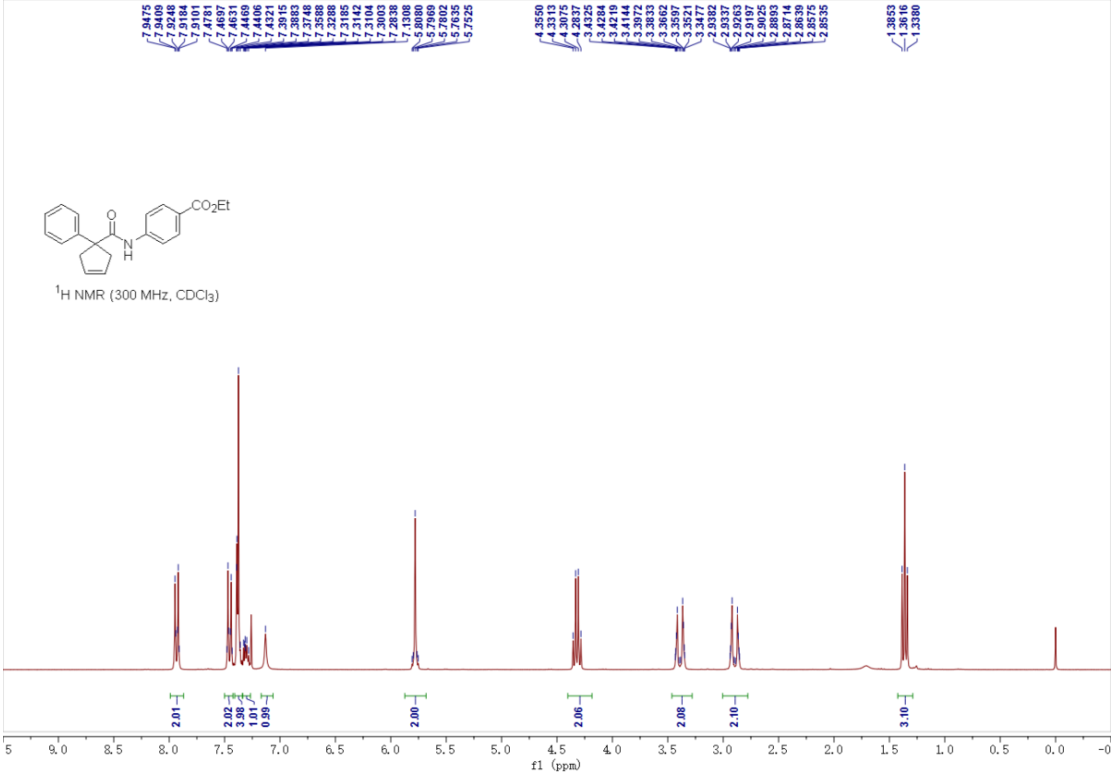

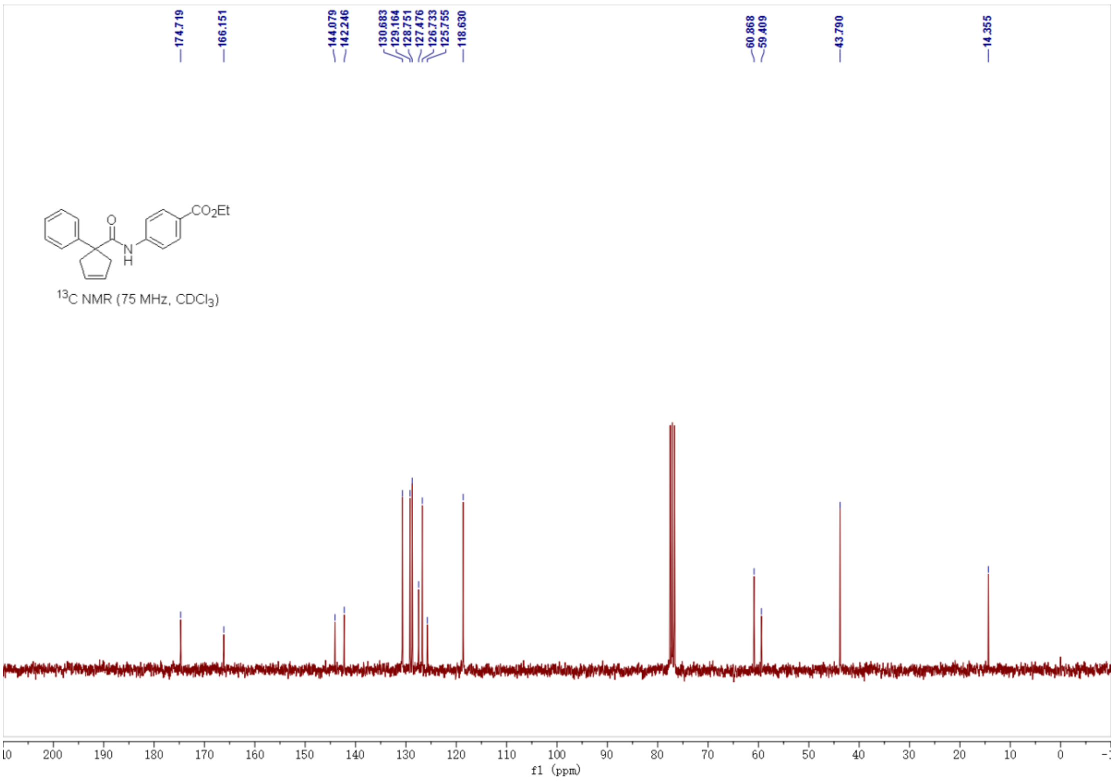

Supplementary Figure 64. ^{1}H and ^{13}C NMR spectra of **1s**

N-ethyl-1-phenylcyclopent-3-ene-1-carboxamide (1t)

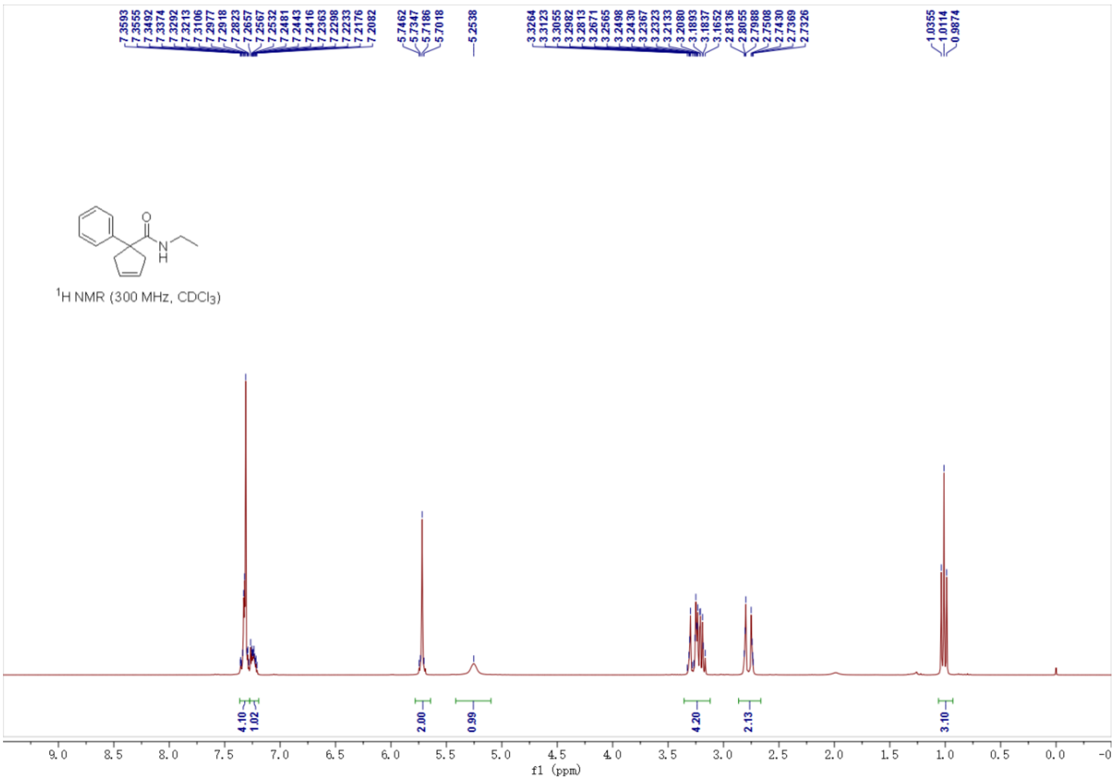

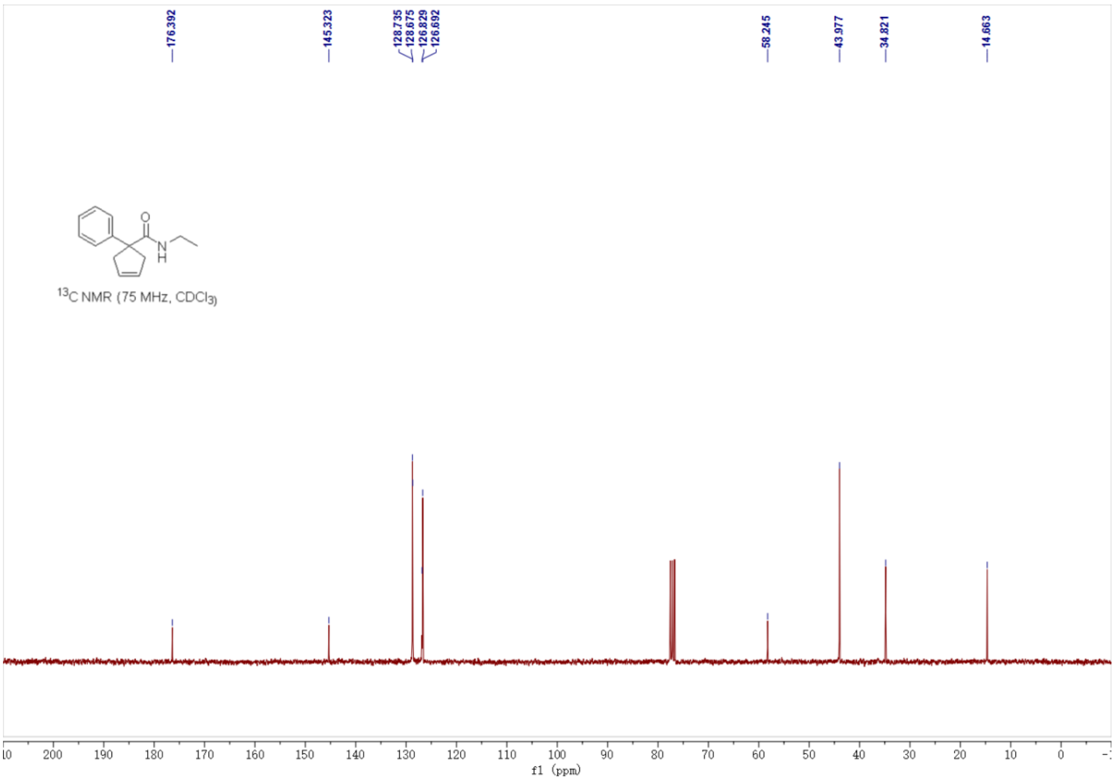

Supplementary Figure 65. ^{1}H and ^{13}C NMR spectra of **1t**

N-cyclohexyl-1-phenylcyclopent-3-ene-1-carboxamide (1v)

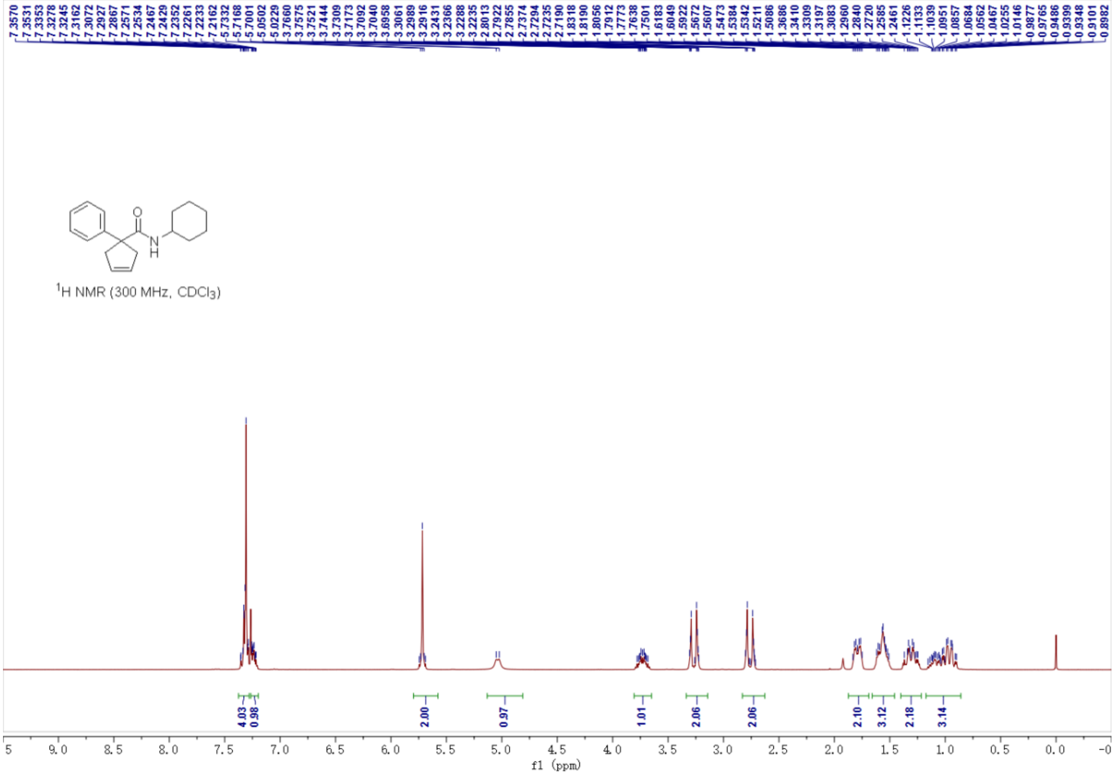

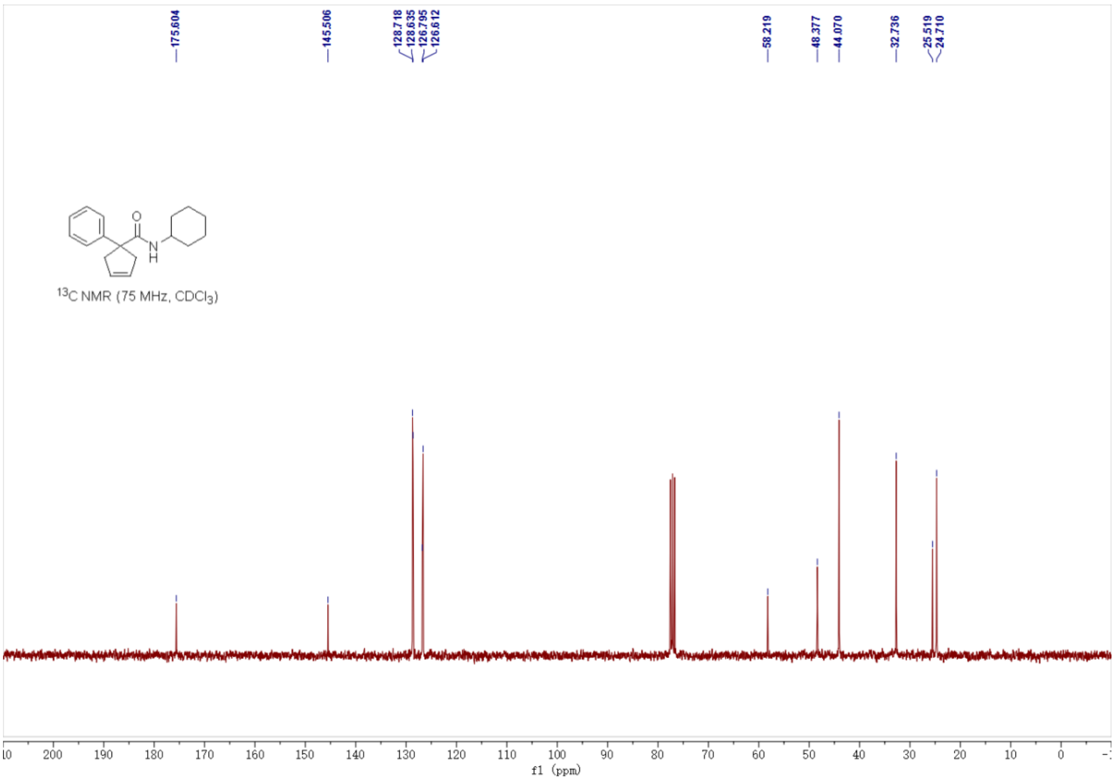

Supplementary Figure 66. ^{1}H and ^{13}C NMR spectra of **1v**

N-(tert-butyl)-1-phenylcyclopent-3-ene-1-carboxamide (1w)

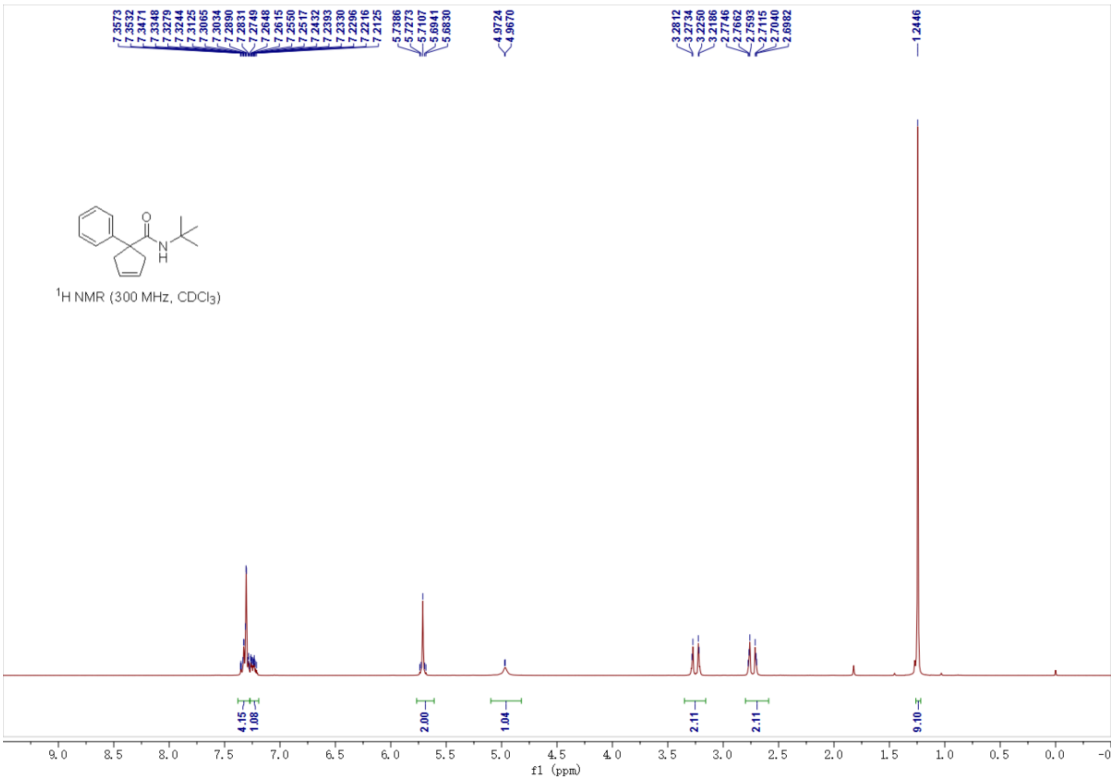

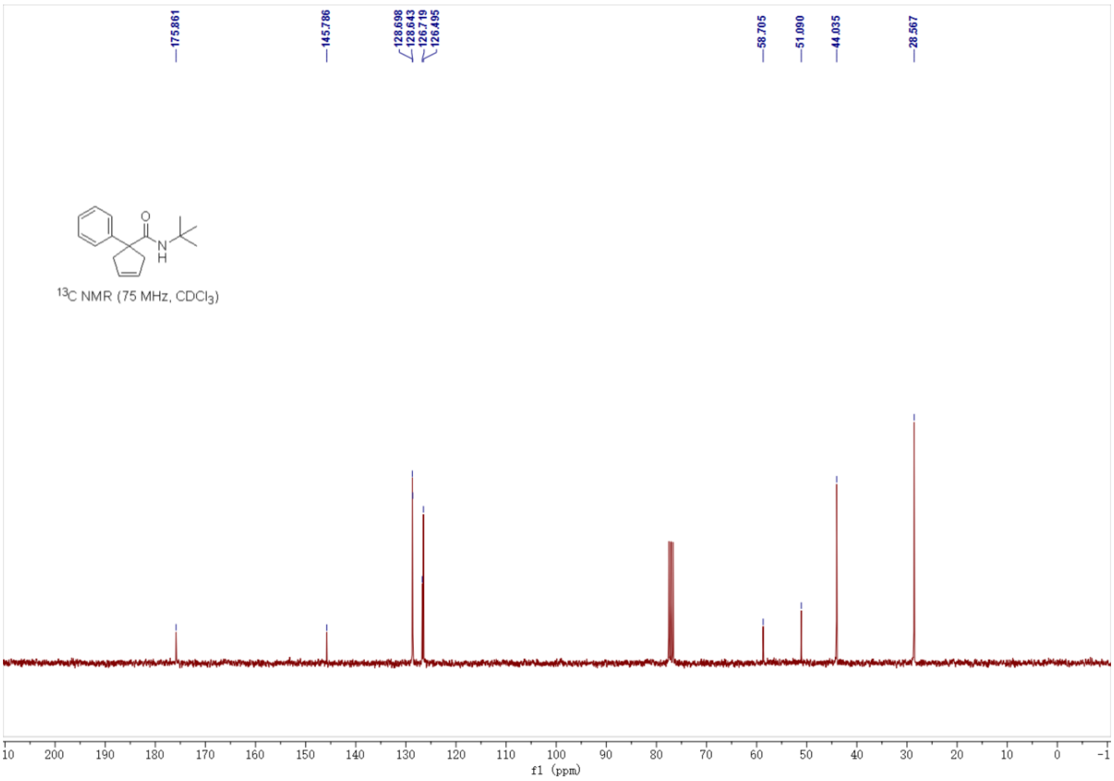

Supplementary Figure 67. ^{1}H and ^{13}C NMR spectra of **1w**

N-methyl-N,1-diphenylcyclopent-3-ene-1-carboxamide (1x)

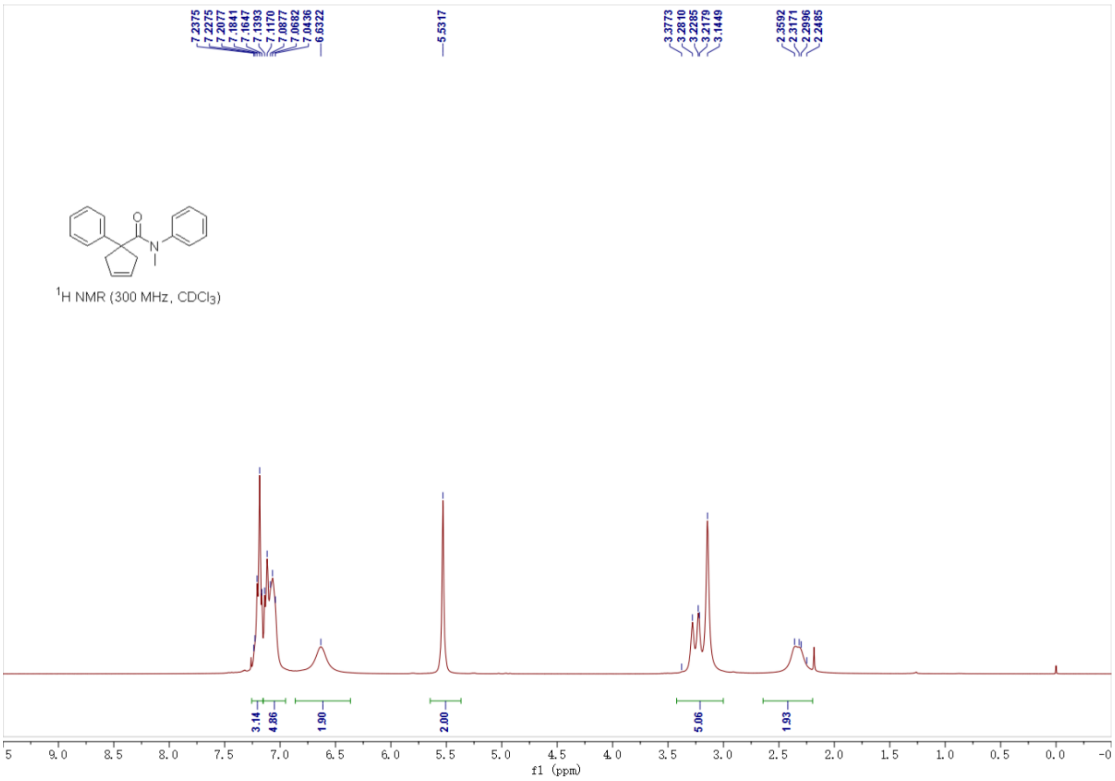

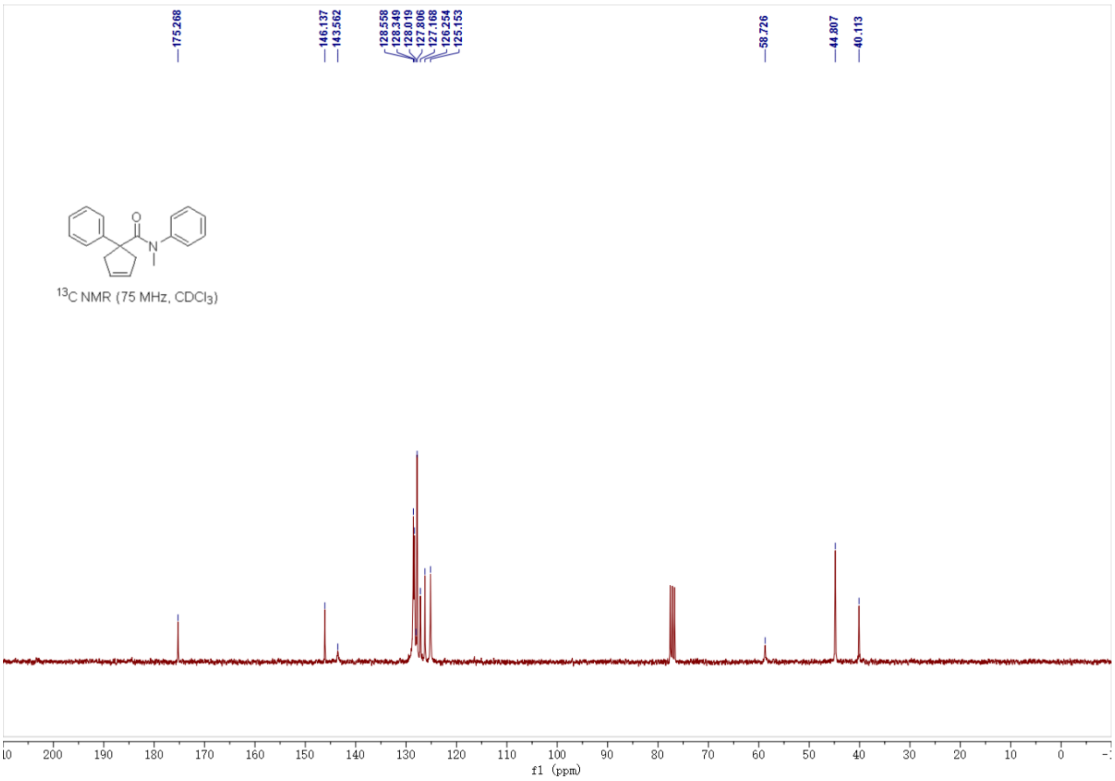

Supplementary Figure 68. ^{1}H and ^{13}C NMR spectra of **1x**

phenyl 1-phenylcyclopent-3-ene-1-carboxylate (1z)

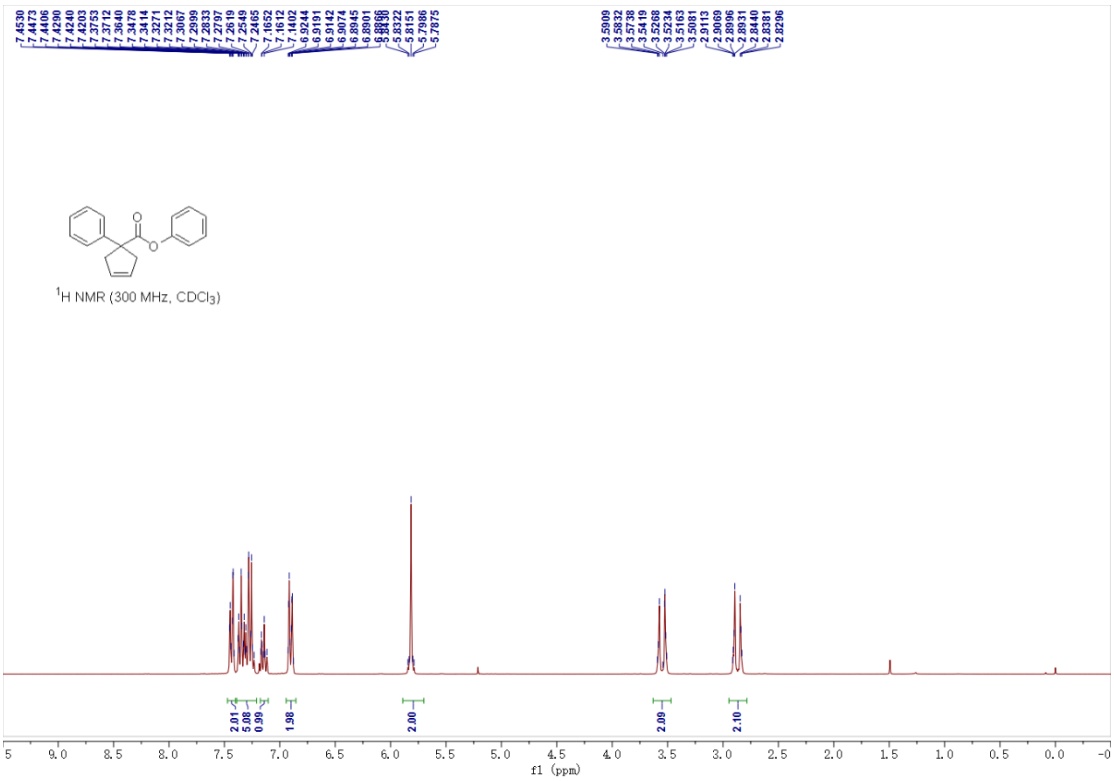

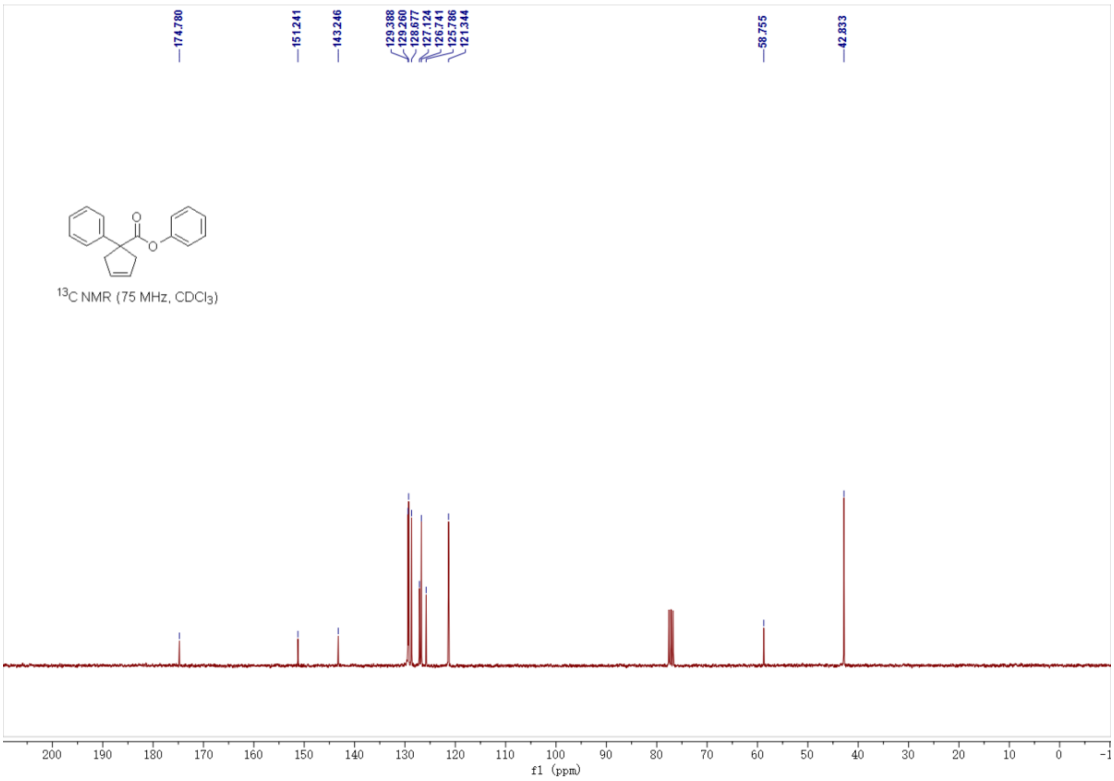

Supplementary Figure 69. ^{1}H and ^{13}C NMR spectra of **1z**

phenyl(1-phenylcyclopent-3-en-1-yl)methanone (1ab)

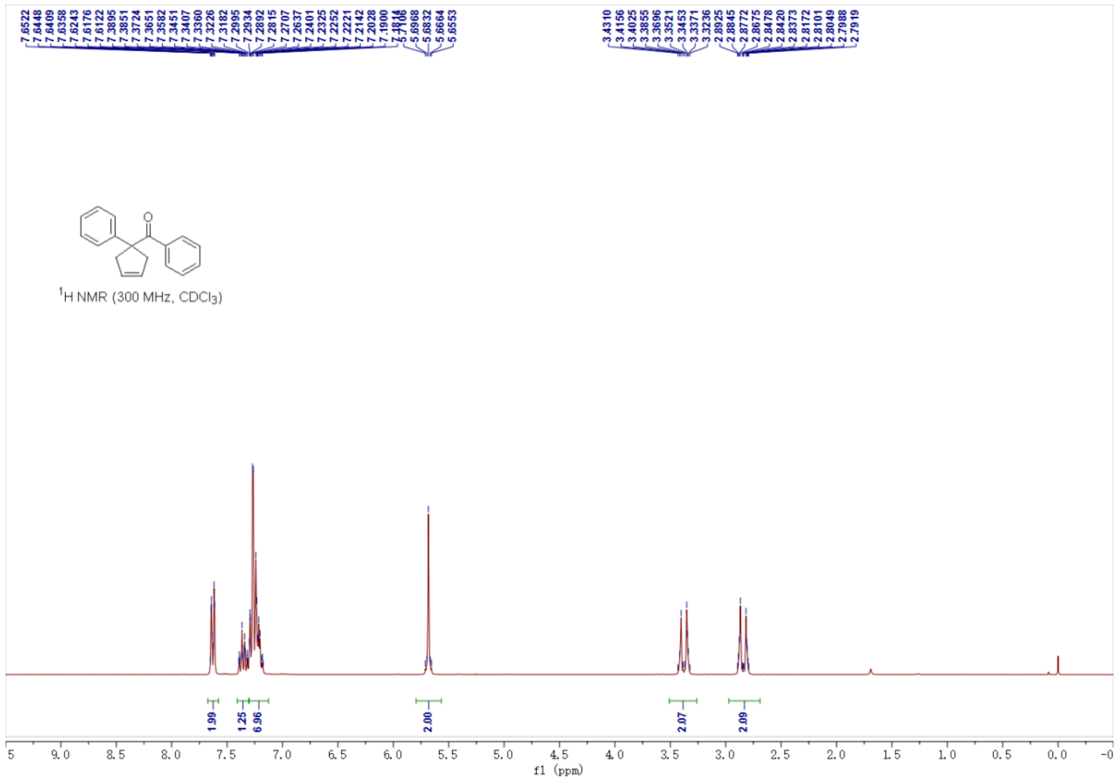

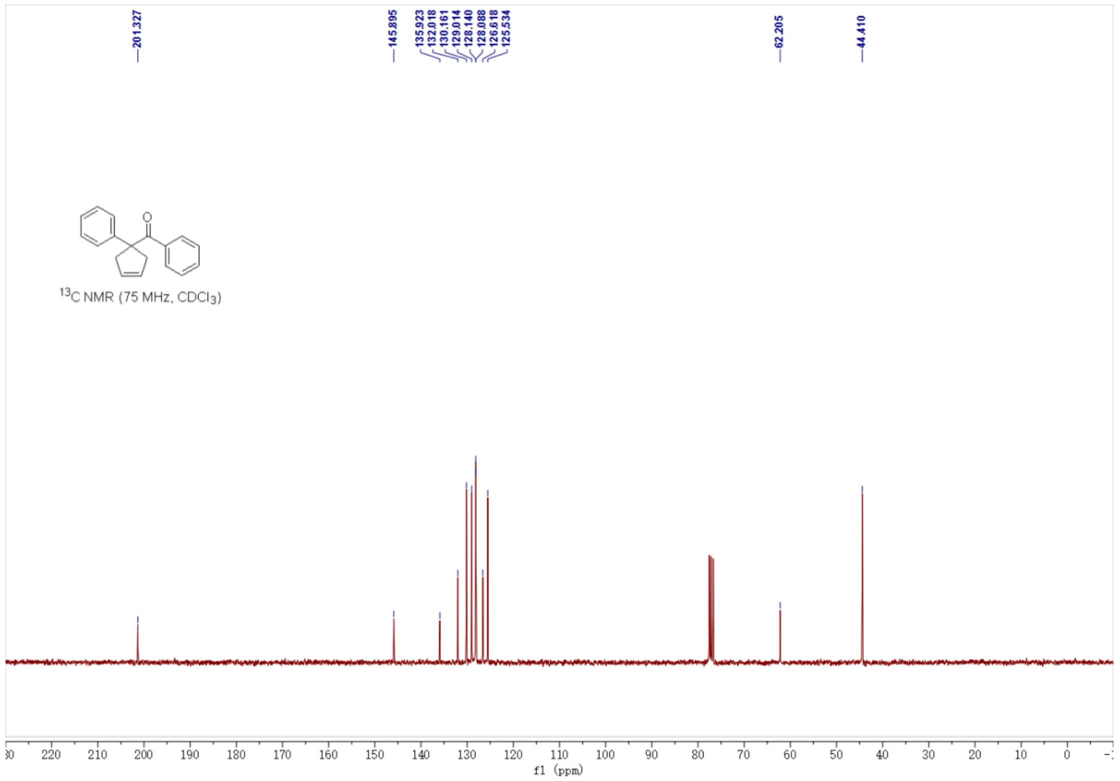

Supplementary Figure 70. ^{1}H and ^{13}C NMR spectra of **1ab**

2-methyl-1-(1-phenylcyclopent-3-en-1-yl)propan-1-one (1ac)

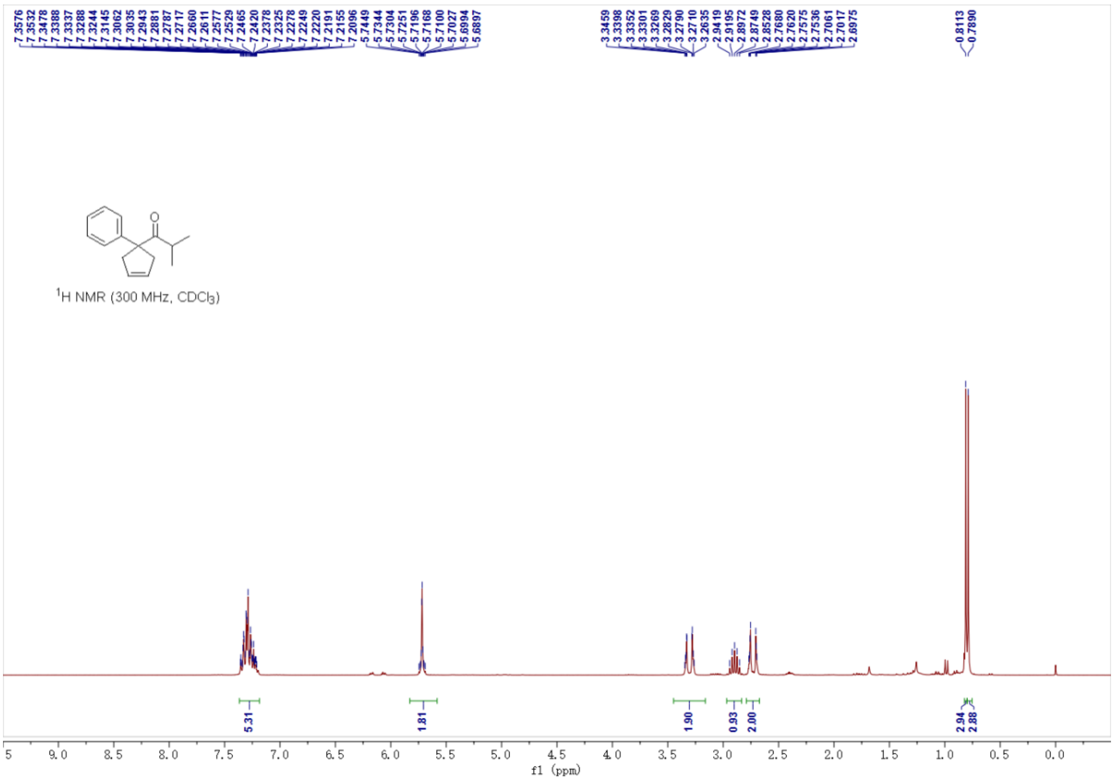

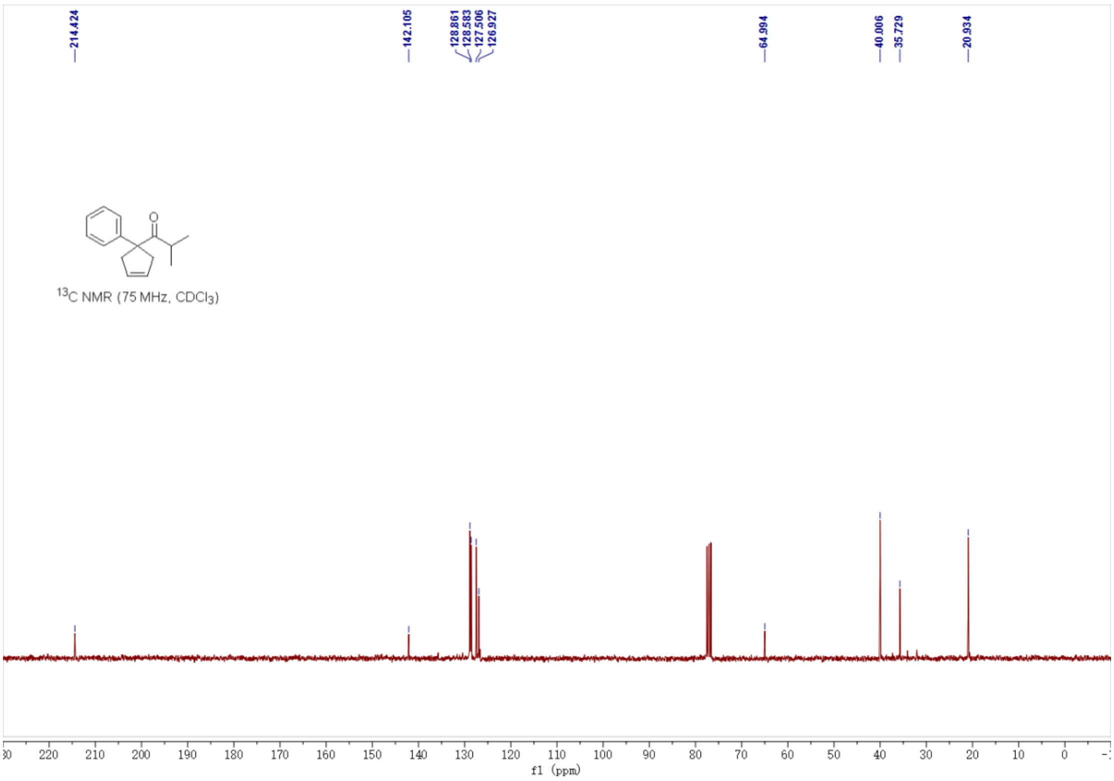

Supplementary Figure 71. ^{1}H and ^{13}C NMR spectra of **1ac**

1-(1-phenylcyclopent-3-en-1-yl)ethan-1-one (1ad)

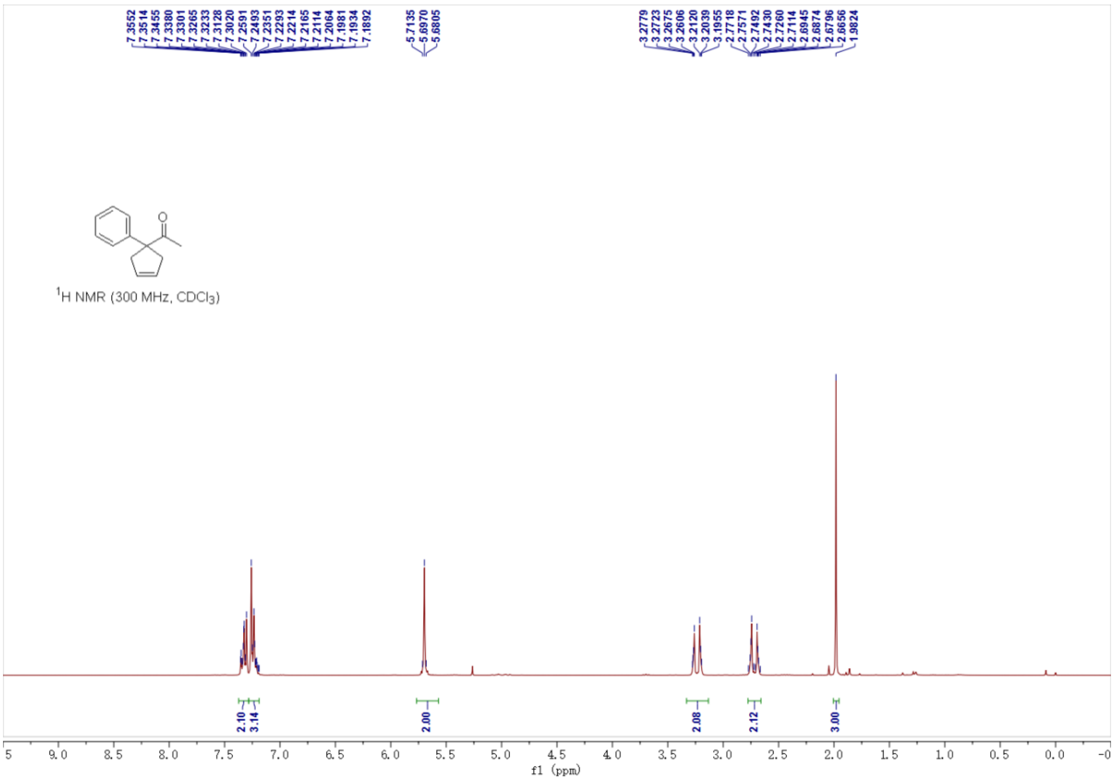

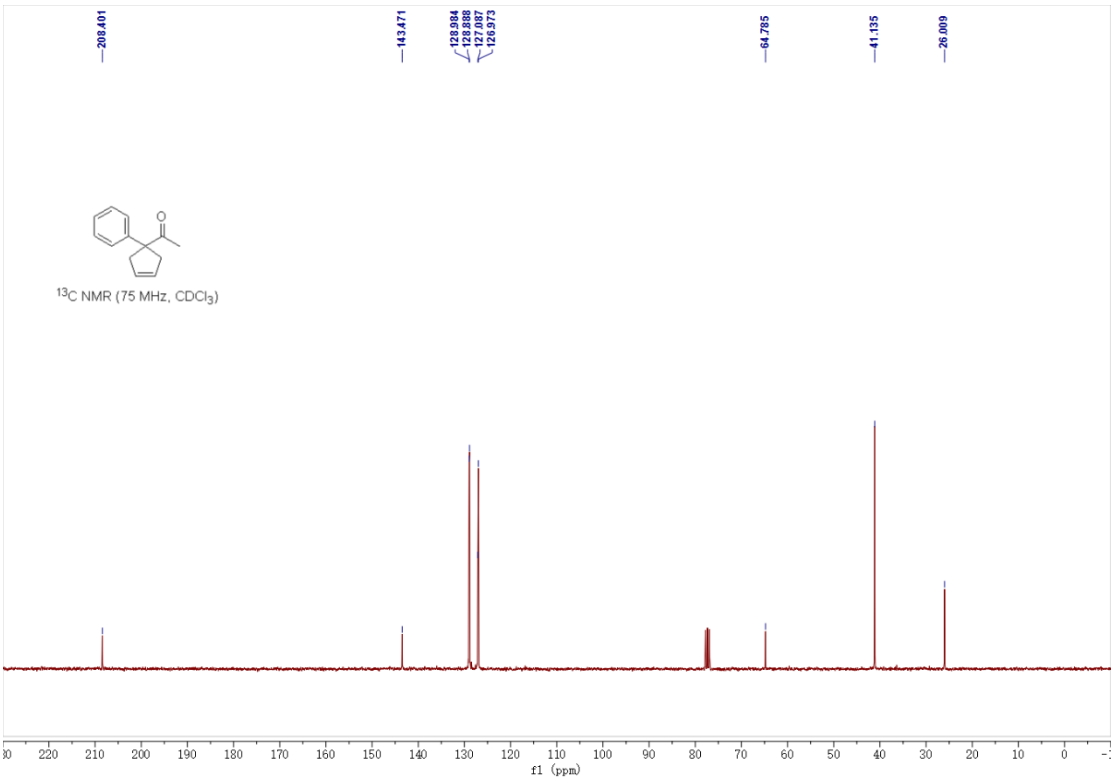

Supplementary Figure 72. ^{1}H and ^{13}C NMR spectra of **1ad**

N-((1-phenylcyclopent-3-en-1-yl)methyl)aniline (1ae)

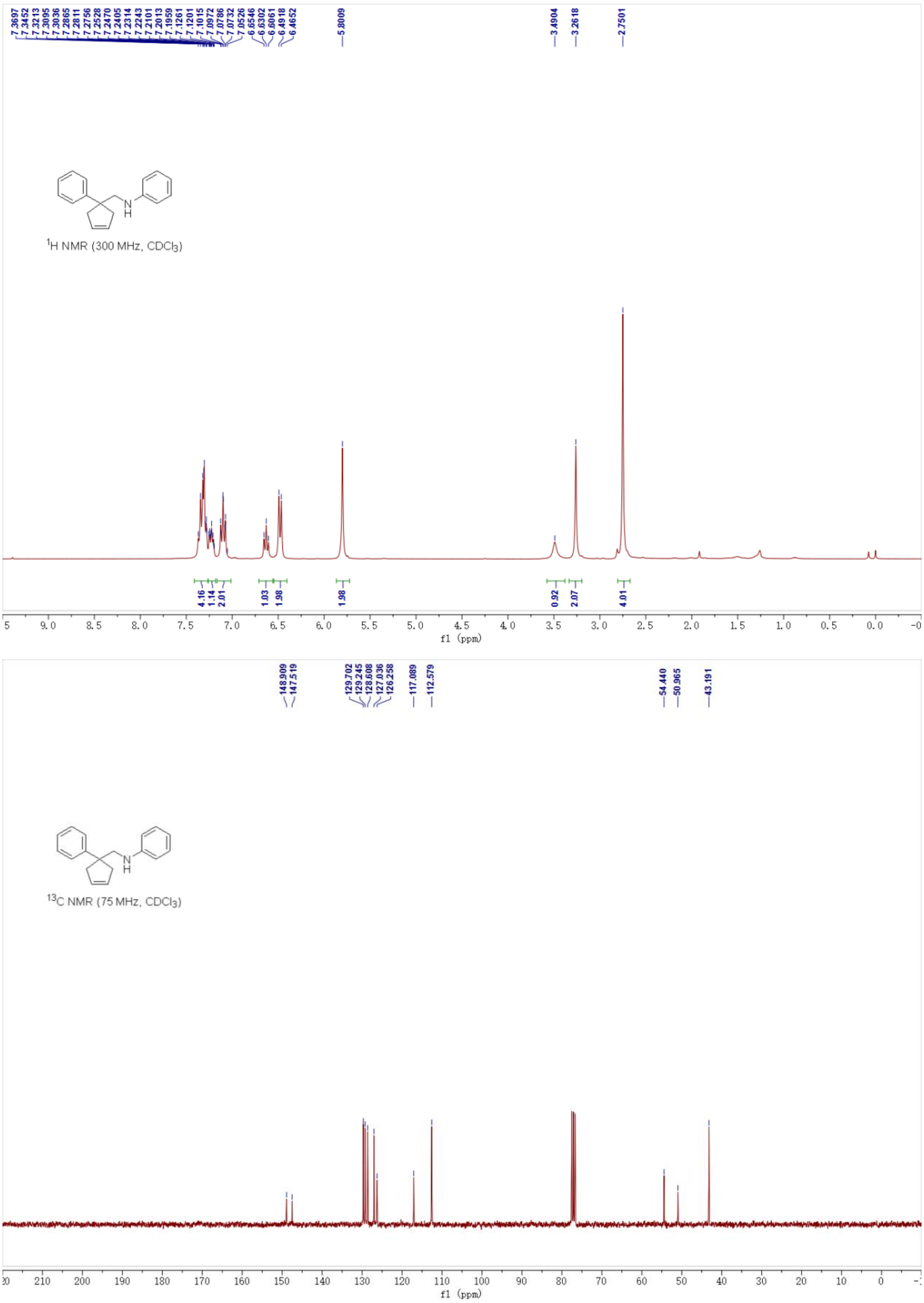

Supplementary Figure 73. ^{1}H and ^{13}C NMR spectra of **1ae**

(1R,4S)-N,1-diphenyl-4-((E)-2-(triisopropylsilyl)vinyl)cyclopent-2-ene-1-carboxamide (3a)

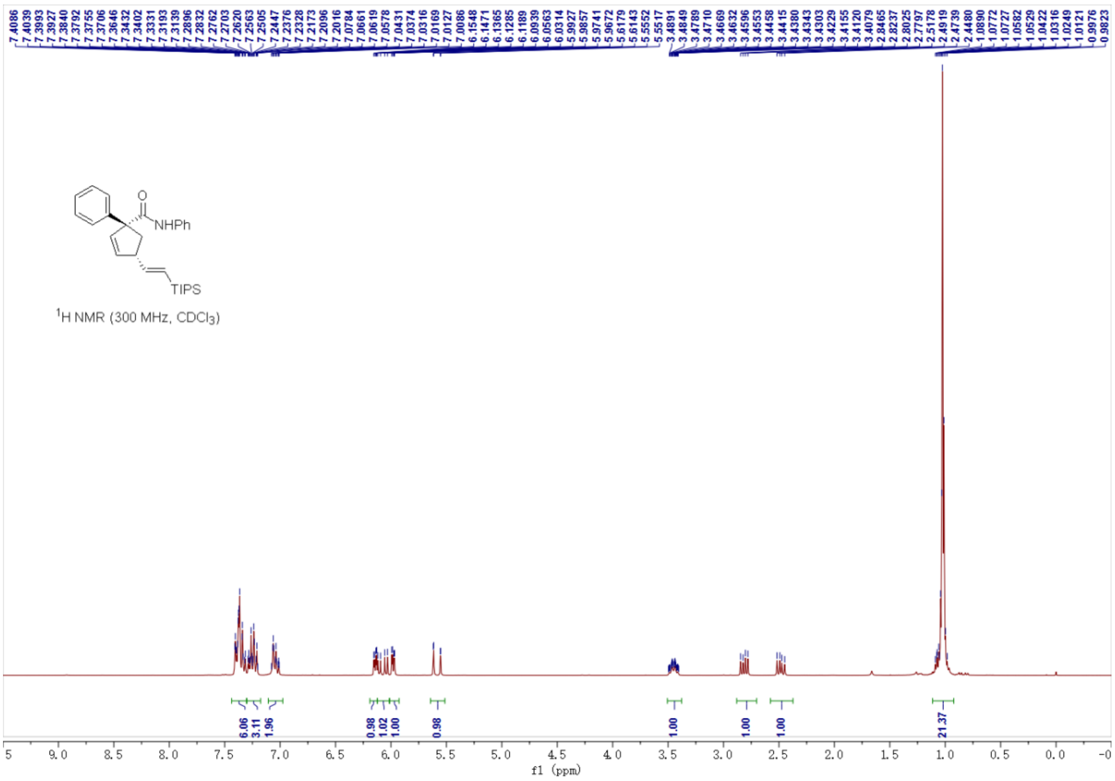

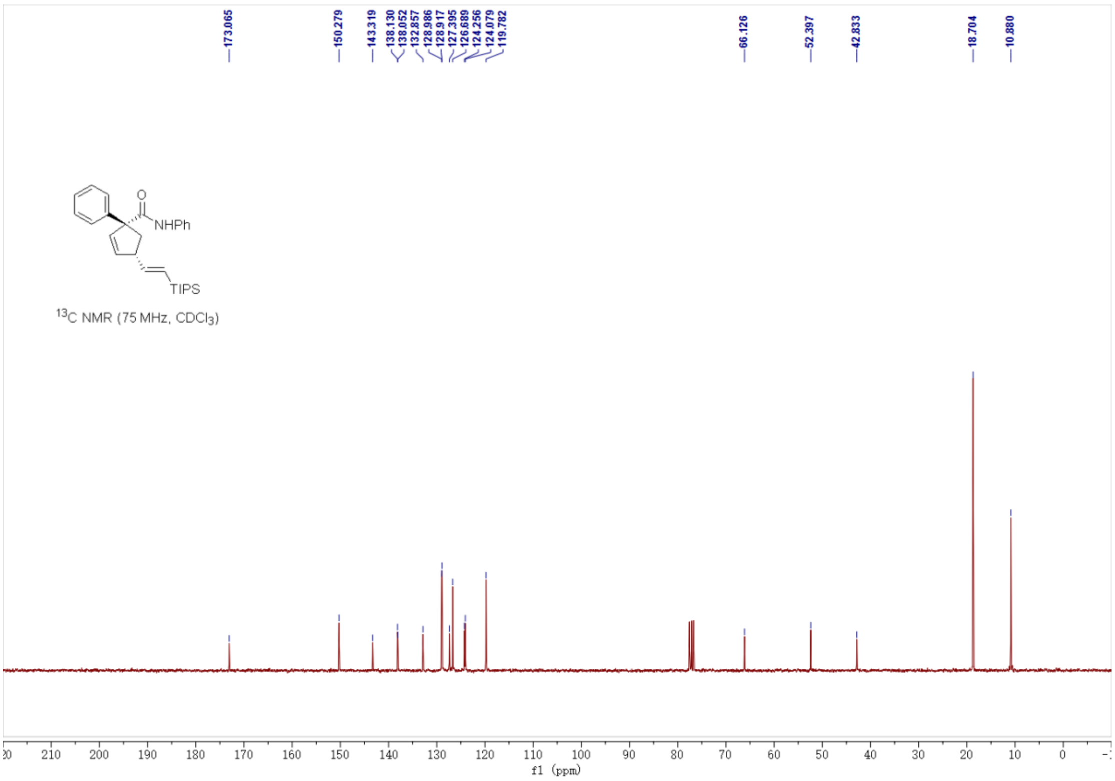

Supplementary Figure 74. ^{1}H and ^{13}C NMR spectra of **3a**

(1R,4S)-N-phenyl-1-(p-tolyl)-4-((E)-2-(triisopropylsilyl)vinyl)cyclopent-2-ene-1-carboxamide (3b)

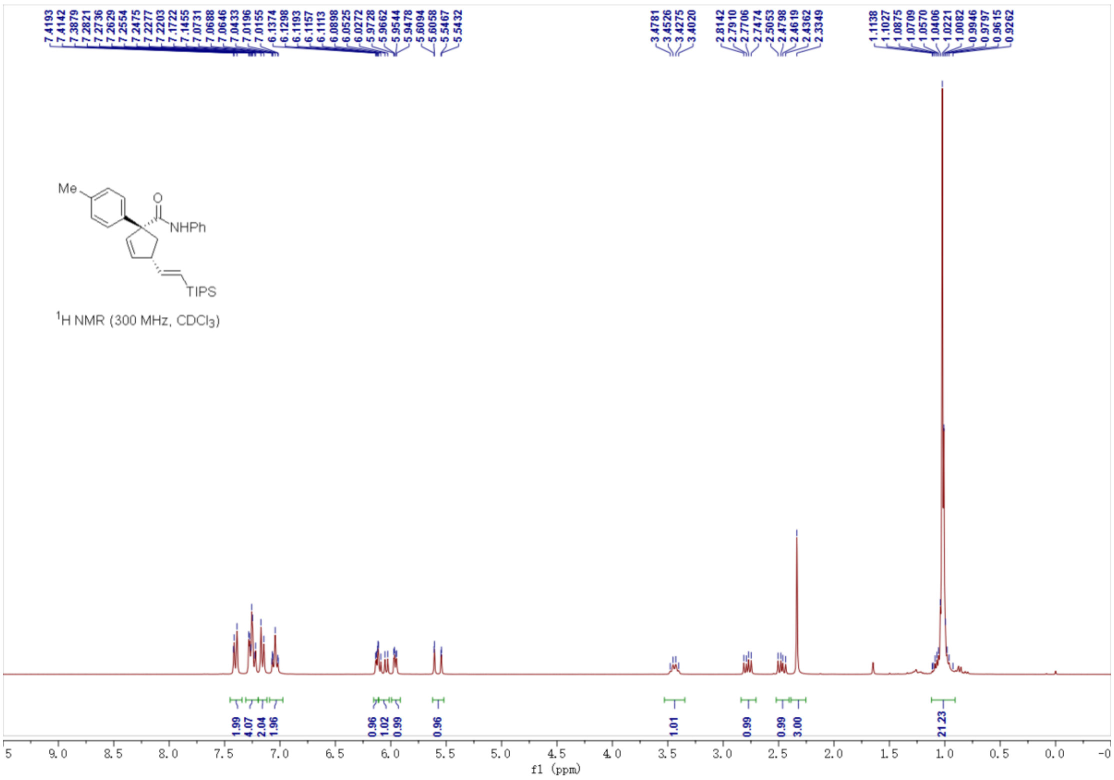

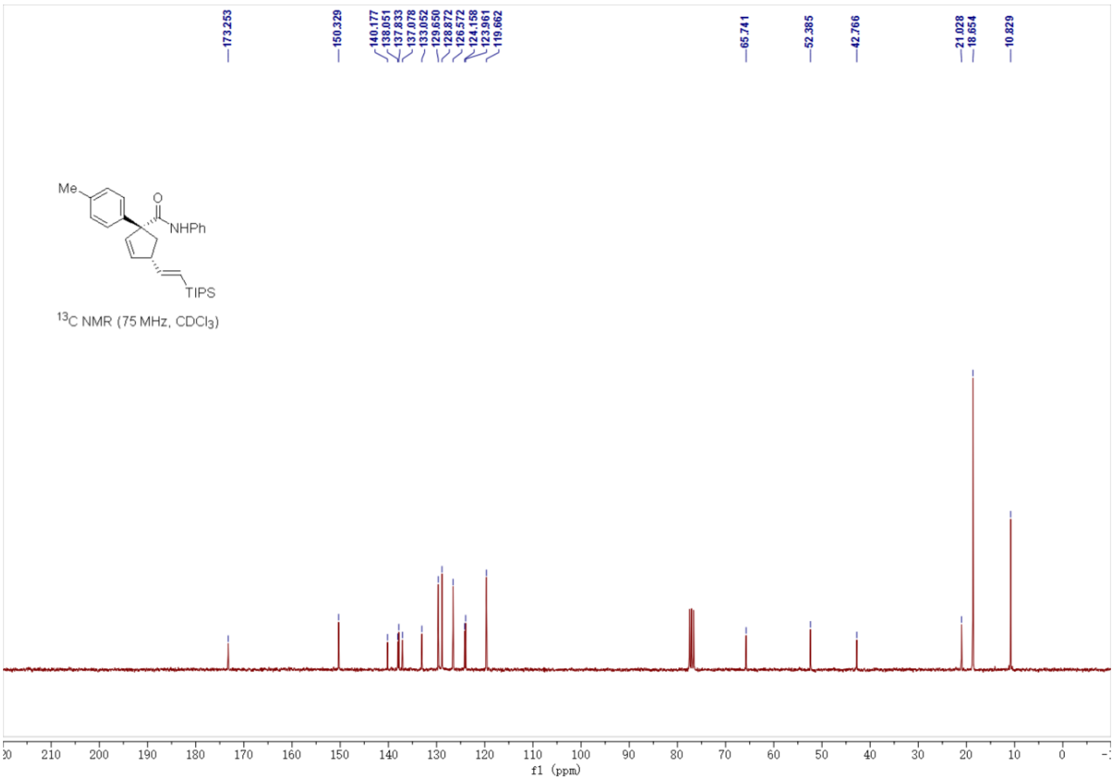

Supplementary Figure 75. ^{1}H and ^{13}C NMR spectra of **3b**

(1R,4S)-1-(4-methoxyphenyl)-N-phenyl-4-((E)-2-(triisopropylsilyl)vinyl)cyclopent-2-ene-1-carboxamide (3c)

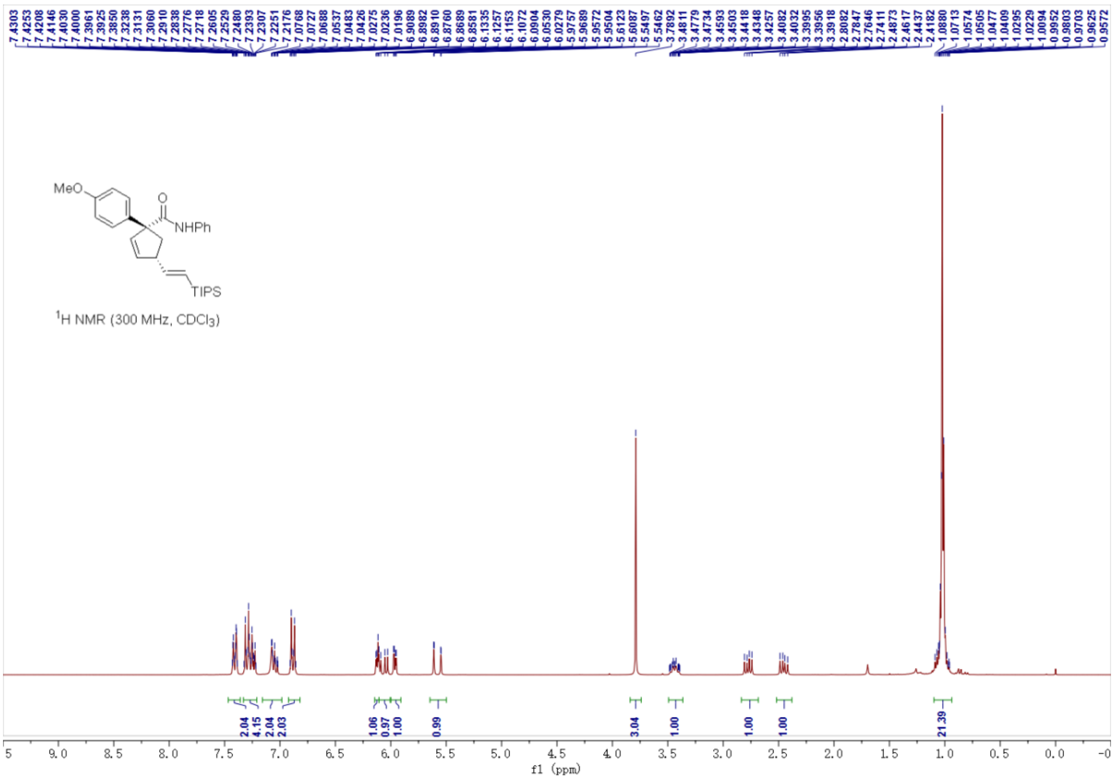

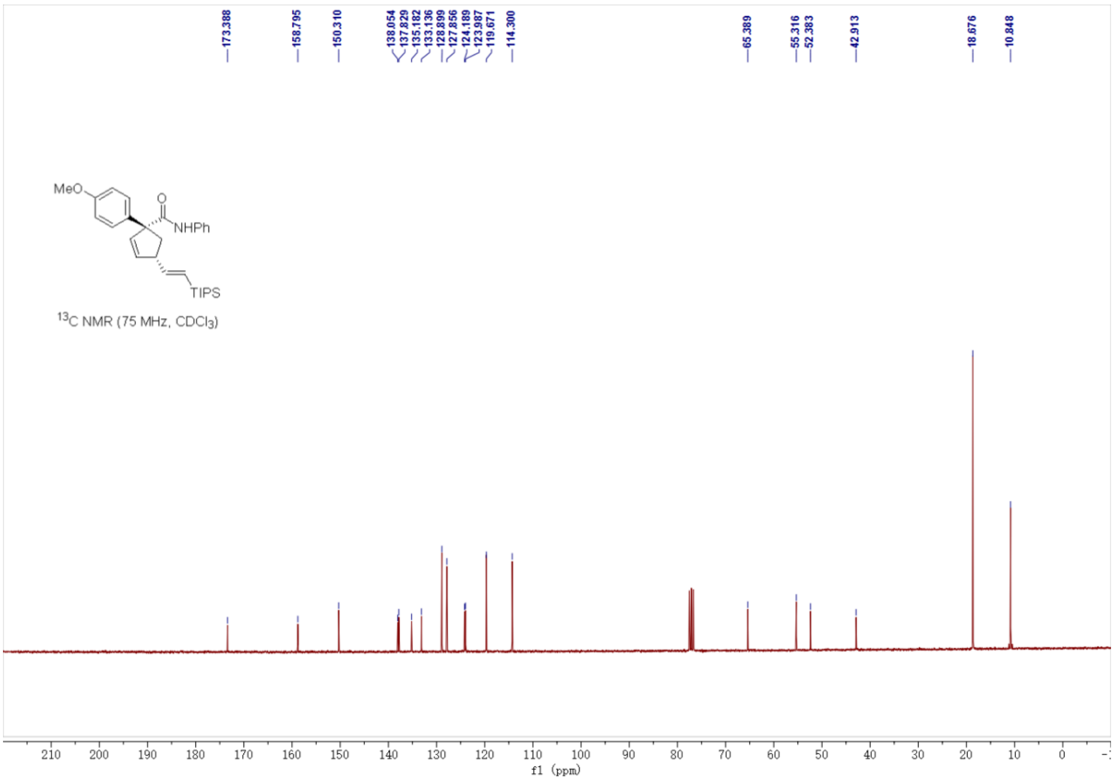

Supplementary Figure 76. ^{1}H and ^{13}C NMR spectra of **3c**

(1R,4S)-1-(4-(tert-butyl)phenyl)-N-phenyl-4-((E)-2-(triisopropylsilyl)vinyl)cyclopent-2-ene-1-carboxamide (3d)

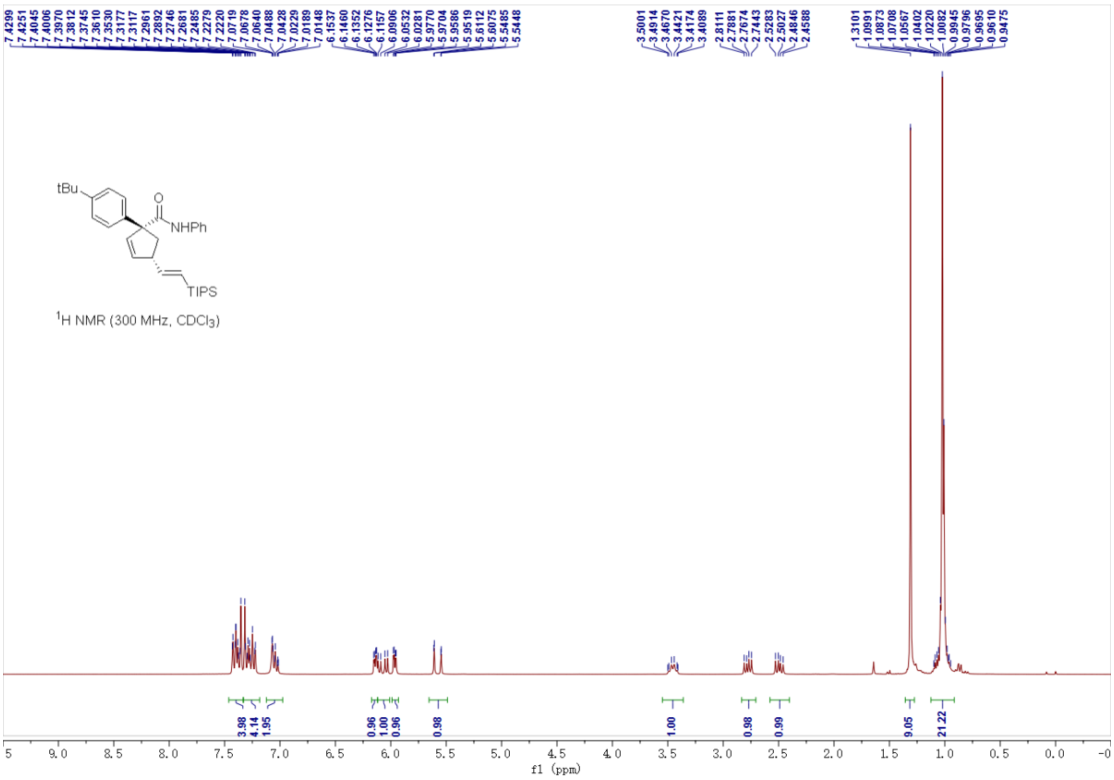

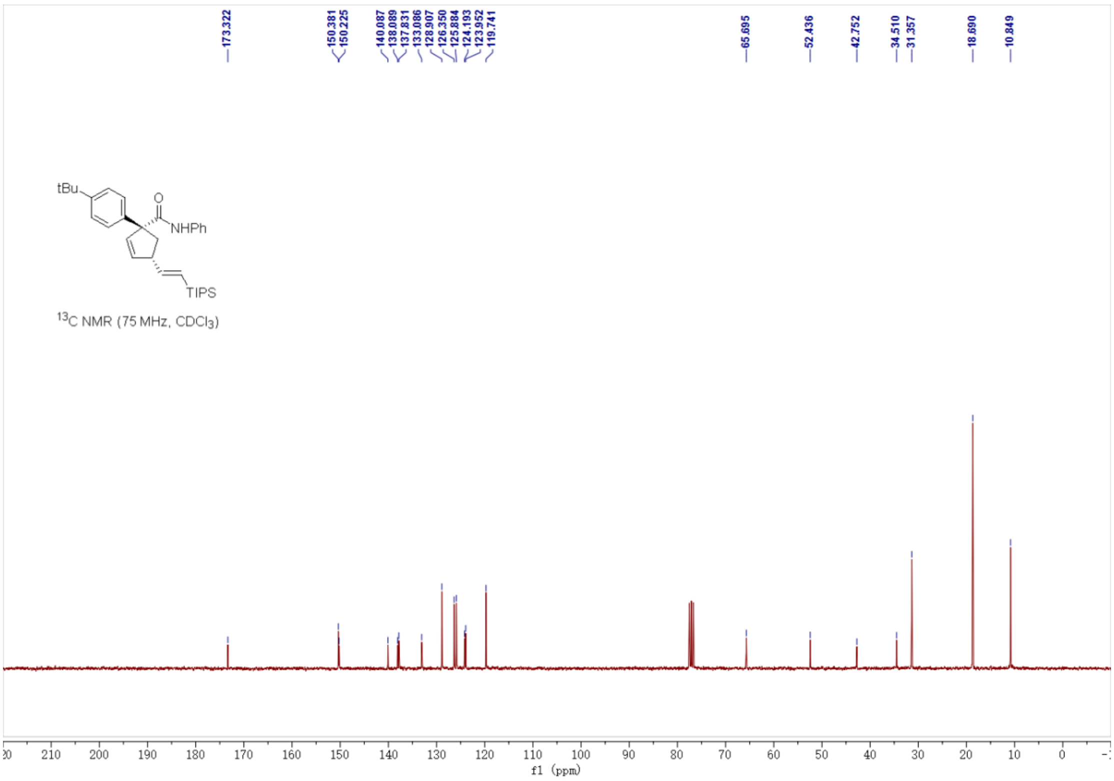

Supplementary Figure 77. ^{1}H and ^{13}C NMR spectra of **3d**

(1R,4S)-1-(4-fluorophenyl)-N-phenyl-4-((E)-2-(triisopropylsilyl)vinyl)cyclopent-2-ene-1-carboxamide (3e)

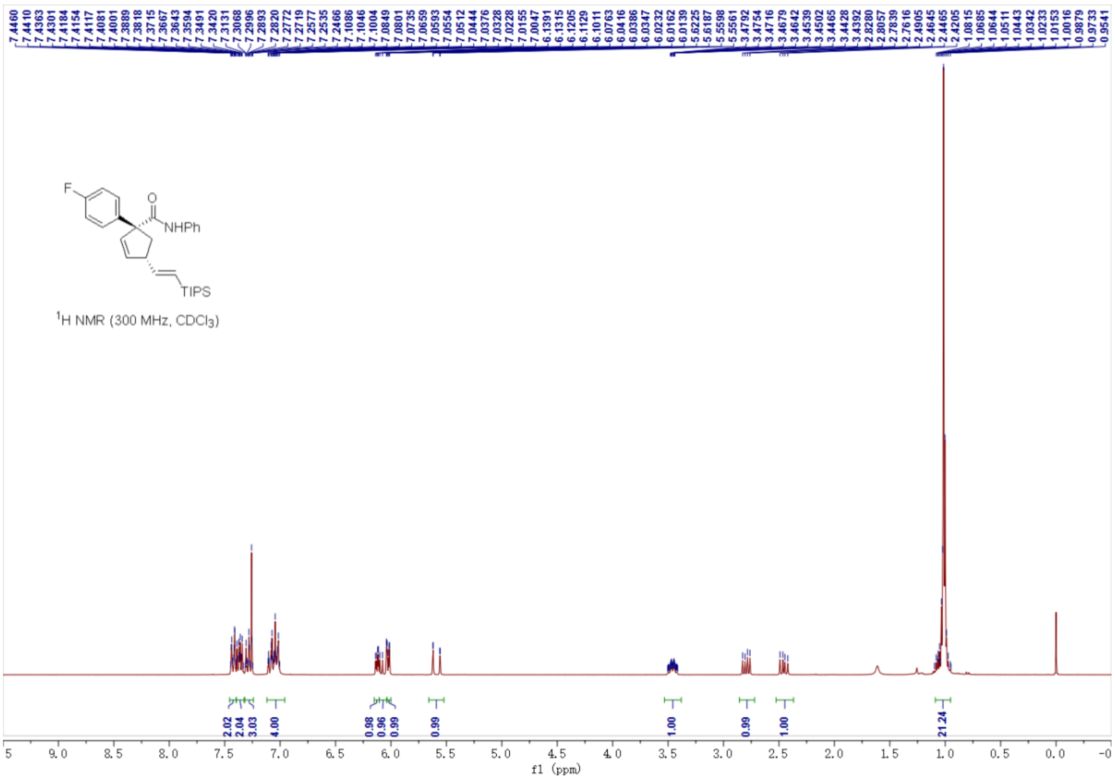

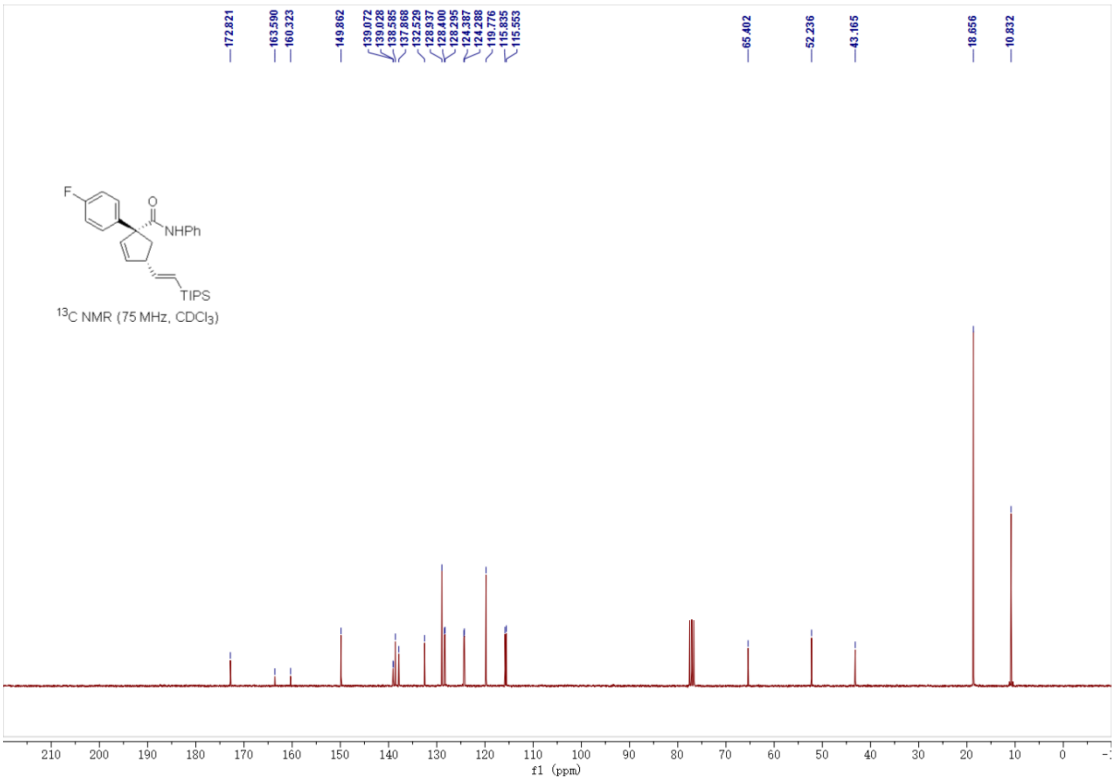

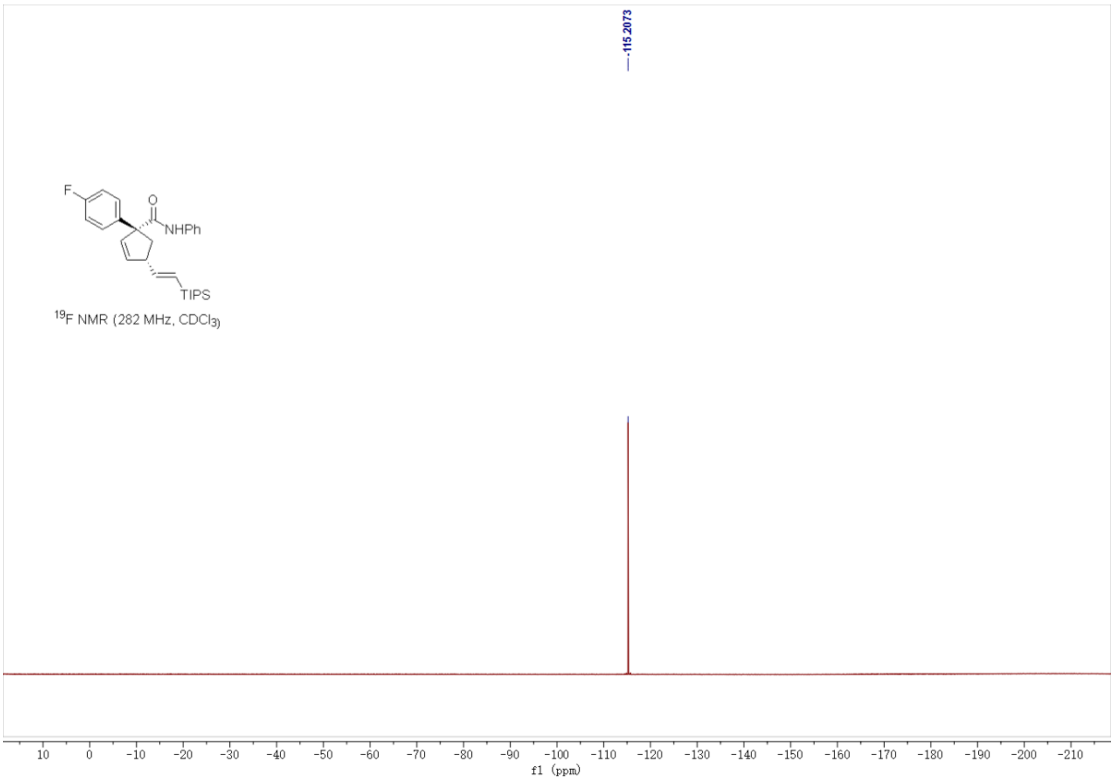

Supplementary Figure 78. ^{1}H , ^{13}C and ^{19}F NMR spectra of **3e**

(1R,4S)-1-(4-bromophenyl)-N-phenyl-4-((E)-2-(triisopropylsilyl)vinyl)cyclopent-2-ene-1-carboxamide (3f)

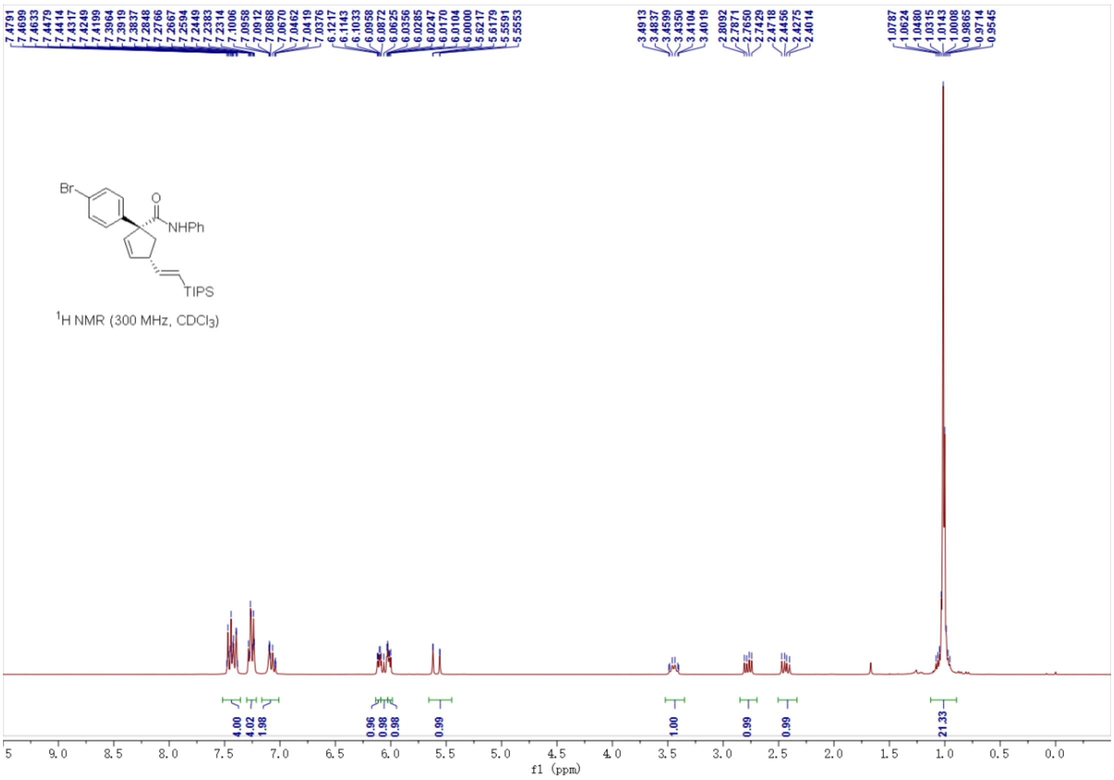

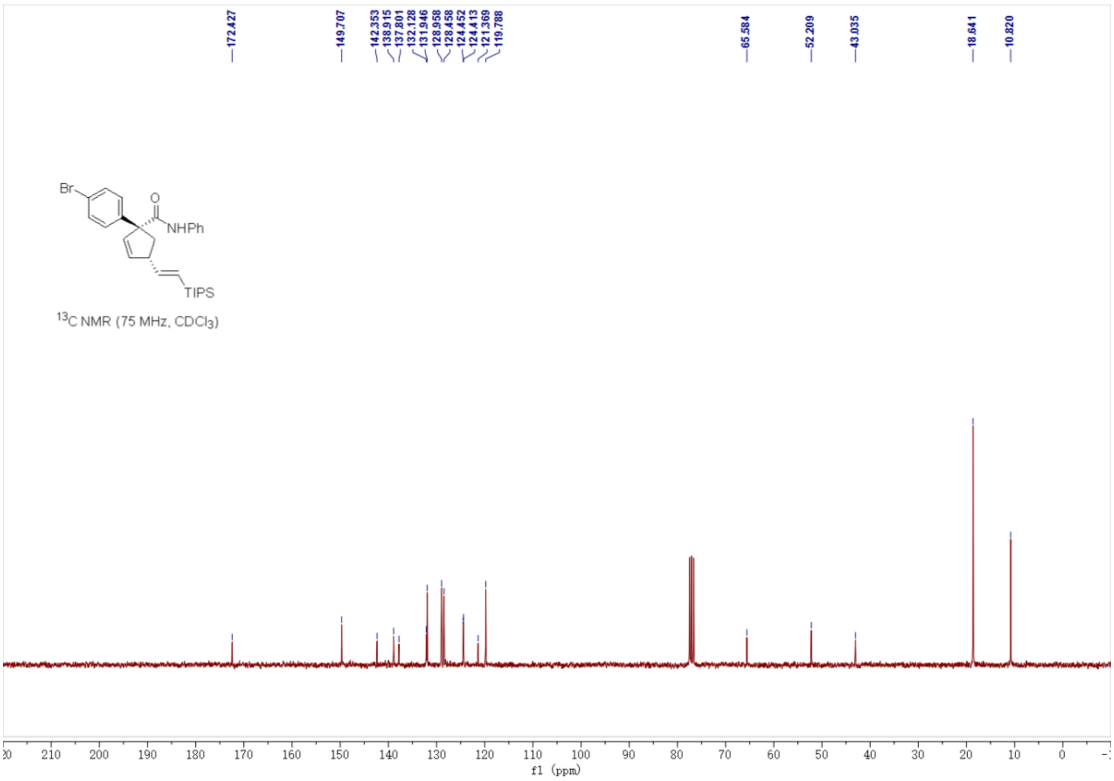

Supplementary Figure 79. ^{1}H and ^{13}C NMR spectra of **3f**

(1R,4S)-N-phenyl-1-(4-(trifluoromethyl)phenyl)-4-((E)-2-(triisopropylsilyl)vinyl)cyclopent-2-ene-1-carboxamide (3g)

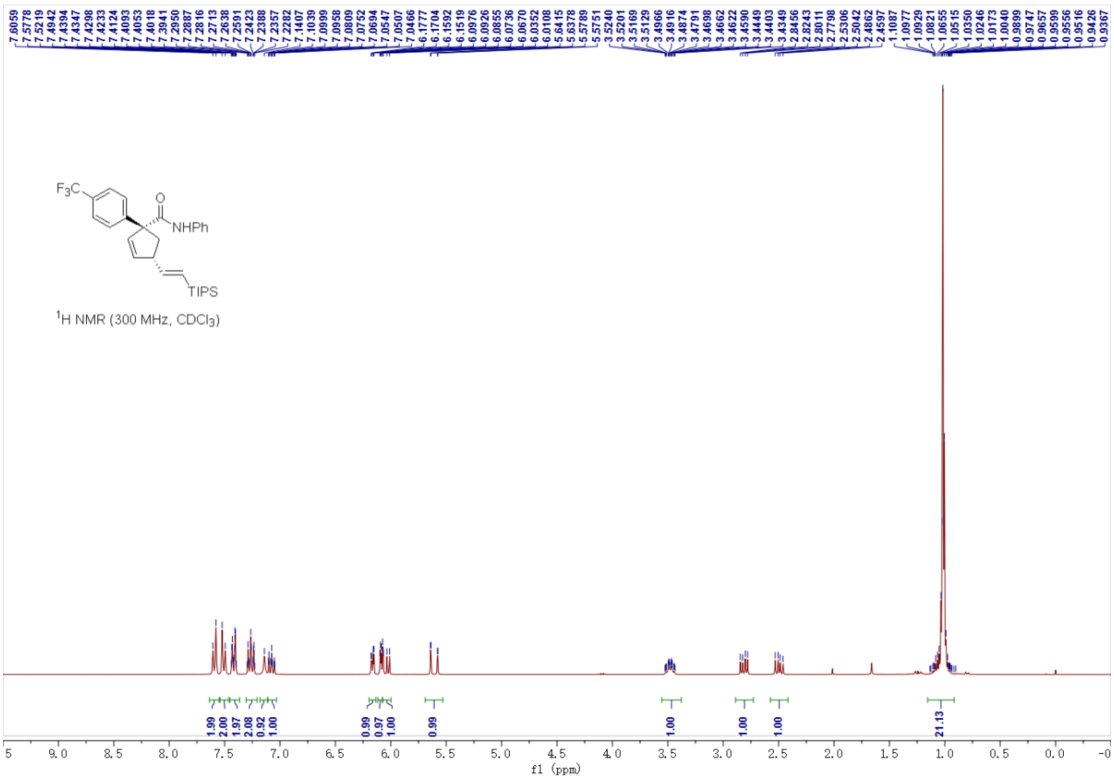

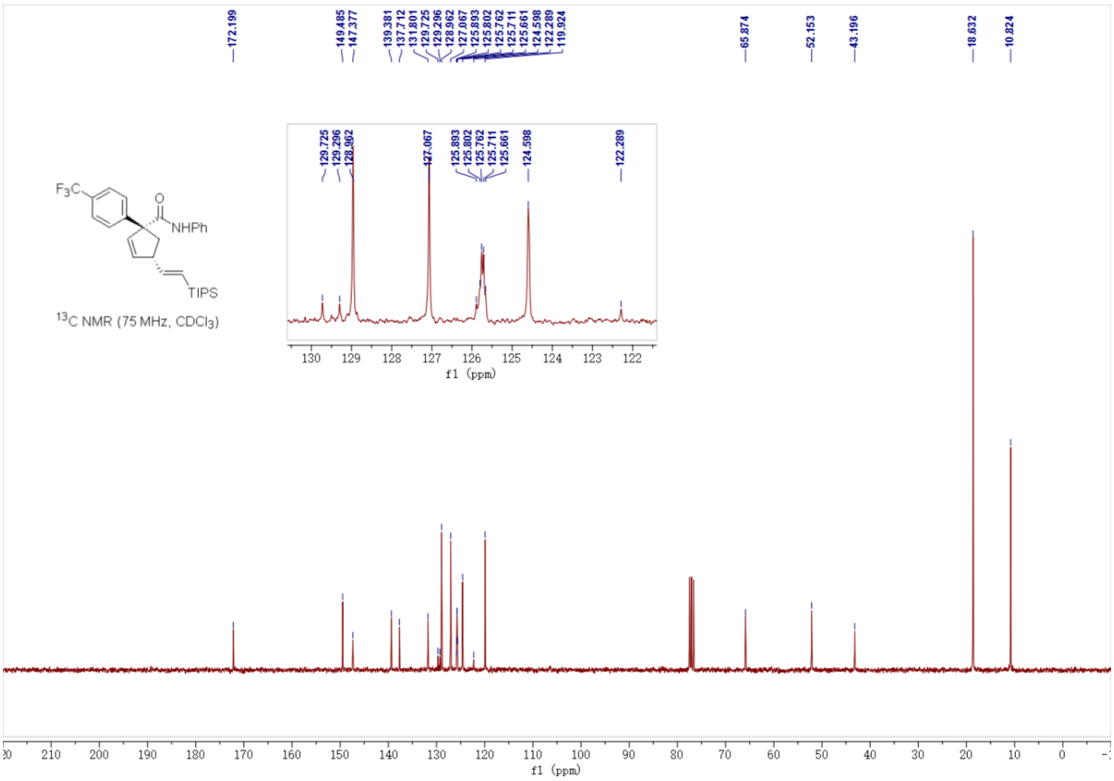

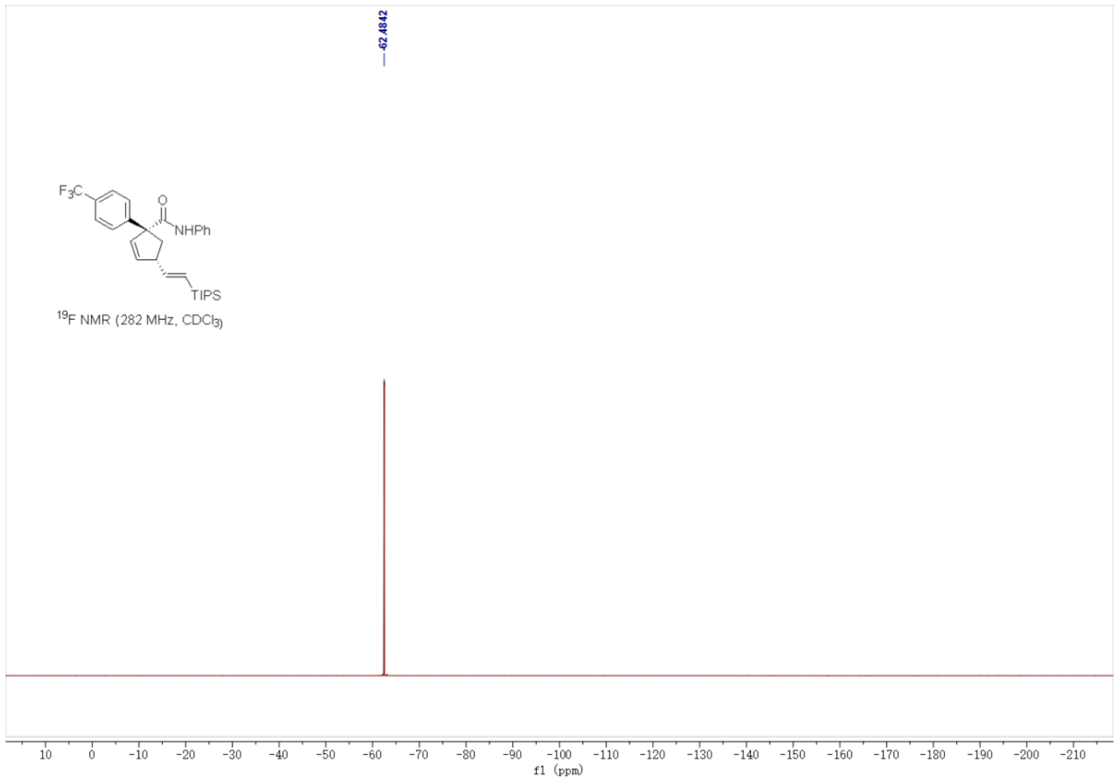

Supplementary Figure 80. ^{1}H , ^{13}C and ^{19}F NMR spectra of **3g**

(1R,4S)-1-(2-chlorophenyl)-N-phenyl-4-((E)-2-(triisopropylsilyl)vinyl)cyclopent-2-ene-1-carboxamide (3h)

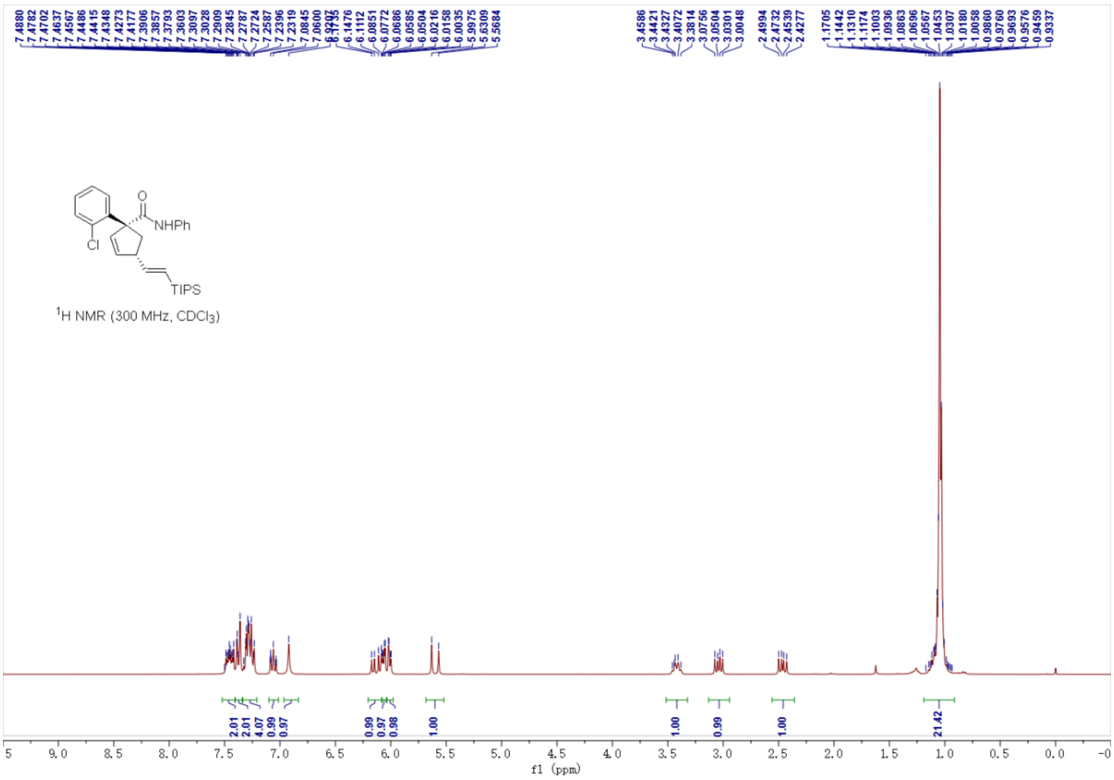

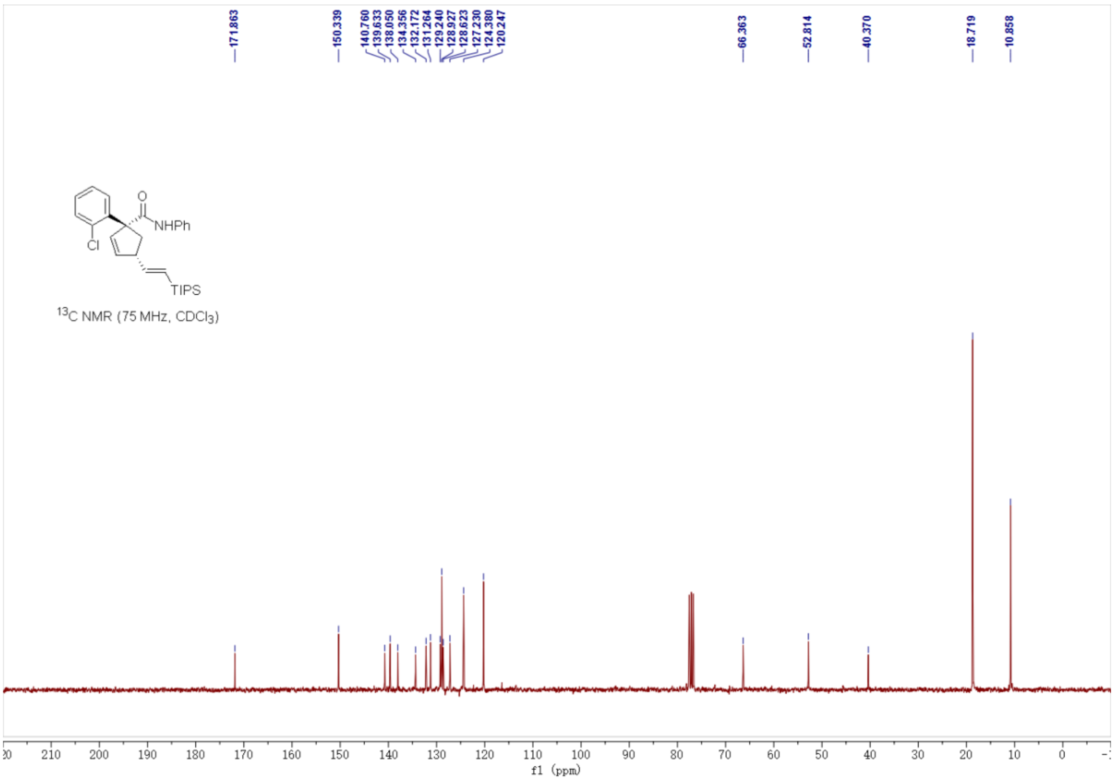

Supplementary Figure 81. ^{1}H and ^{13}C NMR spectra of **3h**

(1R,4S)-N-phenyl-1-(m-tolyl)-4-((E)-2-(triisopropylsilyl)vinyl)cyclopent-2-ene-1-carboxamide (3i)

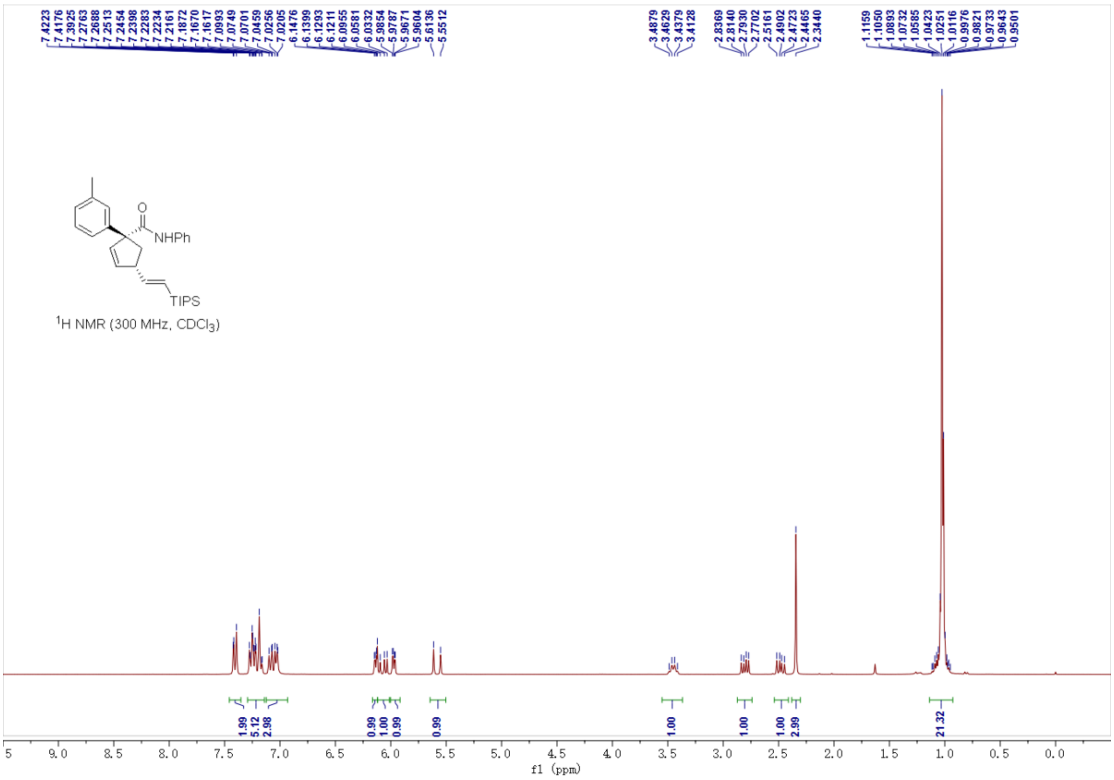

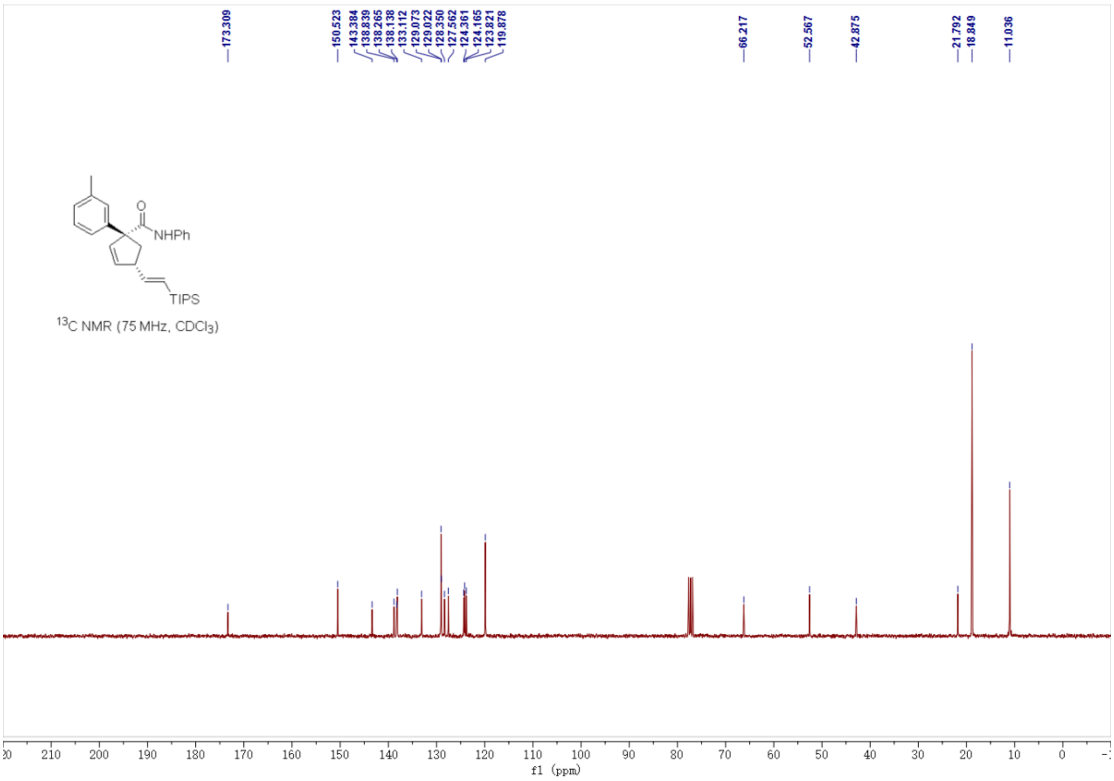

Supplementary Figure 82. ^{1}H and ^{13}C NMR spectra of **3i**

(1S,4S)-N-phenyl-1-(thiophen-2-yl)-4-((E)-2-(triisopropylsilyl)vinyl)cyclopent-2-ene-1-carboxamide (3j)

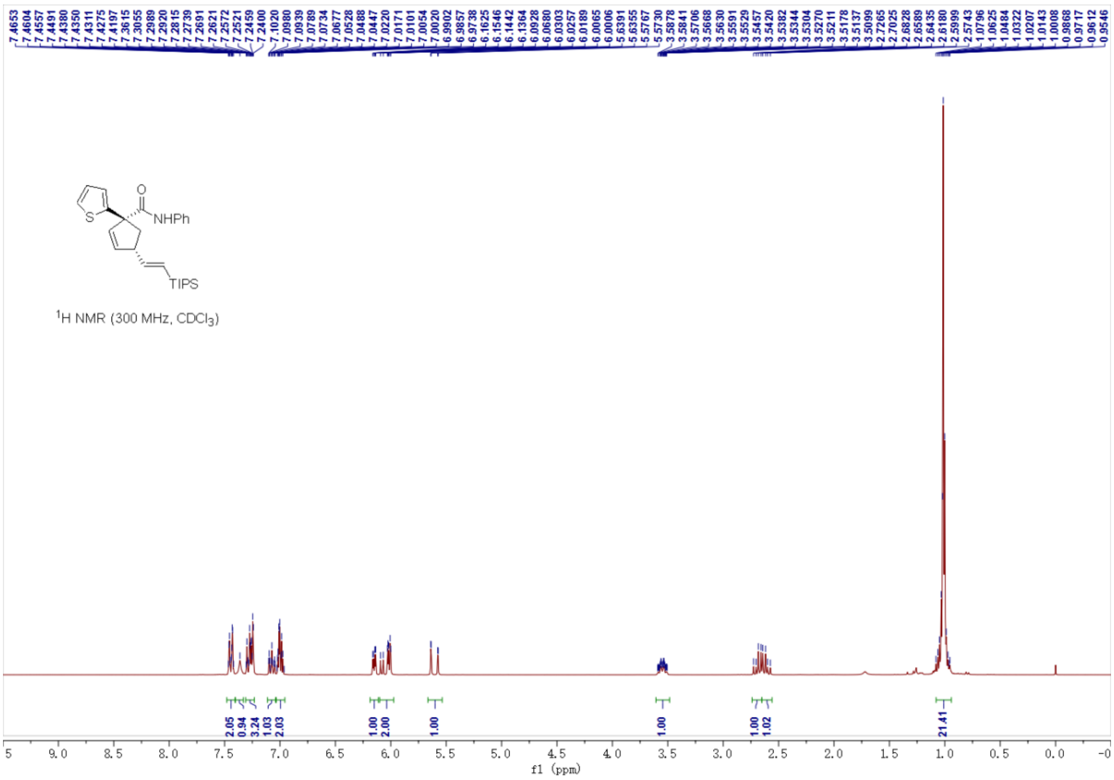

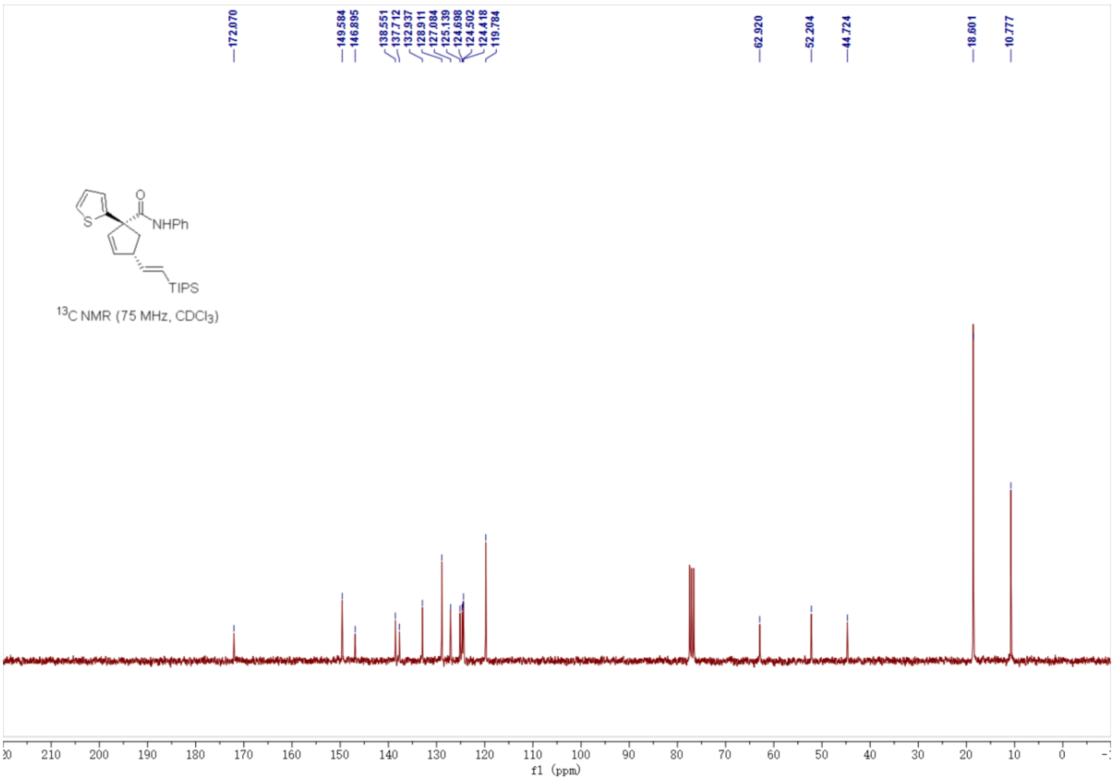

Supplementary Figure 83. ^{1}H and ^{13}C NMR spectra of **3j**

(1S,4S)-1-(furan-3-ylmethyl)-N-phenyl-4-((E)-2-(triisopropylsilyl)vinyl)cyclopent-2-ene-1-carboxamide (3k)

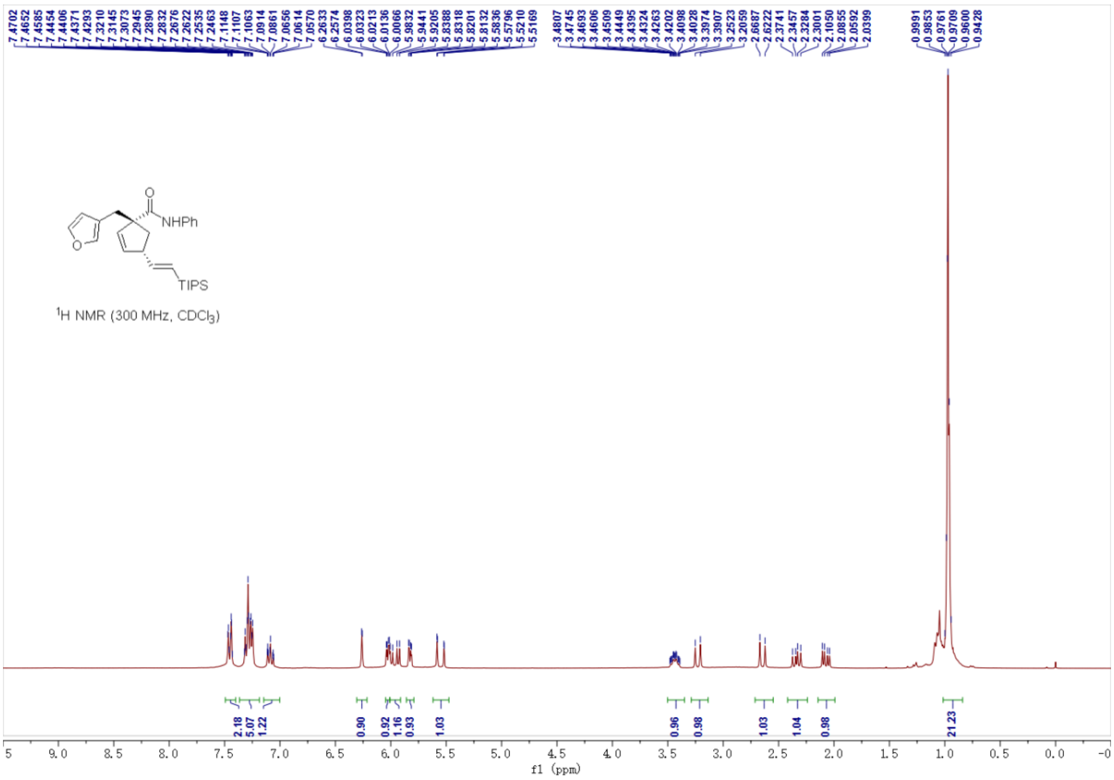

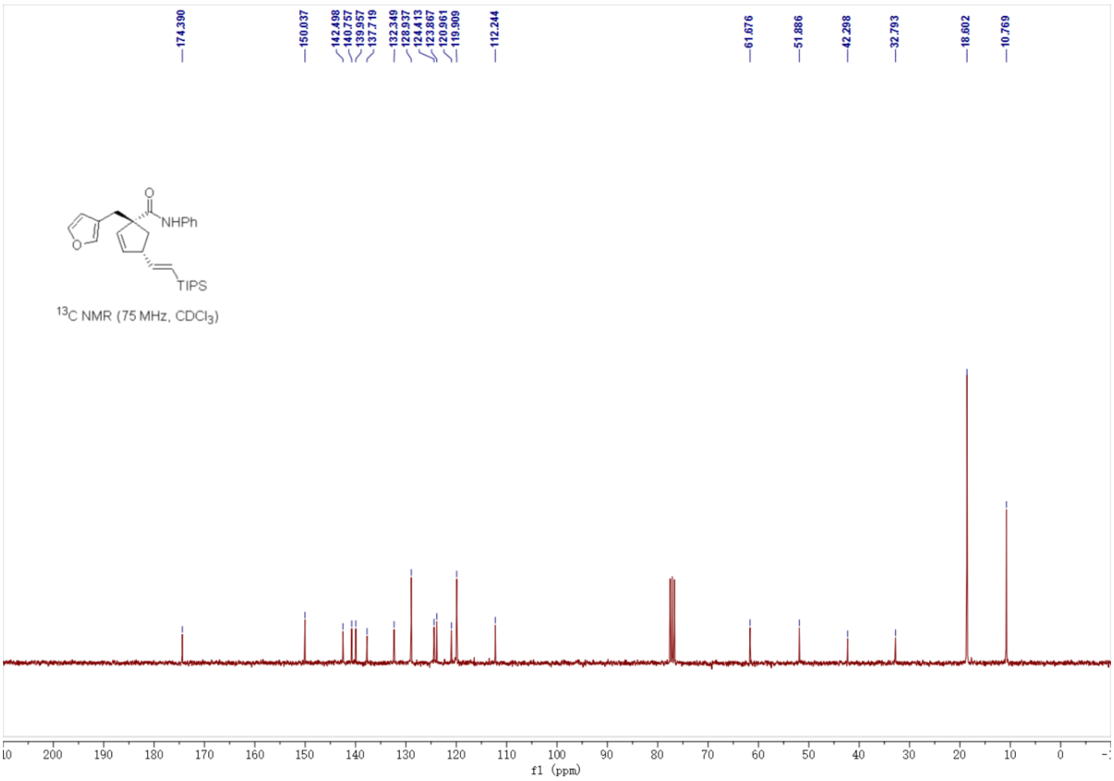

Supplementary Figure 84. ^{1}H and ^{13}C NMR spectra of **3k**

(1S,4S)-1-benzyl-N-phenyl-4-((E)-2-(triisopropylsilyl)vinyl)cyclopent-2-ene-1-carboxamide (3l)

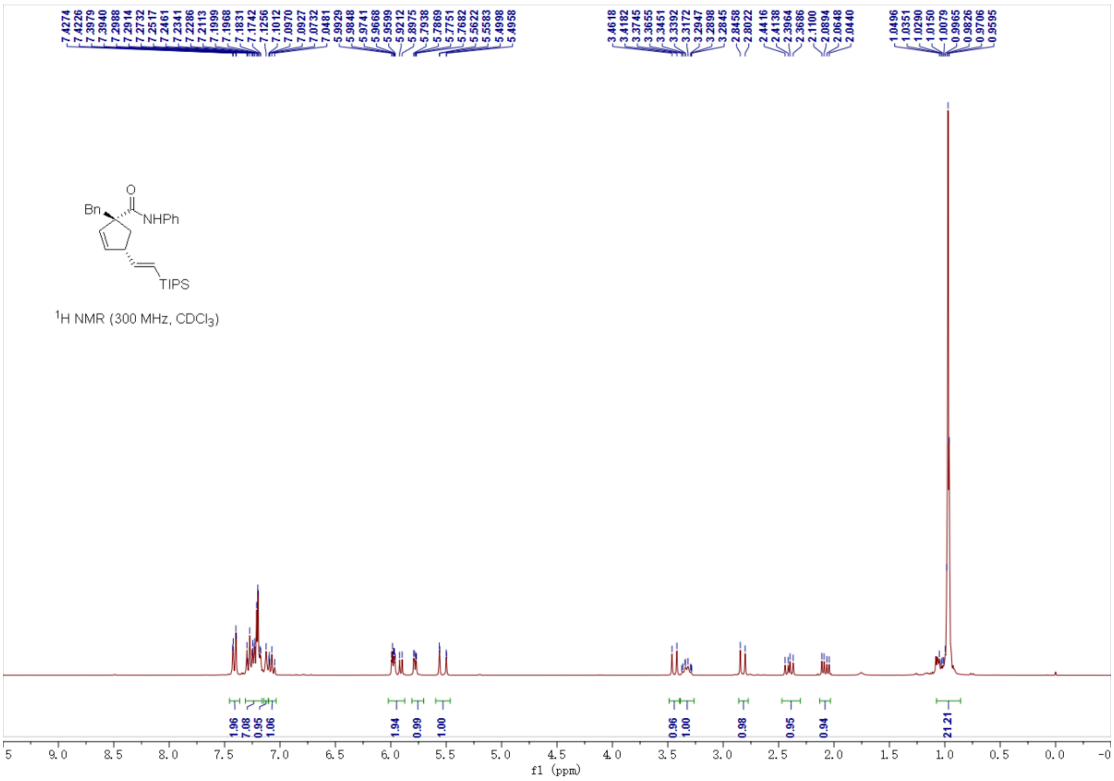

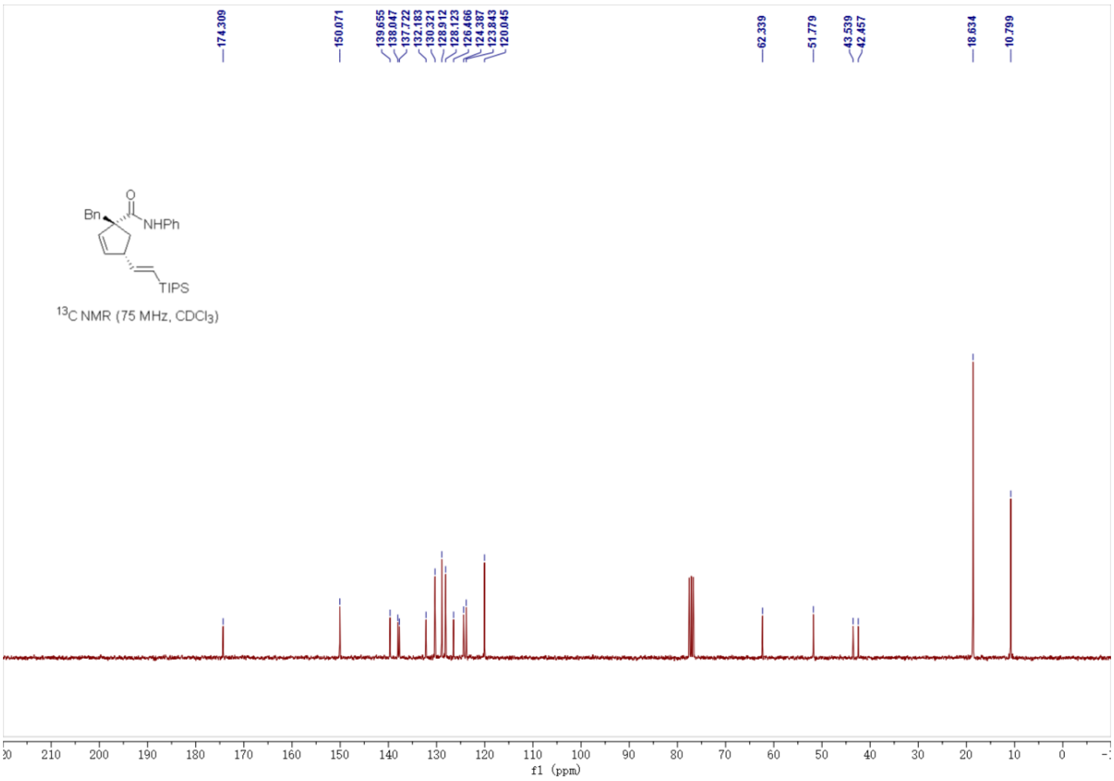

Supplementary Figure 85. ^{1}H and ^{13}C NMR spectra of **3l**

(1R,4S)-1-methyl-N-phenyl-4-((E)-2-(triisopropylsilyl)vinyl)cyclopent-2-ene-1-carboxamide (3m)

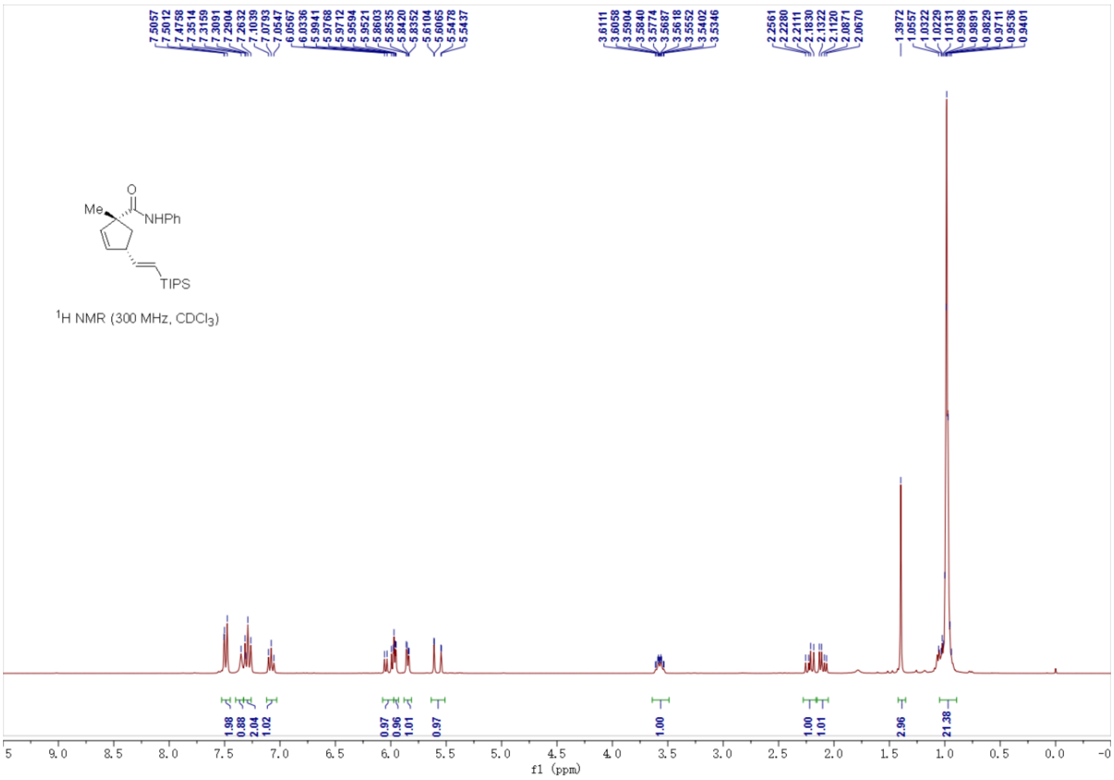

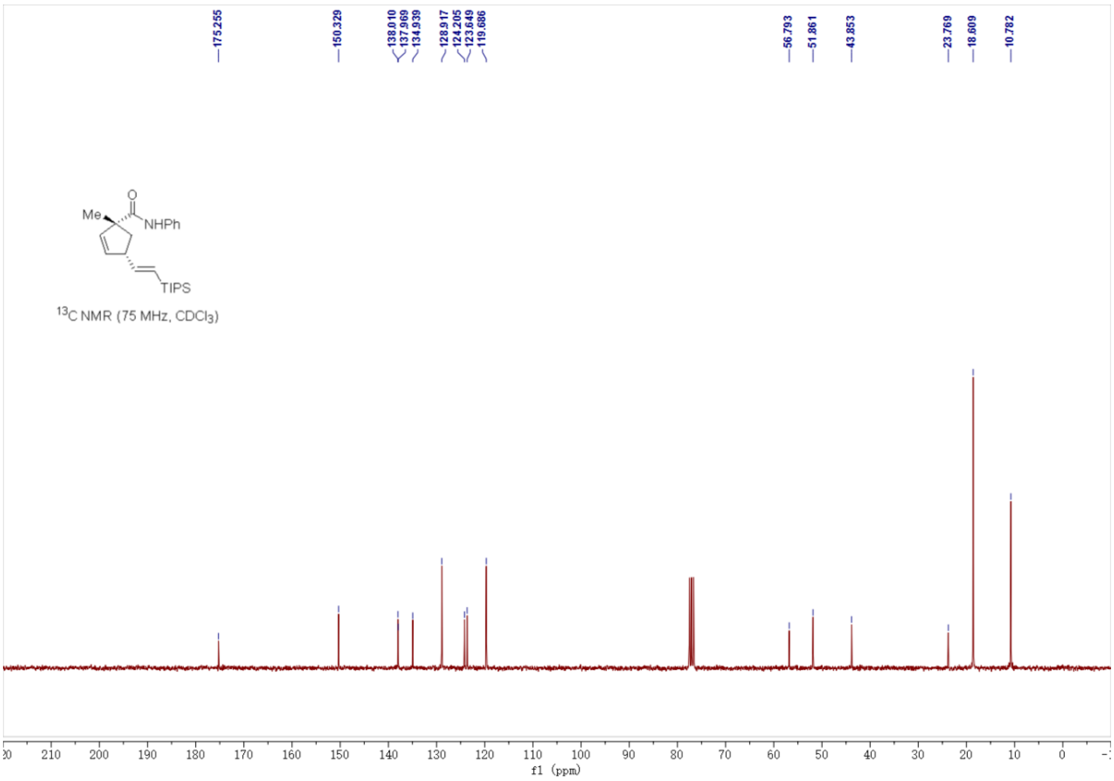

Supplementary Figure 86. ^{1}H and ^{13}C NMR spectra of **3m**

(1S,4S)-1-(2-methylallyl)-N-phenyl-4-((E)-2-(triisopropylsilyl)vinyl)cyclopent-2-ene-1-carboxamide (3n)

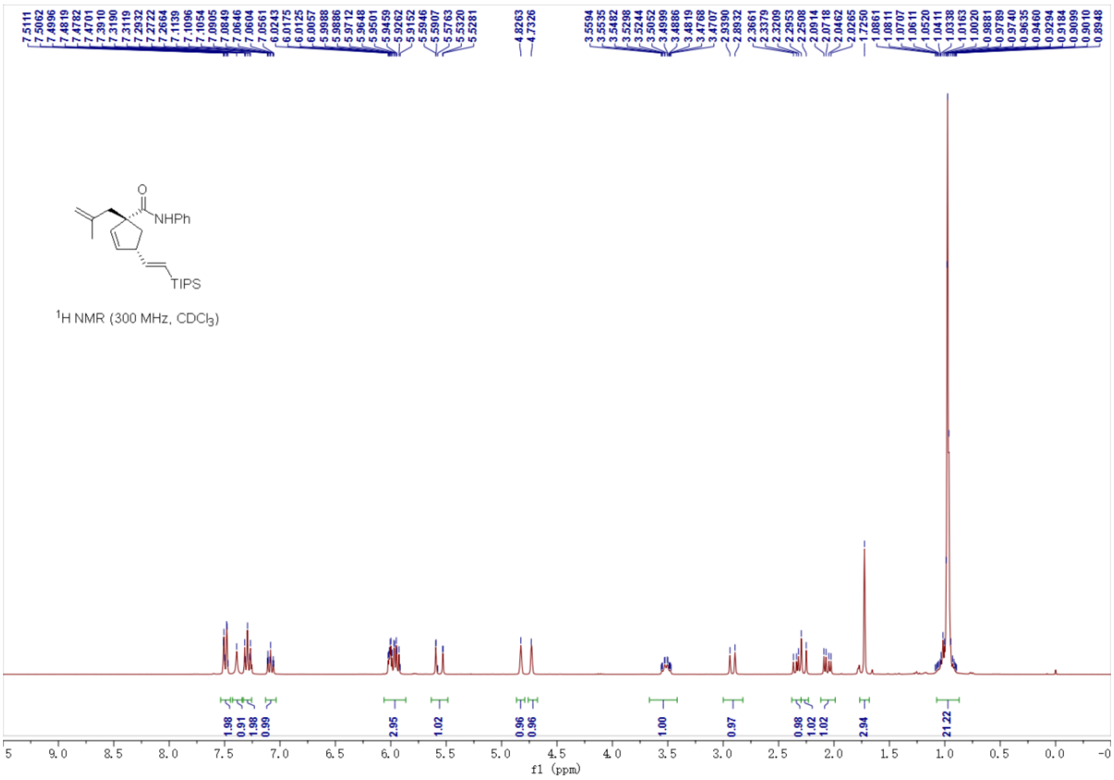

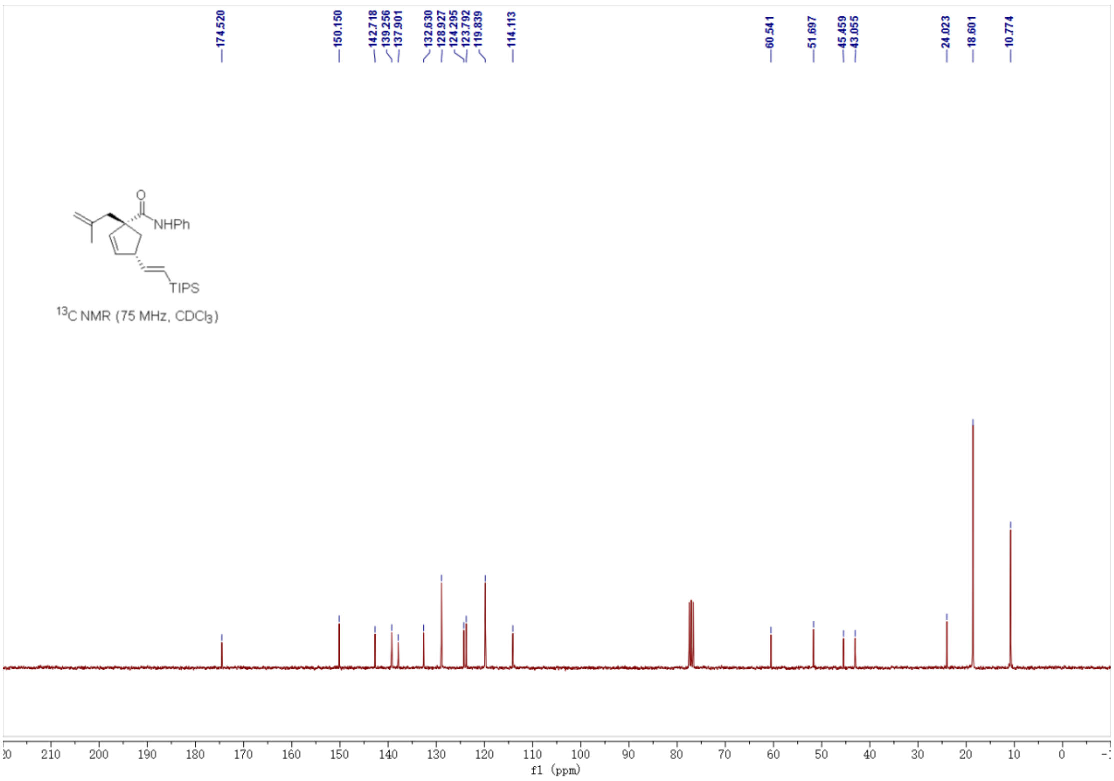

Supplementary Figure 87. ^{1}H and ^{13}C NMR spectra of **3n**

***tert*-butyl ((1S,4S)-1-(phenylcarbamoyl)-4-((E)-2-(triisopropylsilyl)vinyl)cyclopent-2-en-1-yl)carbamate (3o)**

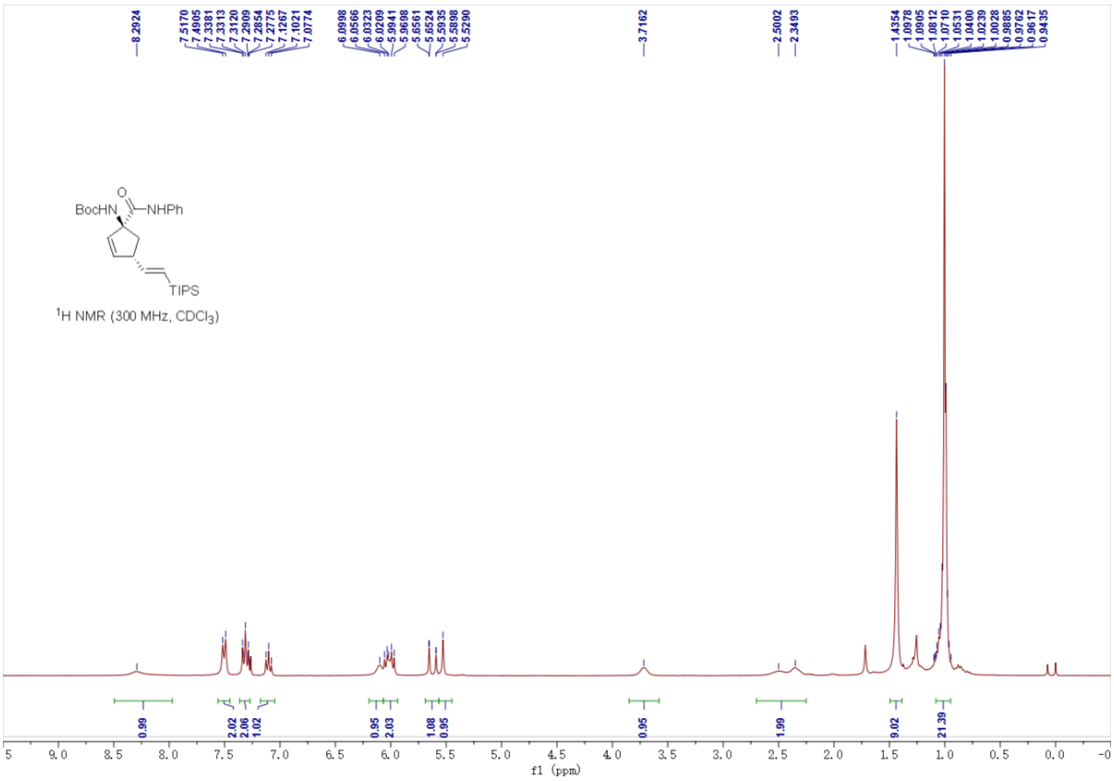

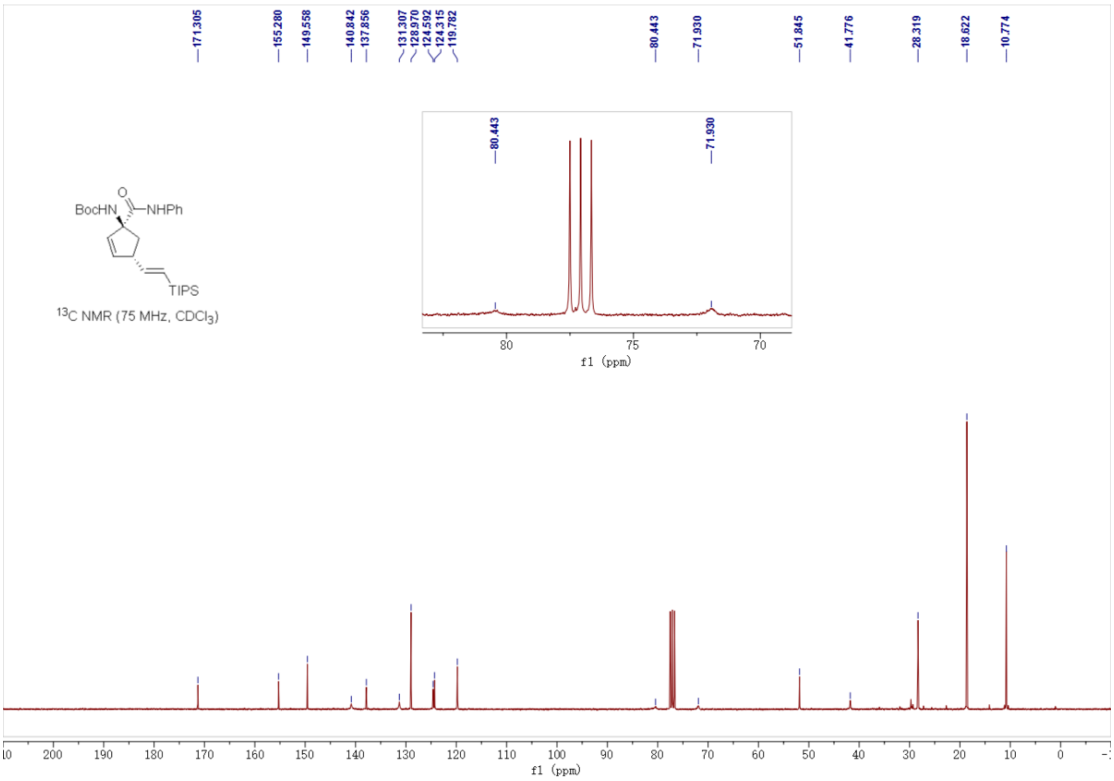

Supplementary Figure 88. ^{1}H and ^{13}C NMR spectra of **3o**

(1R,4S)-N-phenyl-4-((E)-2-(triisopropylsilyl)vinyl)cyclopent-2-ene-1-carboxamide (3p)

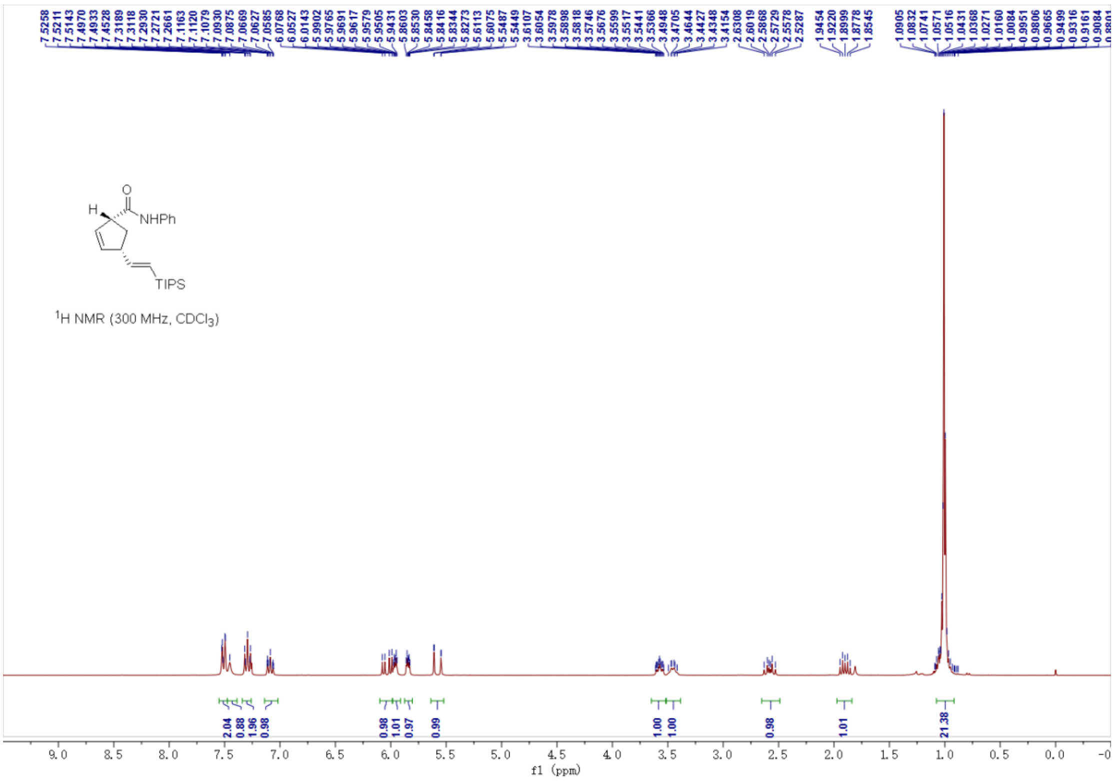

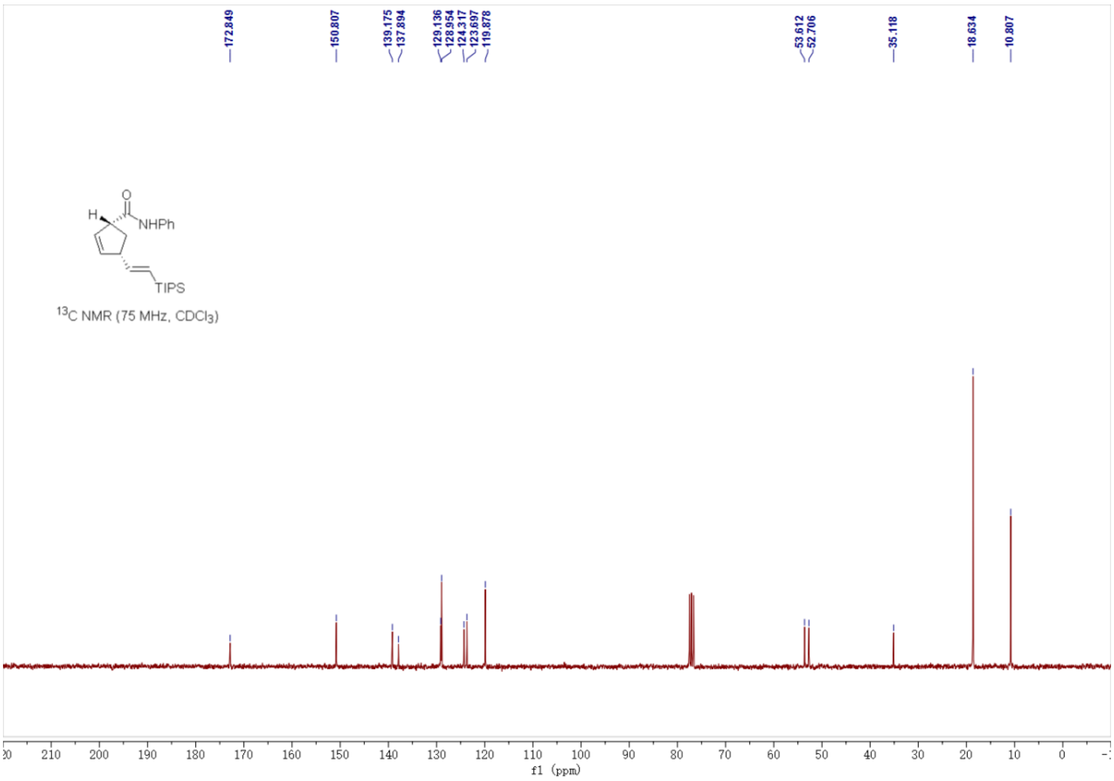

Supplementary Figure 89. ^{1}H and ^{13}C NMR spectra of **3p**

(1R,4S)-1-phenyl-N-(p-tolyl)-4-((E)-2-(triisopropylsilyl)vinyl)cyclopent-2-ene-1-carboxamide (3q)

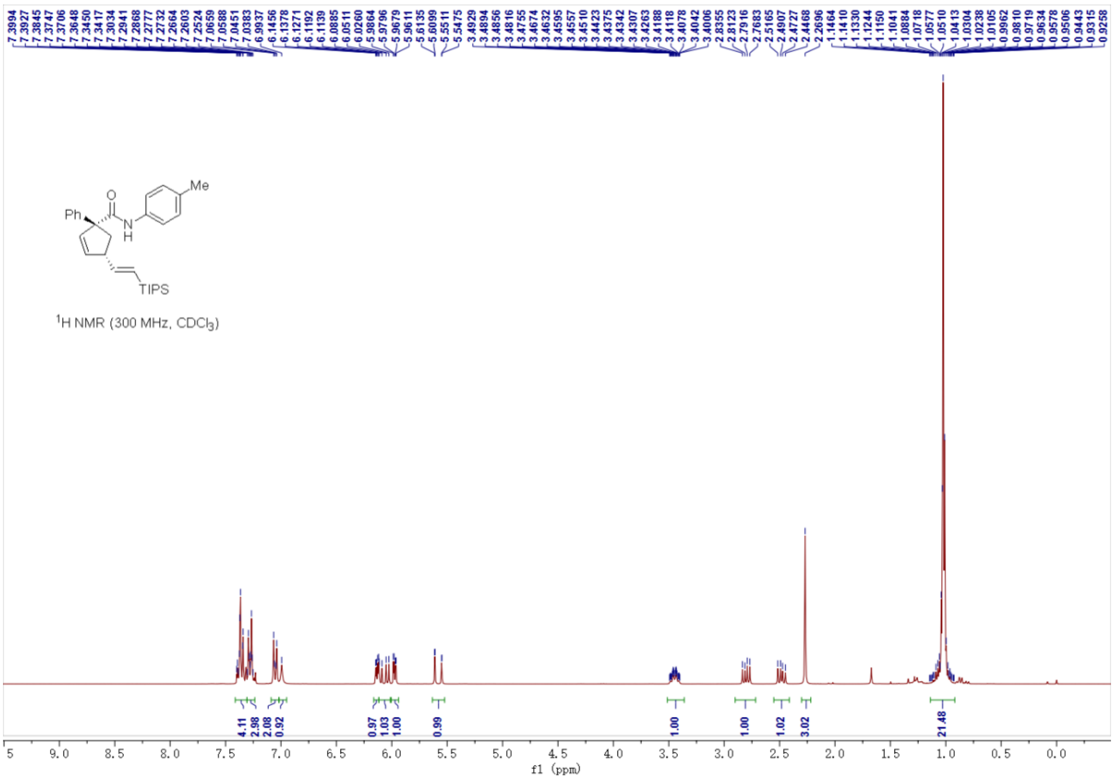

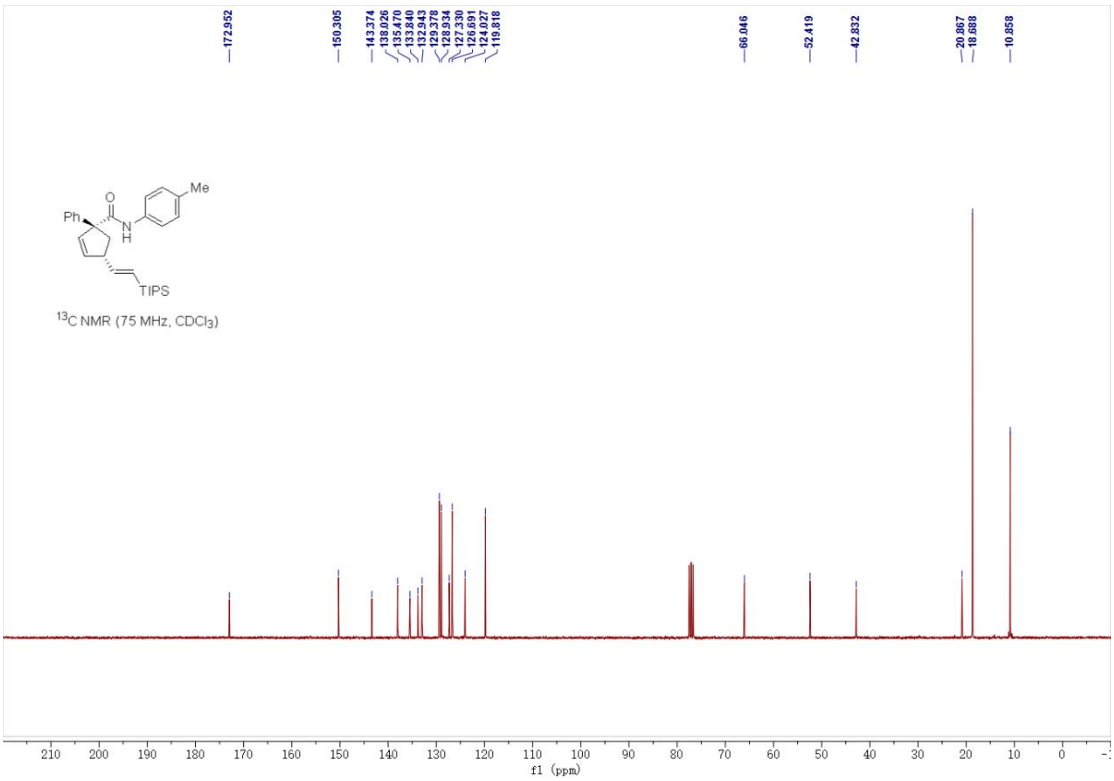

Supplementary Figure 90. ^{1}H and ^{13}C NMR spectra of **3q**

(1R,4S)-N-(4-methoxyphenyl)-1-phenyl-4-((E)-2-(triisopropylsilyl)vinyl)cyclopent-2-ene-1-carboxamide (3r)

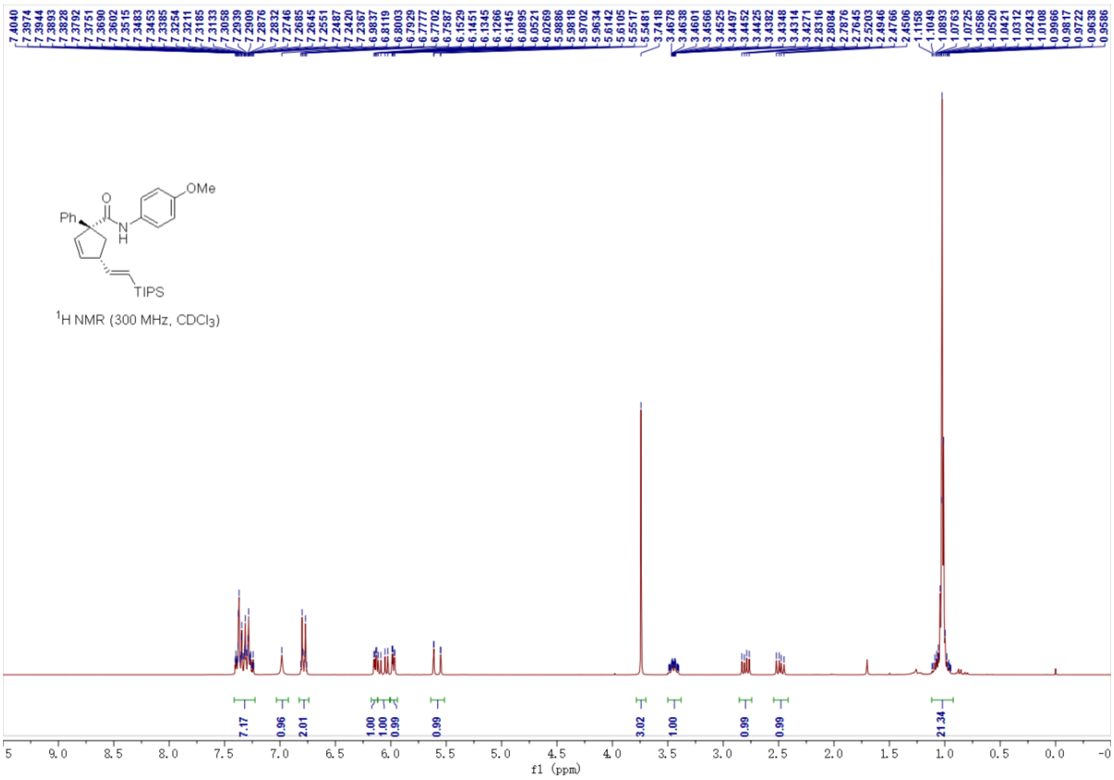

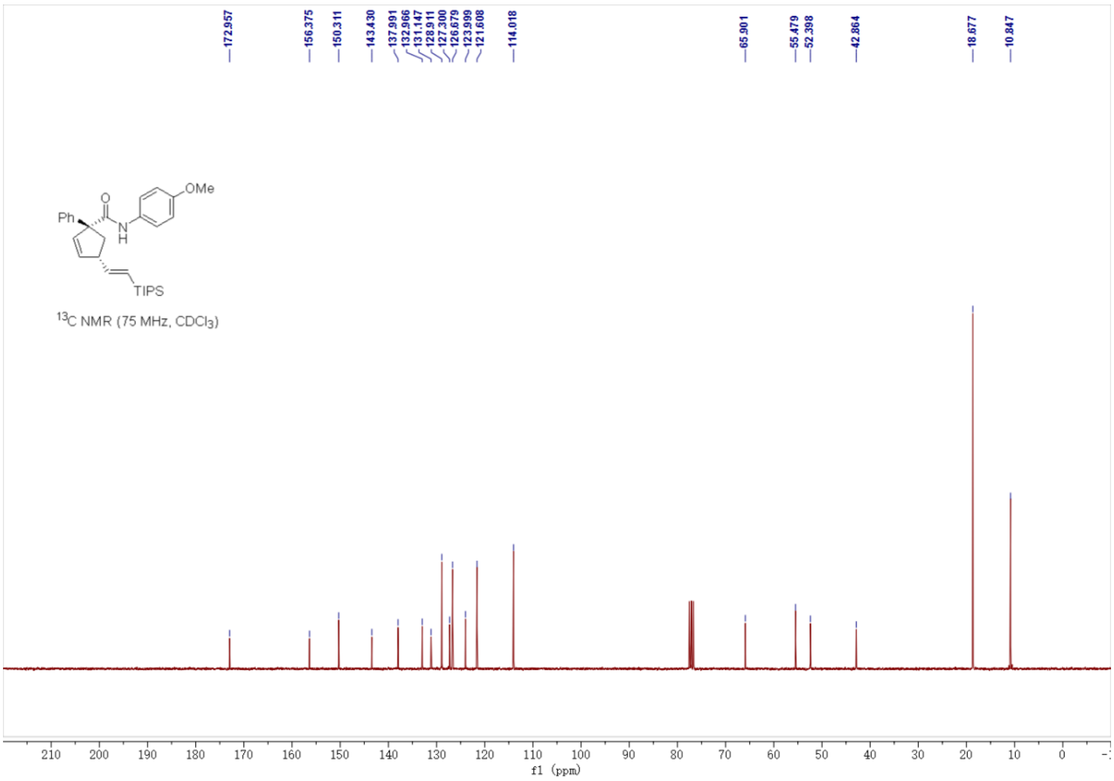

Supplementary Figure 91. ^{1}H and ^{13}C NMR spectra of **3r**

ethyl 4-((1R,4S)-1-phenyl-4-((E)-2-(triisopropylsilyl)vinyl)cyclopent-2-ene-1-carboxamido)benzoate (3s)

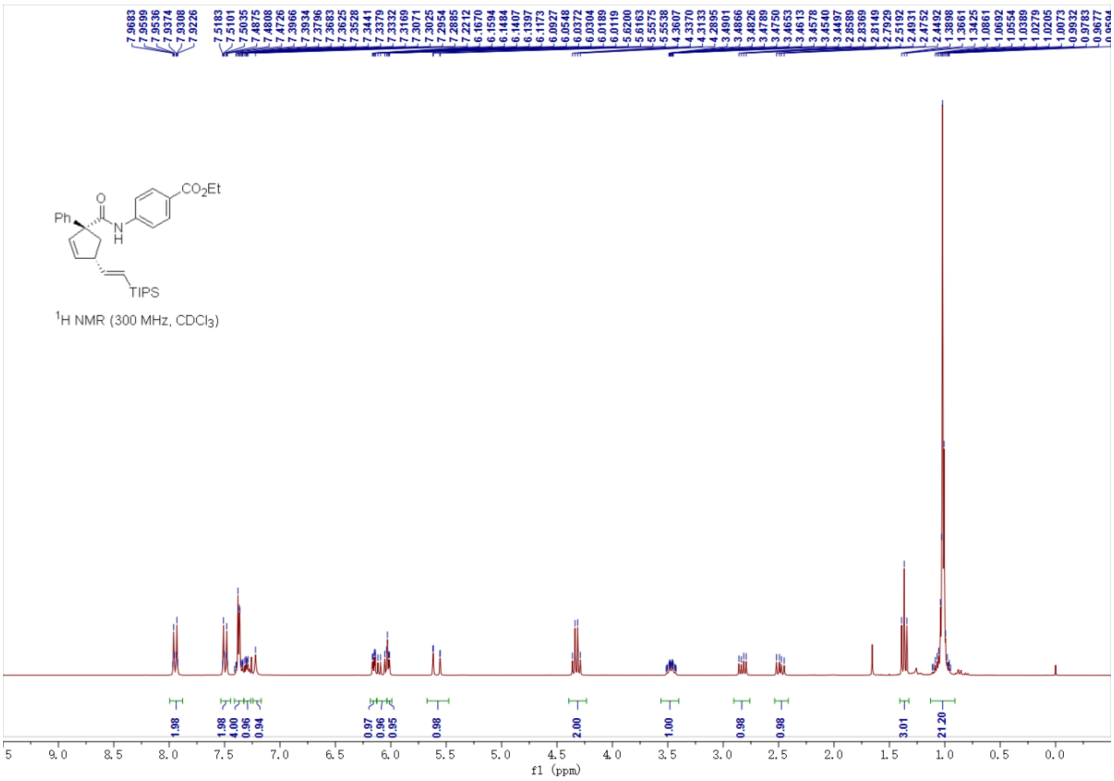

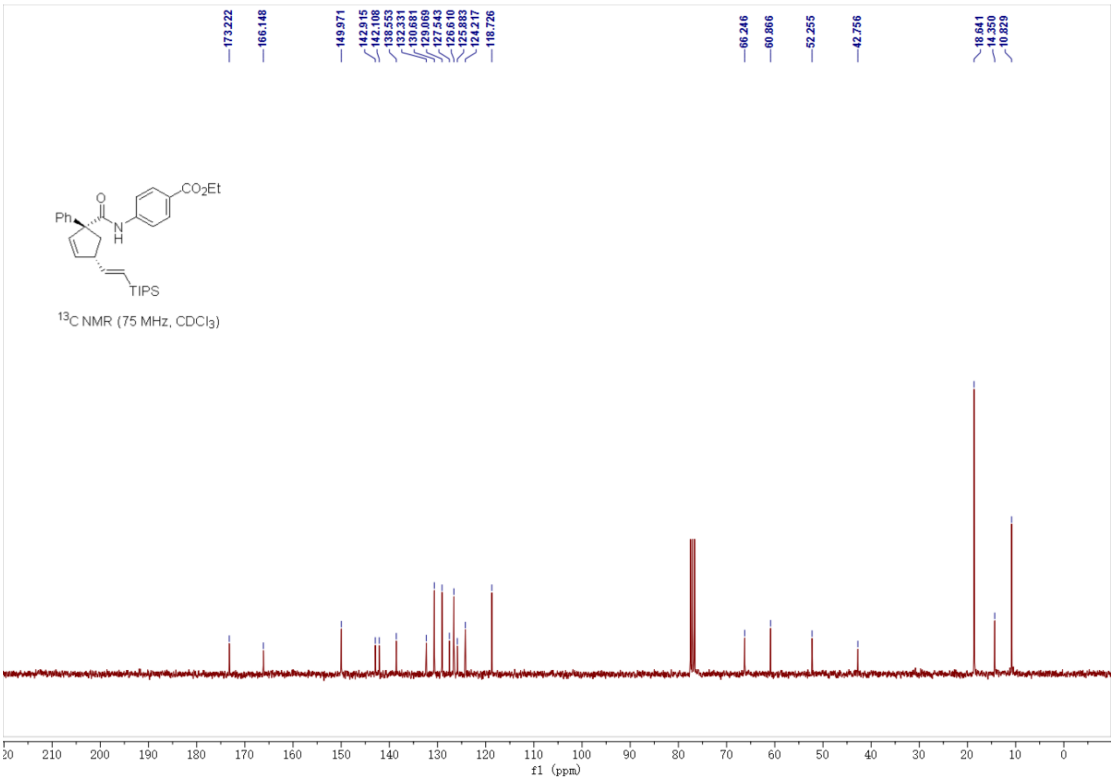

Supplementary Figure 92. ^{1}H and ^{13}C NMR spectra of **3s**

(1R,4S)-N-ethyl-1-phenyl-4-((E)-2-(triisopropylsilyl)vinyl)cyclopent-2-ene-1-carboxamide (3t)

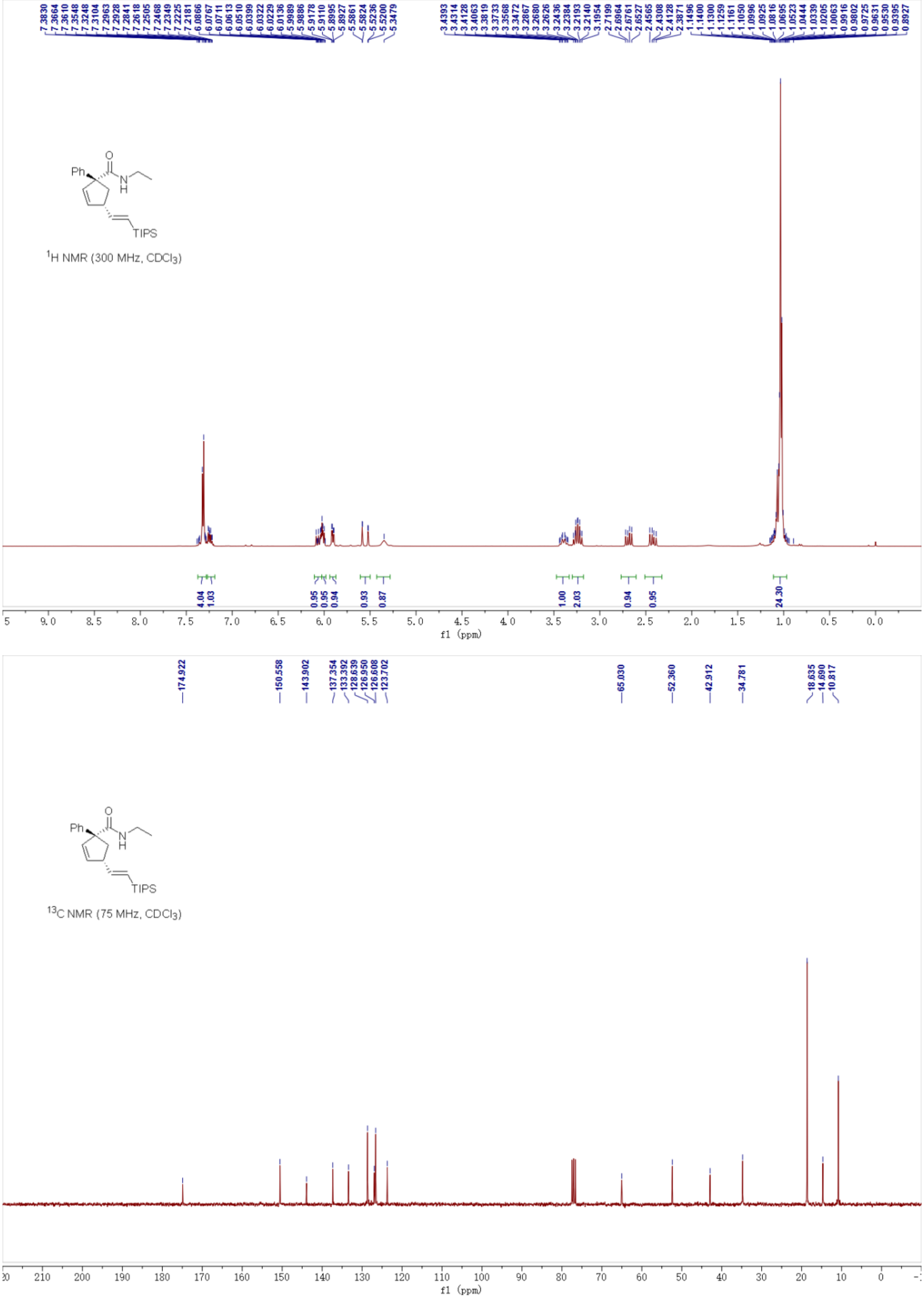

Supplementary Figure 93. ^{1}H and ^{13}C NMR spectra of **3t**

(1R,4S)-N-benzyl-1-phenyl-4-((E)-2-(triisopropylsilyl)vinyl)cyclopent-2-ene-1-carboxamide (3u)

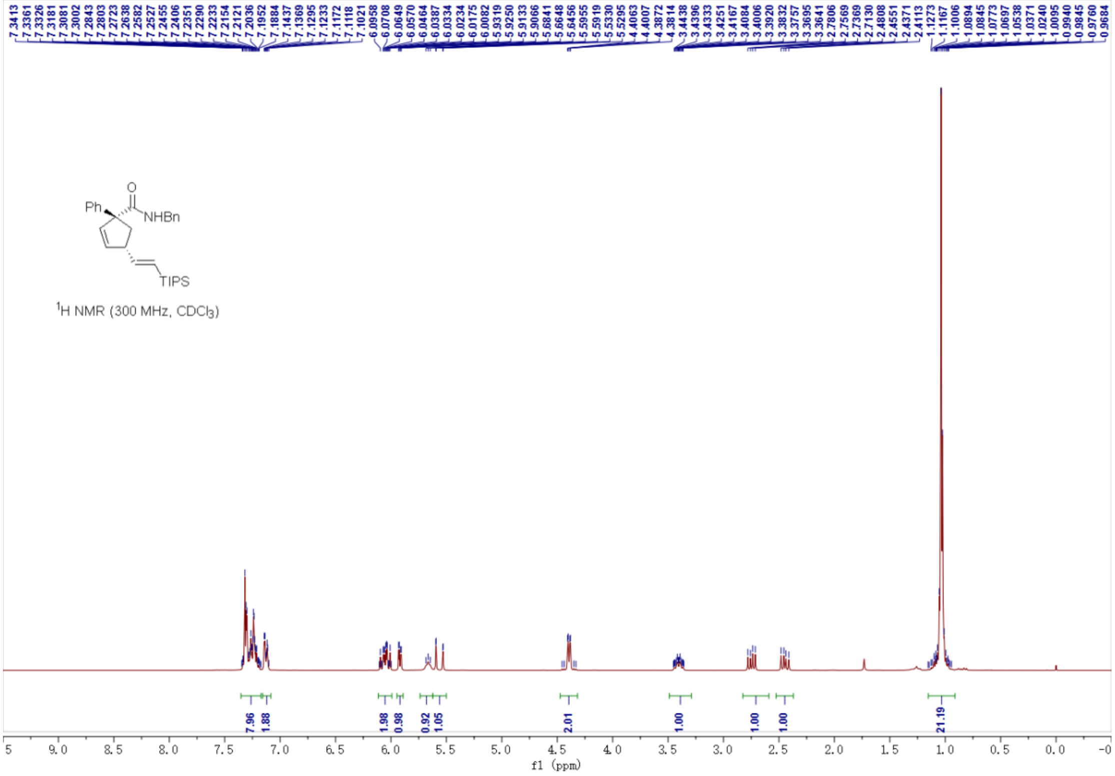

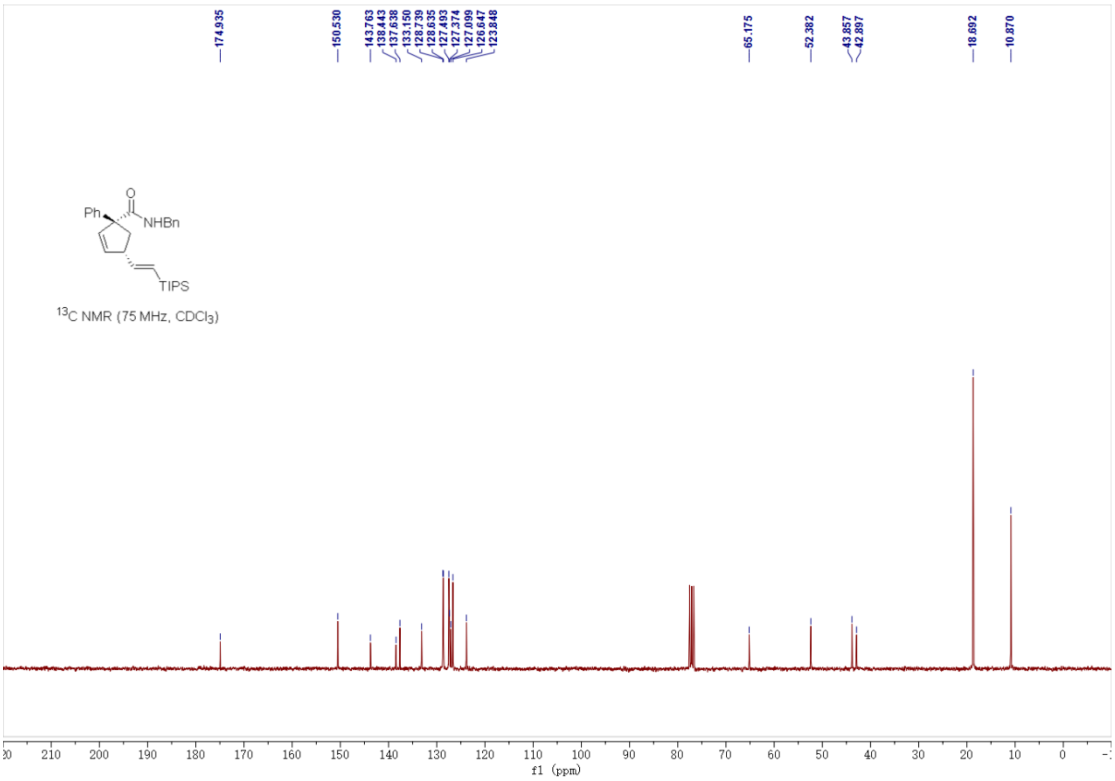

Supplementary Figure 94. ^{1}H and ^{13}C NMR spectra of **3u**

(1R,4S)-N-cyclohexyl-1-phenyl-4-((E)-2-(triisopropylsilyl)vinyl)cyclopent-2-ene-1-carboxamide (3v)

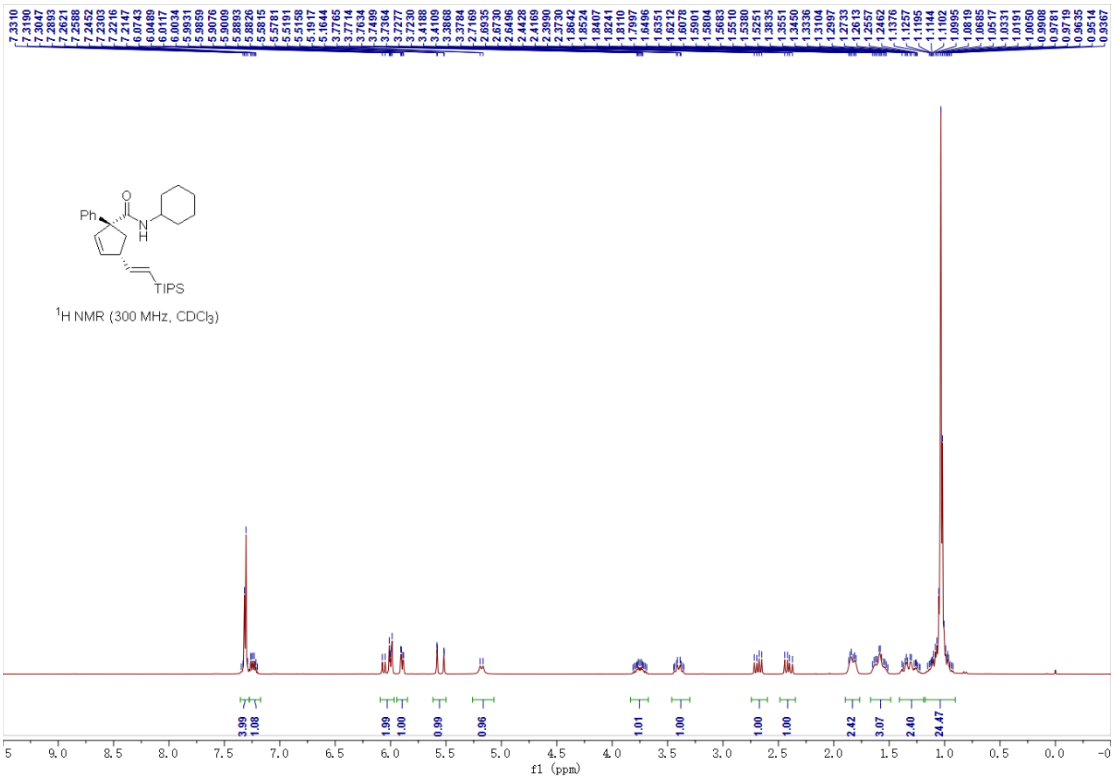

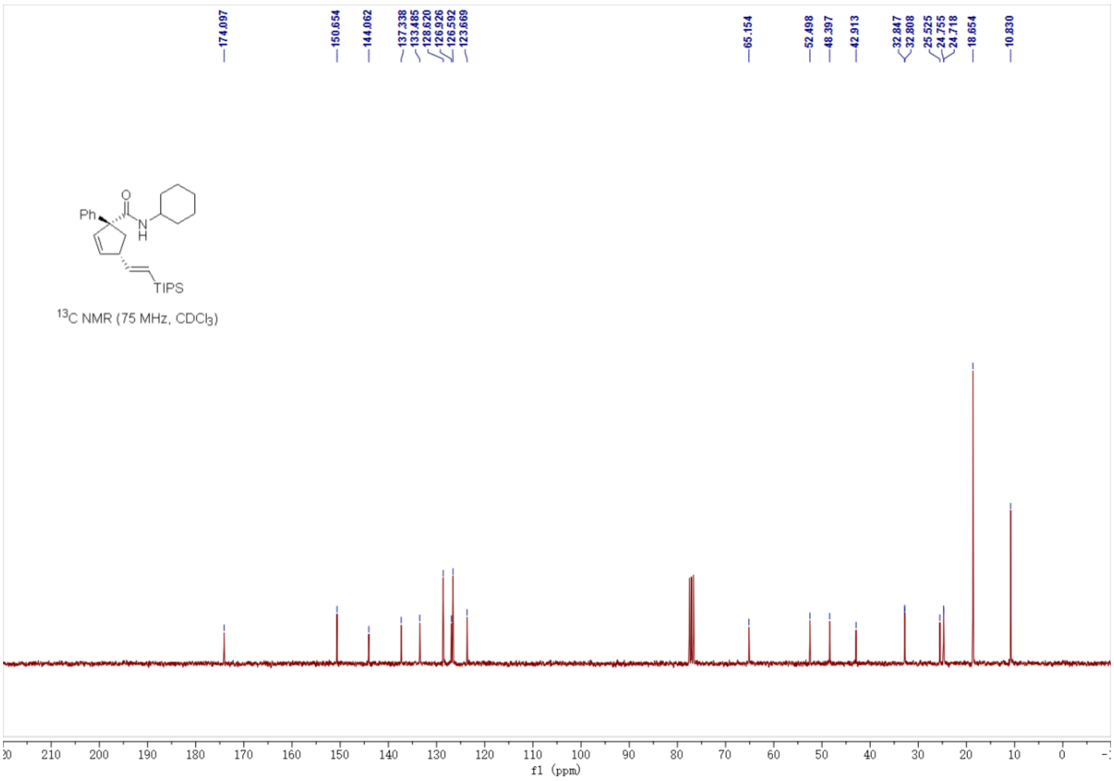

Supplementary Figure 95. ^{1}H and ^{13}C NMR spectra of **3v**

(1R,4S)-N-(tert-butyl)-1-phenyl-4-((E)-2-(triisopropylsilyl)vinyl)cyclopent-2-ene-1-carboxamide (3w)

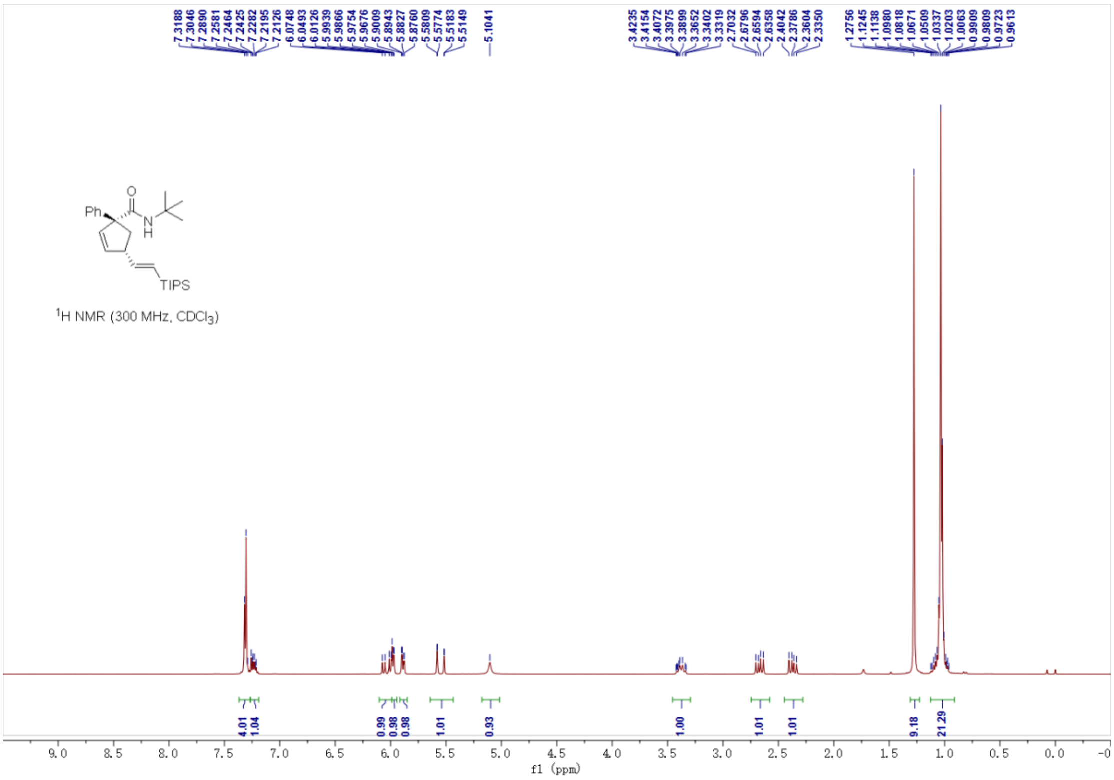

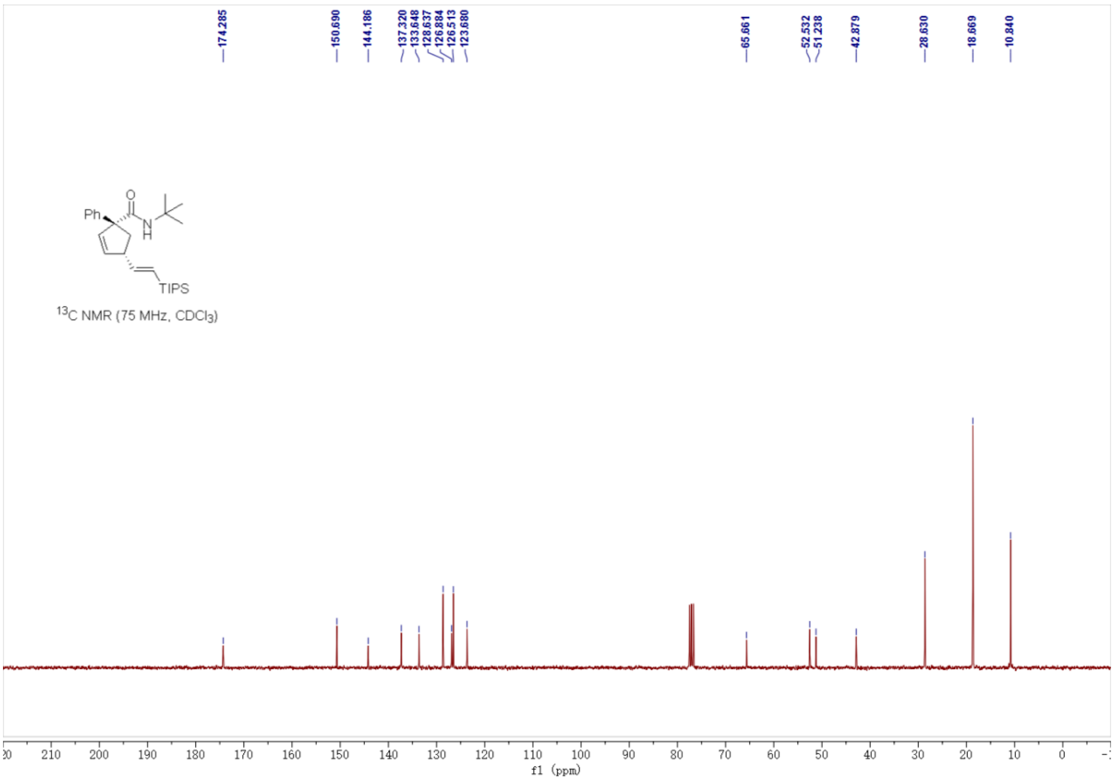

Supplementary Figure 96. ^{1}H and ^{13}C NMR spectra of **3w**

(1R,4S)-N-methyl-N,1-diphenyl-4-((E)-2-(triisopropylsilyl)vinyl)cyclopent-2-ene-1-carboxamide (3x)

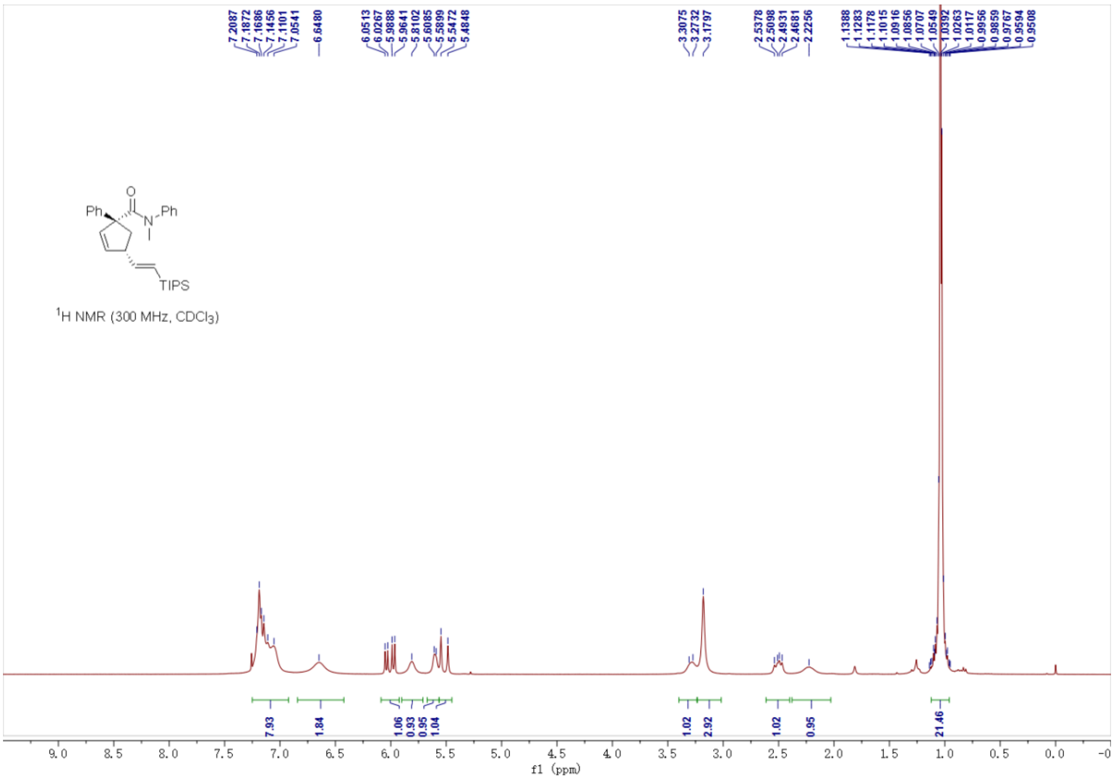

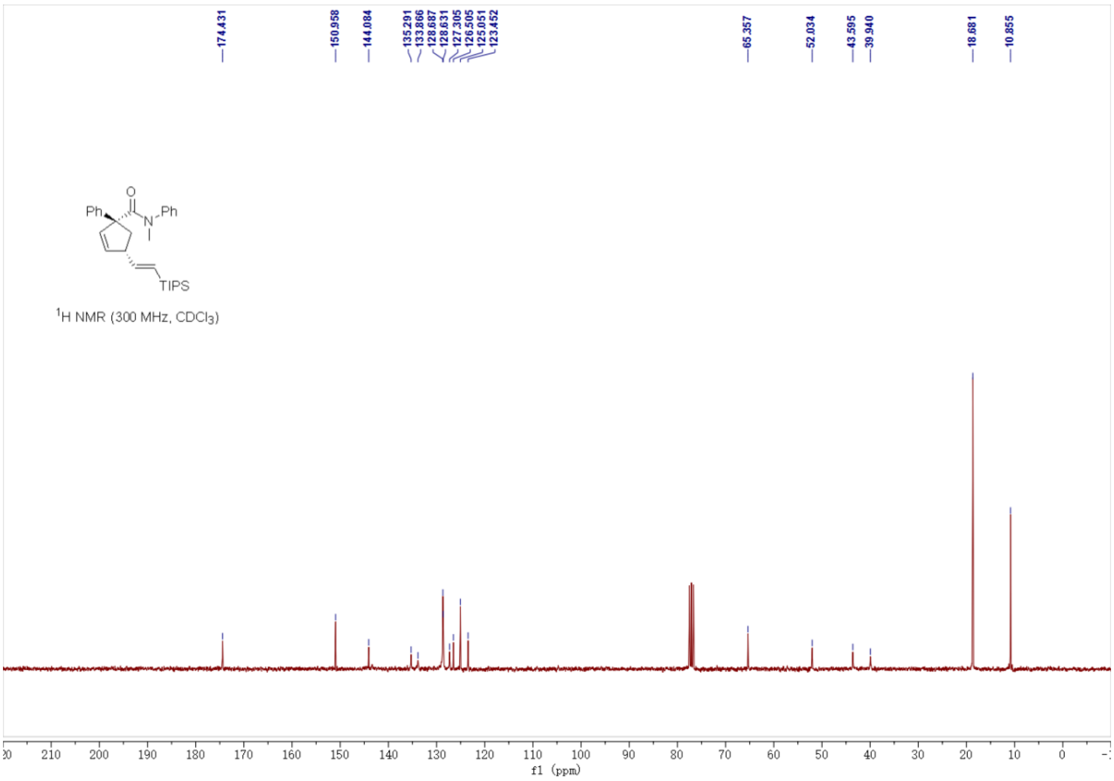

Supplementary Figure 97. ^{1}H and ^{13}C NMR spectra of **3x**

(1R,4S)-4-((E)-2-(triisopropylsilyl)vinyl)spiro[cyclopentane-1,3'-indolin]-2-en-2'-one (3y)

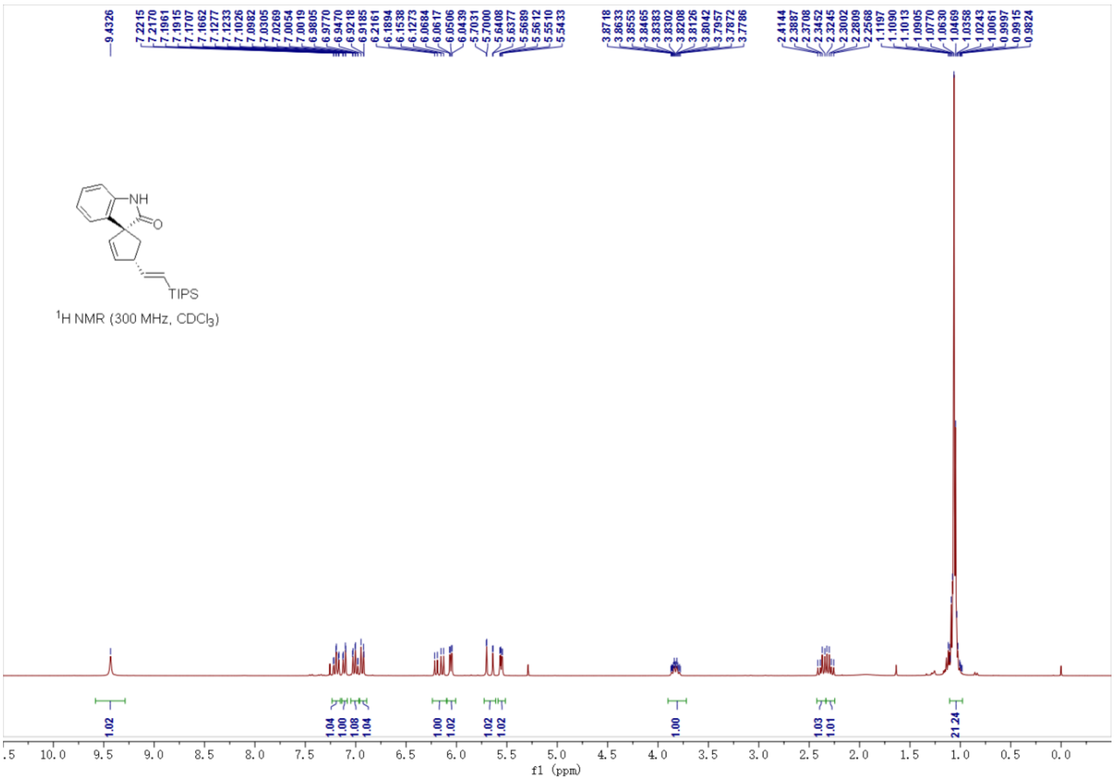

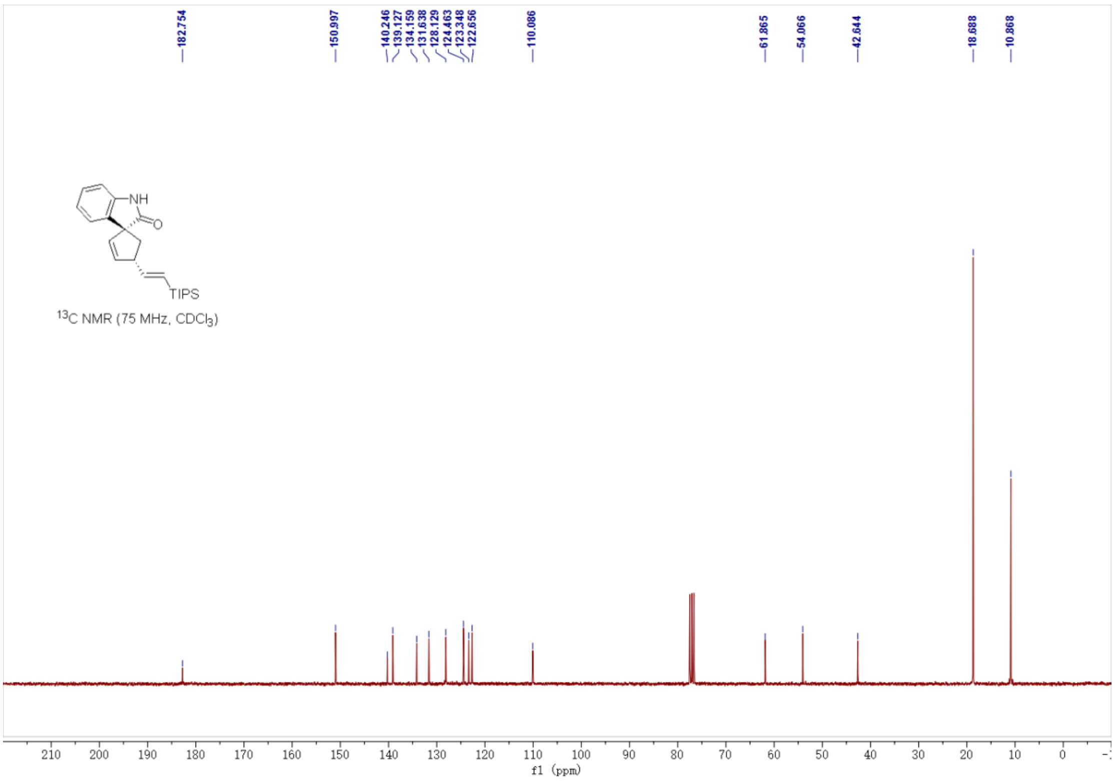

Supplementary Figure 98. ^{1}H and ^{13}C NMR spectra of **3y**

phenyl (1R,4S)-1-phenyl-4-((E)-2-(triisopropylsilyl)vinyl)cyclopent-2-ene-1-carboxylate (3z)

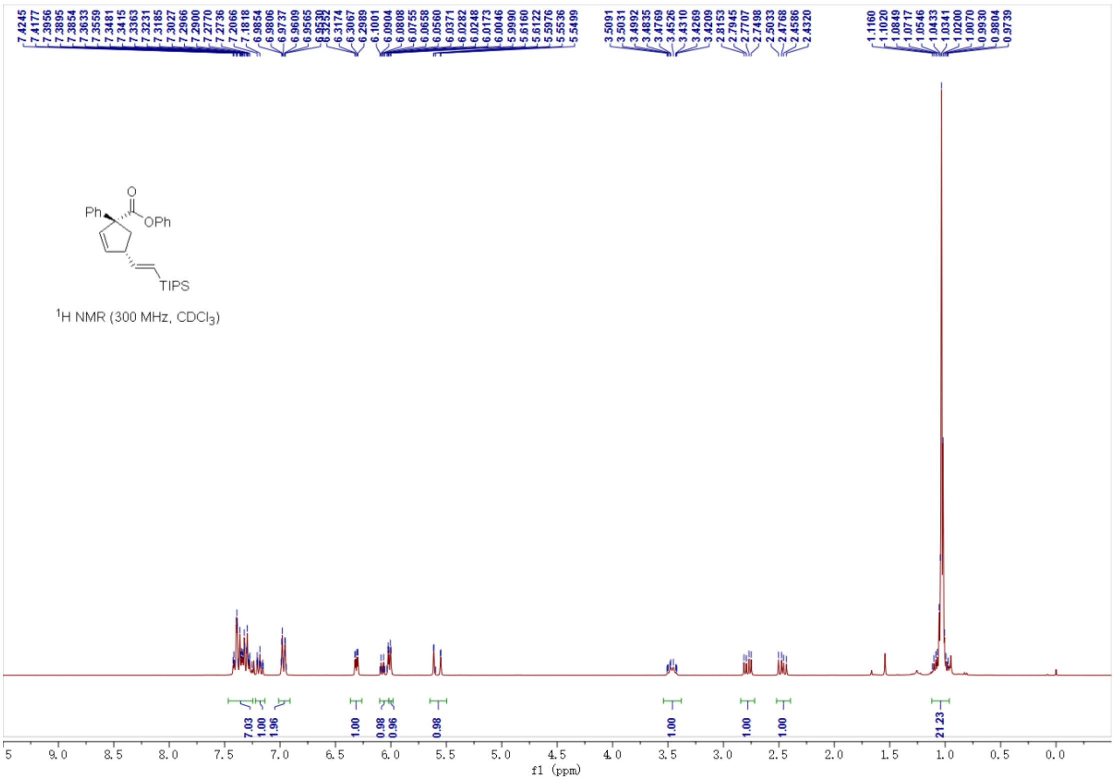

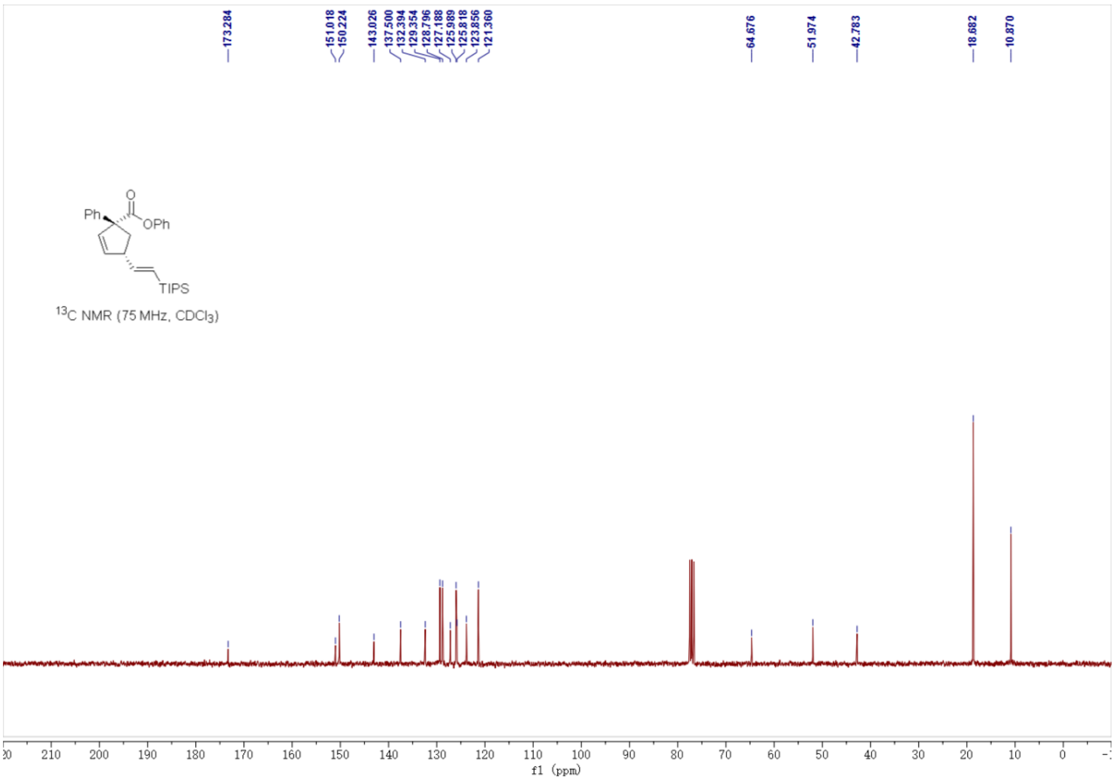

Supplementary Figure 99. ^{1}H and ^{13}C NMR spectra of **3z**

ethyl (1R,4S)-1-phenyl-4-((E)-2-(triisopropylsilyl)vinyl)cyclopent-2-ene-1-carboxylate (3aa)

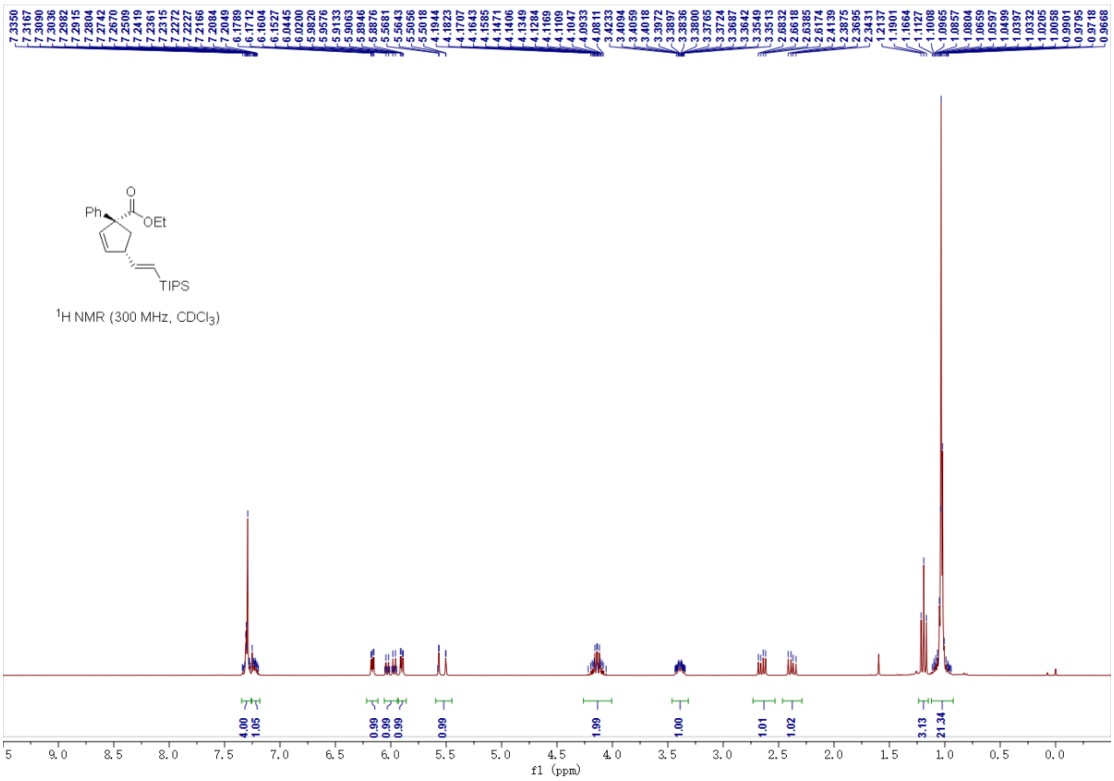

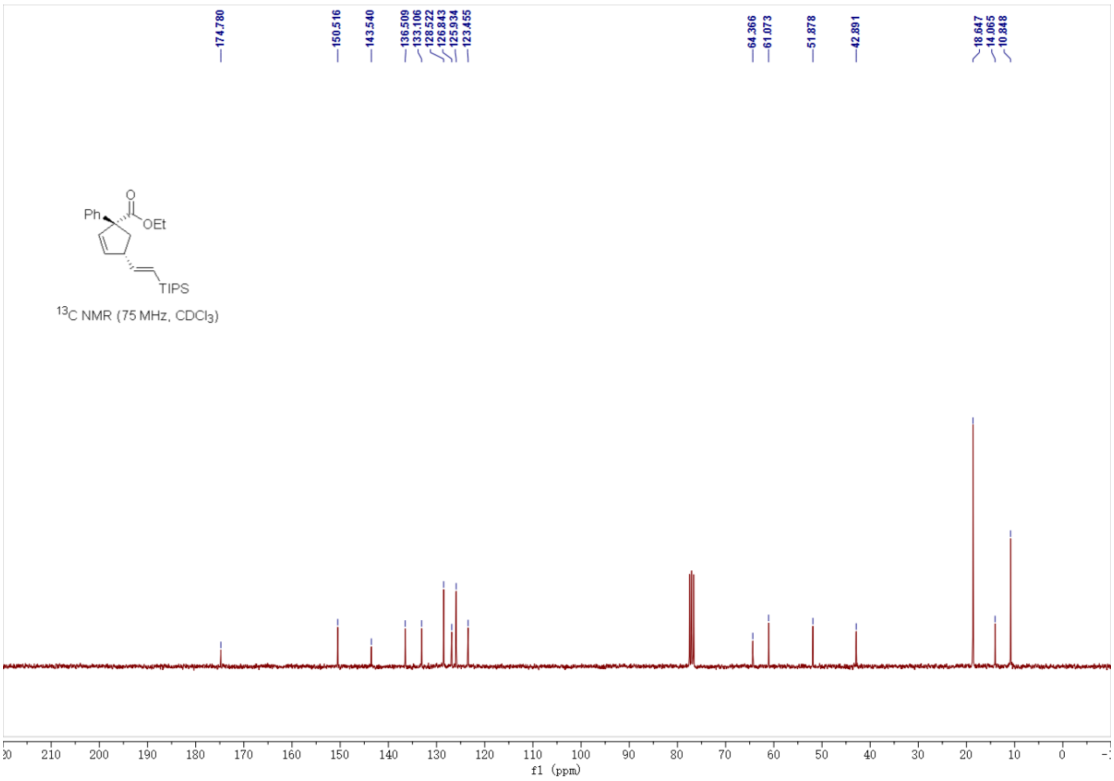

Supplementary Figure 100. ^{1}H and ^{13}C NMR spectra of **3aa**

phenyl((1R,4S)-1-phenyl-4-((E)-2-(triisopropylsilyl)vinyl)cyclopent-2-en-1-yl)methanone (3ab)

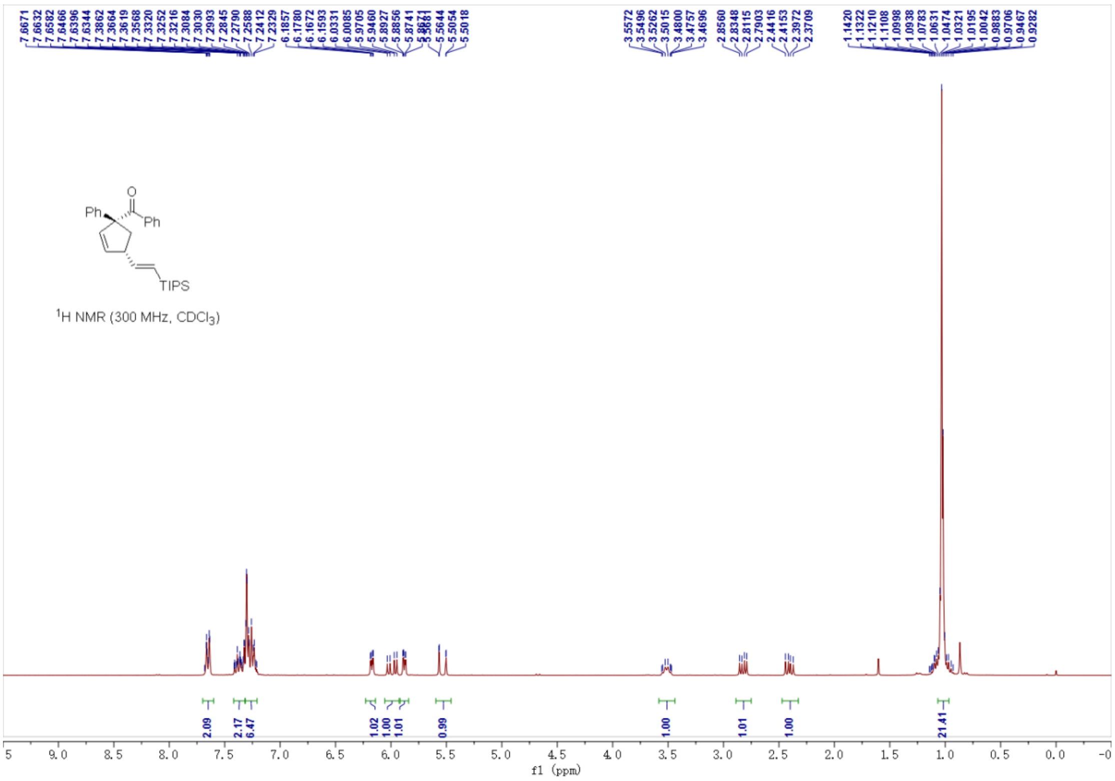

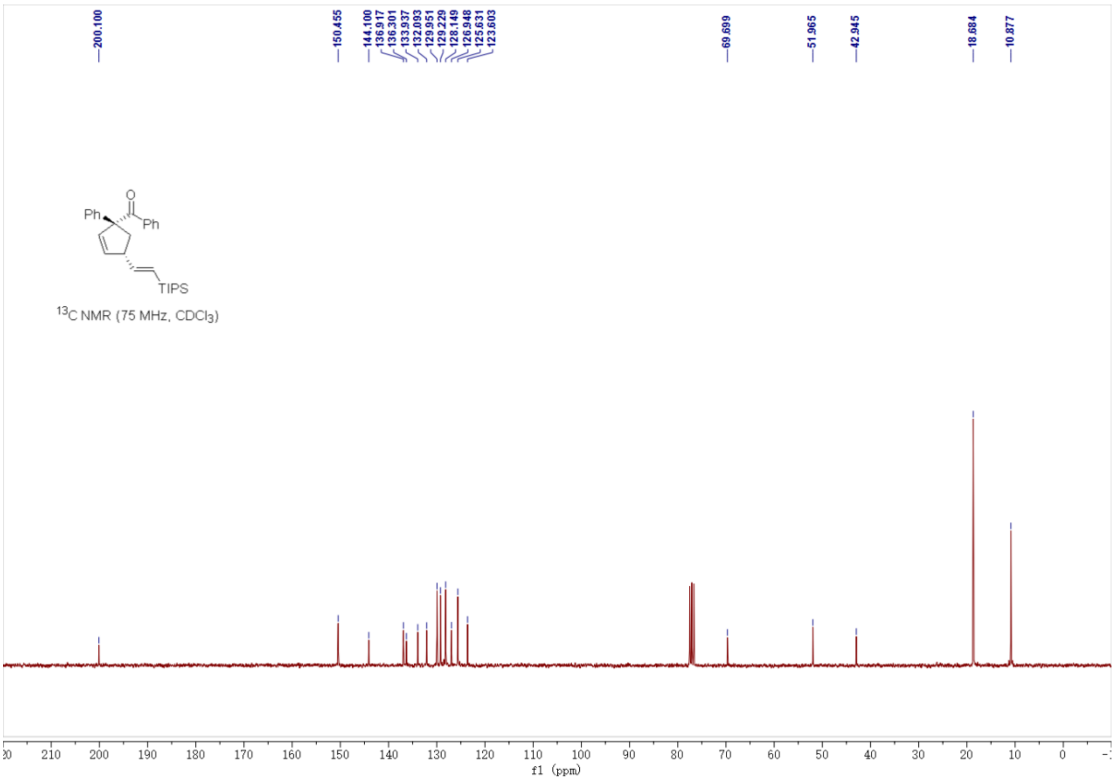

Supplementary Figure 101. ^{1}H and ^{13}C NMR spectra of **3ab**

2-methyl-1-((1R,4S)-1-phenyl-4-((E)-2-(triisopropylsilyl)vinyl)cyclopent-2-en-1-yl)propan-1-one (3ac)

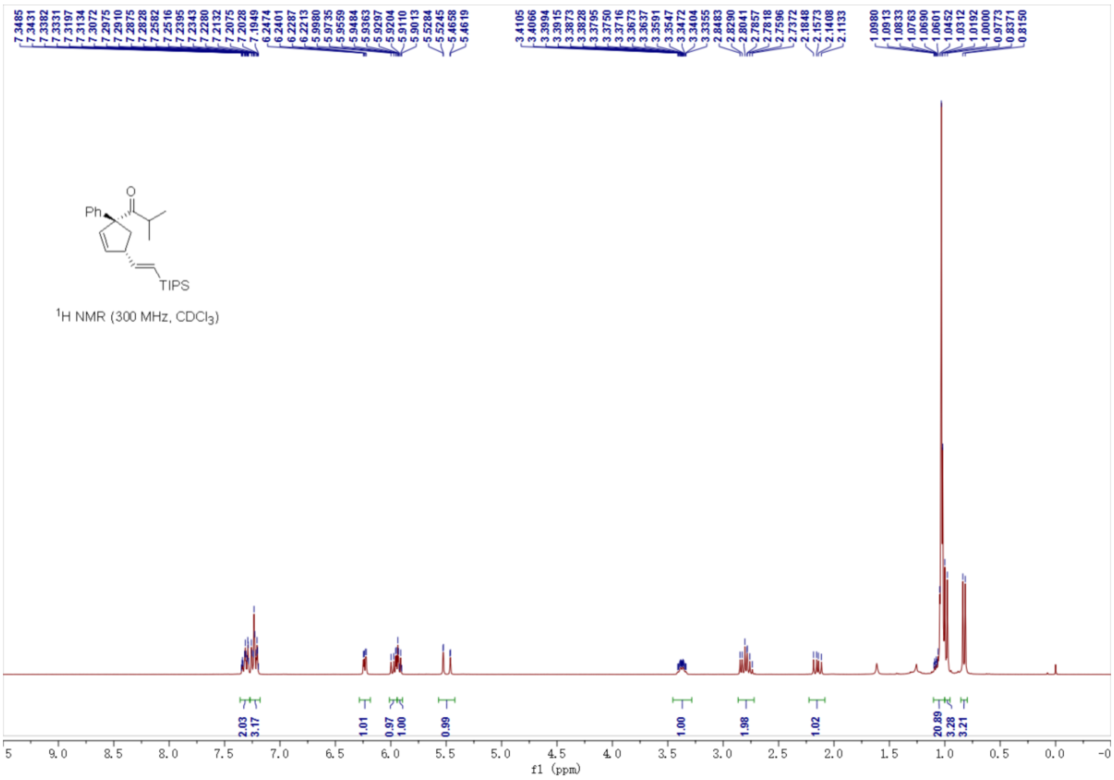

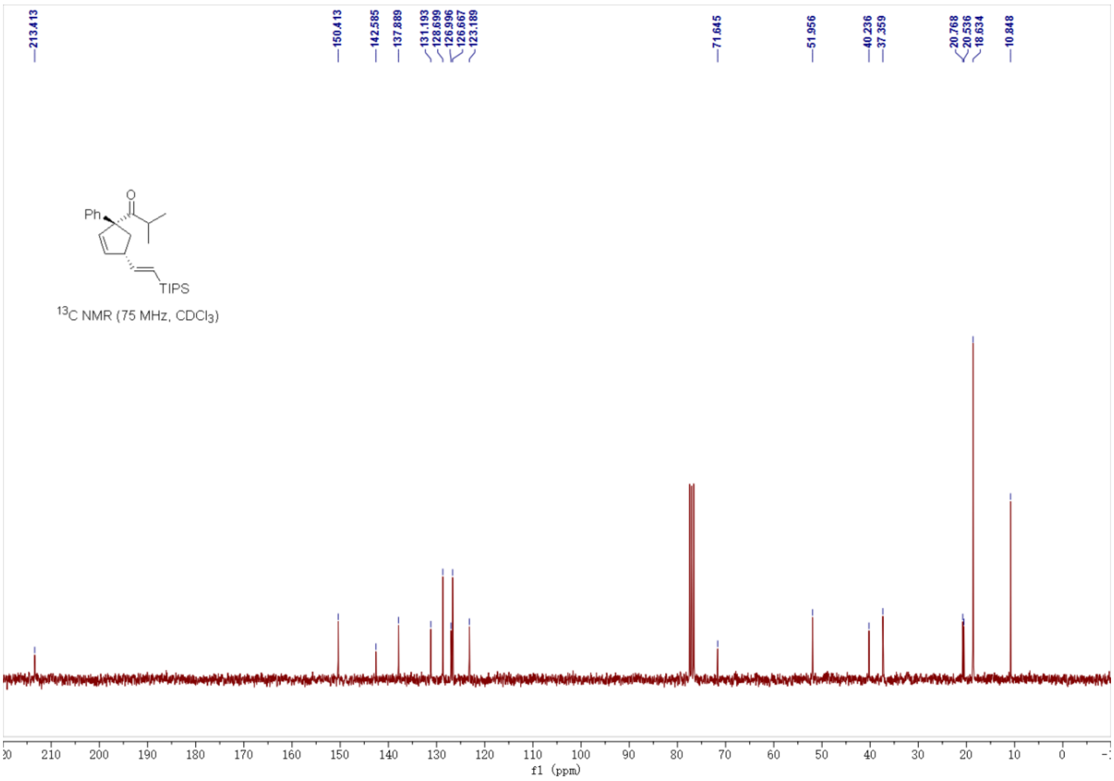

Supplementary Figure 102. ^{1}H and ^{13}C NMR spectra of **3ac**

1-((1R,4S)-1-phenyl-4-((E)-2-(triisopropylsilyl)vinyl)cyclopent-2-en-1-yl)ethan-1-one (3ad)

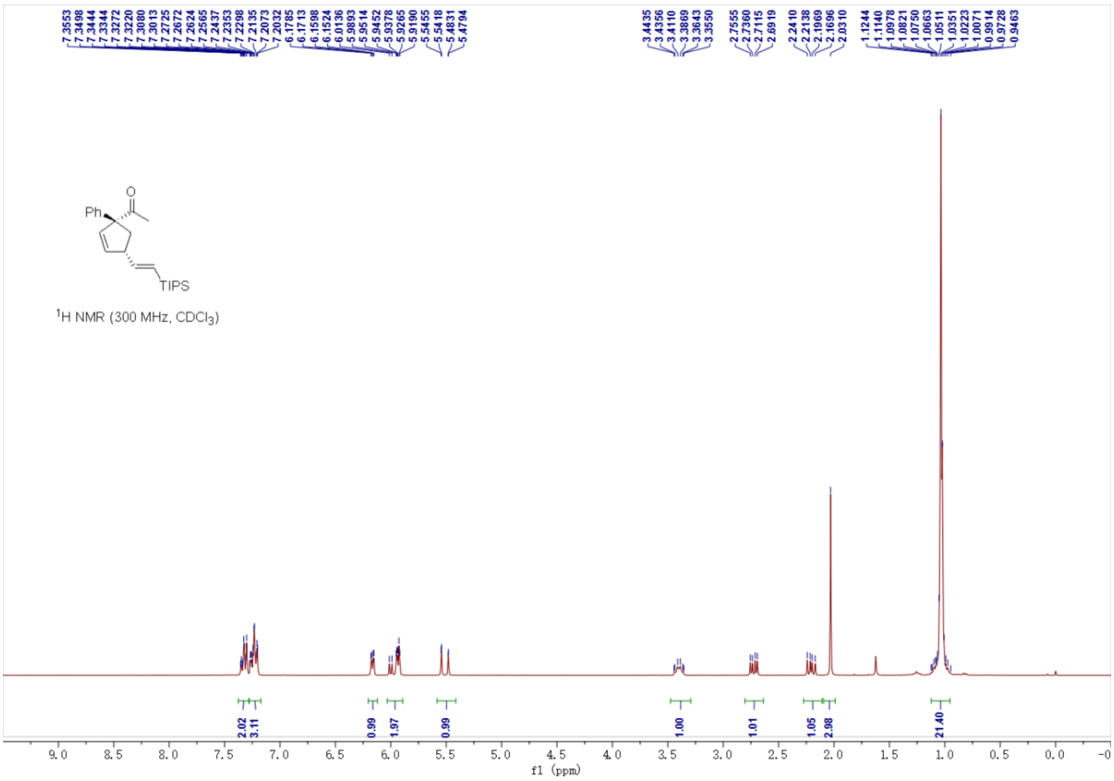

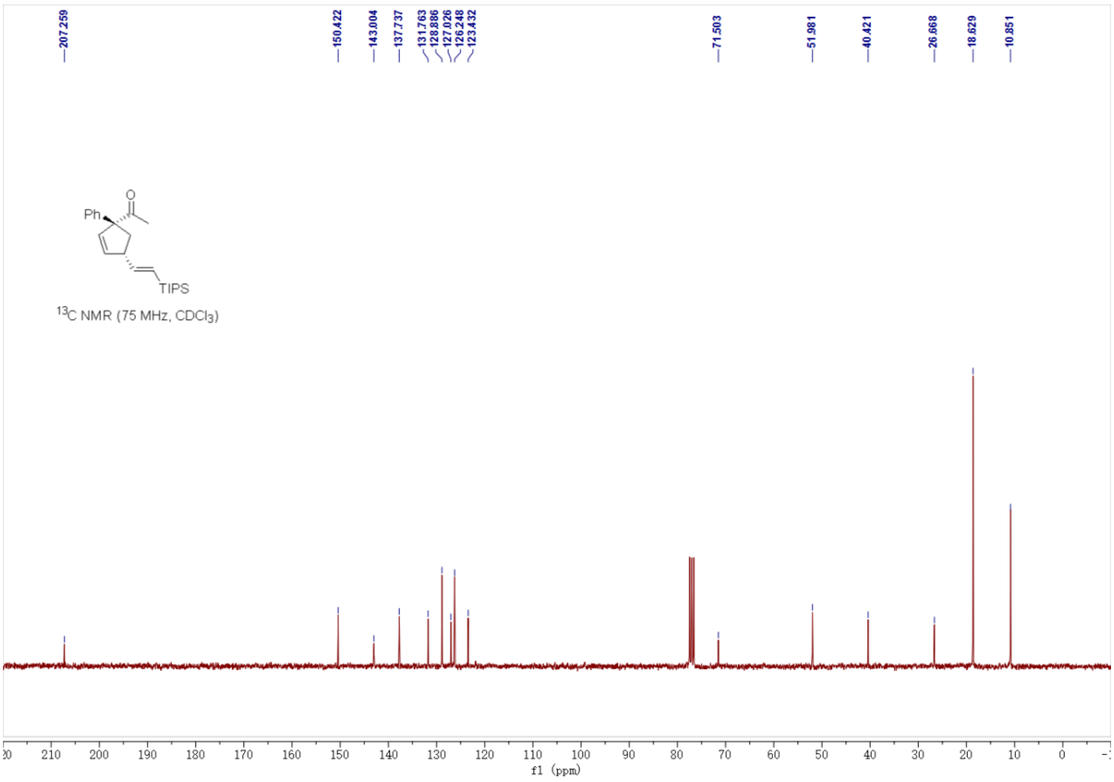

Supplementary Figure 103. ^{1}H and ^{13}C NMR spectra of **3ad**

(1R,4S)-N,1-diphenyl-4-((E)-2-(trimethylsilyl)vinyl)cyclopent-2-ene-1-carboxamide (3af)

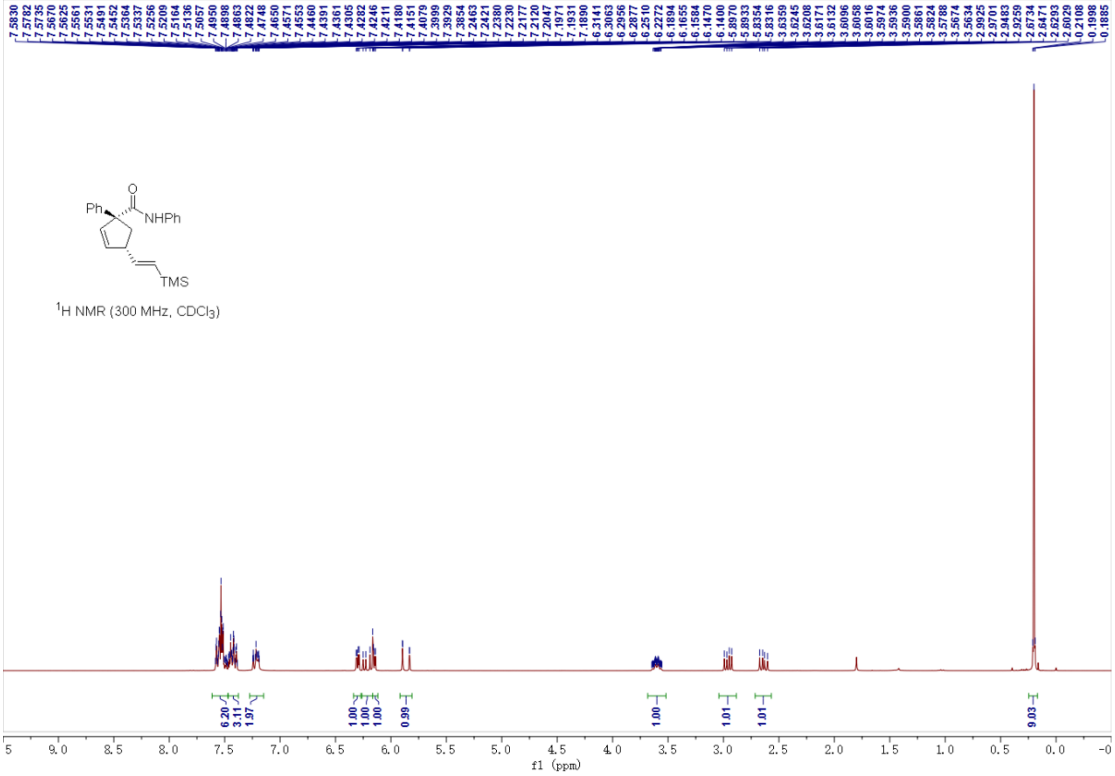

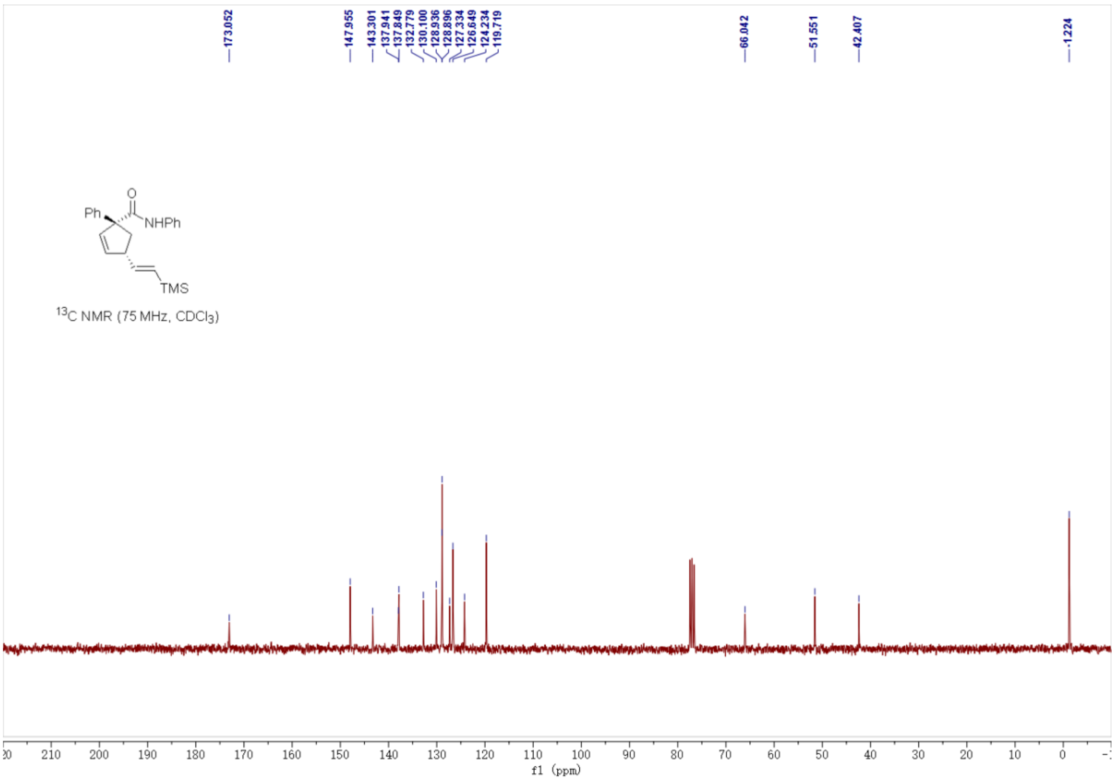

Supplementary Figure 104. ^{1}H and ^{13}C NMR spectra of **3af**

(1R,4S)-4-((E)-2-(tert-butyldimethylsilyl)vinyl)-N,1-diphenylcyclopent-2-ene-1-carboxamide (3ag)

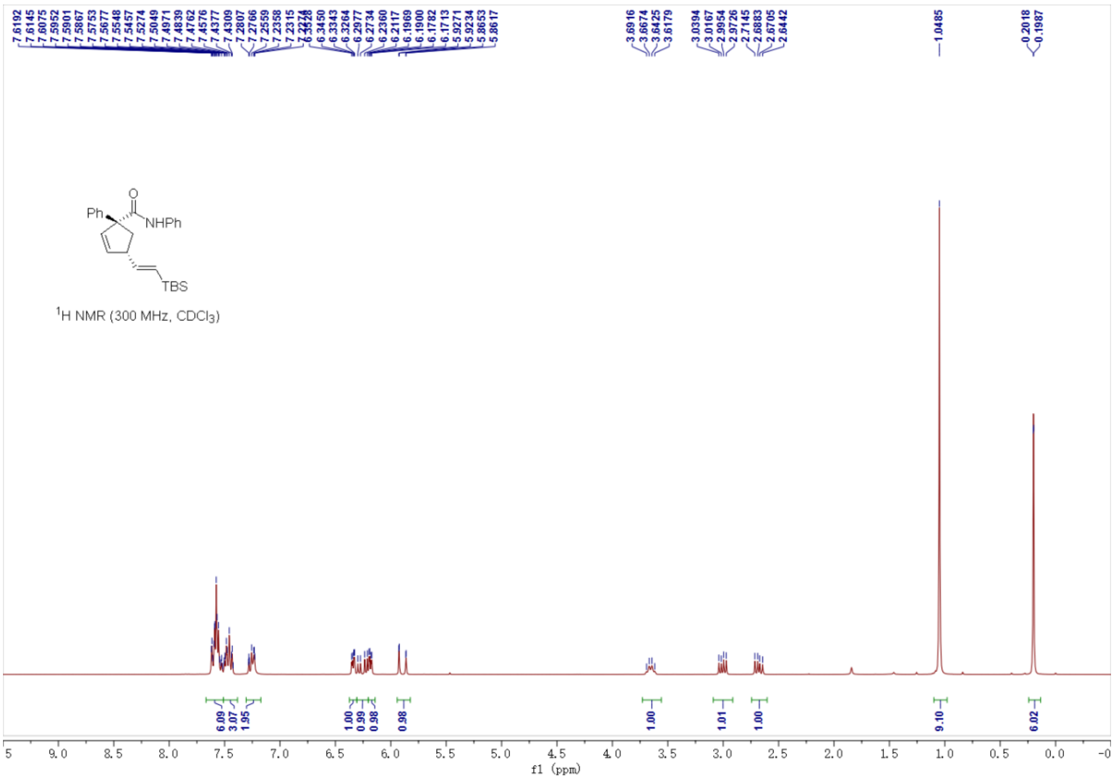

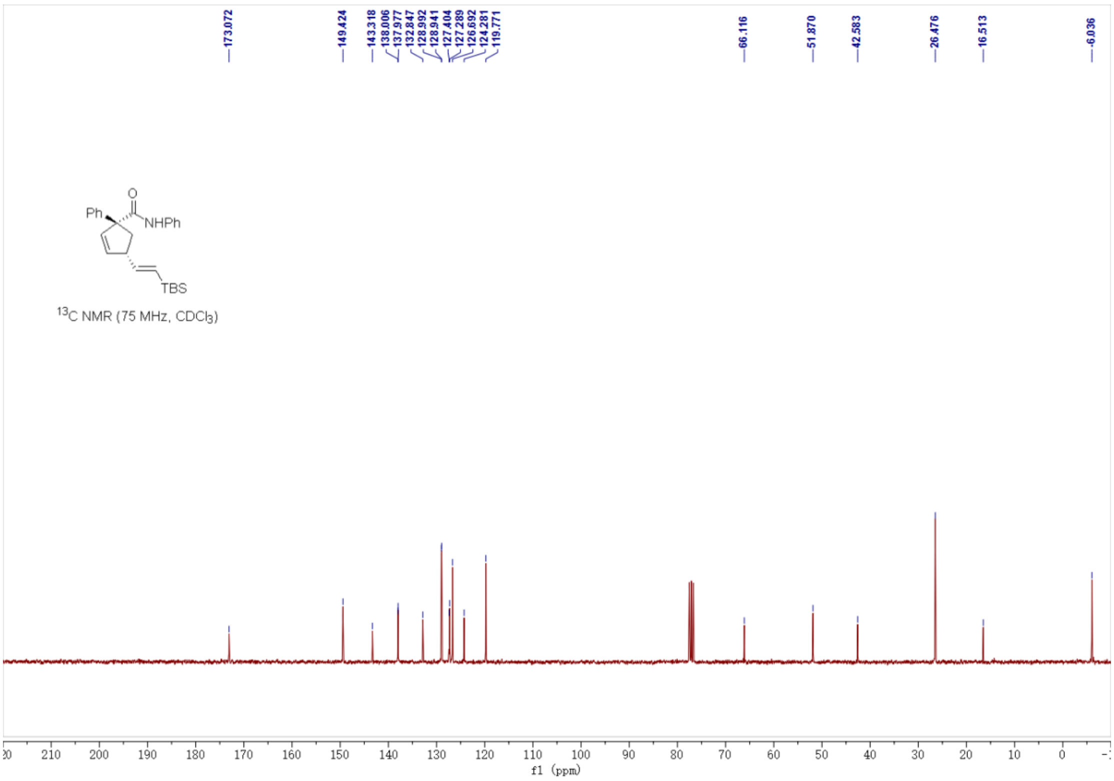

Supplementary Figure 105. ^{1}H and ^{13}C NMR spectra of **3ag**

(1R,4S)-N,1-diphenyl-4-((E)-2-(triethylsilyl)vinyl)cyclopent-2-ene-1-carboxamide (3ah)

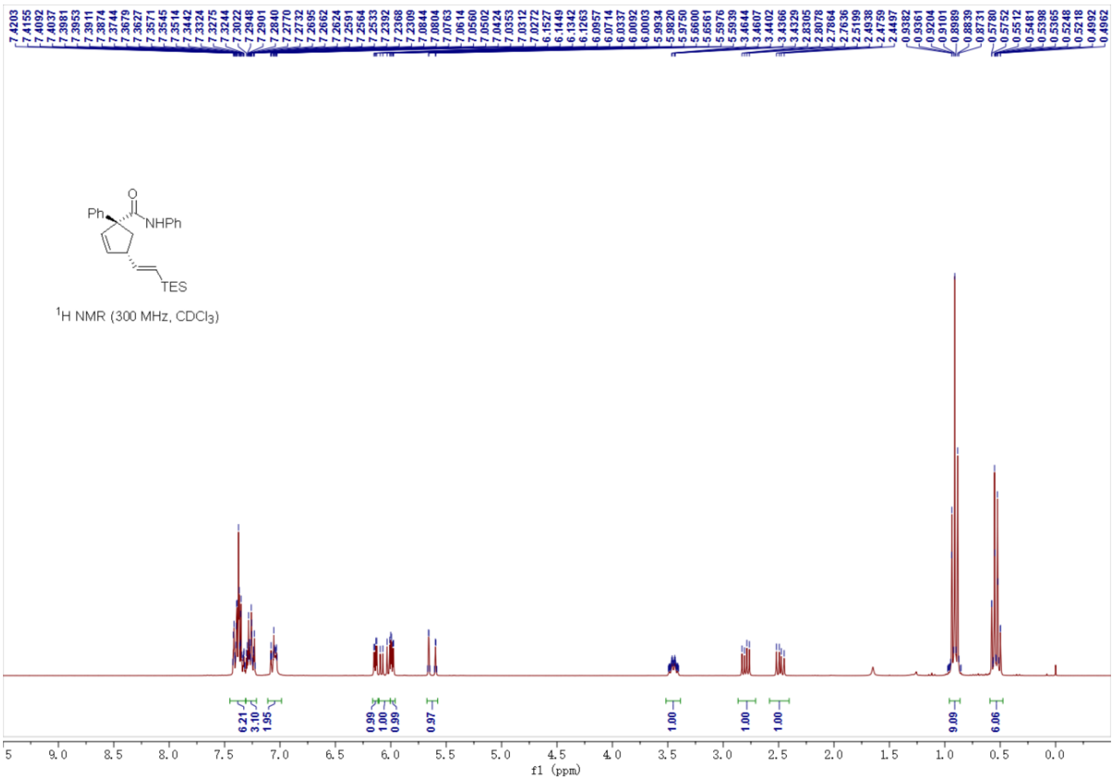

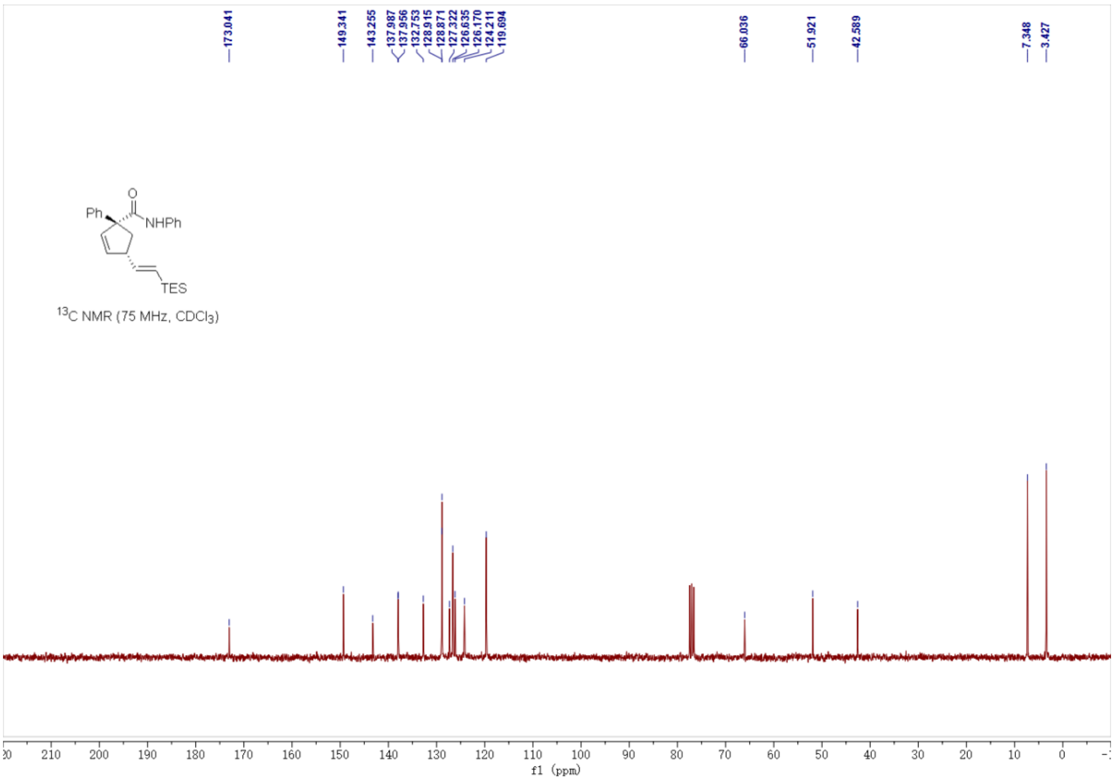

Supplementary Figure 106. ^{1}H and ^{13}C NMR spectra of **3ah**

(1R,4S)-4-((E)-2-(benzyldimethylsilyl)vinyl)-N,1-diphenylcyclopent-2-ene-1-carboxamide (3ai)

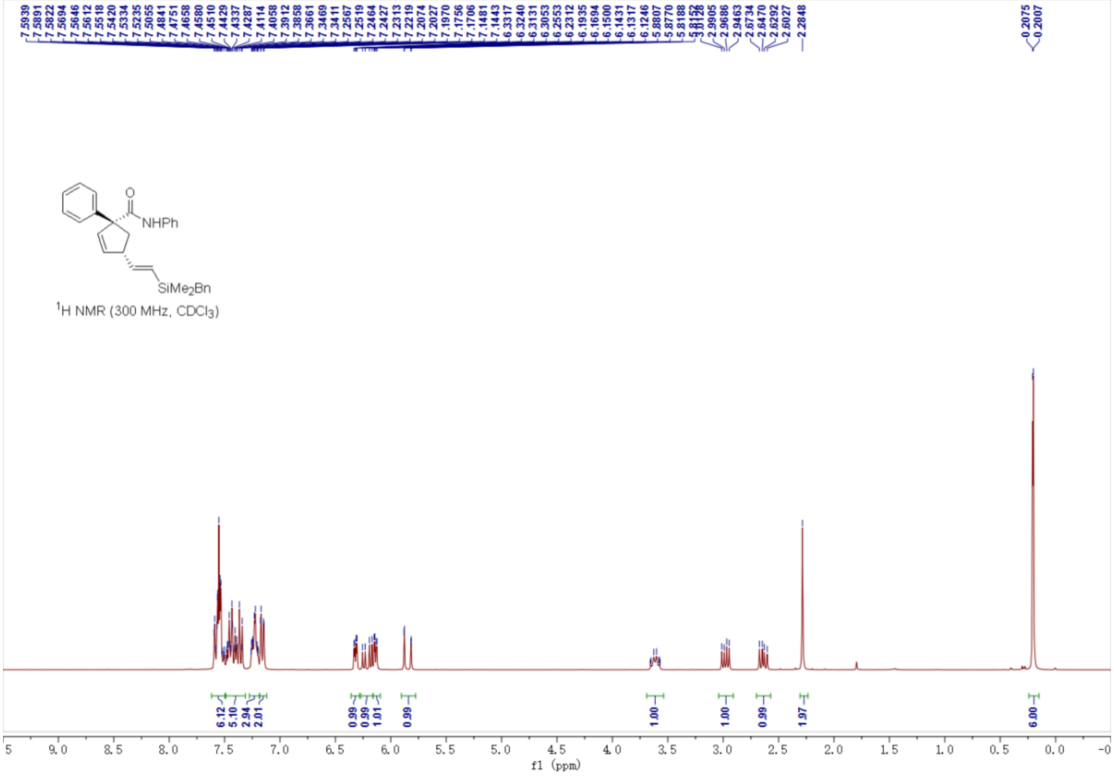

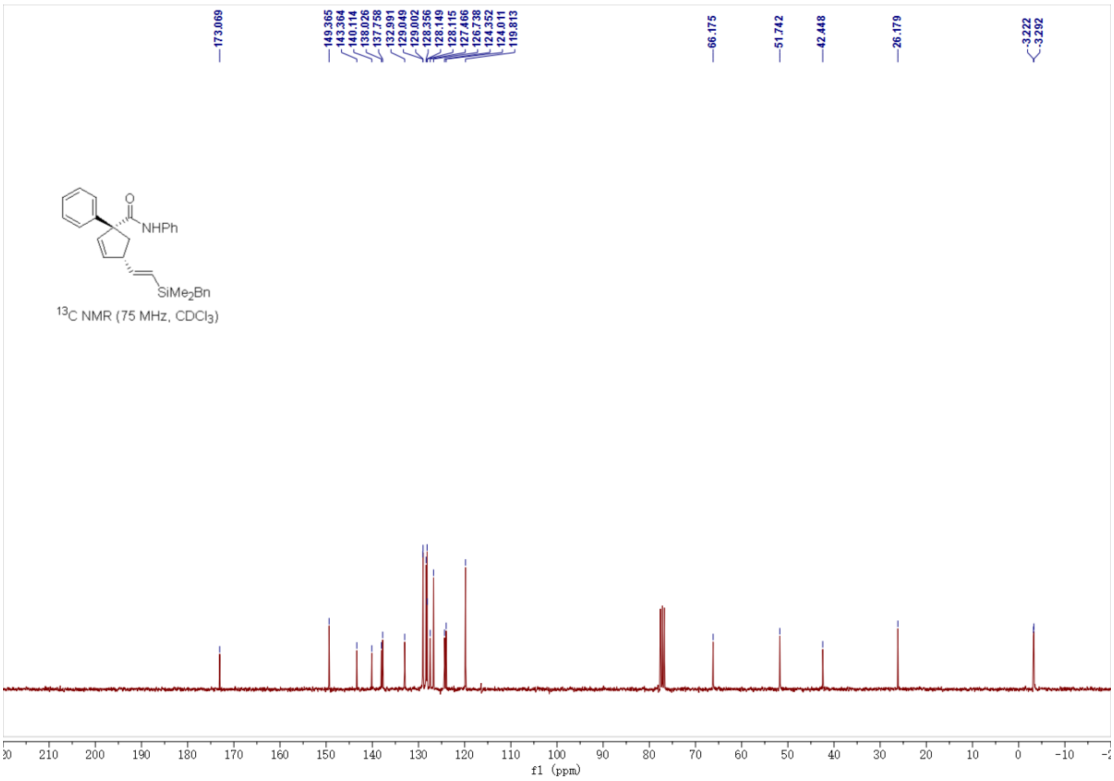

Supplementary Figure 107. ^{1}H and ^{13}C NMR spectra of **3ai**

(1R,4R)-4-((E)-3,3-dimethylbut-1-en-1-yl)-N,1-diphenylcyclopent-2-ene-1-carboxamide (3aj)

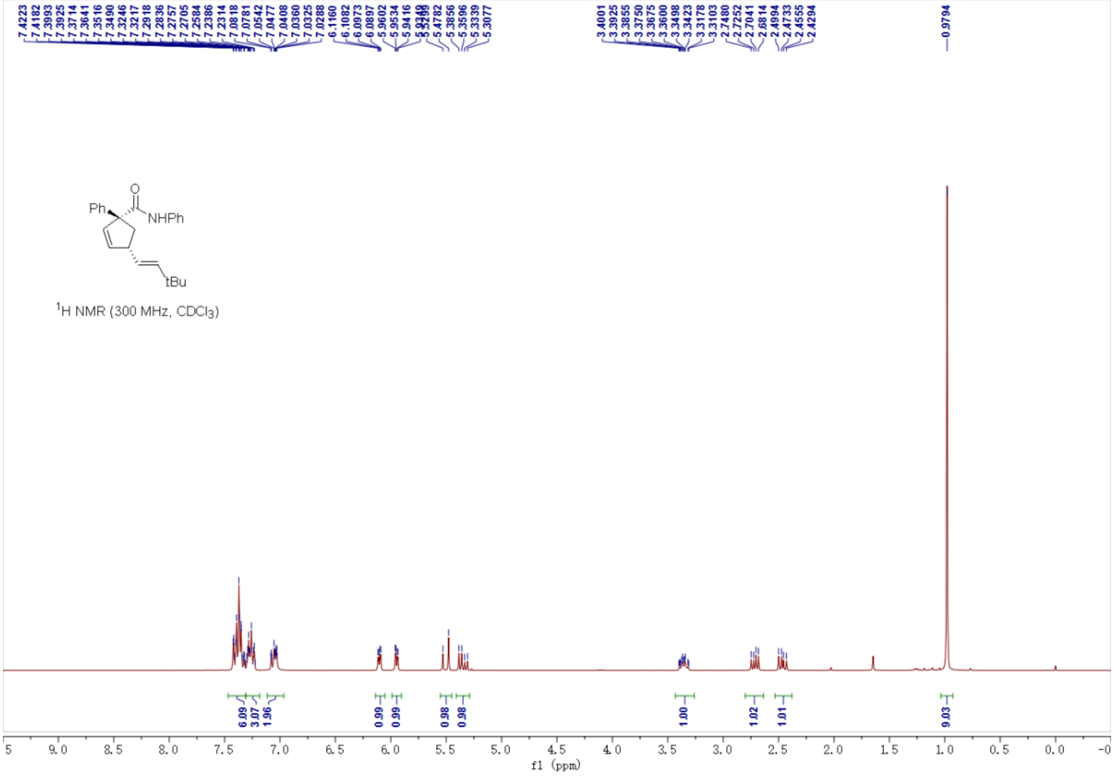

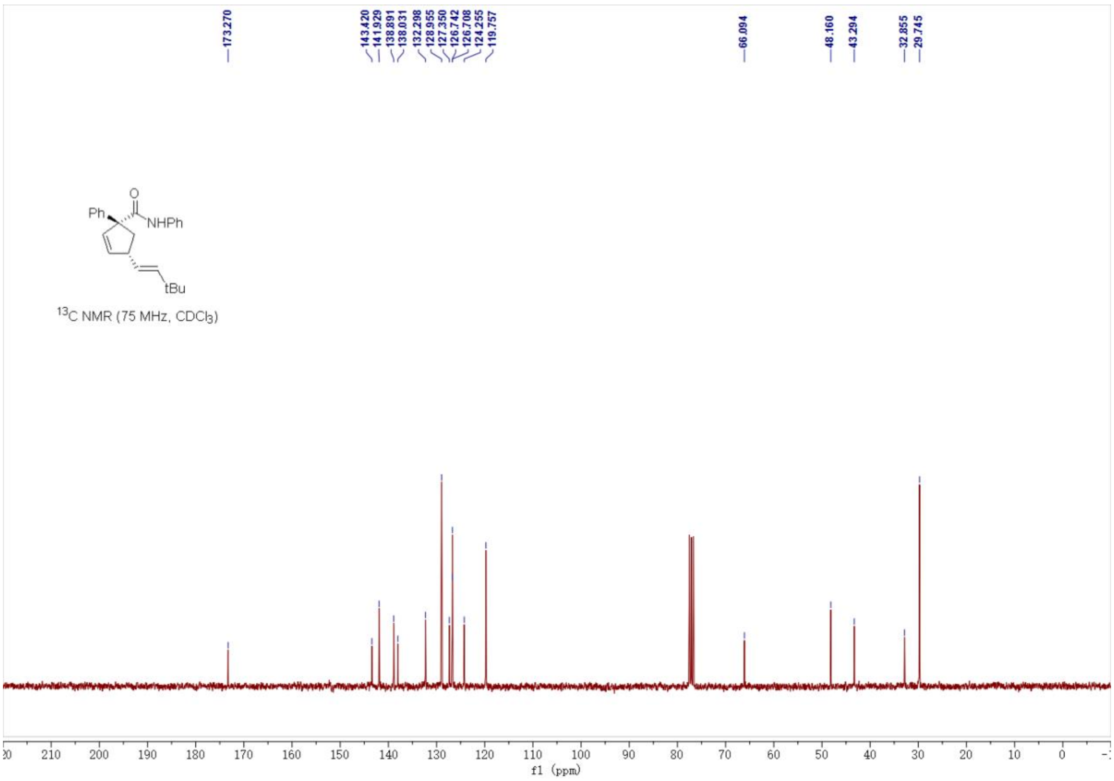

Supplementary Figure 108. ^{1}H and ^{13}C NMR spectra of **3aj**

(1R,4S)-4-(1-(4-methoxyphenyl)vinyl)-N,1-diphenylcyclopent-2-ene-1-carboxamide (3al')

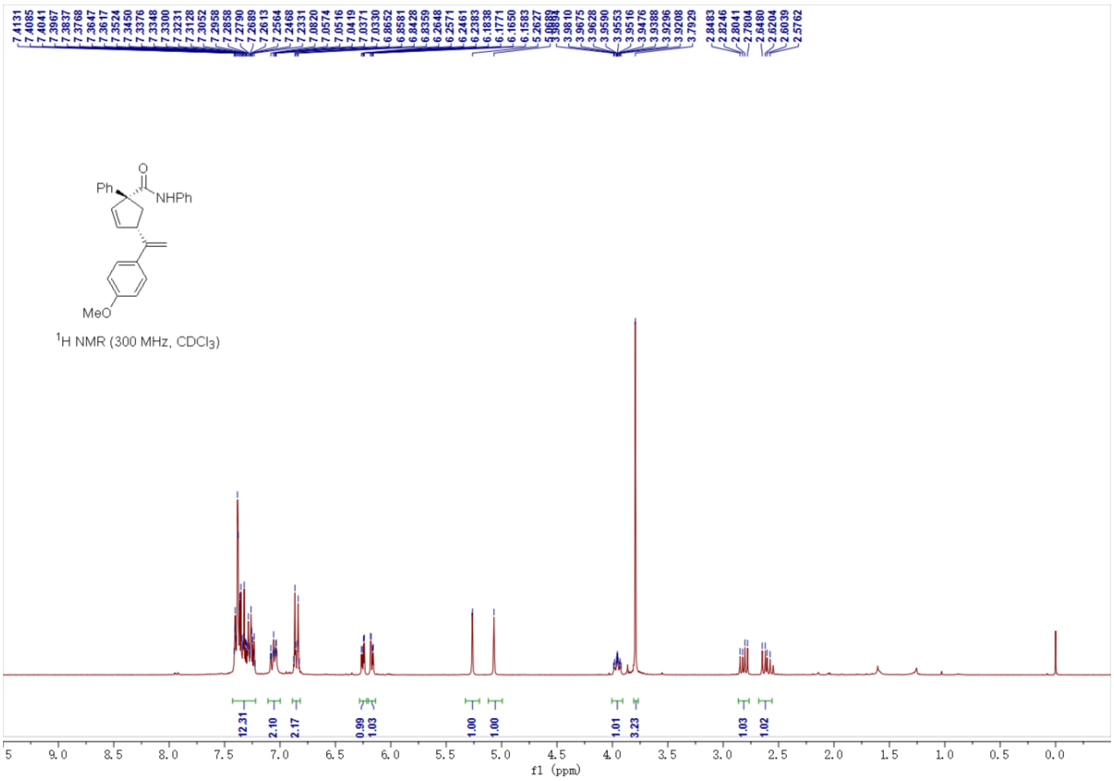

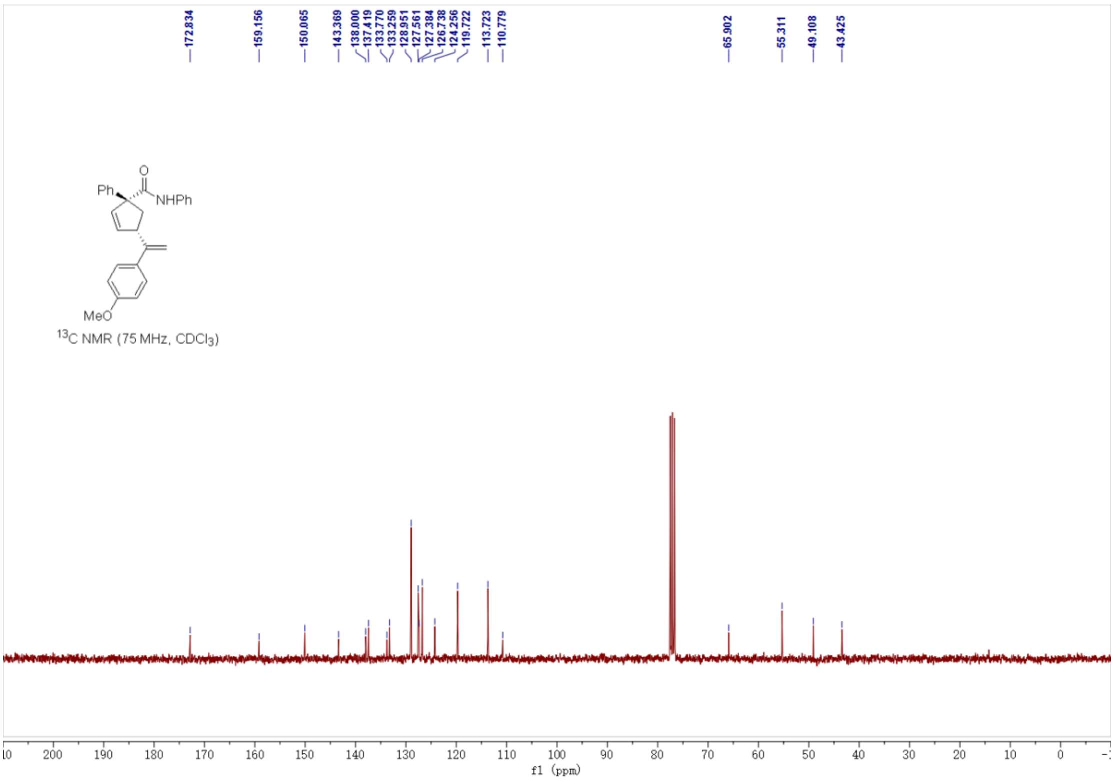

Supplementary Figure 109. ^{1}H and ^{13}C NMR spectra of **3al'**

(1R,4S)-4-((Z)-1,2-diphenylvinyl)-N,1-diphenylcyclopent-2-ene-1-carboxamide (3am)

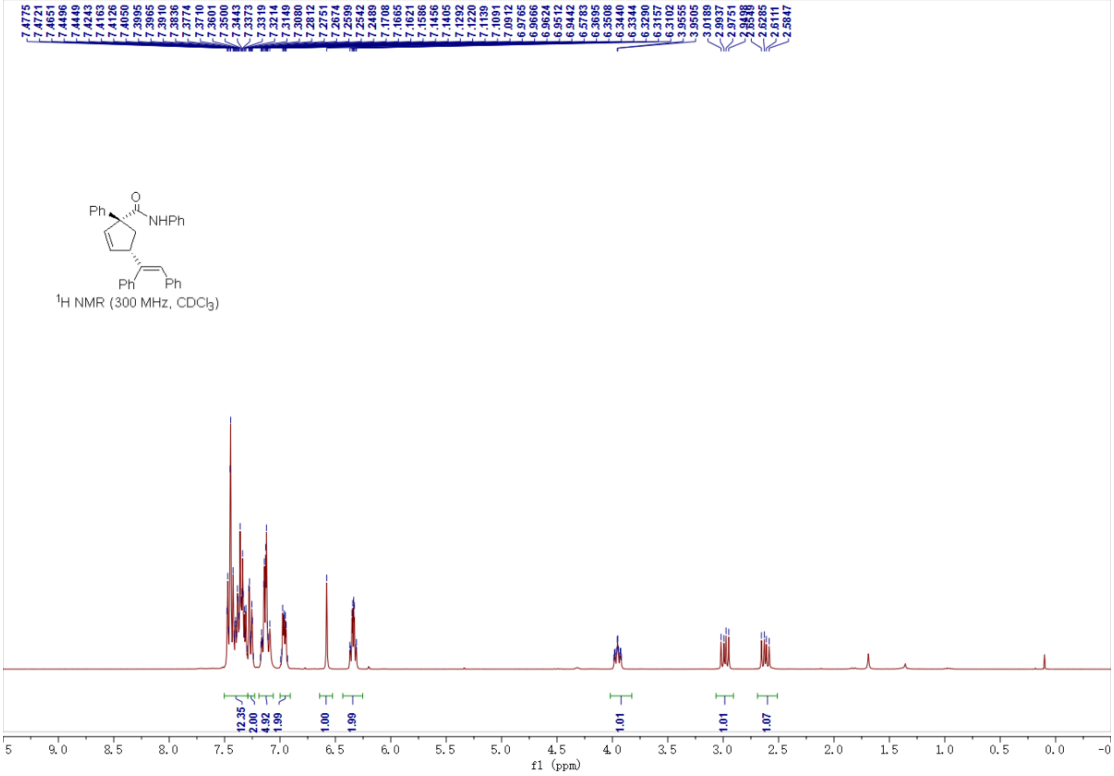

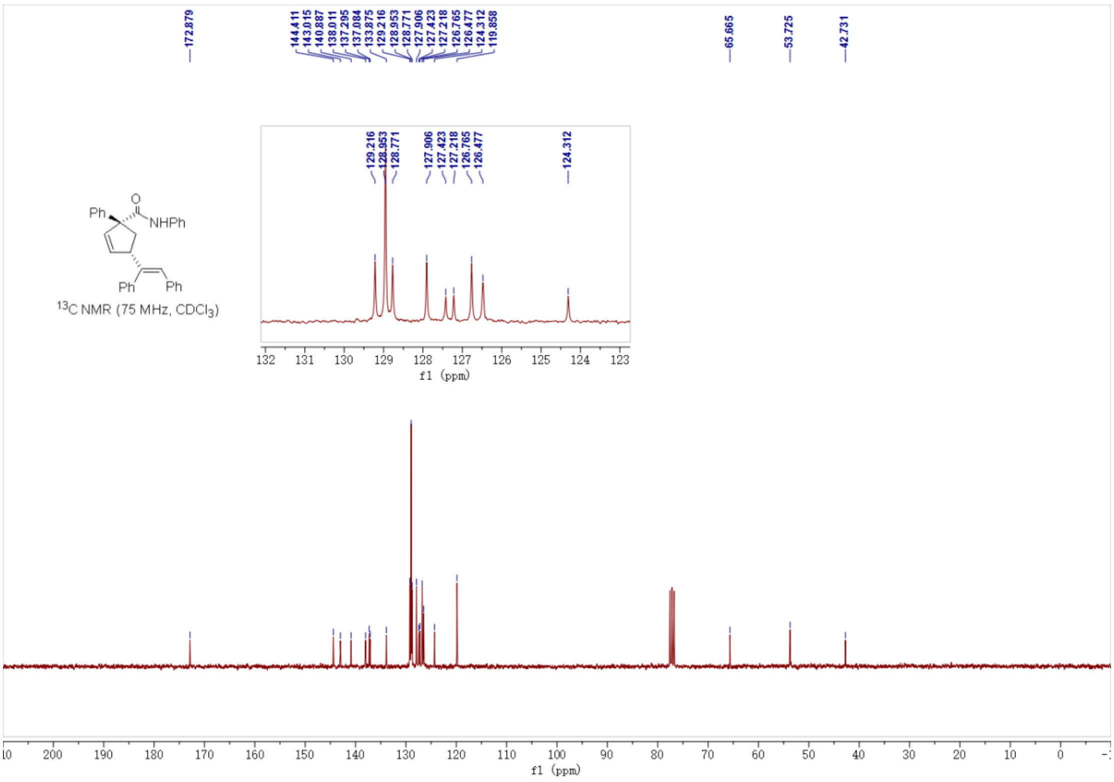

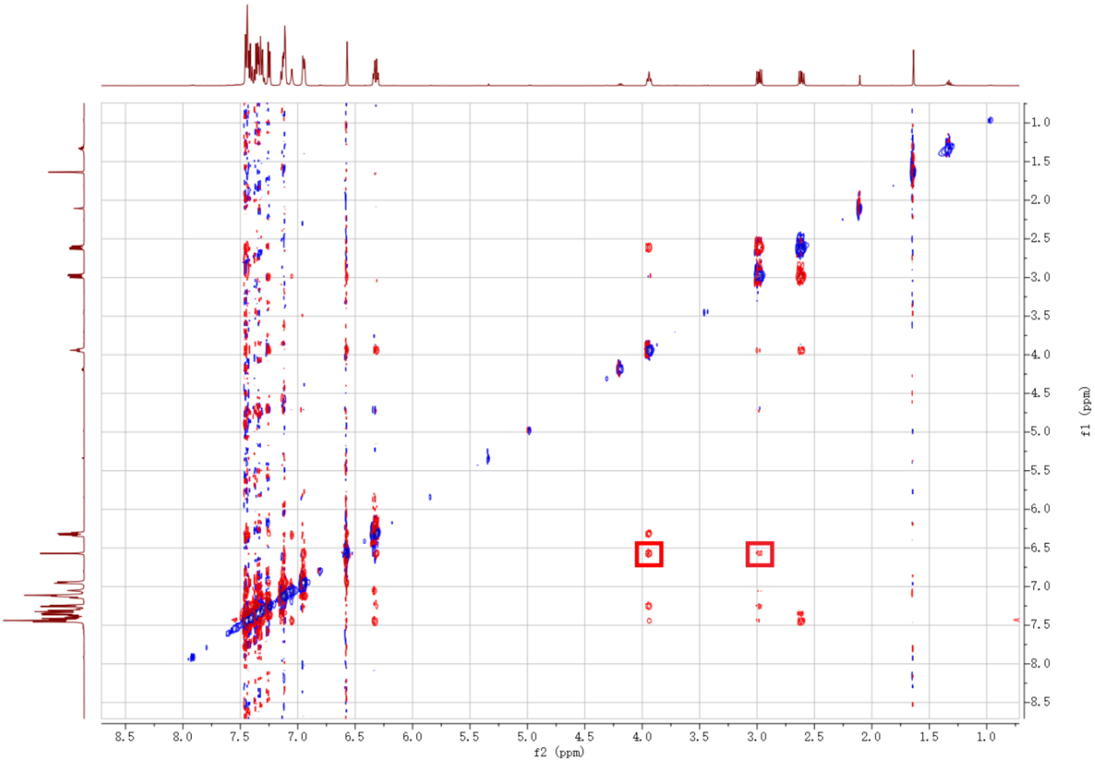

Supplementary Figure 110. ^{1}H and ^{13}C NMR spectra and NOE of **3am**

(1S,3R)-N-phenyl-3-(2-(triisopropylsilyl)ethyl)cyclopentane-1-carboxamide (4)

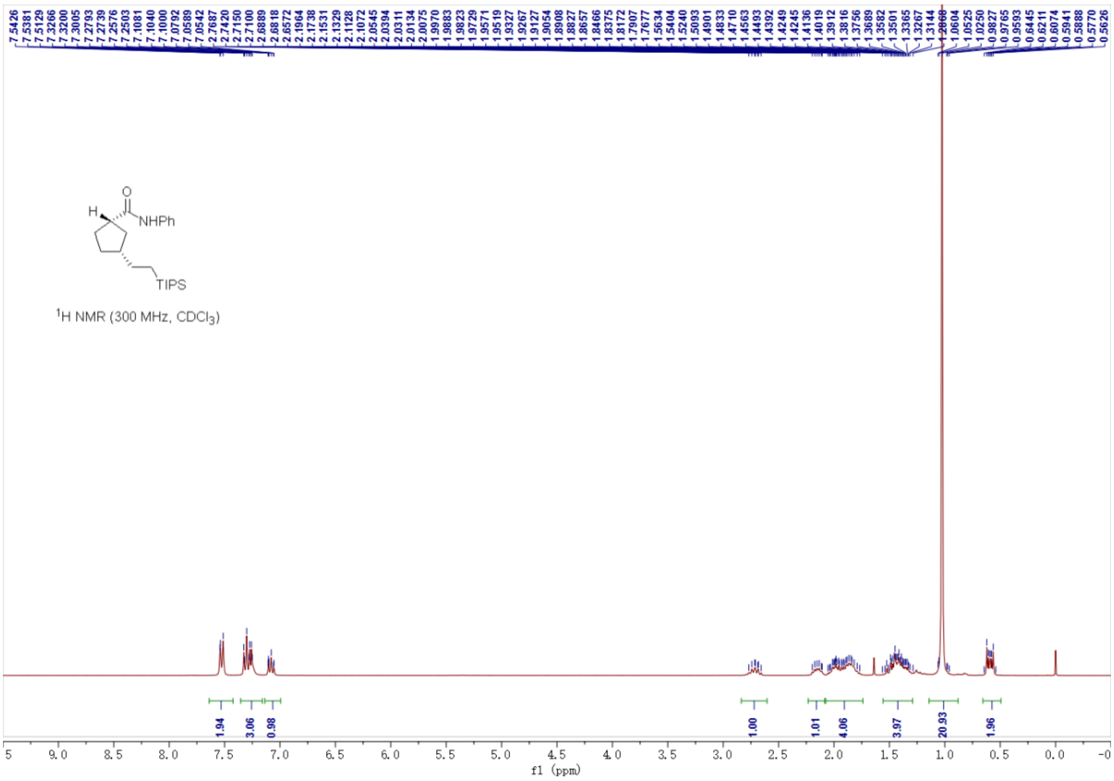

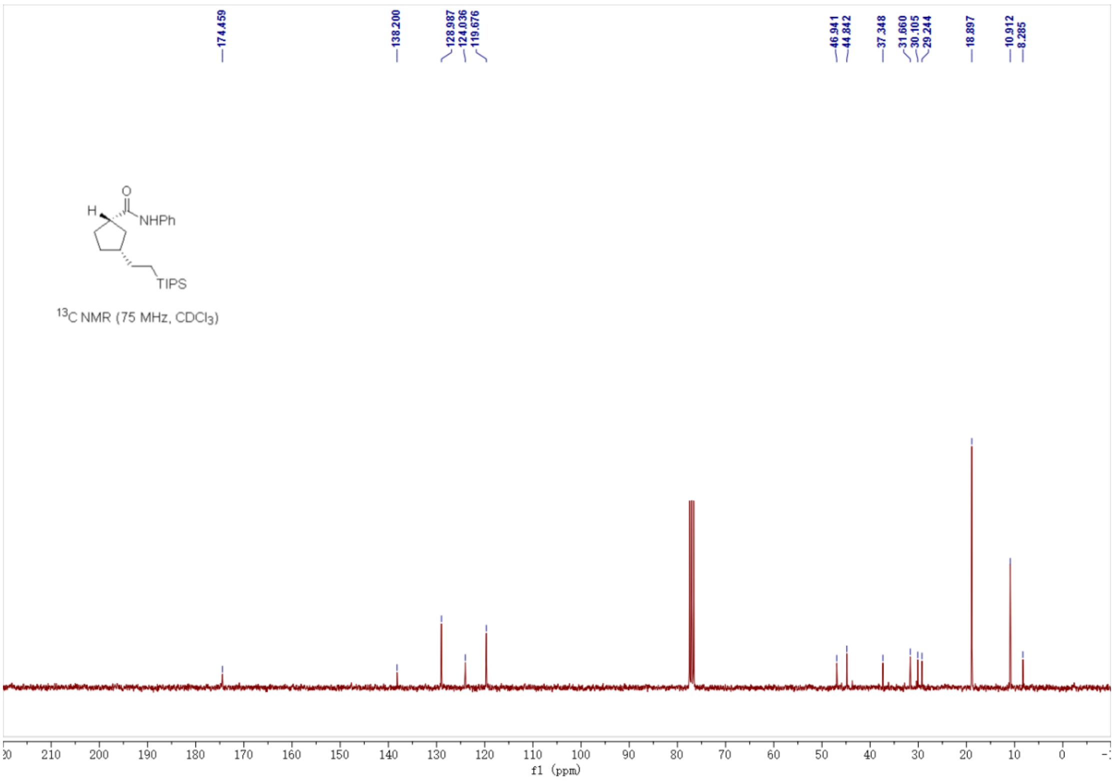

Supplementary Figure 111. ^{1}H and ^{13}C NMR spectra of **4**

***N*-(((1R,4S)-4-((E)-2-(triisopropylsilyl)vinyl)cyclopent-2-en-1-yl)methyl)aniline (5)**

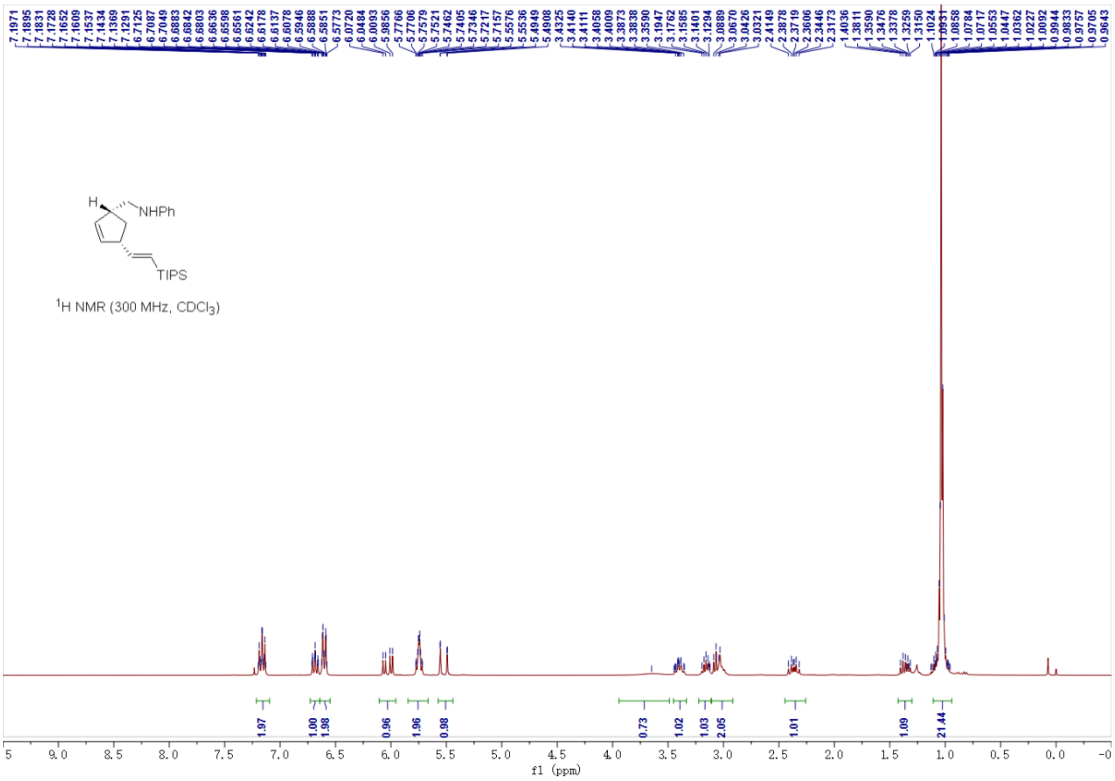

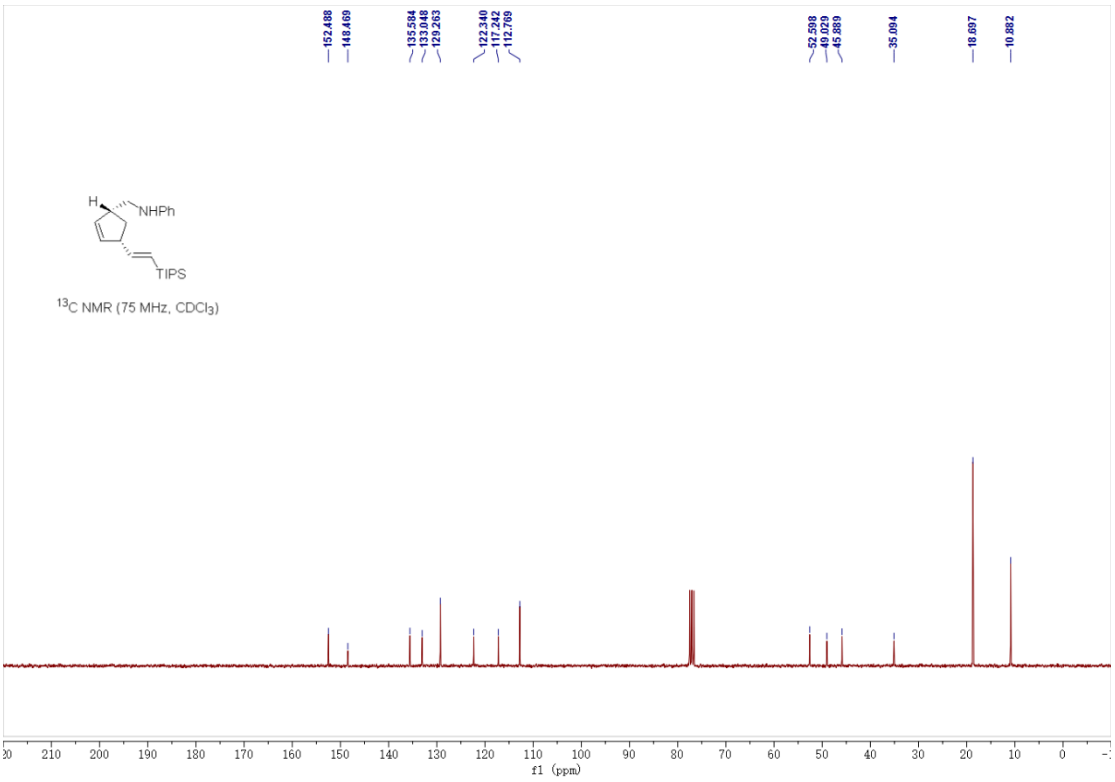

Supplementary Figure 112. ^{1}H and ^{13}C NMR spectra of **5**

(1R,4S)-4-((E)-2-bromovinyl)-N-phenylcyclopent-2-ene-1-carboxamide (6)

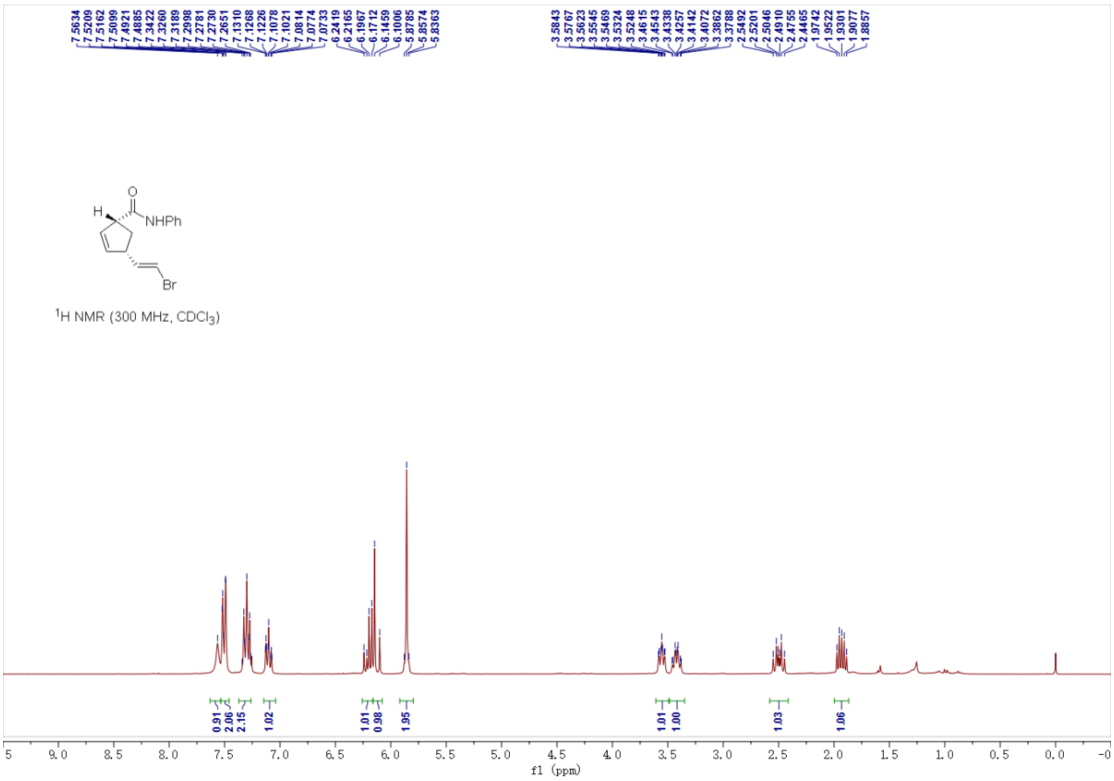

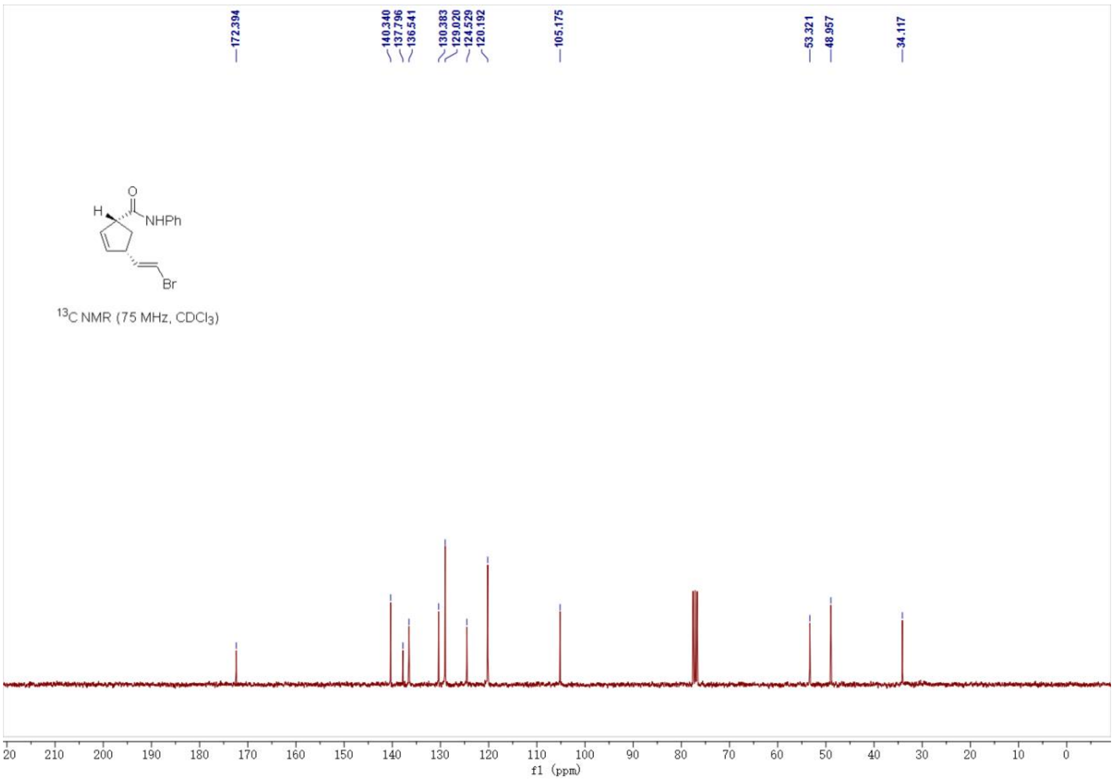

Supplementary Figure 113. ^{1}H and ^{13}C NMR spectra of **6**

(1R,4S)-4-((Z)-2-bromovinyl)-N-phenylcyclopent-2-ene-1-carboxamide (7)

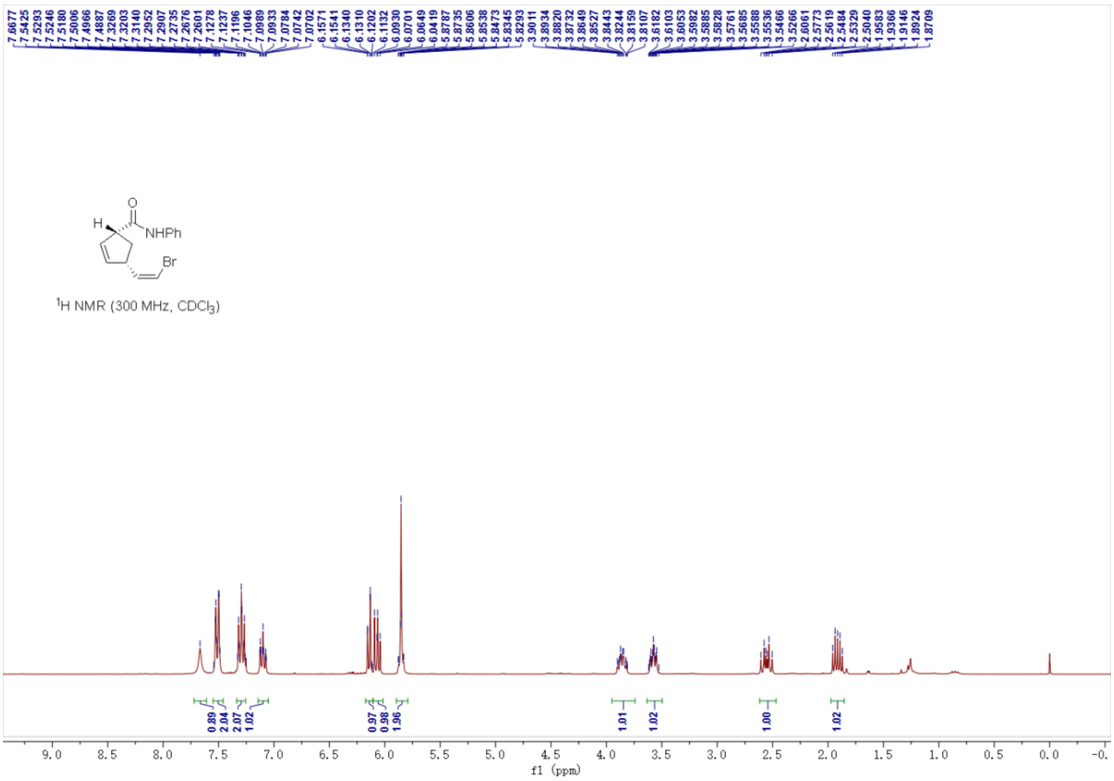

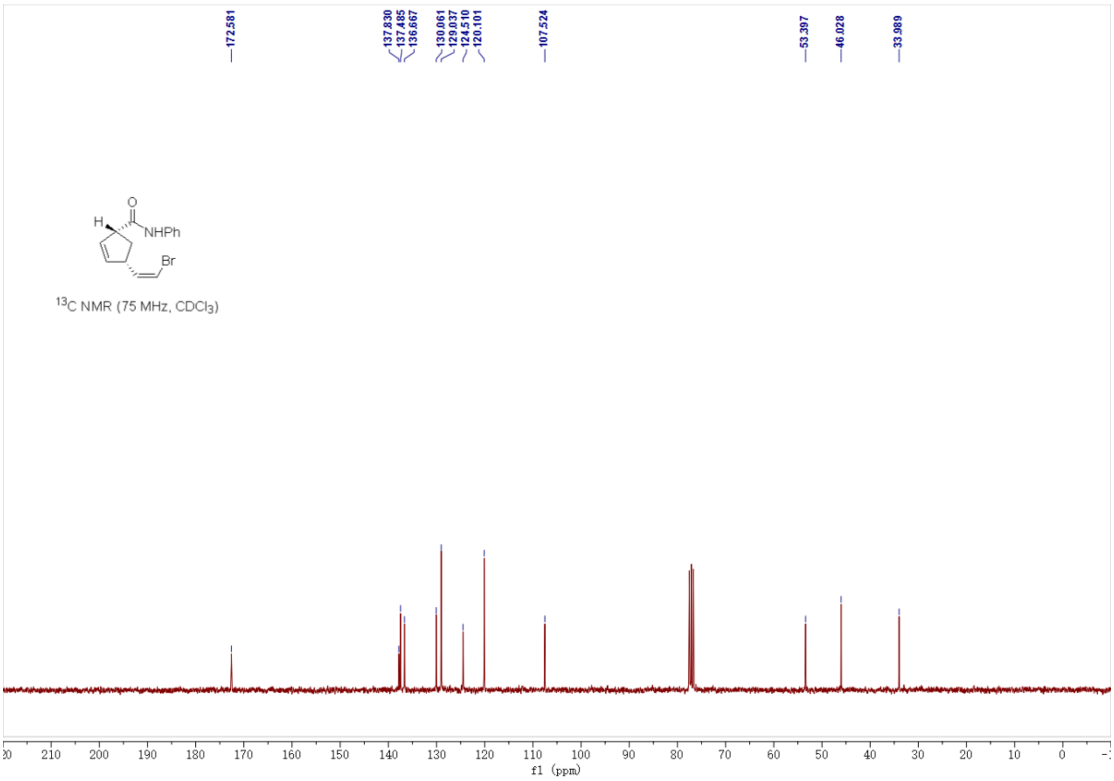

Supplementary Figure 114. ^{1}H and ^{13}C NMR spectra of **7**

(1R,4R)-N,1-diphenyl-4-vinylcyclopent-2-ene-1-carboxamide (8)

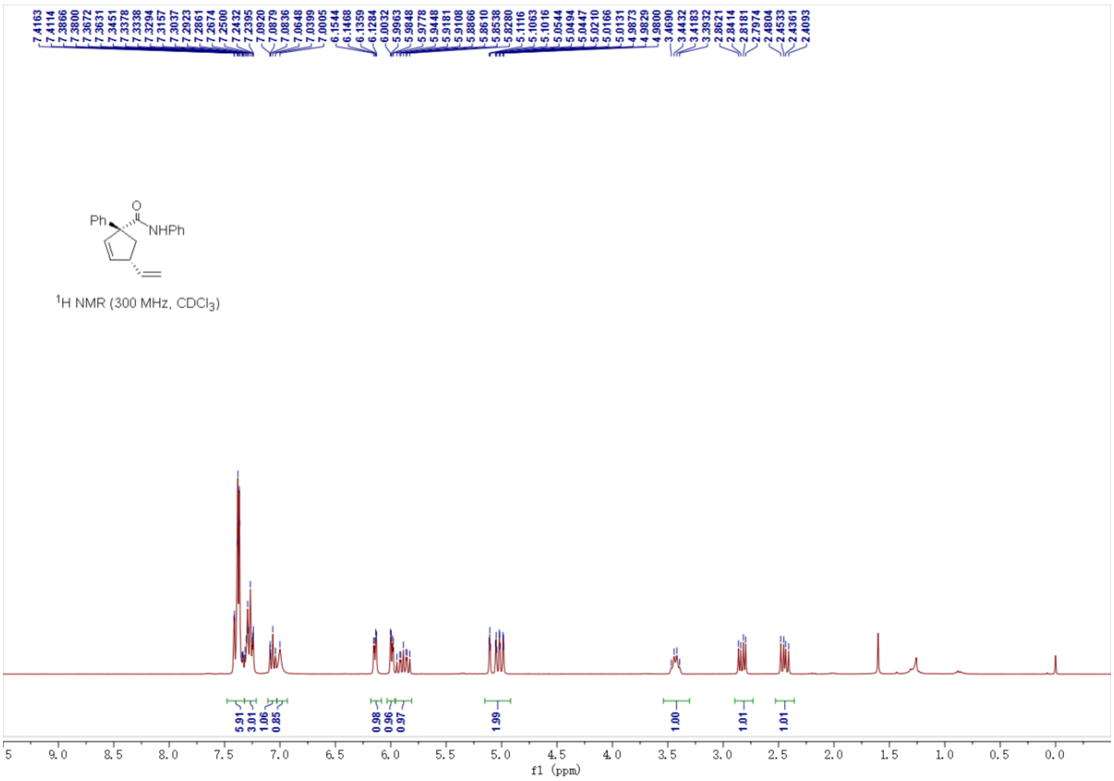

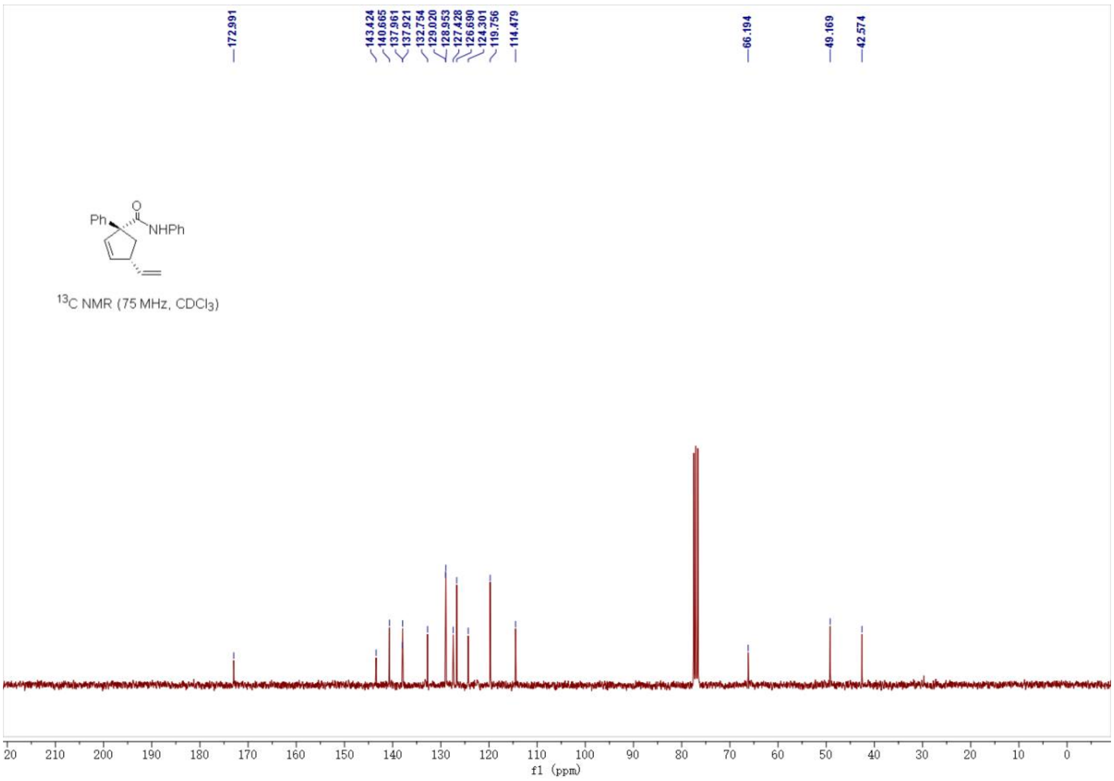

Supplementary Figure 115. ^{1}H and ^{13}C NMR spectra of **8**

(1R,4R)-N,1-diphenyl-4-((E)-styryl)cyclopent-2-ene-1-carboxamide (9)

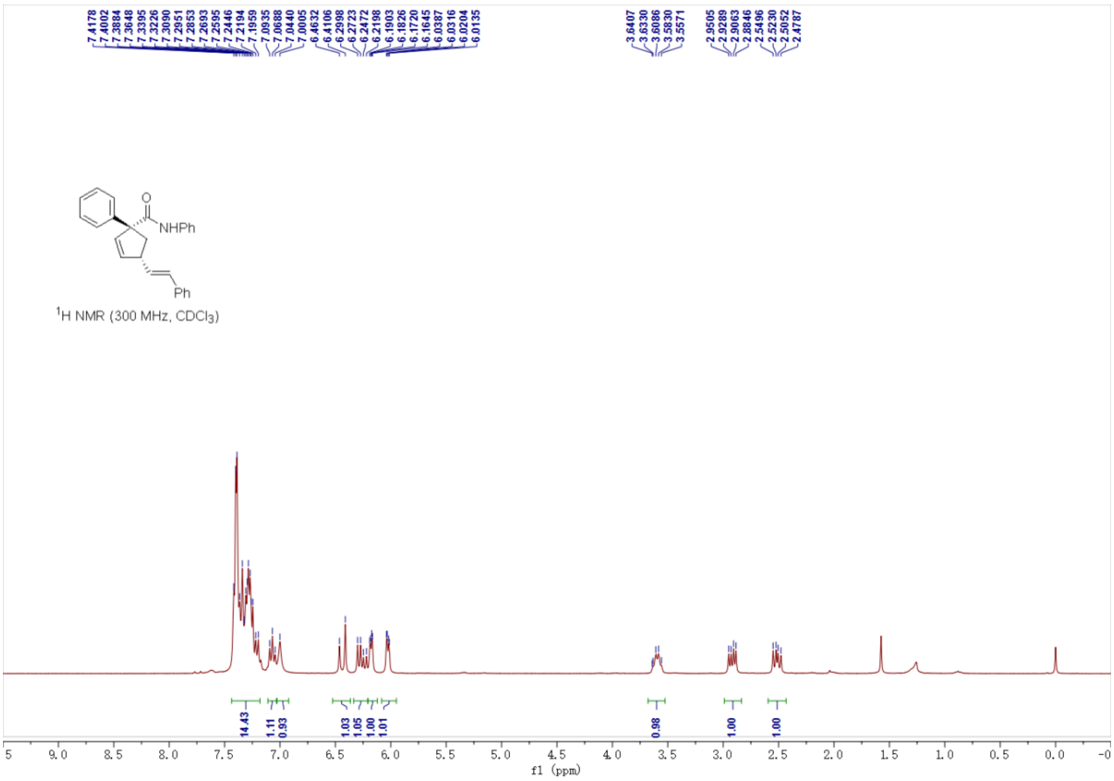

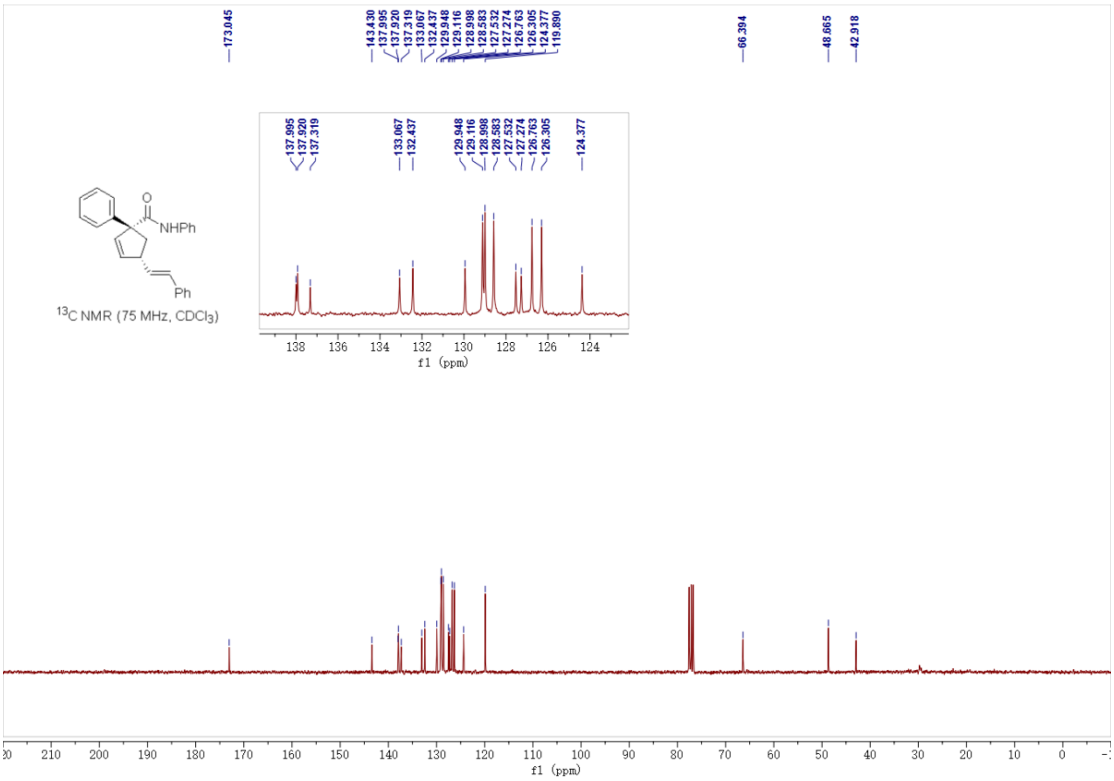

Supplementary Figure 116. ^{1}H and ^{13}C NMR spectra of **9**

(1R,4S)-1-(4-bromophenyl)-4-((E)-2-iodovinyl)-N-phenylcyclopent-2-ene-1-carboxamide (10)

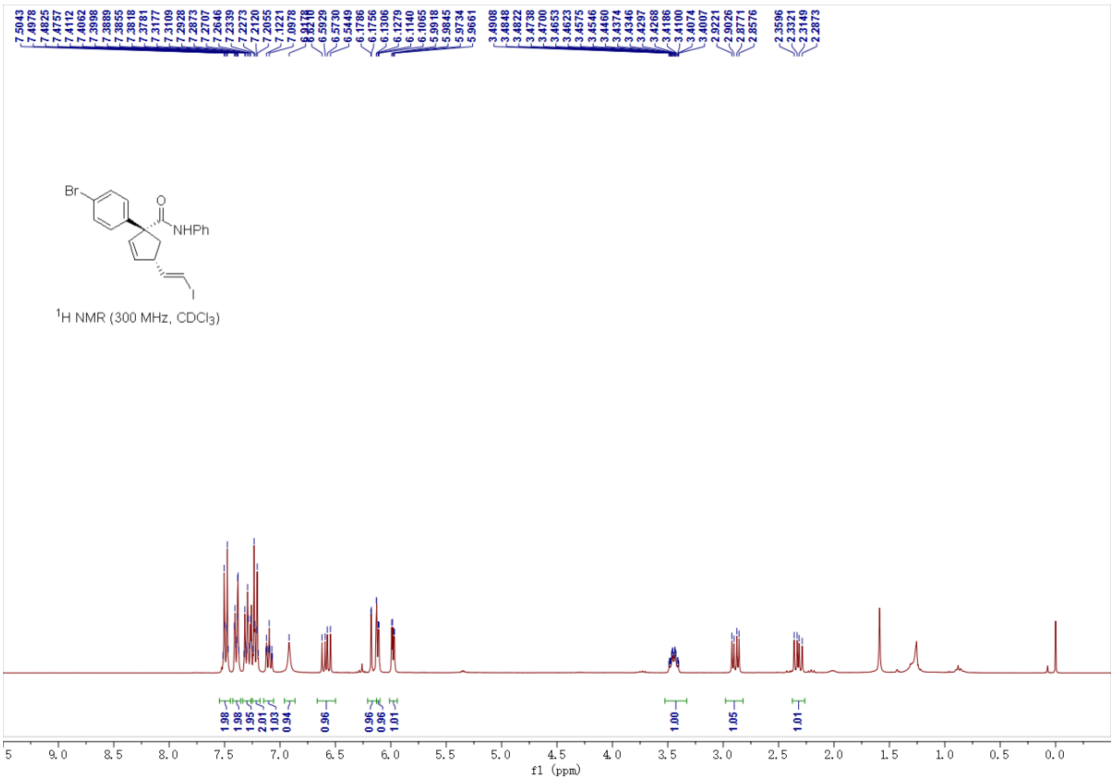

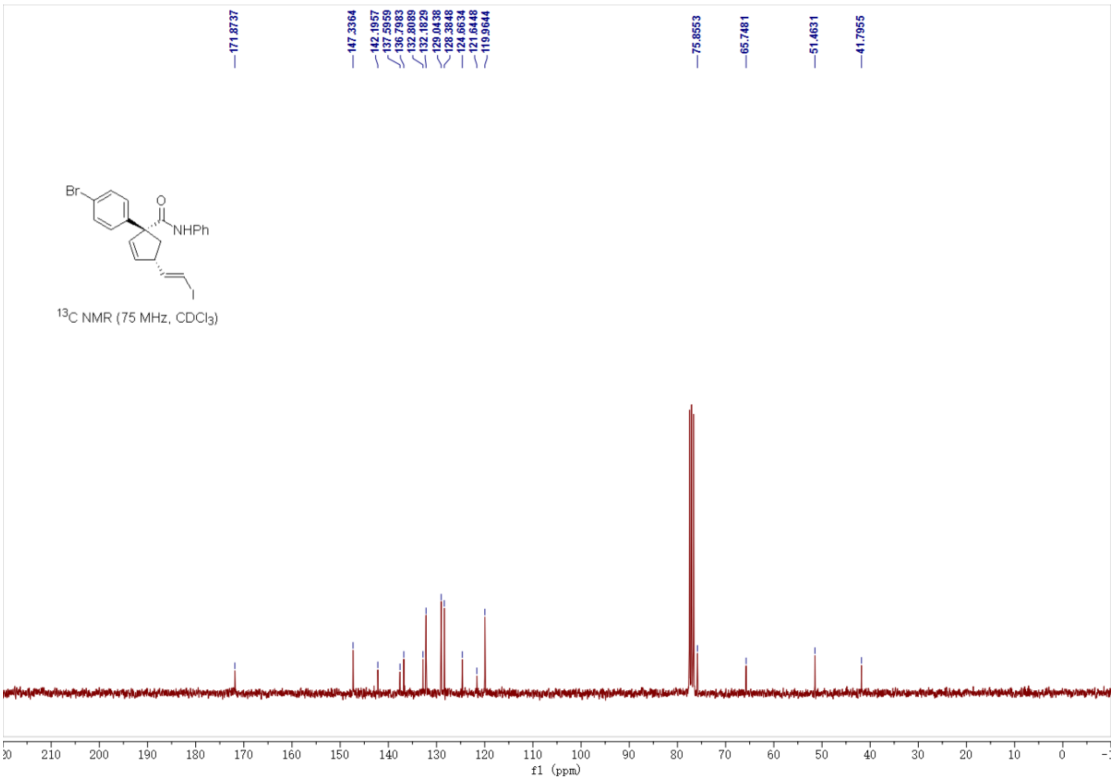

Supplementary Figure 117. ^{1}H and ^{13}C NMR spectra of **10**

Supplementary References

1. Hoang, G. L., Yang, Z.-D., Smith, S. M., Pal, R., Miska, J. L., Pérez, D. E., Pelter, L. S. W., Zeng, X. C. & Takacs, J. M. *Org. Lett*. **17**, 940–943 (2015).
2. Chen, G., Cao, J., Wang, Q. & Zhu, J. Desymmetrization of Prochiral Cyclopentenes Enabled by Enantioselective Palladium-Catalyzed Oxidative Heck Reaction. *Org. Lett.* **22**, 322–325 (2020).
3. Cummings, S. P., Le, T., Fernandez, G. E., Quiambao, L. G. & Stokes, B. J. *J. Am. Chem. Soc.* **138**, 6107–6110 (2016).
4. Sidera, M., Costa, A. M. & Vilarrasa, J. *Org. Lett.* **13**, 4934–4937 (2011).
5. Gudmundsson, H. G., Kuper, C. J., Cornut, D., Urbitsch, F., Elbert, B. L. & Anderson, E. A. *J. Org. Chem*. **84**, 14868–14882 (2019).
