# Supplementary material for: Rhodium-catalyzed intermolecular enantioselective Alder–ene type reaction of cyclopentenes with silylacetylenes
Source: Nat Commun. 2021 Nov 16;12:6627. doi: 10.1038/s41467-021-26955-9 (PMC8595345; doi:10.1038/s41467-021-26955-9)

checkCIF/PLATON report

You have not supplied any structure factors. As a result the full set of tests cannot be run.

THIS REPORT IS FOR GUIDANCE ONLY. IF USED AS PART OF A REVIEW PROCEDURE FOR PUBLICATION, IT SHOULD NOT REPLACE THE EXPERTISE OF AN EXPERIENCED CRYSTALLOGRAPHIC REFEREE.

No syntax errors found.   CIF dictionary   Interpreting this report

Datablock: mo_2021069_0m

```
Bond precision:  C-C = 0.0110 A          Wavelength=0.71073

Cell:             a=9.9538(2)      b=10.1307(3)      c=18.1659(5)
                  alpha=90         beta=90           gamma=90
Temperature:      170 K

                Calculated                Reported
Volume          1831.83(8)                1831.83(8)
Space group     P 21 21 21                P 21 21 21
Hall group      P 2ac 2ab                 P 2ac 2ab
Moiety formula  C20 H17 Br I N O          C20 H17 Br I N O
Sum formula     C20 H17 Br I N O          C20 H17 Br I N O
Mr              494.15                    494.15
Dx,g cm-3       1.792                     1.792
Z               4                         4
Mu (mm-1)       3.935                     3.935
F000            960.0                     960.0
F000'           957.31
h,k,lmax        12,12,22                  12,12,22
Nref            3752[ 2150]               3741
Tmin,Tmax       0.692,0.821               0.462,0.745
Tmin'           0.549

Correction method= # Reported T Limits: Tmin=0.462 Tmax=0.745
AbsCorr = MULTI-SCAN

Data completeness= 1.74/1.00      Theta(max)= 26.395

R(reflections)= 0.0376( 3264)     wR2(reflections)= 0.0916( 3741)

S = 1.070                  Npar= 217
```

The following ALERTS were generated. Each ALERT has the format
test-name_ALERT_alert-type_alert-level.
Click on the hyperlinks for more details of the test.

Alert level C

```
PLAT342_ALERT_3_C Low Bond Precision on  C-C Bonds ...............      0.011 Ang.
```

Alert level G

```
PLAT007_ALERT_5_G Number of Unrefined Donor-H Atoms ..............          1 Report
PLAT012_ALERT_1_G No      _shelx_res_checksum Found in CIF ......     Please Check
PLAT791_ALERT_4_G Model has Chirality at C7          (Sohnke SpGr)          R Verify
PLAT791_ALERT_4_G Model has Chirality at C11         (Sohnke SpGr)          S Verify
PLAT933_ALERT_2_G Number of OMIT Records in Embedded .res File ...          1 Note
```

```
   0 ALERT level A = Most likely a serious problem - resolve or explain
   0 ALERT level B = A potentially serious problem, consider carefully
   1 ALERT level C = Check. Ensure it is not caused by an omission or oversight
   5 ALERT level G = General information/check it is not something unexpected

   1 ALERT type 1 CIF construction/syntax error, inconsistent or missing data
   1 ALERT type 2 Indicator that the structure model may be wrong or deficient
   1 ALERT type 3 Indicator that the structure quality may be low
   2 ALERT type 4 Improvement, methodology, query or suggestion
   1 ALERT type 5 Informative message, check
```

It is advisable to attempt to resolve as many as possible of the alerts in all categories. Often the minor alerts point to easily fixed oversights, errors and omissions in your CIF or refinement strategy, so attention to these fine details can be worthwhile. In order to resolve some of the more serious problems it may be necessary to carry out additional measurements or structure refinements. However, the purpose of your study may justify the reported deviations and the more serious of these should normally be commented upon in the discussion or experimental section of a paper or in the "special_details" fields of the CIF. checkCIF was carefully designed to identify outliers and unusual parameters, but every test has its limitations and alerts that are not important in a particular case may appear. Conversely, the absence of alerts does not guarantee there are no aspects of the results needing attention. It is up to the individual to critically assess their own results and, if necessary, seek expert advice.

Publication of your CIF in IUCr journals

A basic structural check has been run on your CIF. These basic checks will be run on all CIFs submitted for publication in IUCr journals (*Acta Crystallographica*, *Journal of Applied Crystallography*, *Journal of Synchrotron Radiation*); however, if you intend to submit to *Acta Crystallographica Section C* or *E* or *IUCrData*, you should make sure that full publication checks are run on the final version of your CIF prior to submission.

Publication of your CIF in other journals

Please refer to the *Notes for Authors* of the relevant journal for any special instructions relating to CIF submission.

PLATON version of 05/12/2020; check.def file version of 05/12/2020

Datablock mo_2021069_0m - ellipsoid plot

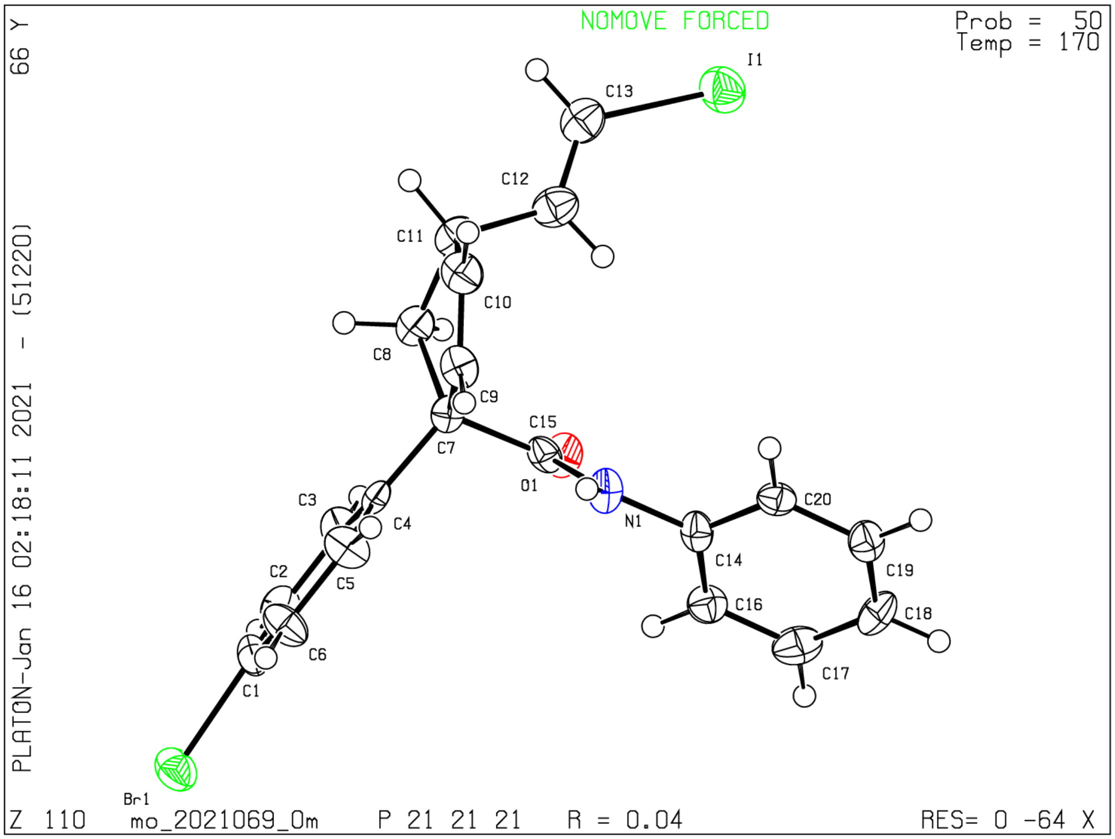

Supplement: Supplementary file 2 — Source Data [file 41467_2021_26955_MOESM2_ESM.pdf]
